# UFFizi: a generic platform for ranking informative features

**DOI:** 10.1186/1471-2105-11-300

**Published:** 2010-06-03

**Authors:** Assaf Gottlieb, Roy Varshavsky, Michal Linial, David Horn

**Affiliations:** 1 School of Physics and Astronomy, Tel Aviv University, Ramat Aviv, Tel Aviv, Israel; 2 Israel Innovation Labs, Microsoft Israel R&D Center, Herzeliya, Israel; 3 Department of Biological Chemistry, Institute of Life Sciences, The Hebrew University of Jerusalem, Jerusalem, Israel

## Abstract

**Background:**

Feature selection is an important pre-processing task in the analysis of complex data. Selecting an appropriate subset of features can improve classification or clustering and lead to better understanding of the data. An important example is that of finding an informative group of genes out of thousands that appear in gene-expression analysis. Numerous supervised methods have been suggested but only a few unsupervised ones exist. Unsupervised Feature Filtering (UFF) is such a method, based on an entropy measure of Singular Value Decomposition (SVD), ranking features and selecting a group of preferred ones.

**Results:**

We analyze the statistical properties of UFF and present an efficient approximation for the calculation of its entropy measure. This allows us to develop a web-tool that implements the UFF algorithm. We propose novel criteria to indicate whether a considered dataset is amenable to feature selection by UFF. Relying on formalism similar to UFF we propose also an Unsupervised Detection of Outliers (UDO) method, providing a novel definition of outliers and producing a measure to rank the "outlier-degree" of an instance.

Our methods are demonstrated on gene and microRNA expression datasets, covering viral infection disease and cancer. We apply UFFizi to select genes from these datasets and discuss their biological and medical relevance.

**Conclusions:**

Statistical properties extracted from the UFF algorithm can distinguish selected features from others. UFFizi is a framework that is based on the UFF algorithm and it is applicable for a wide range of diseases. The framework is also implemented as a web-tool.

The web-tool is available at: http://adios.tau.ac.il/UFFizi

## Background

The present information age is characterized by exponentially increasing data, e.g. in numbers of documents and in records of various kinds or biological data. Improved experimental techniques, such as high throughput methods in biology, allow for the measurement of thousands of features (genes) for each instance (single gene-expression microarray per patient). This leads to a flood of data, whose analysis calls for preprocessing in order to reduce noise and enhance the signal through dimensionality reduction. This is important for both enabling the application of various categorization techniques and allowing for biological inference from the data.

Dimensionality reduction algorithms are usually categorized as extraction or selection methods. Feature extraction transforms all features into a lower dimension space, while feature selection selects a subset of the original features. A benefit of the latter is the ability to attach meaning to the selected features. This is important both for exploration of the biological reality and for preparing a more concise experimental layout. The method to be studied here is categorized as feature selection.

It is customary to divide feature selection methods into two types: supervised, in which a target function is known and one tries to rank features or optimize some objective function relative to it, and unsupervised, in which one has no additional information regarding the instances. In practice, the abundance of unlabeled data or data that might posses multiple possible labeling, calls for an unsupervised approach.

While supervised feature selection methods are abundant [1], unsupervised methods are scarce, most of them tested on labeled data [2]. Nevertheless, unsupervised feature selection methods may play an important role even in supervised cases. Being unbiased by the labeling of the instances, unsupervised feature selection can be used as a preprocessing tool for supervised learning algorithms providing reduction of overfitting (for a comprehensive review we refer to [2]). As described in [3], feature selection from unsupervised data can be applied at three different stages: before, during and after clustering. Methods that operate before clustering are referred to as filter methods. Common methods of unsupervised feature filtering rank features according to either (1) their non-zero loadings in the first principal components [4], (2) their normalized range,(3) entropy or (4) variance of the feature as calculated from its values on all instances [2,5]. All these methods estimate the importance of each feature independently of all others.

Our Unsupervised Feature Filtering (UFF) algorithm [6] differs from aforementioned methods in that it ranks features based on a criterion that involves all other features. It also provides a natural cutoff for selecting the number of features. We have also previously showed that UFF also selects stable feature sets under perturbations [7]. Our aim in this article is to introduce a new framework, based on the UFF. We (1) explore the properties of UFF and the features it selects, (2) introduce a faster approximate version, (3) suggest indicators for the ability to apply the method to certain datasets and (4) extend it by proposing a method called Unsupervised Instance Selection (UIS) for inspecting and eliminating potential outlier instances. A faster version of UFF, together with identification of indicators for the ability to apply the method to different datasets enables the implementation of UFF as a web-tool. The performance of the UFF is shown to surpass commonly used unsupervised filtering methods (e.g. variance, feature entropy) for the datasets used in this study. These findings are consistent with the findings reported in [6].

In the Results section, we explore the properties of UFF on example datasets, introduce a faster algorithm for UFF and analyze which datasets can be evaluated successfully by the UFF method. We then describe the UDO method and provide biological insights on gene and microRNA expression from a wide range of diseased states.

## Results and Discussion

### Analyzing and Improving UFF

In this section, we present analysis of UFF selected features and provide improvements and extensions to the algorithm. The improvements include (i) Faster version of the algorithm and (ii) Addition of a criterion for assessing the quality of the results provided by UFF. We further extend the algorithm by introducing the Unsupervised Detection of Outliers (UDO).

### Properties of selected features

**Figure 1:**
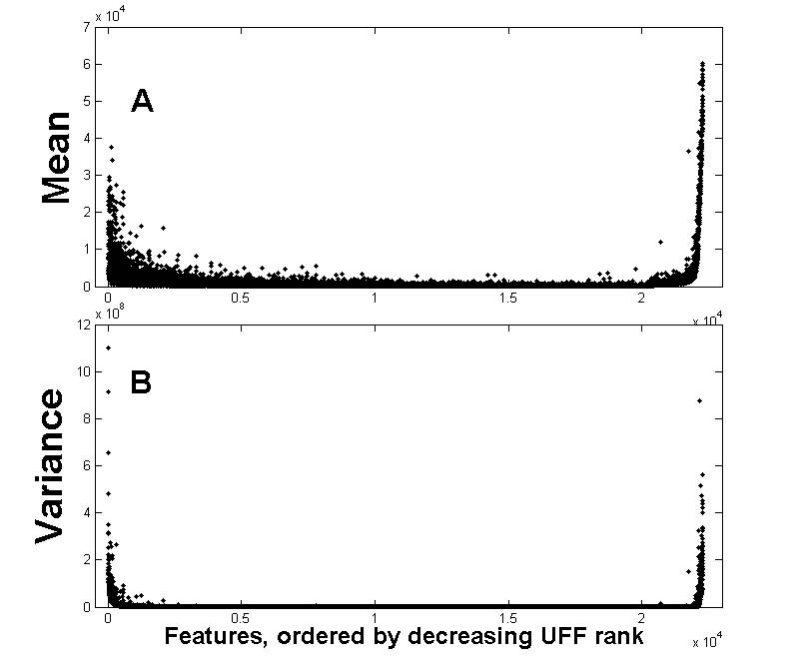
**First moments of the melanoma dataset genes**. (A) Mean and (B) variance of the melanoma dataset (X axis refers to genes ordered according to UFF score).

**Figure 2:**
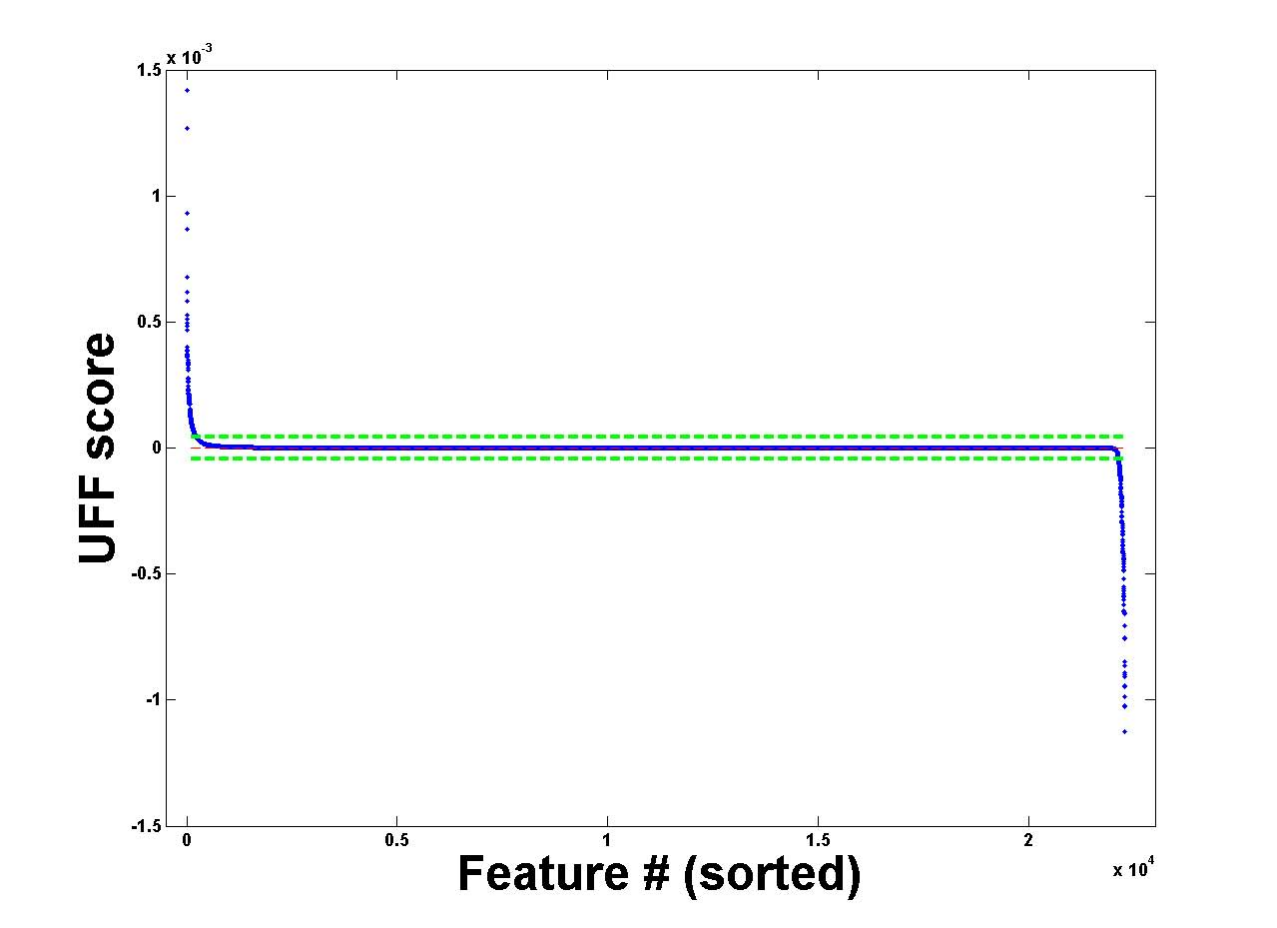
**UFF Scores of the melanoma dataset genes**. UFF Scores of the 22283 genes of the melanoma dataset, ordered by decreasing scores. Dashed lines represent mean(score) ± std(score).

We investigated the general properties of features selected by UFF, by studying their statistical properties. We demonstrate these properties on the melanoma gene expression dataset (see Methods). Figure 1 displays the mean (A) and variance (B) of all features (as measured on all instances), for the melanoma dataset. The features are ordered by their UFF rank, which is displayed in Figure 2. Dotted lines, denoting the mean (score) ± one standard deviation, supply the separation between the positive (group 1), neutral (group 2) and negative (group 3) score features (Methods). Most features belonging to the second (neutral) group possess low mean and variance. It is evident that both the positive score features and the negative score features have high mean (in general high absolute values of mean) and variance. This explains a major difference between UFF and the Variance Selection method: while UFF selects features from group 1, Variance Selection chooses features from both groups 1 and 3. It should be noted that if datasets of this nature (e.g. gene-expression) undergo standardizing operations, UFF selection may be meaningless.

**Figure 3:**
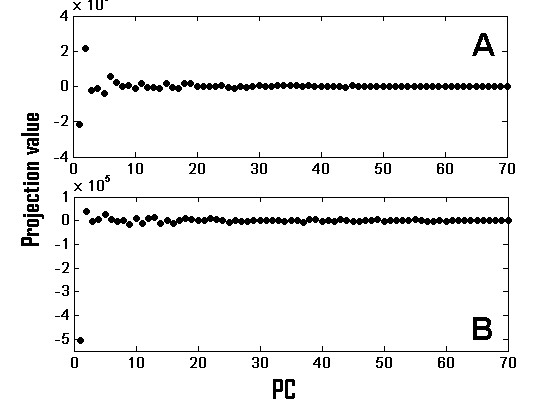
**Projection of features on the principal components**. Projection on the 70 principal components of a typical - (A) positive score and (B) negative score - feature from the melanoma dataset. Note the outstanding value (approximately -5) of PC1 in B.

An important difference between the positive (group 1) and negative (group 3) features is displayed in Figure 3. This figure shows the projection of typical positive and negative features (A and B, respectively) on the SVD eigenvectors (or principal components, PCs) of the original data matrix. Positive score features have more evenly distributed projections on the PCs relative to the negative score features, which project most strongly on the first PC. It is the latter property that explains the negative score: by preferring the leading principal component these features decrease SVD-entropy. We present in the Appendix a proof showing that when a feature lies only on the first PC, it is bound to have a negative score.

The differences in projection on the principal components between the positive and negative scored features, may provide an explanation for the difference between our approach and the sparse-PCA approach [4]. The latter selects genes that correlate mainly with the leading PC, while UFF prefers a wider distribution.

Finally we observe that negative score features have skewness close to zero and kurtosis close to three. Hence we conclude that negative score features possess wide Gaussian distributions, which can be regarded as baring no indicative signal over the instances. These noisy features are discarded by UFF but selected by Variance Selection, which explains their inferior results demonstrated in [6]

### Fast UFF

In order to obtain the UFF ranking of features one performs *M *times the SVD evaluation, where *M *is the number of features. This has the complexity of O(M*min(N,M)^3^) (see [6]). The data matrix *A *of *M *features by *N *instances is often represented by its SVD transformation A = USV^T^, where U and V are unitary and S is the diagonal matrix of the singular values. The associated Gram matrix *C = A*^*T*^*A*, of size *NxN*, can then be written as C = VS^2^V^T^, with eigenvalues that are the squares of the singular values of *A *and thus can be used directly to calculate the SVD-entropy. Removing a row from *A*, i.e. removing the feature *f*^*k *^of length *N*, the Gram matrix *C *changes to(1)

We assume that removal of one feature can be regarded as a small perturbation, an assumption which generally holds for a large enough number of features. The singular values can be approximated by using the eigenvectors of the Gram matrix *C *on the new matrix *C'*. Plugging into equation (1), the changed SVD entropy is:(2)

An extended formulation is given in the Appendix.

This approximation reduces the complexity to O(M*N^2^) leading to considerable faster calculations. Table 1 compares the running times of fast UFF vs. regular UFF for three of the datasets used in this paper. As can be seen, the reduction in running time is substantial, allowing for an online computation.

**Table 1:** Comparison of running time between regular and fast UFF

Dataset	Regular UFF Matlab	Fast UFF Matlab	Regular UFF C++	Fast UFF C++
Melanoma Size = [69 × 22283]	15.3	0.76	94.6	0.5

HIV Size = [40 × 22283]	5.4	0.63	26.7	0.19

Hepatitis C Size = [78 × 54675]	45.3	1.9	300.2	1.5

Running was performed both on Matlab and using a compiled C++ executable. All runs were performed on a single computer. Time is specified in seconds (Difference in running time of Matlab and C++ UFF implementation is due to difference in SVD calculation times. C++ implementation uses external linear algebra package - Lapack++).

The quality of the approximation lies in the assumption of small perturbations. In order to test whether this assumption holds for a given dataset, we inspect the SVD entropy of the matrix, defined to lie between 0 and 1 (see Methods). For most data-sets that we studied it is smaller than 0.1. Such a small value of the entropy guarantees that only a few eigenvalues (principal components) are of importance, and the removal of a single feature is indeed a small perturbation assuring the validity of the approximation (equation 2). In two of the studied datasets (GBM and OV microRNA) the SVD entropy is large (0.59 and 0.34 correspondingly), putting the approximation (equation 2) in doubt. In both cases one should therefore resort to the regular UFF calculation to obtain reliable results

Fast UFF allows for the analysis of much larger datasets. Moreover it enables incorporating this algorithm in a web-based tool. Computationally, it allows for a distributed evaluation of UFF scores, once the eigenvectors of the Gram matrix C are obtained. The calculation of the SVD entropy of the matrix is incorporated into the UFFizi web tool, initiating a warning when the results of the fast UFF might deviate substantially from the regular UFF.

### When is UFF applicable

**Figure 4:**
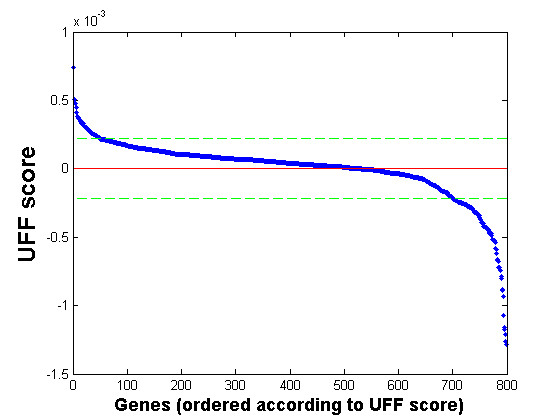
**UFF scores for a non-applicable dataset**. UFF Scores of the Spellman cell-cycle dataset, ordered by decreasing UFF score.

While UFF works very well on many datasets, including most gene-expression data we have analyzed, we have found datasets where selection according to UFF is not effective. Figure 4 presents such an example using a dataset of pre-selected cell-cycle regulated genes. On such a dataset, UFF did not lead to improved clustering. We note that the distribution of score values in Figure 4 is somewhat different from Figure 2. In particular, group 2 features display large variance among their scores.

Working with more than twenty datasets from different domains, we have found measures that allow for separation between datasets on which UFF is effective from datasets in which it is not. One such measure is the normalized entropy of the squares of UFF scores. This, as well as another measure, is presented in the supplementary appendix. They allow for a prior estimate on whether UFF selected features should be used. These measures, formulated in the supplementary appendix, are incorporated into our web-tool, providing a confidence level for relying on UFF results.

### Unsupervised Detection of Outliers (UDO)

Outliers are typically defined as instances that differ significantly from other instances in the data (for extensive surveys, see [8,9]). Detecting such outlier instances may be desirable in certain cases, e.g. when there is a suspicion of faulty or unreliable measurements or for detecting rare events. A multitude of methods for unsupervised outlier detection have been proposed. Most relate to one of two approaches: (1) model based, in which a model is fit to the data and outliers are the ones deviating from the model [10,11], (2) Distance-based methods, which find instances lying far from all instances, nearest instances, or nearby clusters [12-18]. We present here an alternative definition and a method to detect such outliers, based on the UFF framework.

The data-matrix A contains information on instances in terms of features and features in terms of instances, and the singular values are common to both. One may therefore consider a 'leave-one-out' measure applied to instances. This is the Unsupervised Detection of Outliers (UDO) method, to be studied here. UDO identifies instances that, when removed, decrease the entropy of the dataset and thus provide a more homogeneous dataset. Recognizing these entropy-increasing instances as outliers provides a natural definition for an "outlier-degree". UDO attaches to each instance the amount of decrease of the SVD entropy, which is considered the global measure of the "outlier-degree" of each instance in the dataset. As in the UFF method, a threshold of one standard deviation (std) above the mean may be applied to assess the number of such outliers. UDO is a data-driven method, making no prior assumption regarding the distribution of the data such as model-based methods. It is not restricted by small sample size datasets which prohibit creation of valid distribution assessments. It is also different from distance-based outlier detection schemes in that it assesses the influence of instance removal on the entire dataset rather than the mere location in feature space of the instance relative to other instances. In contrast to the Donoho-Stanhel estimator [12], which assesses the "outlier-degree" of an instance relative to one selected direction in feature space, UDO estimates it on all eigenvectors at once. UDO in this sense emphasizes directions along which other instances are relatively comparable. We note that in datasets of relatively low SVD entropy, the correlation between the UDO ranking and the popular outlier detection method of the k^th^-NN ranking [16] is relatively high (0.61 and 0.82 for the melanoma and HIV datasets respectively, k = 5). This can be explained by noting that removal of an instance in such datasets does not alter the leading eigenvectors substantially and UDO thus selects the high-entropy instances that reside mainly farthest along these eigenvectors. In high SVD entropy datasets (e.g. the two microRNA datasets in this paper), the correlation between the two different methods is essentially zero.

Since outlier defining criterion and the methods implementing them are intertwined, evaluation of each method turns often into subjective inspection of the outliers. We note that in the HIV dataset for which we have some clinical information, the first 4 selected instances (out of 5 selected by UDO) are samples of two individuals (containing both CD4+ and CD8+ T cells). The two leading outlier instances belong to the same individual, possessing an HIV infection at a very preliminary stage (~1 month), possibly explaining high divergence of measurements from individuals with longer periods of HIV infection.

### Selected Datasets

In this section we present novel results obtained by applying UFF to gene-expression and microRNA (miRNA) expression datasets.

### Melanoma - UFF selected genes

The melanoma dataset is used for demonstrating the different traits of UFF. Running UFF on this dataset, we obtain 231 genes. The top ranked genes include Stratifin, Keratin 14, Keratin 1 and Loricrin, mutations in which are related to skin cancer and other skin diseases [19-22]. Enrichment analysis includes terms having Bonferroni score < 0.05. GO Enrichment analysis of the selected genes includes functions of biological processes such as ectoderm and epidermis development, homophilic cell adhesion, keratinocyte differentiation and melanin biosynthetic process. Cellular compartments enrichment includes intermediate filament, extracellular region and melanosome. Interestingly, GO molecular function enrichment show various metal ion binding, including copper, cadmium and calcium, all having relations to the tumor suppressor protein p53 [23-25]. Enriched pathways include cell communication, antigen processing and presentation and also breast cancer estrogen signaling. Human phenotype analysis reveals enrichment for palmoplantar hyperkeratosis, keratinization, skin and integument abnormalities. The list of UFF selected genes is provided in Additional file 1, Table S1. The full list of GO enrichment terms is provided in Additional file 2, Table S1.

Talantov, et al. (2005) performed clustering analysis on this dataset, using a filtered list of 15,795 genes. They did not obtain perfect separation between melanoma and benign tumors or normal tissues (obtaining Jaccard score [26] of 0.74). Using UFF selected genes and the Quantum Clustering algorithm [27], we were able to correctly split melanoma from benign tissues, while identifying two clusters in the melanoma samples similar to the ones identified by [28] (Jaccard score of 0.85)32 of UFF selected genes appear also in the 439 differentially expressed genes of [28] (p-value = e^-12^) and 10 out of 33 differentially expressed genes with high fold change (p-value < e^-12^).

**Figure 5:**
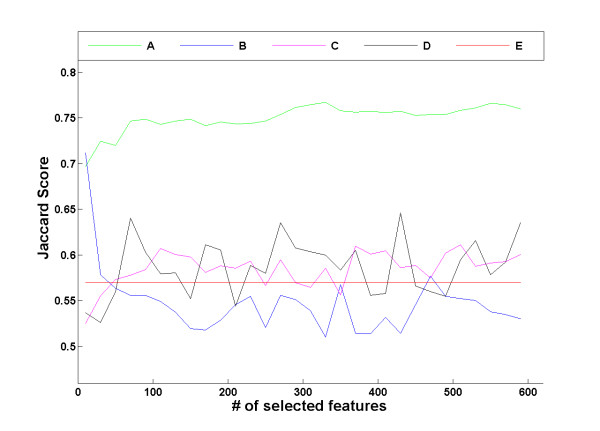
**Comparison of UFF with other selection methods on the melanoma dataset**. Mean Jaccard scores of clustering results for different selection methods on the melanoma dataset. Tested methods include (A) UFF, (B) Variance, (C) Feature entropy, (D) Random selection and (E) All features.

Figure 5 compares the clustering results in terms of Jaccard score using UFF selected genes for different thresholds, with genes selected using variance, feature entropy and random selection and using all the genes (see Methods). It is evident that UFF features provide better clustering results than either selection method or compared to using all the genes for all thresholds (with an exception for the top 10 genes, where variance selection has slightly better Jaccard score). Error bars were removed for clarity. Additional file 3, Figure S1 displays the same comparison with error bars, including a comparison with selection of the first eigenvectors, computed using SVD.

Quantum Clustering results are provided in Additional file 4, Table S1.

### HIV - UFF Selected genes

Next we explored the HIV dataset. UFF selected 179 genes, enabling us to cluster the CD4+ and CD8+ samples into separate clusters with only one misclassification. In comparison, when we clustered the samples using all the genes 2 misclassifications were obtained. In the top ranking genes we find mostly hemoglobin units, but also the specific CD4+ HIV related protein defensin [29] and the CD8+ HIV related CD8 antigen [30]. GO enriched biological processes for the 179 selected genes (Bonferroni < 0.05) include immune system process, immune response, cellular defense response, antigen processing and presentation of peptide antigen via MHC class I and class II. Cellular compartments are enriched for the MHC class I and II protein complexes. Non trivial enriched pathways include Graft-versus-host disease, natural killer cell mediated cytotoxicity and type I diabetes (Bonferroni < 10^-6^). The selected genes involved in the type I diabetes pathway are usually in direct connection with either CD4+ or CD8+ T-cells. This connection is strongly support by literature text mining (not shown). The list of selected genes is provided in Additional file 1, Table S2. Enriched terms are provided in Additional file 2, Table S2.

Similar to figure 5, Additional file 3, Figure S2 displays the performance of clustering the HIV instances using different gene sets, selected by various unsupervised feature selection methods, random selection and using all the genes, as well as comparison to a feature extraction method, selecting the first eigenvectors computed using SVD. The performance of UFF surpasses all other methods in terms of clustering results (see Methods).

### Chronic hepatitis C - UFF selected genes

**Figure 6:**
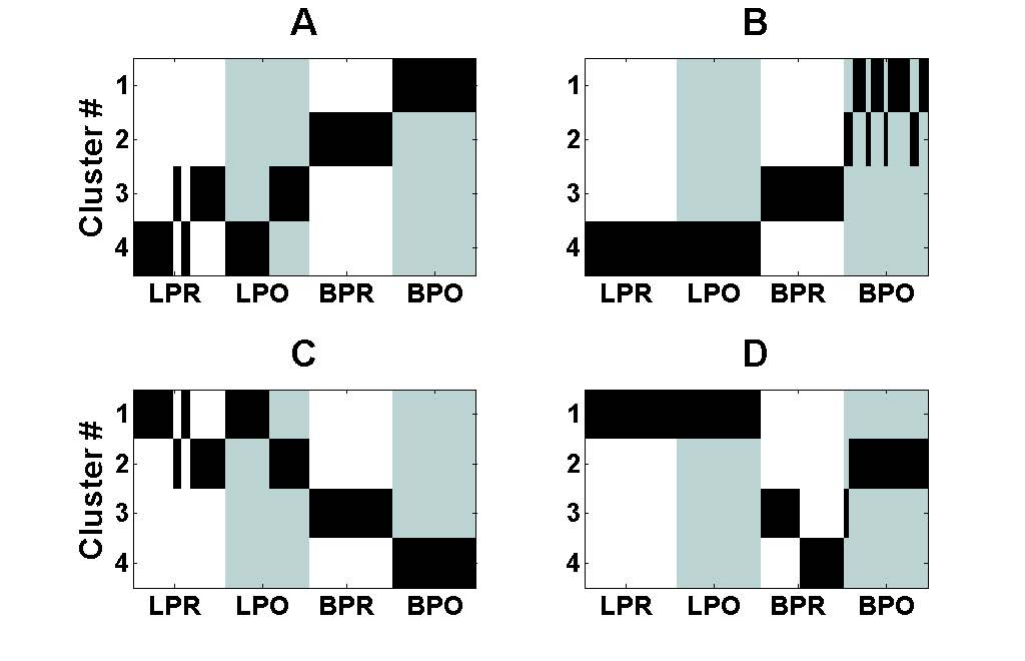
**Clustering of the CHC dataset**. Clustering of the 78 samples of Hepatitis C dataset, relative to known labeling. Y-axis denotes cluster number and X-axis denotes division into pre-interferon liver biopsy (LPR), post-interferon liver biopsy (LPO), pre-interferon blood sample (BPR) and post-interferon blood sample (BPO). Clustering was performed using both *k*-means (k = 4) using UFF selected genes (A) and using all genes (B) and by using Quantum Clustering using UFF selected genes (C) and using all genes (D). Alternating colors are introduced to help view cluster boundaries.

The CHC database is intended for inspecting results of chronic hepatitis C (CHC) treatment with interferon (Figure 6). UFF selected 513 genes. Using these selected genes, we were able to separate perfectly pre-interferon and post interferon blood samples. Liver biopsies, however, were clustered according to sample origin instead of pre and post interferon treatment. The clustering results are different when using all the genes; in this case, liver samples could not be separated at all and blood samples typically split into different clusters. This is displayed in Figure 5. The relevance of the gene selected is demonstrated by the GO enrichment scheme. The GO cellular compartment contains various lipoprotein particles (high-density, plasma, spherical high-density, triglyceride-rich, very-low-density and intermediate-density). Biological process enrichment includes lipid metabolic process, along with regular defense system terms, such as acute inflammatory response, response to wounding and response to xenobiotic stimulus and metabolism of xenobiotics by cytochrome P450 pathway, possibly related to the Interferon treatment [31]. An enriched human phenotype is generalized amyloid deposition, which is reported to relate to hepatitis C [32]. Finally, using the Comparative Toxicogenomics Database (CTD) the UFF selected genes are enriched for Hepatitis and the related immune complex diseases. UFF selected genes and enrichment analysis are provided in Additional file 1, Table S3 and Additional file 2, Table S3 respectively. Clustering results appear in additional file 4, Table S2.

Additional file 3, Figure S3 compares the performance of clustering the Hepatitis-C instances using UFF selected genes with gene sets selected by various unsupervised feature selection methods, random selection and using all the features, as well as comparison to a feature extraction method, selecting the first eigenvectors computed using SVD. The performance of UFF again tops other methods in terms of clustering results.

### Glioblastoma - UFF selected genes

We present results on glioblastoma multiforme (GBM) from The Cancer Genome Atlas (TCGA) project. We selected features from each platform independently, due to the difference between experiments, allowing for identification of genes that differentiate between different platforms, rather than different instance type (UFF was applied to AgilentG4502A_07_1 and AgilentG4502A_07_2 separately, to avoid selection of genes that allows perfect separation of the two platforms). The unsupervised approach displays its full strength in this case, since we do not have access to additional sample information on these datasets.

**Figure 7:**
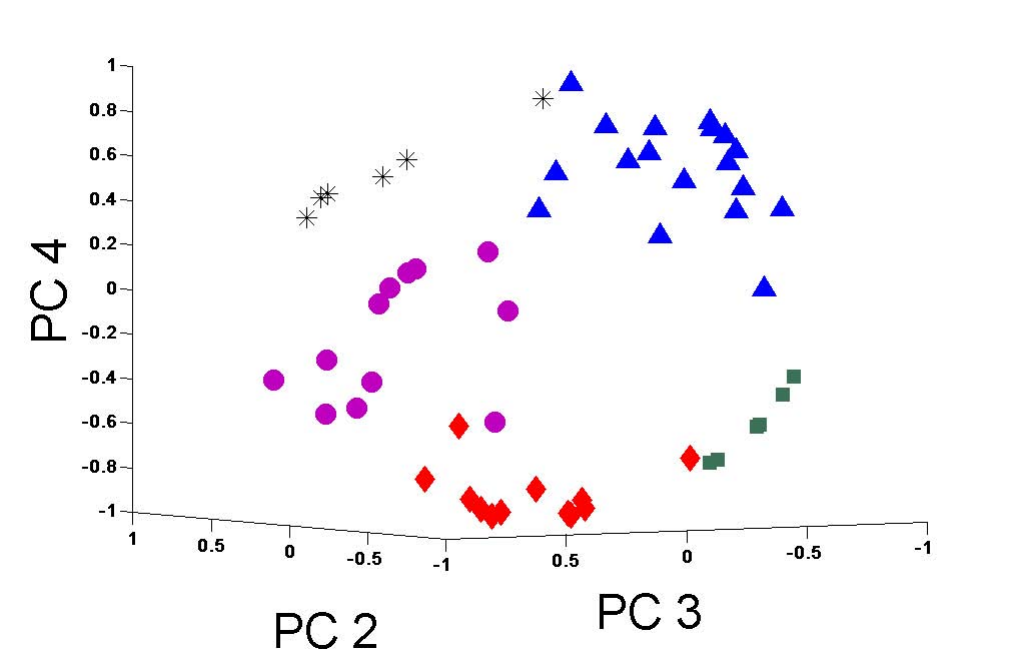
**Clustering of a GBM dataset**. Clustering of 54 samples of GBM Agilent G4502A_07_1.4.2.0 array, colors and shapes denote different clusters. Image displays projection on principal components 2-4.

Based on UFF selected genes, we clearly identify clustering of the instances in each dataset into a small number of groups. As clinical details of the subjects are not specified, we cannot link these clusters to known labels. An example of the clustering results for one of the GBM datasets is displayed in Figure 7. Clustering results of selected datasets are found in Additional file 4, Table S3.

There are variations between the number of genes selected on Agilent and Affymetrix gene expression platforms (563 and 731 genes for Agilent 1 and 2 platforms, while only 140 for Affymetrix).

We focus on the list of 44 genes, which are common to both platforms. 13 genes from this list also appear in the list of top 100 primary glioblastoma-associated genes expressed at higher levels compared with normal brain tissue [33]. We note also that 3 out of 4 patented markers for glioblastoma (patent #7115265) appear on this common list (the 4th marker, ABCC3, appears in genes selected from Agilent 2 platform). The top 10 genes from this list, in terms of minimal UFF rank, are displayed in Table 2 (Detailed list is available in Additional file 5, Table S1). Additional file 6, Table S1 provides detailed explanations on relations to cancer biomarkers. UFF selected genes and the 44 common genes appear in Additional file 1, Table S4.

**Table 2:** Top 10 ranked genes for glioblastoma multiforme

Gene name	Minimal UFF rank across platforms	Related to Cancer Biomarkers
RPS4Y1	1	N.D

SEC61G	1	Yes

POSTN (*)	2	Yes

ECOP	7	Yes

TMSL8 (*)	9	N.D.

SERPINA3 (*)	10	Yes.

COL1A2 (*)	12	Yes

NPTX2	13	Yes

TIMP1 (*)	14	Yes

VSNL1	17	Yes

Genes with asterisk appear on the list of [33]. N.D = Not Determined.

Although Agilent and Affymetrix datasets show high variance in the number of genes selected by UFF, the highest GO enrichment terms are common to both. Both show high GO enrichment of general biological processes such as regulation of multicellular organismal process, cell proliferation and nervous system development (Bonferroni < 0.05) and nervous system development in Affymetrix, (FDR < 0.05, but Bonferroni < 0.1). UFF selected genes on Affymetrix also show inflammatory response while UFF selected genes of Agilent are enriched for cell adhesion. Both platforms are also enriched for cellular compartment of extracellular matrix and both were highly enriched for 'signal peptide' and 'secreted' (Bonferroni < 0.0005) based on UniProt keywords. UFF selected genes on both platforms are enriched for molecular function of protein and receptor binding, which includes various ligands such as polysaccharide, heparin and neuropeptide hormone activity binding (Agilent platform), and lipid and ferric iron binding (Affymetrix platform). Enrichment analysis is provided in Additional file 2, Table S4.

### OV - UFF selected genes

We performed similar analysis of the glioblastoma multiforme (GBM) datasets on the ovarian serous cystadenocarcinoma (OV) dataset from TCGA. UFF selects 669 and 998 genes from Agilent and Affymetrix platform datasets respectively. GO enrichment analysis reveals that UFF selected genes expose very similar GO terms as UFF selected genes on GBM.

The first interesting exception is cellular compartment enrichment in which OV shows enrichment for collagen and fibril, which are identified as predictors for ovarian cancer [34,35]. An enrichment term which includes arthritis and osteoarthritis is of special interest, as the former was postulated as a marker for ovarian cancer [36], while the later has not been determined. Finally, enriched diseases show stomach and breast neoplasms. Clustering of the samples according to the UFF selected genes is provided in Additional file 4, Table S4. Enrichment analysis is provided in Additional file 2, Table S5.

190 genes are common to both Agilent and Affymetrix platforms. Table 3 lists the top 10 common genes in terms of minimal UFF rank. Additional file 5, Table S2 provides detailed explanations for Table 3. List of UFF OV selected genes and the 190 platform-shared genes are provided in Additional file 1, Table S5.

**Table 3:** Top 10 ranked genes for ovarian serous (OV) cystadenocarcinoma

Gene name	Minimal UFF rank across platforms	Related to Cancer Biomarkers
IGF2	1	Yes

HOXA4	2	Yes

POSTN	3	Yes

LMO3	5	Yes

ZIC1	7	Yes

HOXA9	8	Yes

PCP4	8	N.D

OVGP1	9	Yes

PON3	9	N.D

CXCL1	10	Yes

N.D = Not Determined.

7 of the UFF selected genes are common to both GBM and OV. These are POSTN, NPTX2, GJA1, NNMT, CSRP2, SCG5 and HSPA1A, all of them related to cancer biomarkers. Additional file 5, Table S3 provides further details on relation of these 7 common genes to cancer biomarkers. Note that POSTN appears in the top 10 selected genes in both GBM and OV datasets.

### Selected miRNA for GBM and OV

We also report UFF selected microRNAs (miRNA) from TCGA microarrays for the glioblastoma (GBM) and ovarian (OV) cancers. There are 534 miRNAs in GBM, taken from 325 samples and 799 miRNAs taken from 295 OV samples. UFF selected 43 and 63 miRNAs in GBM and OV respectively.

Almost all of the UFF selected miRNAs are human miRNAs (hypergeometric p-value = 0.003 and 0.05 for GBM and OV respectively). The selected miRNAs for GBM and OV are enriched in comparison to [37] list of up or down-regulated miRNAs relative to normal tissue (15 and 20 genes, corresponding to p-values of 7*10^-5 ^and 9*10^-6 ^for GBM and OV respectively). In comparison, negative entropy miRNAs are not enriched relative to this list.

12 of the selected miRNAs appear in both GBM and OV tumors. They are listed in Table 4. Additional file 6, Table S1 provides further details on relation of these miRNAs to cancer biomarkers. Selected miRNAs for GBM and OV are also listed in Additional file 6, Tables S2 and S3.

**Table 4:** MicroRNAs selected by UFF, common to GBM and OV

microRNA	Minimal UFF rank	Related to Cancer Biomarkers
hsa-mir-181a^1^	3	Yes

hsa-mir-363	4	N.D

hsa-mir-210^2^	6	Yes

hsa-mir-451	7	Yes

hsa-mir-10a	7	Yes

hsa-mir-31^1^	8	Yes

hsa-mir-196a^1^	8	Yes

hsa-mir-145*^2,3^	10	Yes

hsa-mir-135b^1^	11	Yes

hsa-mir-10b^1,2,4^	11	Yes

hsa-mir-10b*^1,2,4^	11	Yes

hsa-mir-31*^1^	12	Yes

hsa-mir-424^4^	18	Yes

hsa-mir-155^1,4^	20	Yes

hsa-mir-222^1,2^	25	Yes

hsa-mir-30a*^1,4^	26	Yes

hsa-mir-517*	31	N.D

MicroRNAs selected by UFF, common to GBM and OV.

^1 ^up or down-regulated microRNAs relative to normal tissue according to [37]

^2 ^MicroRNAs that affect the properties of cancer cells according to [37]

^3 ^down-regulated in ovarian cancer [37]

^4 ^Differentially expressed miRNAs in ovarian cancer tissues and cell lines [52].

N.D = Not Determined.

## Conclusions

We present an improved method, and a new web tool, that enable users to benefit from the power of UFF, an unsupervised approach that scores and ranks each feature according to its influence on the singular values distribution.

A statistical characterization of the selected features shows that our method selects features of high variance (over instances), but only those that do not have large correlation only with the first principal component. It turns out that thus we ignore noisy features that have Gaussian distributions. The strength of our method lies in selecting features that both capture inherent clustering of the instances and possess high variance. The combination of the two is significant in the case of biological datasets such as expression microarrays.

By studying various empirical datasets and evaluating different scoring functions we show that our approach is generic, and can identify the subset of relevant features. In contradistinction to other methods we can estimate the size of the group of selected relevant features. Furthermore, we present a novel approximation method, enabling significantly faster calculation of the UFF feature scores.

UFF is a heuristic method which exposes its strength in realistic applications. Nevertheless, not all datasets are amenable to feature selection by UFF. We propose criteria for deciding when UFF application is effective. This information is also provided in the online UFF tool. We further extend the capabilities of UFF by introducing the Unsupervised Detection of Outliers (UDO) method. UDO provides a novel definition of an "outlier-degree" of an instance and identifies such outliers in the dataset. This enables the researcher to detect rare events in the dataset or filter faulty instances before proceeding with further analysis.

Finally, we analyze various gene expression and microRNA expression datasets to show the strength of our approach and to expose interesting findings on these datasets with possible biological relevance.

Web tool: http://adios.tau.ac.il/UFFizi

## Methods

### Datasets

We use three gene-expression microarray datasets with known labeling in order to demonstrate the performance of UFF. They were compiled from the online public repository of the National Center for Biotechnology Information/GenBank Gene Expression Omnibus (GEO) database [38,39]. Data collections are: (i) Gene expression measurements taken from skin tissues including 7 normal skin tissues, 18 benign melanocytic lesions and 45 malignant melanoma [28] (series entry GSE3189); (ii) HIV dataset (series entry GSE6740), containing gene expression measurements from 20 CD4+ and 20 CD8+ T cells from HIV patients at different clinical stages; (iii) Hepatitis C (series entry GSE11190) containing gene expression measurements from 78 samples, comprising of 38 blood samples and 40 liver biopsy, before and after interferon treatment of Hepatitis C (19 blood samples before and after the treatment, 21 and 19 liver biopsies before and after respectively). All these datasets are Affymetrix Human Genome U133A Array (Hepatitis C is a U133 plus 2.0 array).

In addition, we present results obtained from using UFF on The Cancer Genome Atlas (TCGA) gene-expression and microRNA (miRNA) expression datasets[40]. These datasets are comprised of samples taken from (i) glioblastoma multiforme (GBM) and (ii) ovarian serous cystadenocarcinoma (OV) patients. Gene-expression datasets are measured using Affymetrix Human Genome U133A Arrays and Agilent G4502A_07 platforms. miRNA expression is measured using Agilent Human miRNA Microarray Rel12.0 and Agilent 8 × 15 K Human miRNA-specific platforms. Details of these datasets are specified in Additional file 7, Table S1.

### Unsupervised Feature Filtering (UFF)

UFF is based on an entropy measure applied to Singular Value Decomposition (SVD). Let A denote a matrix, whose elements A*ij *denote the measurement of feature *i *on instance *j*, e.g. expression of gene *i *under condition *j*. SVD decomposes the original matrix A into A = USV^T^, where U and V are unitary matrices whose columns form orthonormal bases. The diagonal matrix S is composed of singular values (*s*_*k*_) ordered from highest to lowest. SVD is a common technique in feature extraction. UFF uses the information contained in the singular values in order to select the features.

Let q be the rank of the matrix (q = min(*n,m*), where *n *is the number of instances and *m *is the number of features). Using the singular values, *s*_*k*_, one may define the normalized relative squared values *ρ*_*k *_[41,42]:(3)

A dataset that is characterized by only a few high normalized singular values, whereas the rest are significantly smaller, reflects large redundancy in the data. On the other hand, non-redundant datasets lead to uniformity in the singular values spectrum. UFF exploits this property of the spectrum in order to measure how each feature *i *influences this redundancy, while favoring features which decrease redundancy. The score of a feature *i *is defined using a leave-one-out principle. A function ƒ is calculated on the set of all singular values for the original matrix and for the corresponding set of the matrix without feature *i*. The difference in the values of & defines the score of each feature i. In this work, we use the SVD-entropy (*H*) as the function *f *[42,43] (note that this 'Shannon'-like function does not use probabilities). The score of a feature can be thus regarded as its contribution to the SVD-entropy.(4)

Other functions may be used instead of *H*. They have to be monotonic and vary from a maximum, when all singular values are equal, to a minimum when there is only one singular value bigger than zero. Two such functions that we tested are the negative value of sum of squares and the geometric mean. The results using these functions are very similar to those obtained using the SVD-entropy, hence we will not elaborate further on them.

Figure 2 displays the typical results after applying the UFF algorithm to the melanoma dataset (see the datasets subsection for description), and sorting the features according to the decreasing score of the UFF. Clearly, one can divide the features into three groups:

1. Features with positive score. These features increase the entropy.

2. Neutral features. These features have negligible influence on the entropy.

3. Negative score features. These features decrease the entropy.

We follow the Simple Ranking (SR) method of UFF, denoting positive score features (group 1) as features whose scores lie above the mean score + one std (upper dotted line in figure 2), negative score features (group 3) as features whose scores lie below the mean score - one std (lower dotted line) and neutral features (group 2) the rest. Note that most features fall into group 2, while groups 1 and 3 represent minorities. UFF [6] selects group 1 as containing the most relevant features. The rationale behind this selection is that, because these features increase the entropy, they decrease redundancy. Hence one may expect that instances will be better separated in the space spanned by these features. Further analysis of this group and its comparison with the two other groups is presented in the "properties of selected features" section.

In this paper, we follow the Simple Ranking (SR) method of UFF, selecting all positive score features (group 1). Alternative UFF methods suggested in [6] are not shown.

### GO and Pathway Enrichment

Enrichment of Gene Ontology (GO), KEGG pathways and PubMed papers presented here were calculated using the DAVID [44,45] and ToppGene tools [46]. Verifications were also done using other tools such as Ontologizer [47] and GO Tree Machine [48].

### UFF performance validation

Clustering comparison between different unsupervised feature selection methods was performed using the widely used *k*-means clustering algorithm. In order to provide an unbiased comparison, all feature selection methods were tested with the same input parameter *k *(*k *= 3 for the melanoma dataset, *k *= 2 for the HIV dataset and *k *= 4 for the Hepatitis-C dataset) for the *k*-means clustering algorithm with no additional preprocessing. The clustering was repeated 100 times for each feature selection method and each number of selected features.

Random selection was used to generate 100 different sets. Feature entropy was performed on each feature individually, using the same formalism as in equation 3. We used the Jaccard score [26] to measure the quality of the clustering relative to known labels.

## Abbreviations

List of abbreviations used in this paper: UFF: Unsupervised Feature Filtering; SVD: Singular Value Decomposition; UIS: Unsupervised Instance Selection; CTD: Comparative Toxicogenomics Database.

## Authors' contributions

AG performed the statistical analysis of the UFF, suggested the fast UFF approximation and the criterion of UFF applicability and performed the analysis of the datasets. RV performed the statistical analysis and suggested the UDO algorithm. ML helped to draft the manuscript. DH formulated the proof in the Appendix and helped to draft the manuscript. All authors read and approved the final manuscript.

## Appendix

### Connection between projection on first principal component and negative entropy score

One can prove that in the extreme case, where a feature is lying only on the first PC, it is bound to have a negative score. We shall now prove it for the SVD-entropy function. This proof can be extended to cover also the alternative measures mentioned in the methods section (UFF sub-section).

Starting with the positive-definite Gram matrix *C*, defined as(5)

for the data matrix A of M features by N instances (where, without loss of generality we assume N ≤ M). We use the eigenvalues of the Gram matrix, defined by c_i_≡s_i_^2 ^(see equation 3) to define:(6)

T is positive. SVD entropy can be related to K through(7)

where, for simplicity, we dropped the normalization constant (log(N)) in the definition of H. Consider the small perturbation of removing one feature from the matrix A. The assumption of a small perturbation generally holds for a large enough number of features. Using equation (8), we can write the resulting change of H as(8)

If the removed feature projects only on the first PC, it can change only the first singular value. It follows then that(9)

Plugging the terms in (10) into equation (9), we arrive at(10)

This means that removing such a feature always leads to increase of entropy.

To complete the proof we show that the right hand side is indeed positive. T is positive, and so is the sum of the two terms in brackets, since c_1 _is the leading eigenvalue and the following inequality holds:(11)

We now prove that dc_1 _< 0. Note that, by definition,(12)

The first order perturbation of the eigenvalues of C is related to the change of the original matrix C by the original unitary transformation V. This follows from the unitarity constraint on V(13)

and is the discrete analog of the Hellman-Feynman theorem [49-51].

Removing a row to A, i.e. removing the feature vector f^K ^of size N, the Gram matrix C changes to(14)

Plugging it back into equation (13), we conclude the proof with showing that dc_1 _is negative according to:(15)

where V*i *is the *i*-th eigenvector of C.

Adjusting appropriately S and K, it is easy to prove this also for the sum of squares and the geometric mean functions mentioned in the methods section.

### When is UFF applicable?

We present two measures that allow for a separation between datasets on which UFF is effective, from those in which it is not. The first is SE, an entropy-like measure on normalized squares of UFF score-values.(16)(17)

and the second is VE, an entropy-like measure on the variance-values (i.e. variance of feature-values on all instances)(18)(19)

Suitable datasets can then be defined as those lying below certain thresholds in both measures. We tested more than a dozen 'suitable' and ten 'not-suitable' datasets (not shown) using UFF and clustering algorithms. It seems that combining the two measures using the geometric mean provides the best test for applicability. We found 'suitable' datasets to lie below a threshold of 0.8 of the combined score.

## Supplementary Material

Additional file 1 **UFF selected genes for various datasets**. Gene_list_tables.pdf: UFF selected genes for the viral infection disease and cancer datasets. Click here for file

Additional file 2 **Enrichment of UFF selected genes for various datasets**. Enrichment_tables.pdf: Enrichment of UFF selected genes for the viral infection disease and cancer datasets (using DAVID and ToppGene tools). ToppGene results that appear in DAVID tool were removed. All ToppGene enrichments have Bonferroni < 0.05. Click here for file

Additional file 3 **Additional figures**. Supp_figures.pdf: Comparison of UFF with other selection methods in terms of clustering results on the Melanoma, HIV and Hepatitis-C datasets. Click here for file

Additional file 4 **Clustering of various dataset instances using UFF selected genes**. Clustering_results_tables.pdf: Clustering of the melanoma, Hepatitis-C, GBM and OV dataset instances using UFF selected genes. Click here for file

Additional file 5 **Top ranked genes, selected on all platforms of TCGA datasets**. TCGA_top_ranked_genes.pdf: Top ranked genes, selected on all platforms of TCGA datasets. Click here for file

Additional file 6 **microRNAs, selected by UFF on the TCGA datasets**. miRNA_tables.pdf: lists of microRNAs, selected by UFF on the TCGA datasets. Click here for file

Additional file 7 **List of datasets used in this paper**. Datasets.pdf: List of datasets used in this paper. GEO, Gene Expression Omnibus; TCGA, The Cancer Genome Atlas; GBM, glioblastoma multiforme; OV, ovarian serous cystadenocarcinoma. Click here for file
