# Supplementary material for: UFFizi: a generic platform for ranking informative features
Source: BMC Bioinformatics. 2010 Jun 3;11:300. doi: 10.1186/1471-2105-11-300 (PMC2893168; doi:10.1186/1471-2105-11-300)
Supplement: Additional file 1 — UFF selected genes for various datasets. Gene_list_tables.pdf: UFF selected genes for the viral infection disease and cancer datasets. [file 1471-2105-11-300-S1.PDF]

Table S1: UFF selected genes for the Melanoma dataset.

Melanoma associated genes appear in Talantov et al. *Clin Cancer Res* 2005, **11**(20)
(+ for the up-regulated genes, ++ for the up-regulated and >10 fold genes).

UFF rank	Affymetrix ID	Gene name	Gene annotation	Melanoma associated genes
1	33322_i_at	SFN	stratifin	-
2	209351_at	KRT14	keratin 14	-
3	33323_r_at	SFN	stratifin	-
4	205900_at	KRT1	keratin 1	-
5	207720_at	LOR	loricrin	-
6	201820_at	KRT5	keratin 4	-
7	215121_x_at	IGL@	Immunoglobulin lambda joining 3	-
8	209138_x_at	IGLC2	Immunoglobulin lambda joining 4	-
9	207023_x_at	KRT10	keratin 10	-
10	209848_s_at	SILV	silver homolog (mouse)	+
11	211430_s_at	IGHM	immunoglobulin heavy locus	-
12	202917_s_at	S100A8	s100 calcium binding protein a8 (calgranulin a)	-
13	211745_x_at	HBA2	hemoglobin, alpha 1	-
14	204018_x_at	HBA2	hemoglobin, alpha 1	-
15	210633_x_at	KRT10	keratin 10	-
16	205338_s_at	DCT	dopachrome tautomerase (dopachrome delta-isomerase, tyrosine-related protein 2)	-
17	202403_s_at	COL1A2	collagen, type i, alpha 2	-
18	206400_at	LGALS7	lectin, galactoside-binding, soluble, 7 (galectin 7)	-
19	221651_x_at	IGKV1-5	immunoglobulin kappa variable 1-5	-
20	205916_at	S100A7	s100 calcium binding protein a7 (psoriasin 1)	-
21	218002_s_at	CXCL14	chemokine (c-x-c motif) ligand 14	-
22	213287_s_at	KRT10	keratin 10	-
23	212185_x_at	MT2A	metallothionein 2a	-
24	209458_x_at	HBA2	hemoglobin, alpha 1	-
25	214677_x_at	IGLV3-25	immunoglobulin lambda variable 3-25	-
26	202310_s_at	COL1A1	collagen, type i, alpha 1	-
27	213680_at	KRT6B	keratin 6b	-
28	221671_x_at	IGKV1-5	immunoglobulin kappa variable 1-5	-
29	207908_at	KRT2	keratin 2a (epidermal ichthyosis bullosa of siemens)	-
30	208306_x_at	HLA-DRB5	major histocompatibility complex, class ii, dr beta 1	-
31	201105_at	LGALS1	lectin, galactoside-binding, soluble, 1 (galectin 1)	+
32	214317_x_at	RPS9	ribosomal protein s9	-
33	215379_x_at	IGLJ3	immunoglobulin lambda variable 3-25	-
34	200839_s_at	CTSB	cathepsin b	+
35	201954_at	ARPC1B	actin related protein 2/3 complex, subunit 1b, 41kda	+
36	211696_x_at	HBB	hemoglobin, beta	-
37	217232_x_at	HBB	hemoglobin, beta	-
38	204268_at	S100A2	s100 calcium binding protein a2	-

39	215076_s_at	COL3A1	collagen, type iii, alpha 1 (ehlers-danlos syndrome type iv, autosomal dominant)	-
40	211911_x_at	HLA-C	major histocompatibility complex, class i, b	-
41	208581_x_at	MT1X	metallothionein 1x	-
42	AFFX-HUMGAPDH/M33197_M_at	GAPDH	glyceraldehyde-3-phosphate dehydrogenase	-
43	AFFX-HUMGAPDH/M33197_5_at	GAPDH	glyceraldehyde-3-phosphate dehydrogenase	-
44	217022_s_at	IGHA2	hypothetical protein mgc27165	-
45	217414_x_at	HBA2	hemoglobin, alpha 1	-
46	216231_s_at	B2M	beta-2-microglobulin	-
47	206378_at	SCGB2A2	secretoglobin, family 2a, member 2	-
48	209140_x_at	HLA-C	major histocompatibility complex, class i, b	-
49	AFFX-HUMRGE/M10098_3_at	SRP68	signal recognition particle 68kda	-
50	209312_x_at	HLA-DRB5	major histocompatibility complex, class ii, dr beta 1	-
51	201251_at	PKM2	pyruvate kinase, muscle	+
52	217728_at	S100A6	s100 calcium binding protein a6 (calcyclin)	-
53	AFFX-r2-Hs18SrRNA-3_s_at	NA	na	-
54	209116_x_at	HBB	hemoglobin, beta	-
55	201852_x_at	COL3A1	collagen, type iii, alpha 1 (ehlers-danlos syndrome type iv, autosomal dominant)	-
56	206630_at	TYR	tyrosinase (oculocutaneous albinism ia)	+
57	213274_s_at	CTSB	cathepsin b	++
58	201525_at	APOD	apolipoprotein d	-
59	209125_at	KRT6C	keratin 6e	-
60	211699_x_at	HBA2	hemoglobin, alpha 1	-
61	211456_x_at	RPL35	ribosomal protein l35	-
62	39248_at	AQP3	aquaporin 3 (gill blood group)	-
63	206427_s_at	MLANA	melan-a	-
64	201041_s_at	DUSP1	dual specificity phosphatase 1	-
65	204971_at	CSTA	cystatin a (stefin a)	-
66	202404_s_at	COL1A2	collagen, type i, alpha 2	-
67	214414_x_at	HBA2	hemoglobin, alpha 1	-
68	205064_at	SPRR1B	small proline-rich protein 1b (cornifin)	-
69	214459_x_at	HLA-C	major histocompatibility complex, class i, b	-
70	203936_s_at	MMP9	matrix metallopeptidase 9 (gelatinase b, 92kda gelatinase, 92kda type iv collagenase)	-
71	203980_at	FABP4	fatty acid binding protein 4, adipocyte	-
72	212236_x_at	KRT17	keratin 17	-
73	215193_x_at	HLA-DRB5	major histocompatibility complex, class ii, dr beta 1	-
74	213275_x_at	CTSB	cathepsin b	+
75	209126_x_at	KRT6B	keratin 6b	-
76	208729_x_at	HLA-C	major histocompatibility complex, class i, b	-
77	211858_x_at	GNAS	gnas complex locus	-

78	209619_at	CD74	cd74 antigen (invariant polypeptide of major histocompatibility complex, class ii antigen-associated)	-
79	200838_at	CTSB	cathepsin b	+
80	214327_x_at	TPT1	tumor protein, translationally-controlled 1	-
81	211666_x_at	RPL3	ribosomal protein l3	-
82	216512_s_at	DCT	dopachrome tautomerase (dopachrome delta-isomerase, tyrosine-related protein 2)	-
83	AFFX-HSAC07/X00351_5_at	ACTB	actin, beta	-
84	201893_x_at	DCN	decorin	-
85	AFFX-HSAC07/X00351_M_at	ACTB	actin, beta	-
86	213187_x_at	FTLL1	ferritin, light polypeptide	-
87	206509_at	PIP	prolactin-induced protein	-
88	206595_at	CST6	cystatin e/m	-
89	200026_at	LOC342994	ribosomal protein l34	-
90	203535_at	S100A9	s100 calcium binding protein a9 (calgranulin b)	-
91	211959_at	IGFBP5	insulin-like growth factor binding protein 5	-
92	205694_at	TYRP1	tyrosinase-related protein 1	-
93	210495_x_at	FN1	fibronectin 1	-
94	204326_x_at	MT1X	metallothionein 1x	-
95	204670_x_at	HLA-DRB5	major histocompatibility complex, class ii, dr beta 1	-
96	216442_x_at	FN1	fibronectin 1	-
97	200869_at	LOC390354	ribosomal protein l18a	-
98	202859_x_at	IL8	interleukin 8	-
99	210338_s_at	HSPA8	heat shock 70kda protein 8	-
100	211813_x_at	DCN	decorin	-
101	216526_x_at	HLA-C	major histocompatibility complex, class i, b	-
102	211528_x_at	HLA-G	hla-g histocompatibility antigen, class i, g	-
103	206799_at	SCGB1D2	secretoglobin, family 1d, member 2	-
104	211529_x_at	HLA-G	hla-g histocompatibility antigen, class i, g	-
105	202504_at	TRIM29	tripartite motif-containing 29	-
106	217871_s_at	MIF	macrophage migration inhibitory factor (glycosylation-inhibiting factor)	+
107	217398_x_at	GAPDH	glyceraldehyde-3-phosphate dehydrogenase	-
108	213796_at	SPRR1A	small proline-rich protein 1a	-
109	201348_at	GPX3	glutathione peroxidase 3 (plasma)	-
110	210982_s_at	HLA-DRA	major histocompatibility complex, class ii, dr alpha	-
111	206461_x_at	MT1H	metallothionein 1h	-
112	205382_s_at	CFD	complement factor d (adipsin)	-
113	202295_s_at	CTSH	cathepsin h	+
114	209466_x_at	PTN	pleiotrophin (heparin binding growth factor 8, neurite growth-promoting factor 1)	-
115	201069_at	MMP2	matrix metallopeptidase 2 (gelatinase a, 72kda gelatinase, 72kda type iv collagenase)	-
116	209800_at	KRT16	keratin 16 (focal non-epidermolytic palmoplantar keratoderma)	-

117	212190_at	SERPINE2	serpin peptidase inhibitor, clade e (nexin, plasminogen activator inhibitor type 1), member 2	-
118	208894_at	HLA-DRA	major histocompatibility complex, class ii, dr alpha	-
119	211719_x_at	FN1	fibronectin 1	-
120	212203_x_at	IFITM3	interferon induced transmembrane protein 3 (1-8u)	-
121	202450_s_at	CTSK	cathepsin k (pycnodysostosis)	-
122	217377_x_at	NTRK3	neurotrophic tyrosine kinase, receptor, type 3	++
123	201470_at	GSTO1	glutathione s-transferase omega 1	+
124	201540_at	FHL1	four and a half lim domains 1	-
125	215115_x_at	NTRK3	neurotrophic tyrosine kinase, receptor, type 3	++
126	205157_s_at	KRT17	keratin 17	-
127	201850_at	CAPG	capping protein (actin filament), gelsolin-like	++
128	211969_at	HSP90AA1	heat shock protein 90kda alpha (cytosolic), class a member 1	-
129	202961_s_at	ATP5J2	atp synthase, h+ transporting, mitochondrial f0 complex, subunit f2	+
130	200606_at	DSP	desmoplakin	-
131	200966_x_at	ALDOA	aldolase a, fructose-bisphosphate	+
132	202345_s_at	FABP5	fatty acid binding protein 5 (psoriasis-associated)	-
133	208168_s_at	CHIT1	chitinase 1 (chitotriosidase)	-
134	203381_s_at	APOE	apolipoprotein e	-
135	216207_x_at	IGKV1D-13	na	-
136	200017_at	RPS27A	ribosomal protein s27a	-
137	204734_at	KRT15	keratin 15	-
138	213960_at	NA	na	++
139	209156_s_at	COL6A2	collagen, type vi, alpha 2	-
140	AFFX-CreX-5_at	CRE	cyclization recombinase	-
141	215311_at	NA	na	++
142	63825_at	ABHD2	abhydrolase domain containing 2	+
143	201666_at	TIMP1	timp metallopeptidase inhibitor 1	-
144	212859_x_at	MT1E	metallothionein 1e (functional)	-
145	214669_x_at	IGKC	immunoglobulin kappa constant	-
146	219410_at	TMEM45A	transmembrane protein 45a	-
147	201162_at	IGFBP7	insulin-like growth factor binding protein 7	-
148	221875_x_at	HLA-F	major histocompatibility complex, class i, f	-
149	212464_s_at	FN1	fibronectin 1	-
150	214394_x_at	EEF1D	eukaryotic translation elongation factor 1 delta (guanine nucleotide exchange protein)	-
151	213453_x_at	GAPDH	glyceraldehyde-3-phosphate dehydrogenase	-
152	209122_at	ADFP	adipose differentiation-related protein	-
153	217033_x_at	NTRK3	neurotrophic tyrosine kinase, receptor, type 3	+
154	211968_s_at	HSP90AA1	heat shock protein 90kda alpha (cytosolic), class a member 1	-
155	200799_at	HSPA1B	heat shock 70kda protein 1a	-
156	214580_x_at	KRT6A	keratin 6a	-
157	211990_at	HLA-DPA1	major histocompatibility complex, class ii, dp alpha 1	-
158	209875_s_at	SPP1	secreted phosphoprotein 1 (osteopontin, bone sialoprotein i, early t-lymphocyte activation 1)	++

159	202286_s_at	TACSTD2	tumor-associated calcium signal transducer 2	-
160	213428_s_at	COL6A1	collagen, type vi, alpha 1	-
161	200696_s_at	GSN	gelsolin (amyloidosis, finnish type)	-
162	200866_s_at	PSAP	prosaposin (variant gaucher disease and variant metachromatic leukodystrophy)	-
163	200905_x_at	HLA-E	major histocompatibility complex, class i, e	-
164	211600_at	PTPRO	protein tyrosine phosphatase, receptor type, o	-
165	214836_x_at	NA	na	-
166	209283_at	CRYAB	crystallin, alpha b	+
167	201360_at	CST3	cystatin c (amyloid angiopathy and cerebral hemorrhage)	-
168	202237_at	NNMT	nicotinamide n-methyltransferase	-
169	202411_at	IFI27	interferon, alpha-inducible protein 27	-
170	215176_x_at	NA	na	-
171	200974_at	ACTG2	actin, alpha 2, smooth muscle, aorta	-
172	204584_at	L1CAM	l1 cell adhesion molecule	++
173	201667_at	GJA1	gap junction protein, alpha 1, 43kda (connexin 43)	-
174	208812_x_at	HLA-C	major histocompatibility complex, class i, b	-
175	206462_s_at	NTRK3	neurotrophic tyrosine kinase, receptor, type 3	+
176	217466_x_at	LOC148430	ribosomal protein s2	-
177	201010_s_at	TXNIP	thioredoxin interacting protein	-
178	216438_s_at	TMSL3	thymosin, beta 4, x-linked	-
179	214687_x_at	ALDOA	aldolase a, fructose-bisphosphate	+
180	AFFX-r2-Hs18SrRNA-5_at	NA	na	-
181	210514_x_at	HLA-G	hla-g histocompatibility antigen, class i, g	-
182	218678_at	NES	nestin	++
183	201891_s_at	B2M	beta-2-microglobulin	-
184	214022_s_at	IFITM1	interferon induced transmembrane protein 1 (9-27)	-
185	211986_at	AHNAK	ahnak nucleoprotein (desmoyokin)	-
186	200748_s_at	FTH1	ferritin, heavy polypeptide 1	-
187	217430_x_at	COL1A1	collagen, type i, alpha 1	-
188	217378_x_at	NA	na	-
189	209374_s_at	IGHM	immunoglobulin heavy locus	-
190	208539_x_at	SPRR2E	small proline-rich protein 2b	-
191	AFFX-HUMRGE/M10098_5_at	#N/A	#N/A	-
192	210095_s_at	IGFBP3	insulin-like growth factor binding protein 3	-
193	208687_x_at	HSPA8	heat shock 70kda protein 8	-
194	203074_at	ANXA8L2	annexin a8	-
195	200665_s_at	SPARC	secreted protein, acidic, cysteine-rich (osteonectin)	-
196	211737_x_at	PTN	pleiotrophin (heparin binding growth factor 8, neurite growth-promoting factor 1)	-
197	211597_s_at	HOPX	homeodomain-only protein	-
198	205337_at	DCT	dopachrome tautomerase (dopachrome delta-isomerase, tyrosine-related protein 2)	-
199	209189_at	FOS	v-fos fbj murine osteosarcoma viral oncogene homolog	-
200	201422_at	IFI30	interferon, gamma-inducible protein 30	-

201	208308_s_at	GPI	glucose phosphate isomerase	+
202	213587_s_at	ATP6V0E2	atpase, h+ transporting v0 subunit e2-like (rat)	++
203	200823_x_at	RPL29	ribosomal protein l29	-
204	200795_at	SPARCL1	sparc-like 1 (mast9, hevin)	-
205	215704_at	FLG	filaggrin	-
206	204379_s_at	FGFR3	fibroblast growth factor receptor 3 (achondroplasia, thanatophoric dwarfism)	-
207	211896_s_at	DCN	decorin	-
208	211748_x_at	PTGDS	prostaglandin d2 synthase 21kda (brain)	-
209	201721_s_at	LAPTM5	lysosomal associated multispanning membrane protein 5	-
210	200601_at	ACTN4	actinin, alpha 4	+
211	201601_x_at	IFITM1	interferon induced transmembrane protein 1 (9-27)	-
212	209079_x_at	PCDHGA6	protocadherin gamma subfamily a, 6	+
213	201909_at	RPS4Y1	ribosomal protein s4, y-linked 1	-
214	206560_s_at	MIA	melanoma inhibitory activity	-
215	214091_s_at	GPX3	glutathione peroxidase 3 (plasma)	-
216	211799_x_at	HLA-C	major histocompatibility complex, class i, b	-
217	203186_s_at	S100A4	s100 calcium binding protein a4 (calcium protein, calvasculin, metastasin, murine placental homolog)	-
218	205185_at	SPINK5	serine peptidase inhibitor, kazal type 5	-
219	204806_x_at	HLA-F	major histocompatibility complex, class i, f	-
220	200660_at	S100A11	s100 calcium binding protein a11 (calgizzarin)	+
221	217757_at	A2M	alpha-2-macroglobulin	-
222	208627_s_at	YBX1	y box binding protein 1	-
223	200983_x_at	CD59	cd59 antigen, complement regulatory protein	-
224	208626_s_at	VAT1	vesicle amine transport protein 1 homolog (t californica)	-
225	204475_at	MMP1	matrix metallopeptidase 1 (interstitial collagenase)	-
226	211996_s_at	LOC440354	slc7a5 pseudogene	-
227	213507_s_at	KPNB1	karyopherin (importin) beta 1	-
228	211921_x_at	PTMA	prothymosin, alpha (gene sequence 28)	-
229	221854_at	PKP1	plakophilin 1 (ectodermal dysplasia/skin fragility syndrome)	-
230	201012_at	ANXA1	annexin a1	-
231	207670_at	KRT85	keratin, hair, basic, 5	-

Table S2: UFF selected genes for the HIV dataset.

UFF rank	Affymetrix ID	Gene name	Gene annotation
1	211745_x_at	HBA2	hemoglobin, alpha 1
2	209458_x_at	HBA2	hemoglobin, alpha 1
3	204018_x_at	HBA2	hemoglobin, alpha 1
4	211699_x_at	HBA2	hemoglobin, alpha 1
5	209116_x_at	HBB	hemoglobin, beta
6	205033_s_at	DEFA3	defensin, alpha 1
7	217414_x_at	HBA2	hemoglobin, alpha 1
8	205758_at	CD8A	cd8 antigen, alpha polypeptide (p32)
9	204655_at	CCL5	chemokine (c-c motif) ligand 5
10	217232_x_at	HBB	hemoglobin, beta

11	211696_x_at	HBB	hemoglobin, beta
12	213915_at	NKG7	natural killer cell group 7 sequence
13	217753_s_at	RPS26L	ribosomal protein s26
14	AFFX-r2-Hs18SrRNA-3_s_at	NA	,m10098.1 human 18s rrna sequence, length 1969 bases, 3 prime target bases 1293-1938
15	AFFX-HUMRGE/M10098_3_at	SRP68	signal recognition particle 68kda
16	210321_at	GZMH	granzyme h (cathepsin g-like 2, protein h-ccpx)
17	AFFX-r2-Hs18SrRNA-5_at	NA	,m10098.1 human 18s rrna sequence, length 1969 bases, 5 prime target bases 1-646
18	AFFX-HUMRGE/M10098_5_at	NA	,m10098 human 18s rrna gene, complete (_5, _m, _3 represent transcript regions 5 prime, middle, and 3 prime respectively),h. Sapiens /gen=18s rrna /db_xref=gb:m10098.1 /note=sif corresponding to nucleotides 115-595 of gb:m10098.1 /def=human 18s rrna gene, complete.,homo sapiens /ref=m10098 /def=human 18s rrna gene, complete /len=1969 (_5
19	214146_s_at	PPBP	pro-platelet basic protein (chemokine (c-x-c motif) ligand 7)
20	214317_x_at	RPS9	ribosomal protein s9
21	214414_x_at	HBA2	hemoglobin, alpha 1
22	214327_x_at	TPT1	tumor protein, translationally-controlled 1
23	1405_i_at	CCL5	chemokine (c-c motif) ligand 5
24	201891_s_at	B2M	beta-2-microglobulin
25	210514_x_at	HLA-G	hla-g histocompatibility antigen, class I, g
26	214617_at	PRF1	perforin 1 (pore forming protein)
27	207339_s_at	LTB	lymphotoxin beta (tnf superfamily, member 3)
28	214334_x_at	DAZAP2	daz associated protein 2
29	211074_at	FOLR1	folate receptor 1 (adult)
30	208646_at	RPS14	ribosomal protein s14
31	205821_at	KLRK1	killer cell lectin-like receptor subfamily c, member 4
32	206390_x_at	PF4	platelet factor 4 (chemokine (c-x-c motif) ligand 4)
33	207979_s_at	CD8B	cd8 antigen, beta polypeptide 1 (p37)
34	211530_x_at	HLA-G	hla-g histocompatibility antigen, class I, g
35	208306_x_at	HLA-DRB5	major histocompatibility complex, class ii, dr beta 1
36	205495_s_at	GNLY	granulysin
37	205488_at	GZMA	granzyme a (granzyme 1, cytotoxic t-lymphocyte-associated serine esterase 3)
38	205798_at	IL7R	interleukin 7 receptor
39	201137_s_at	HLA-DPB1	major histocompatibility complex, class ii, dp beta 1
40	213932_x_at	HLA-A	major histocompatibility complex, class I, a
41	210140_at	CST7	cystatin f (leukocystatin)
42	214677_x_at	IGLV3-25	immunoglobulin lambda constant 1 (mcg

			marker)
43	209312_x_at	HLA-DRB5	major histocompatibility complex, class ii, dr beta 1
44	206666_at	GZMK	granzyme k (granzyme 3; tryptase ii)
45	201473_at	JUNB	jun b proto-oncogene
46	37145_at	GNLY	granulysin
47	217466_x_at	LOC148430	similar to 40s ribosomal protein s2
48	200859_x_at	FLNA	filamin a, alpha (actin binding protein 280)
49	216920_s_at	TRGV9	t cell receptor gamma constant 2
50	201008_s_at	TXNIP	thioredoxin interacting protein
51	211990_at	HLA-DPA1	major histocompatibility complex, class ii, dp alpha 1
52	221671_x_at	IGKV1-5	immunoglobulin kappa variable 1-5
53	221651_x_at	IGKV1-5	immunoglobulin kappa variable 1-5
54	202917_s_at	S100A8	s100 calcium binding protein a8 (calgranulin a)
55	201009_s_at	TXNIP	thioredoxin interacting protein
56	215806_x_at	TRGC2	t cell receptor gamma constant 2
57	211799_x_at	HLA-C	major histocompatibility complex, class I, b
58	216248_s_at	NR4A2	nuclear receptor subfamily 4, group a, member 2
59	205483_s_at	ISG15	interferon, alpha-inducible protein (clone ifi-15k)
60	221419_s_at	NA	,gb:nm_013307.1 /def=homo sapiens non-functional folate binding protein (hsaf000381), mrna. /Fea=cds /gen=hsaf000381 /prod=non-functional folate binding protein /db_xref=gi:7019412 /fl=gb:nm_013307.1,gb:nm_013307.1 /db_xref=gi:7019412 /gen=hsaf000381 /fea=flmrna /cnt=1 /tid=hsaffx.900006.1069 /tier=fl /stk=0 /def=homo sapiens non-functional folate binding protein (hsaf000381), mrna. /Prod=non-functional folate binding protein /fl=gb:nm_013307.1
61	201041_s_at	DUSP1	dual specificity phosphatase 1
62	209813_x_at	TARP	t cell receptor gamma constant 2
63	211628_x_at	FTHP1	ferritin, heavy polypeptide pseudogene 1
64	217983_s_at	RNASET2	ribonuclease t2
65	AFFX-r2-Hs28SrRNA-3_at	NA	,m11167.1 human 28s rrna sequence, length 5025 bases, middle target bases 1666-3330
66	208727_s_at	CDC42	cell division cycle 42 (gtp binding protein, 25kda)
67	209138_x_at	IGLC2	immunoglobulin lambda constant 1 (mcg marker)
68	216384_x_at	NA	similar to prothymosin alpha
69	AFFX-r2-P1-cre-3_at	NA	cyclization recombinase
70	200748_s_at	FTH1	ferritin, heavy polypeptide 1
71	201502_s_at	NFKBIA	nuclear factor of kappa light polypeptide gene enhancer in b-cells inhibitor, alpha

72	204794_at	DUSP2	dual specificity phosphatase 2
73	217022_s_at	IGHA2	immunoglobulin heavy constant alpha 2 (a2m marker)
74	209619_at	CD74	cd74 antigen (invariant polypeptide of major histocompatibility complex, class ii antigen-associated)
75	205419_at	EBI2	epstein-barr virus induced gene 2 (lymphocyte-specific g protein-coupled receptor)
76	215193_x_at	HLA-DRB5	major histocompatibility complex, class ii, dr beta 1
77	214290_s_at	HIST2H2AA3	histone 2, h2aa
78	214450_at	CTSW	cathepsin w (lymphopain)
79	209189_at	FOS	v-fos fbj murine osteosarcoma viral oncogene homolog
80	AFFX-CreX-3_at	NA	cyclization recombinase
81	201531_at	ZFP36	zinc finger protein 36, c3h type, homolog (mouse)
82	205898_at	CX3CR1	chemokine (c-x3-c motif) receptor 1
83	201464_x_at	JUN	v-jun sarcoma virus 17 oncogene homolog (avian)
84	210164_at	GZMB	granzyme b (granzyme 2, cytotoxic t-lymphocyte-associated serine esterase 1)
85	204670_x_at	HLA-DRB5	major histocompatibility complex, class ii, dr beta 1
86	AFFX-CreX-5_at	NA	cyclization recombinase
87	203113_s_at	EEF1D	eukaryotic translation elongation factor 1 delta (guanine nucleotide exchange protein)
88	204198_s_at	RUNX3	runt-related transcription factor 3
89	212203_x_at	IFITM3	interferon induced transmembrane protein 3 (1-8u)
90	219228_at	ZNF331	zinc finger protein 331
91	AFFX-HUMRGE/M10098_M_at	GPR34	g protein-coupled receptor 34
92	204622_x_at	NR4A2	nuclear receptor subfamily 4, group a, member 2
93	211445_x_at	NACAP1	nascent-polypeptide-associated complex alpha polypeptide pseudogene 1
94	208113_x_at	PABPC3	poly(a) binding protein, cytoplasmic 3
95	218723_s_at	C13ORF15	response gene to complement 32
96	201859_at	SRGN	proteoglycan 1, secretory granule
97	221558_s_at	LEF1	lymphoid enhancer-binding factor 1
98	214022_s_at	IFITM1	interferon induced transmembrane protein 1 (9-27)
99	211144_x_at	TRGC2	t cell receptor gamma constant 2
100	212240_s_at	PIK3R1	phosphoinositide-3-kinase, regulatory subunit 1 (p85 alpha)
101	200704_at	LITAF	lipopolysaccharide-induced tnf factor
102	202499_s_at	SLC2A3	solute carrier family 2 (facilitated glucose transporter), member 3
103	216834_at	RGS1	regulator of g-protein signalling 1
104	200887_s_at	STAT1	signal transducer and activator of transcription 1, 91kda
105	204197_s_at	RUNX3	runt-related transcription factor 3

106	AFFX-HUMGAPDH/M33197_5_at	GAPDH	glyceraldehyde-3-phosphate dehydrogenase
107	202497_x_at	SLC2A3	solute carrier family 2 (facilitated glucose transporter), member 3
108	209185_s_at	IRS2	insulin receptor substrate 2
109	206559_x_at	EEF1A1	eukaryotic translation elongation factor 1 alpha 1
110	215121_x_at	IGL@	immunoglobulin lambda constant 1 (mcg marker)
111	208679_s_at	ARPC2	actin related protein 2/3 complex, subunit 2, 34kda
112	200943_at	HMGN1	high-mobility group nucleosome binding domain 1
113	213619_at	HNRPH1	heterogeneous nuclear ribonucleoprotein h1 (h)
114	215071_s_at	HIST1H2AC	histone 1, h2ac
115	202086_at	MX1	myxovirus (influenza virus) resistance 1, interferon-inducible protein p78 (mouse)
116	201315_x_at	IFITM2	interferon induced transmembrane protein 2 (1-8d)
117	212242_at	TUBA4A	tubulin, alpha 1 (testis specific)
118	201762_s_at	PSME2	proteasome (prosome, macropain) activator subunit 2 (pa28 beta)
119	209066_x_at	UQCRB	ubiquinol-cytochrome c reductase binding protein
120	209034_at	PNRC1	proline-rich nuclear receptor coactivator 1
121	204439_at	IFI44L	interferon-induced protein 44-like
122	202803_s_at	ITGB2	integrin, beta 2 (complement component 3 receptor 3 and 4 subunit)
123	212185_x_at	MT2A	metallothionein 2a
124	200634_at	PFN1	profilin 1
125	201858_s_at	SRGN	proteoglycan 1, secretory granule
126	217436_x_at	HLA-H	hla-g histocompatibility antigen, class I, g
127	217456_x_at	HLA-E	major histocompatibility complex, class I, e
128	AFFX-M27830_5_at	SOX18	sry (sex determining region y)-box 18
129	204777_s_at	MAL	mal, t-cell differentiation protein
130	204070_at	RARRES3	retinoic acid receptor responder (tazarotene induced) 3
131	217731_s_at	ITM2B	integral membrane protein 2b
132	214470_at	KLRB1	killer cell lectin-like receptor subfamily b, member 1
133	218084_x_at	FXYD5	fxyd domain containing ion transport regulator 5
134	212070_at	GPR56	g protein-coupled receptor 56
135	212588_at	PTPRC	protein tyrosine phosphatase, receptor type, c
136	221269_s_at	SH3BGRL3	sh3 domain binding glutamic acid-rich protein like 3
137	203413_at	NELL2	nel-like 2 (chicken)
138	208549_x_at	LOC441454	similar to prothymosin alpha
139	221756_at	PIK3IP1	hgfl gene

140	AFFX-r2-Hs18SrRNA-M_x_at	NA	,m10098.1 human 18s rrna sequence, length 1969 bases, middle target bases 647-1292
141	39248_at	AQP3	aquaporin 3 (gill blood group)
142	201601_x_at	IFITM1	interferon induced transmembrane protein 1 (9-27)
143	200080_s_at	H3F3B	h3 histone, family 3a pseudogene
144	204103_at	CCL4L2	chemokine (c-c motif) ligand 4
145	200630_x_at	SET	set translocation (myeloid leukemia-associated)
146	204081_at	NRGN	neurogranin (protein kinase c substrate, rc3)
147	211945_s_at	ITGB1	integrin, beta 1 (fibronectin receptor, beta polypeptide, antigen cd29 includes mdf2, msk12)
148	AFFX-M27830_M_at	NA	loc440118
149	208894_at	HLA-DRA	major histocompatibility complex, class ii, dr alpha
150	201123_s_at	EIF5A	eukaryotic translation initiation factor 5a
151	202887_s_at	DDIT4	dna-damage-inducible transcript 4
152	210183_x_at	PNN	pinin, desmosome associated protein
153	202411_at	IFI27	interferon, alpha-inducible protein 27
154	207001_x_at	TSC22D3	tsc22 domain family, member 3
155	212826_s_at	SLC25A6	solute carrier family 25 (mitochondrial carrier; adenine nucleotide translocator), member 6
156	204621_s_at	NR4A2	nuclear receptor subfamily 4, group a, member 2
157	210606_x_at	KLRD1	killer cell lectin-like receptor subfamily d, member 1
158	200664_s_at	DNAJB1	dnaj (hsp40) homolog, subfamily b, member 1
159	201090_x_at	TUBA1C	tubulin, alpha, ubiquitous
160	201029_s_at	CD99	cd99 antigen
161	217963_s_at	NGFRAP1	nerve growth factor receptor (tnfrsf16) associated protein 1
162	207840_at	CD160	cd160 antigen
163	217984_at	RNASET2	ribonuclease t2
164	208961_s_at	KLF6	kruppel-like factor 6
165	217379_at	RPL10	ribosomal protein l10
166	209118_s_at	TUBA1A	tubulin, alpha 3
167	200736_s_at	GPX1	glutathione peroxidase 1
168	201369_s_at	ZFP36L2	zinc finger protein 36, c3h type-like 2
169	212560_at	C11ORF32	chromosome 11 open reading frame 32
170	221474_at	MRLC2	myosin regulatory light chain mrcl3
171	204805_s_at	H1FX	h1 histone family, member x
172	211967_at	TMEM123	transmembrane protein 123
173	213646_x_at	TUBA1C	tubulin, alpha, ubiquitous
174	204279_at	PSMB9	proteasome (prosome, macropain) subunit, beta type, 9 (large multifunctional peptidase 2)
175	213798_s_at	CAP1	cap, adenylate cyclase-associated protein 1 (yeast)
176	202028_s_at	RPL38	ribosomal protein l38

177	204419_x_at	HBG2	hemoglobin, gamma a
178	214453_s_at	IFI44	interferon-induced protein 44
179	201341_at	ENC1	ectodermal-neural cortex (with btb-like domain)

Table S3: UFF selected genes for the Hepatitis-C dataset.

UFF rank	Affymetrix ID	Gene name	Gene annotation
1	1555745_a_at	LYZ	lysozyme (renal amyloidosis)
2	211506_s_at	IL8	interleukin 8
3	214414_x_at	HBA2	hemoglobin, alpha 1
4	213975_s_at	LILRB1	leukocyte immunoglobulin-like receptor, subfamily b (with tm and itim domains), member 1
5	1565228_s_at	ALB	albumin
6	229450_at	IFIT3	interferon-induced protein with tetratricopeptide repeats 3
7	205483_s_at	ISG15	interferon, alpha-inducible protein (clone ifi-15k)
8	209116_x_at	HBB	hemoglobin, beta
9	203153_at	IFIT1	interferon-induced protein with tetratricopeptide repeats 1
10	211745_x_at	HBA2	hemoglobin, alpha 1
11	202411_at	IFI27	interferon, alpha-inducible protein 27
12	209458_x_at	HBA2	hemoglobin, alpha 1
13	226757_at	IFIT2	interferon-induced protein with tetratricopeptide repeats 2
14	201858_s_at	SRGN	proteoglycan 1, secretory granule
15	220491_at	HAMP	hepcidin antimicrobial peptide
16	208470_s_at	HPR	haptoglobin-related protein
17	203535_at	S100A9	s100 calcium binding protein a9 (calgranulin b)
18	204326_x_at	MT1X	metallothionein 1x
19	224567_x_at	MALAT1	metastasis associated lung adenocarcinoma transcript 1 (non-coding rna)
20	202917_s_at	S100A8	s100 calcium binding protein a8 (calgranulin a)
21	211699_x_at	HBA2	hemoglobin, alpha 1
22	202237_at	NNMT	nicotinamide n-methyltransferase
23	204018_x_at	HBA2	hemoglobin, alpha 1
24	211430_s_at	IGHG3	immunoglobulin heavy constant gamma 3 (g3m marker)
25	200832_s_at	SCD	stearoyl-coa desaturase (delta-9-desaturase)
26	201427_s_at	SEPP1	selenoprotein p, plasma, 1
27	211298_s_at	ALB	albumin
28	214677_x_at	IGL@	immunoglobulin lambda locus
29	1558678_s_at	MALAT1	metastasis associated lung adenocarcinoma transcript 1 (non-coding rna)
30	202688_at	TNFSF10	tumor necrosis factor (ligand) superfamily, member 10
31	204533_at	CXCL10	chemokine (c-x-c motif) ligand 10
32	205033_s_at	DEFA3	defensin, alpha 1
33	206697_s_at	HP	haptoglobin

34	217232_x_at	HBB	hemoglobin, beta
35	217414_x_at	HBA2	hemoglobin, alpha 1
36	202086_at	MX1	myxovirus (influenza virus) resistance 1, interferon-inducible protein p78 (mouse)
37	202388_at	RGS2	regulator of g-protein signalling 2, 24kda
38	1558034_s_at	CP	ceruloplasmin (ferroxidase)
39	207076_s_at	ASS1	argininosuccinate synthetase
40	224646_x_at	H19	h19, imprinted maternally expressed untranslated mrna
41	217028_at	CXCR4	chemokine (c-x-c motif) receptor 4
42	219465_at	APOA2	apolipoprotein a-ii
43	211357_s_at	ALDOB	aldolase b, fructose-bisphosphate
44	205216_s_at	APOH	apolipoprotein h (beta-2-glycoprotein i)
45	217757_at	A2M	alpha-2-macroglobulin
46	242625_at	RSAD2	radical s-adenosyl methionine domain containing 2
47	205999_x_at	CYP3A4	cytochrome p450, subfamily iiia (niphedipine oxidase), polypeptide 3
48	219140_s_at	RBP4	retinol binding protein 4, plasma
49	202238_s_at	NNMT	nicotinamide n-methyltransferase
50	217739_s_at	PBEF1	pre-b-cell colony enhancing factor 1
51	205041_s_at	ORM2	orosomucoid 2
52	205040_at	ORM1	orosomucoid 1
53	205820_s_at	APOC3	apolipoprotein c-iii
54	224559_at	MALAT1	metastasis associated lung adenocarcinoma transcript 1 (non-coding rna)
55	AFFX-M27830_5_at	SOX18	sry (sex determining region y)-box 18
56	219863_at	HERC5	hect domain and rld 5
57	213524_s_at	G0S2	g0/g1switch 2
58	208581_x_at	MT1X	metallothionein 1x
59	208607_s_at	SAA2	serum amyloid a2
60	209138_x_at	IGLC2	immunoglobulin lambda constant 1 (mcg marker)
61	211696_x_at	HBB	hemoglobin, beta
62	210929_s_at	AHSG	alpha-2-hs-glycoprotein
63	201909_at	RPS4Y1	ribosomal protein s4, y-linked 1
64	213797_at	RSAD2	radical s-adenosyl methionine domain containing 2
65	219466_s_at	APOA2	apolipoprotein a-ii
66	203400_s_at	TF	transferrin
67	219612_s_at	FGG	fibrinogen gamma chain
68	203649_s_at	PLA2G2A	phospholipase a2, group iia (platelets, synovial fluid)
69	1555229_a_at	C1S	complement component 1, s subcomponent
70	217973_at	DCXR	dicarbonyl/l-xylulose reductase
71	1558048_x_at	NA	na
72	225155_at	SNHG5	chromosome 6 open reading frame 160
73	204551_s_at	AHSG	alpha-2-hs-glycoprotein
74	209660_at	TTR	transthyretin (prealbumin, amyloidosis type i)
75	208383_s_at	PCK1	phosphoenolpyruvate carboxykinase 1 (soluble)
76	204747_at	IFIT3	interferon-induced protein with tetratricopeptide repeats 3
77	204006_s_at	FCGR3B	fc fragment of igg, low affinity iiib, receptor

			(cd16b)
78	214146_s_at	PPBP	pro-platelet basic protein (chemokine (c-x-c motif) ligand 7)
79	207608_x_at	CYP1A2	cytochrome p450, family 1, subfamily a, polypeptide 2
80	204959_at	MNDA	myeloid cell nuclear differentiation antigen
81	204416_x_at	APOC1	apolipoprotein c-i
82	205108_s_at	APOB	apolipoprotein b (including ag(x) antigen)
83	225239_at	TNCRNA	trophoblast-derived noncoding rna
84	204450_x_at	APOA1	apolipoprotein a-i
85	217238_s_at	ALDOB	aldolase b, fructose-bisphosphate
86	201502_s_at	NFKBIA	nuclear factor of kappa light polypeptide gene enhancer in b-cells inhibitor, alpha
87	214063_s_at	TF	transferrin
88	231678_s_at	ADH4	alcohol dehydrogenase 4 (class ii), pi polypeptide
89	209977_at	PLG	plasminogen
90	205305_at	FGL1	fibrinogen-like 1
91	205650_s_at	FGA	fibrinogen alpha chain
92	205892_s_at	FABP1	fatty acid binding protein 1, liver
93	204745_x_at	MT1M	metallothionein 1g
94	204988_at	FGB	fibrinogen beta chain
95	217073_x_at	APOA1	apolipoprotein a-i
96	214456_x_at	SAA1	serum amyloid a1
97	230333_at	SAT1	spermidine/spermine n1-acetyltransferase
98	212185_x_at	MT2A	metallothionein 2a
99	221651_x_at	IGKV1-5	immunoglobulin kappa variable 1-5
100	211996_s_at	LOC440345	hypothetical gene loc283846
101	226702_at	LOC129607	hypothetical protein loc129607
102	216238_s_at	FGB	fibrinogen beta chain
103	215388_s_at	CFH	complement factor h
104	217502_at	IFIT2	interferon-induced protein with tetratricopeptide repeats 2
105	205477_s_at	AMBP	alpha-1-microglobulin/bikunin precursor
106	204561_x_at	APOC2	apolipoprotein c-ii
107	209975_at	CYP2E1	cytochrome p450, family 2, subfamily e, polypeptide 1
108	1553575_at	ND6	nadh dehydrogenase subunit 6
109	202834_at	AGT	angiotensinogen (serpin peptidase inhibitor, clade a, member 8)
110	209309_at	AZGP1	alpha-2-glycoprotein 1, zinc
111	217014_s_at	AZGP1	alpha-2-glycoprotein 1, zinc
112	213988_s_at	SAT1	spermidine/spermine n1-acetyltransferase
113	211997_x_at	H3F3B	h3 histone, family 3a
114	209976_s_at	CYP2E1	cytochrome p450, family 2, subfamily e, polypeptide 1
115	227404_s_at	EGR1	early growth response 1
116	229819_at	A1BG	alpha-1-b glycoprotein
117	224588_at	XIST	x (inactive)-specific transcript
118	217767_at	C3	complement component 3
119	243296_at	PBEF1	pre-b-cell colony enhancing factor 1
120	207820_at	ADH1C	alcohol dehydrogenase 1a (class i), alpha polypeptide

121	201041_s_at	DUSP1	dual specificity phosphatase 1
122	206054_at	KNG1	kininogen 1
123	205141_at	RNASE4	ribonuclease, rnase a family, 4
124	1565162_s_at	MGST1	microsomal glutathione s-transferase 1
125	200909_s_at	RPLP2	ribosomal protein, large, p2
126	212859_x_at	MT1E	metallothionein 1e (functional)
127	209613_s_at	ADH1C	alcohol dehydrogenase 1a (class i), alpha polypeptide
128	217165_x_at	MT1F	metallothionein 1f (functional)
129	209978_s_at	PLG	plasminogen
130	206390_x_at	PF4	platelet factor 4 (chemokine (c-x-c motif) ligand 4)
131	221731_x_at	VCAN	chondroitin sulfate proteoglycan 2 (versican)
132	205681_at	BCL2A1	bcl2-related protein a1
133	AFFX-HUMRGE/M10098_5_at	#N/A	#N/A
134	205943_at	TDO2	tryptophan 2,3-dioxygenase
135	212657_s_at	IL1RN	interleukin 1 receptor antagonist
136	203924_at	GSTA1	glutathione s-transferase a1
137	206350_at	APCS	amyloid p component, serum
138	1431_at	CYP2E1	cytochrome p450, family 2, subfamily e, polypeptide 1
139	209459_s_at	ABAT	4-aminobutyrate aminotransferase
140	1554491_a_at	SERPINC1	serpin peptidase inhibitor, clade c (antithrombin), member 1
141	208147_s_at	CYP2C8	cytochrome p450, family 2, subfamily c, polypeptide 8
142	210013_at	HPX	hemopexin
143	206262_at	ADH1C	alcohol dehydrogenase 1a (class i), alpha polypeptide
144	216598_s_at	CCL2	chemokine (c-c motif) ligand 2
145	217022_s_at	IGHA2	immunoglobulin heavy constant alpha 2 (a2m marker)
146	204705_x_at	ALDOB	aldolase b, fructose-bisphosphate
147	231577_s_at	GBP1	guanylate binding protein 1, interferon-inducible, 67kda
148	206226_at	HRG	histidine-rich glycoprotein
149	224795_x_at	IGKC	immunoglobulin kappa constant
150	208451_s_at	C4B	complement component 4a (rodgers blood group)
151	209118_s_at	TUBA1A	tubulin, alpha 3
152	214274_s_at	ACAA1	acetyl-coenzyme a acyltransferase 1 (peroxisomal 3-oxoacyl-coenzyme a thiolase)
153	208792_s_at	CLU	clusterin
154	204748_at	PTGS2	prostaglandin-endoperoxide synthase 2 (prostaglandin g/h synthase and cyclooxygenase)
155	211919_s_at	CXCR4	chemokine (c-x-c motif) receptor 4
156	31835_at	HRG	histidine-rich glycoprotein
157	221671_x_at	IGKV1-5	immunoglobulin kappa variable 1-5
158	204965_at	GC	group-specific component (vitamin d binding protein)
159	207874_s_at	CFHR4	complement factor h-related 4
160	37020_at	CRP	c-reactive protein, pentraxin-related
161	206287_s_at	ITIH4	inter-alpha (globulin) inhibitor h4 (plasma kallikrein-sensitive glycoprotein)

162	206323_x_at	OPHN1	oligophrenin 1
163	210327_s_at	AGXT	alanine-glyoxylate aminotransferase (oxalosis i; hyperoxaluria i; glycolicaciduria; serine-pyruvate aminotransferase)
164	1553185_at	RASEF	ras and ef-hand domain containing
165	203381_s_at	APOE	apolipoprotein e
166	213629_x_at	MT1F	metallothionein 1f (functional)
167	39763_at	HPX	hemopexin
168	201008_s_at	TXNIP	thioredoxin interacting protein
169	213915_at	NKG7	natural killer cell group 7 sequence
170	212501_at	CEBPB	ccaat/enhancer binding protein (c/ebp), beta
171	204415_at	IFI6	interferon, alpha-inducible protein (clone ifi-6-16)
172	208596_s_at	UGT1A7	udp glucuronosyltransferase 1 family, polypeptide a7
173	214478_at	SPP2	secreted phosphoprotein 2, 24kda
174	218543_s_at	PARP12	poly (adp-ribose) polymerase family, member 12
175	204534_at	VTN	vitronectin
176	214617_at	PRF1	perforin 1 (pore forming protein)
177	208367_x_at	CYP3A4	cytochrome p450, subfamily iiia (niphedipine oxidase), polypeptide 3
178	212884_x_at	APOE	apolipoprotein e
179	206293_at	SULT2A1	sulfotransferase family, cytosolic, 2a, dehydroepiandrosterone (dhea)-preferring, member 1
180	238701_x_at	FLJ45803	flj45803 protein
181	213553_x_at	APOC1	apolipoprotein c-i
182	209616_s_at	CES1	carboxylesterase 1 (monocyte/macrophage serine esterase 1)
183	208747_s_at	C1S	complement component 1, s subcomponent
184	205119_s_at	FPR1	formyl peptide receptor 1
185	209069_s_at	H3F3B	h3 histone, family 3a
186	204007_at	FCGR3B	fc fragment of igg, low affinity iiib, receptor (cd16b)
187	205863_at	S100A12	s100 calcium binding protein a12 (calgranulin c)
188	202017_at	EPHX1	epoxide hydrolase 1, microsomal (xenobiotic)
189	212224_at	ALDH1A1	aldehyde dehydrogenase 1 family, member a1
190	202270_at	GBP1	guanylate binding protein 1, interferon-inducible, 67kda
191	210592_s_at	SAT1	spermidine/spermine n1-acetyltransferase
192	209728_at	HLA-DRB5	major histocompatibility complex, class ii, dr beta 1
193	208791_at	CLU	clusterin
194	202376_at	SERPINA3	serpin peptidase inhibitor, clade a (alpha-1 antiproteinase, antitrypsin), member 3
195	207609_s_at	CYP1A2	cytochrome p450, family 1, subfamily a, polypeptide 2
196	205939_at	CYP3A7	cytochrome p450, family 3, subfamily a, polypeptide 7
197	204607_at	HMGCS2	3-hydroxy-3-methylglutaryl-coenzyme a synthase 2 (mitochondrial)
198	240165_at	NA	na

199	201739_at	SGK1	serum/glucocorticoid regulated kinase
200	202357_s_at	CFB	complement factor b
201	226675_s_at	MALAT1	metastasis associated lung adenocarcinoma transcript 1 (non-coding rna)
202	206505_at	UGT2B4	udp glucuronosyltransferase 2 family, polypeptide b4
203	211452_x_at	LRRFIP1	leucine rich repeat (in flii) interacting protein 1
204	207244_x_at	CYP2A6	cytochrome p450, family 2, subfamily a, polypeptide 6
205	217933_s_at	LAP3	leucine aminopeptidase 3
206	202859_x_at	IL8	interleukin 8
207	214465_at	ORM2	orosomucoid 2
208	225636_at	STAT2	signal transducer and activator of transcription 2, 113kda
209	232266_x_at	CDC2L5	cell division cycle 2-like 5 (cholinesterase-related cell division controller)
210	209374_s_at	IGHM	immunoglobulin heavy locus
211	209969_s_at	STAT1	signal transducer and activator of transcription 1, 91kda
212	207201_s_at	SLC22A1	solute carrier family 22 (organic cation transporter), member 1
213	205654_at	C4BPA	complement component 4 binding protein, alpha
214	211456_x_at	RPL35	ribosomal protein l35
215	211843_x_at	CYP3A7	cytochrome p450, family 3, subfamily a, polypeptide 7
216	215121_x_at	IGLC2	immunoglobulin lambda constant 1 (mcg marker)
217	204620_s_at	VCAN	chondroitin sulfate proteoglycan 2 (versican)
218	209460_at	ABAT	4-aminobutyrate aminotransferase
219	211719_x_at	FN1	fibronectin 1
220	206024_at	HPD	4-hydroxyphenylpyruvate dioxygenase
221	209612_s_at	ADH1C	alcohol dehydrogenase 1a (class i), alpha polypeptide
222	211600_at	PTPRO	protein tyrosine phosphatase, receptor type, o
223	205237_at	FCN1	ficolin (collagen/fibrinogen domain containing) 1
224	205898_at	CX3CR1	chemokine (c-x3-c motif) receptor 1
225	206292_s_at	SULT2A1	sulfotransferase family, cytosolic, 2a, dehydroepiandrosterone (dhea)-preferring, member 1
226	209201_x_at	CXCR4	chemokine (c-x-c motif) receptor 4
227	206094_x_at	UGT1A6	udp glucuronosyltransferase 1 family, polypeptide a6
228	205719_s_at	PAH	phenylalanine hydroxylase
229	214421_x_at	CYP2C9	cytochrome p450, family 2, subfamily c, polypeptide 9
230	209189_at	FOS	v-fos fbj murine osteosarcoma viral oncogene homolog
231	204698_at	ISG20	interferon stimulated exonuclease gene 20kda
232	213695_at	PON3	paraoxonase 3
233	201162_at	IGFBP7	insulin-like growth factor binding protein 7
234	206119_at	BHMT	betaine-homocysteine methyltransferase
235	202687_s_at	TNFSF10	tumor necrosis factor (ligand) superfamily, member 10

236	231736_x_at	MGST1	microsomal glutathione s-transferase 1
237	204920_at	CPS1	carbamoyl-phosphate synthetase 1, mitochondrial
238	210049_at	SERPINC1	serpin peptidase inhibitor, clade c (antithrombin), member 1
239	203186_s_at	S100A4	s100 calcium binding protein a4 (calcium protein, calvasculin, metastasin, murine placental homolog)
240	214453_s_at	IFI44	interferon-induced protein 44
241	AFFX-BioDn-5_at	BIOD	dethiobiotin synthetase
242	206461_x_at	MT1H	metallothionein 1h
243	211796_s_at	TRBV19	t cell receptor beta variable 19
244	206332_s_at	IFI16	interferon, gamma-inducible protein 16
245	228617_at	XAF1	xiap associated factor-1
246	221269_s_at	SH3BGRL3	sh3 domain binding glutamic acid-rich protein like 3
247	235529_x_at	C20ORF118	chromosome 20 open reading frame 118
248	228531_at	SAMD9	sterile alpha motif domain containing 9
249	215223_s_at	SOD2	superoxide dismutase 2, mitochondrial
250	202446_s_at	PLSCR1	phospholipid scramblase 1
251	223781_x_at	ADH4	alcohol dehydrogenase 4 (class ii), pi polypeptide
252	215726_s_at	CYB5A	cytochrome b5 type a (microsomal)
253	207126_x_at	UGT1A8	udp glucuronosyltransferase 1 family, polypeptide a8
254	214290_s_at	HIST2H2AA3	histone 2, h2aa
255	206177_s_at	ARG1	arginase, liver
256	204224_s_at	GCH1	gtp cyclohydrolase 1 (dopa-responsive dystonia)
257	223298_s_at	NT5C3	5'-nucleotidase, cytosolic iii
258	201720_s_at	LAPTM5	lysosomal associated multispanning membrane protein 5
259	212587_s_at	PTPRC	protein tyrosine phosphatase, receptor type, c
260	215125_s_at	UGT1A1	udp glucuronosyltransferase 1 family, polypeptide a1
261	223502_s_at	TNFSF13B	tumor necrosis factor (ligand) superfamily, member 13b
262	221345_at	FFAR2	free fatty acid receptor 2
263	203382_s_at	APOE	apolipoprotein e
264	204439_at	IFI44L	interferon-induced protein 44-like
265	219371_s_at	KLF2	kruppel-like factor 2 (lung)
266	208763_s_at	TSC22D3	tsc22 domain family, member 3
267	204994_at	MX2	myxovirus (influenza virus) resistance 2 (mouse)
268	205754_at	F2	coagulation factor ii (thrombin)
269	204846_at	CP	ceruloplasmin (ferroxidase)
270	205753_at	CRP	c-reactive protein, pentraxin-related
271	227364_at	CAPZA1	capping protein (actin filament) muscle z-line, alpha 1
272	207409_at	LECT2	leukocyte cell-derived chemotaxin 2
273	204151_x_at	AKR1C1	aldo-keto reductase family 1, member c1 (dihydrodiol dehydrogenase 1; 20-alpha (3-alpha)-hydroxysteroid dehydrogenase)
274	1494_f_at	CYP2A6	cytochrome p450, family 2, subfamily a, polypeptide 6

275	213293_s_at	TRIM22	tripartite motif-containing 22
276	AFFX-HUMRGE/M10098_M_at	GPR34	g protein-coupled receptor 34
277	209122_at	ADFP	adipose differentiation-related protein
278	227265_at	FGL2	fibrinogen-like 2
279	214038_at	CCL8	chemokine (c-c motif) ligand 8
280	216025_x_at	CYP2C9	cytochrome p450, family 2, subfamily c, polypeptide 9
281	235061_at	PPM1K	protein phosphatase 1k (pp2c domain containing)
282	213800_at	CFH	complement factor h
283	208966_x_at	IFI16	interferon, gamma-inducible protein 16
284	216594_x_at	AKR1C1	aldo-keto reductase family 1, member c1 (dihydrodiol dehydrogenase 1; 20-alpha (3-alpha)-hydroxysteroid dehydrogenase)
285	211295_x_at	CYP2A6	cytochrome p450, family 2, subfamily a, polypeptide 6
286	223940_x_at	MALAT1	metastasis associated lung adenocarcinoma transcript 1 (non-coding rna)
287	203455_s_at	SAT1	spermidine/spermine n1-acetyltransferase
288	205255_x_at	TCF7	transcription factor 7 (t-cell specific, hmg-box)
289	216442_x_at	FN1	fibronectin 1
290	206651_s_at	CPB2	carboxypeptidase b2 (plasma, carboxypeptidase u)
291	205755_at	ITIH3	inter-alpha (globulin) inhibitor h3
292	202869_at	OAS1	2',5'-oligoadenylate synthetase 1, 40/46kda
293	204987_at	ITIH2	inter-alpha (globulin) inhibitor h2
294	218943_s_at	DDX58	dead (asp-glu-ala-asp) box polypeptide 58
295	203923_s_at	CYBB	cytochrome b-245, beta polypeptide (chronic granulomatous disease)
296	235094_at	TPM4	tropomyosin 4
297	202768_at	FOSB	fbj murine osteosarcoma viral oncogene homolog b
298	210495_x_at	FN1	fibronectin 1
299	214016_s_at	SFPQ	splicing factor proline/glutamine-rich (polypyrimidine tract binding protein associated)
300	206345_s_at	PON1	paraoxonase 1
301	225344_at	NCOA7	nuclear receptor coactivator 7
302	211653_x_at	AKR1C2	aldo-keto reductase family 1, member c2 (dihydrodiol dehydrogenase 2; bile acid binding protein; 3-alpha hydroxysteroid dehydrogenase, type iii)
303	200986_at	SERPING1	serpin peptidase inhibitor, clade g (c1 inhibitor), member 1, (angioedema, hereditary)
304	220017_x_at	CYP2C9	cytochrome p450, family 2, subfamily c, polypeptide 9
305	208949_s_at	LGALS3	lectin, galactoside-binding, soluble, 3 (galectin 3)
306	202018_s_at	LTF	lactotransferrin
307	228592_at	MS4A1	membrane-spanning 4-domains, subfamily a, member 1
308	202241_at	TRIB1	tribbles homolog 1 (drosophila)
309	205552_s_at	OAS1	2',5'-oligoadenylate synthetase 1, 40/46kda

310	209240_at	OGT	o-linked n-acetylglucosamine (glcnac) transferase (udp-n-acetylglucosamine:polypeptide-n-acetylglucosaminyl transferase)
311	208891_at	DUSP6	dual specificity phosphatase 6
312	203752_s_at	JUND	jun d proto-oncogene
313	204532_x_at	UGT1A3	udp glucuronosyltransferase 1 family, polypeptide a3
314	202464_s_at	PFKFB3	6-phosphofructo-2-kinase/fructose-2,6-biphosphatase 3
315	1405_i_at	CCL5	chemokine (c-c motif) ligand 5
316	216661_x_at	CYP2C9	cytochrome p450, family 2, subfamily c, polypeptide 9
317	202499_s_at	SLC2A3	solute carrier family 2 (facilitated glucose transporter), member 3
318	210797_s_at	OASL	2'-5'-oligoadenylate synthetase-like
319	201425_at	ALDH2	aldehyde dehydrogenase 2 family (mitochondrial)
320	207218_at	F9	coagulation factor ix (plasma thromboplastic component, christmas disease, hemophilia b)
321	212592_at	IGJ	immunoglobulin j polypeptide, linker protein for immunoglobulin alpha and mu polypeptides
322	205649_s_at	FGA	fibrinogen alpha chain
323	201422_at	IFI30	interferon, gamma-inducible protein 30
324	205890_s_at	UBD	ubiquitin d
325	205404_at	HSD11B1	hydroxysteroid (11-beta) dehydrogenase 1
326	202283_at	SERPINF1	serpin peptidase inhibitor, clade f (alpha-2 antiplasmin, pigment epithelium derived factor), member 1
327	33304_at	ISG20	interferon stimulated exonuclease gene 20kda
328	209699_x_at	AKR1C2	aldo-keto reductase family 1, member c2 (dihydrodiol dehydrogenase 2; bile acid binding protein; 3-alpha hydroxysteroid dehydrogenase, type iii)
329	201135_at	ECHS1	enoyl coenzyme a hydratase, short chain, 1, mitochondrial
330	212464_s_at	FN1	fibronectin 1
331	204563_at	SELL	selectin l (lymphocyte adhesion molecule 1)
332	201721_s_at	LAPTM5	lysosomal associated multispanning membrane protein 5
333	201031_s_at	HNRPH1	heterogeneous nuclear ribonucleoprotein h1 (h)
334	227253_at	CP	ceruloplasmin (ferroxidase)
335	220796_x_at	SLC35E1	solute carrier family 35, member e1
336	204081_at	NRGN	neurogranin (protein kinase c substrate, rc3)
337	209146_at	SC4MOL	sterol-c4-methyl oxidase-like
338	209395_at	CHI3L1	chitinase 3-like 1 (cartilage glycoprotein-39)
339	44673_at	SIGLEC1	sialic acid binding ig-like lectin 1, sialoadhesin
340	209774_x_at	CXCL2	chemokine (c-x-c motif) ligand 2
341	205660_at	OASL	2'-5'-oligoadenylate synthetase-like
342	239979_at	EPSTI1	epithelial stromal interaction 1 (breast)
343	AFFX-BioDn-3_at	BIOD	dethiobiotin synthetase
344	207096_at	SAA4	serum amyloid a4, constitutive
345	218400_at	OAS3	2'-5'-oligoadenylate synthetase 3, 100kda

346	1555812_a_at	ARHGDIB	rho gdp dissociation inhibitor (gdi) beta
347	214722_at	NOTCH2NL	notch homolog 2 (drosophila) n-terminal like
348	206754_s_at	CYP2B7P1	cytochrome p450, family 2, subfamily b, polypeptide 7 pseudogene 1
349	205576_at	SERPIND1	serpin peptidase inhibitor, clade d (heparin cofactor), member 1
350	221816_s_at	PHF11	phd finger protein 11
351	202644_s_at	TNFAIP3	tumor necrosis factor, alpha-induced protein 3
352	219803_at	ANGPTL3	angiopoietin-like 3
353	209366_x_at	CYB5A	cytochrome b5 type a (microsomal)
354	213507_s_at	KPNB1	karyopherin (importin) beta 1
355	201669_s_at	MARCKS	myristoylated alanine-rich protein kinase c substrate
356	219434_at	TREM1	triggering receptor expressed on myeloid cells 1
357	211991_s_at	HLA-DPA1	major histocompatibility complex, class ii, dp alpha 1
358	210982_s_at	HLA-DRA	major histocompatibility complex, class ii, dr alpha
359	203790_s_at	HRSP12	heat-responsive protein 12
360	226603_at	SAMD9L	sterile alpha motif domain containing 9-like
361	202269_x_at	GBP1	guanylate binding protein 1, interferon-inducible, 67kda
362	206910_x_at	CFHR2	complement factor h-related 2
363	201315_x_at	IFITM2	interferon induced transmembrane protein 2 (1-8d)
364	229625_at	GBP5	guanylate binding protein 5
365	1557987_at	IMAA	slc7a5 pseudogene
366	219014_at	PLAC8	placenta-specific 8
367	211571_s_at	VCAN	chondroitin sulfate proteoglycan 2 (versican)
368	217761_at	ADI1	acireductone dioxygenase 1
369	213872_at	C6ORF62	chromosome 6 open reading frame 62
370	204998_s_at	ATF5	activating transcription factor 5
371	200800_s_at	HSPA1B	heat shock 70kda protein 1a
372	1555167_s_at	PBEF1	pre-b-cell colony enhancing factor 1
373	204661_at	CD52	cd52 antigen (campath-1 antigen)
374	208471_at	HPR	haptoglobin-related protein
375	1552362_a_at	LEAP2	liver-expressed antimicrobial peptide 2
376	203915_at	CXCL9	chemokine (c-x-c motif) ligand 9
377	1553906_s_at	FGD2	fyve, rhogef and ph domain containing 2
378	221008_s_at	AGXT2L1	alanine-glyoxylate aminotransferase 2-like 1
379	207574_s_at	GADD45B	growth arrest and dna-damage-inducible, beta
380	226218_at	IL7R	interleukin 7 receptor
381	207392_x_at	UGT2B15	udp glucuronosyltransferase 2 family, polypeptide b15
382	224565_at	TNCRNA	trophoblast-derived noncoding rna
383	225415_at	DTX3L	deltex 3-like (drosophila)
384	214320_x_at	CYP2A6	cytochrome p450, family 2, subfamily a, polypeptide 6
385	209614_at	ADH1C	alcohol dehydrogenase 1a (class i), alpha polypeptide
386	227609_at	EPSTI1	epithelial stromal interaction 1 (breast)
387	210825_s_at	PEBP1	phosphatidylethanolamine binding protein 1

388	210873_x_at	APOBEC3A	apolipoprotein b mrna editing enzyme, catalytic polypeptide-like 3a
389	209304_x_at	GADD45B	growth arrest and dna-damage-inducible, beta
390	202902_s_at	CTSS	cathepsin s
391	202307_s_at	TAP1	transporter 1, atp-binding cassette, sub-family b (mdr/tap)
392	224568_x_at	MALAT1	metastasis associated lung adenocarcinoma transcript 1 (non-coding rna)
393	213603_s_at	RAC2	ras-related c3 botulinum toxin substrate 2 (rho family, small gtp binding protein rac2)
394	200799_at	HSPA1B	heat shock 70kda protein 1a
395	205488_at	GZMA	granzyme a (granzyme 1, cytotoxic t-lymphocyte-associated serine esterase 3)
396	211682_x_at	UGT2B28	udp glucuronosyltransferase 2 family, polypeptide b28
397	210397_at	DEFB1	defensin, beta 1
398	230036_at	SAMD9L	sterile alpha motif domain containing 9-like
399	201531_at	ZFP36	zinc finger protein 36, c3h type, homolog (mouse)
400	1553186_x_at	RASEF	ras and ef-hand domain containing
401	230466_s_at	NA	na
402	214211_at	FTH1	ferritin, heavy polypeptide 1
403	1555832_s_at	KLF6	kruppel-like factor 6
404	228498_at	B4GALT1	udp-gal:betaglcnac beta 1,4-galactosyltransferase, polypeptide 1
405	219519_s_at	SIGLEC1	sialic acid binding ig-like lectin 1, sialoadhesin
406	231897_at	LTB4DH	leukotriene b4 12-hydroxydehydrogenase
407	200629_at	WARS	interferon-induced protein 53
408	216336_x_at	MT1M	metallothionein 1g
409	216733_s_at	GATM	glycine amidinotransferase (l-arginine:glycine amidinotransferase)
410	208965_s_at	IFI16	interferon, gamma-inducible protein 16
411	208892_s_at	DUSP6	dual specificity phosphatase 6
412	202803_s_at	ITGB2	integrin, beta 2 (complement component 3 receptor 3 and 4 subunit)
413	229476_s_at	THRSP	thyroid hormone responsive (spot14 homolog, rat)
414	218559_s_at	MAFB	v-maf musculoaponeurotic fibrosarcoma oncogene homolog b (avian)
415	202081_at	IER2	immediate early response 2
416	234989_at	TNCRNA	trophoblast-derived noncoding rna
417	202718_at	IGFBP2	insulin-like growth factor binding protein 2, 36kda
418	227522_at	CMBL	similar to mouse 2310016a09rik gene
419	204122_at	TYROBP	tyro protein tyrosine kinase binding protein
420	221558_s_at	LEF1	lymphoid enhancer-binding factor 1
421	AFFX-r2-Ec-bioD-5_at	BIOD	dethiobiotin synthetase
422	215646_s_at	VCAN	chondroitin sulfate proteoglycan 2 (versican)
423	224569_s_at	IRF2BP2	interferon regulatory factor 2 binding protein 2
424	204158_s_at	TCIRG1	t-cell, immune regulator 1, atpase, h+ transporting, lysosomal v0 subunit a3
425	203178_at	GATM	glycine amidinotransferase (l-arginine:glycine amidinotransferase)

426	230000_at	RNF213	chromosome 17 open reading frame 27
427	202887_s_at	DDIT4	dna-damage-inducible transcript 4
428	208982_at	PECAM1	platelet/endothelial cell adhesion molecule (cd31 antigen)
429	212067_s_at	C1R	complement component 1, r subcomponent
430	205370_x_at	DBT	dihydrolipoamide branched chain transacylase e2
431	206245_s_at	IVNS1ABP	influenza virus ns1a binding protein
432	208894_at	HLA-DRA	major histocompatibility complex, class ii, dr alpha
433	203964_at	NMI	n-myc (and stat) interactor
434	214261_s_at	ADH6	alcohol dehydrogenase 6 (class v)
435	201743_at	CD14	cd14 antigen
436	210845_s_at	PLAUR	plasminogen activator, urokinase receptor
437	201649_at	UBE2L6	ubiquitin-conjugating enzyme e2l 6
438	210972_x_at	TRAC	t cell receptor alpha constant
439	200598_s_at	HSP90B1	heat shock protein 90kda beta (grp94), member 1
440	209305_s_at	GADD45B	growth arrest and dna-damage-inducible, beta
441	213564_x_at	LDHB	lactate dehydrogenase b
442	205081_at	CRIP1	cysteine-rich protein 1 (intestinal)
443	214329_x_at	TNFSF10	tumor necrosis factor (ligand) superfamily, member 10
444	212827_at	IGHM	immunoglobulin heavy locus
445	205500_at	C5	complement component 5
446	1555564_a_at	CFI	complement factor i
447	207339_s_at	LTB	lymphotoxin beta (tnf superfamily, member 3)
448	211990_at	HLA-DPA1	major histocompatibility complex, class ii, dp alpha 1
449	230554_at	ACSM2B	hypothetical protein loc123876
450	213193_x_at	TRBV19	t cell receptor beta variable 19
451	206727_at	C9	complement component 9
452	209185_s_at	IRS2	insulin receptor substrate 2
453	225673_at	MYADM	myeloid-associated differentiation marker
454	201893_x_at	DCN	decorin
455	208962_s_at	FADS1	fatty acid desaturase 1
456	208998_at	UCP2	uncoupling protein 2 (mitochondrial, proton carrier)
457	229026_at	CDC42SE2	cdc42 small effector 2
458	200831_s_at	SCD	stearoyl-coa desaturase (delta-9-desaturase)
459	235276_at	EPSTI1	epithelial stromal interaction 1 (breast)
460	210558_at	AKR1C4	aldo-keto reductase family 1, member c4 (chlordecone reductase; 3-alpha hydroxysteroid dehydrogenase, type i; dihydrodiol dehydrogenase 4)
461	218345_at	TMEM176A	hepatocellular carcinoma-associated antigen 112
462	214022_s_at	IFITM1	interferon induced transmembrane protein 1 (9-27)
463	201275_at	FDPS	farnesyl diphosphate synthase (farnesyl pyrophosphate synthetase, dimethylallyltranstransferase, geranyltranstransferase)
464	210888_s_at	ITIH1	inter-alpha (globulin) inhibitor h1
465	205495_s_at	GNLY	granulysin

466	215176_x_at	NA	na
467	1555759_a_at	CCL5	chemokine (c-c motif) ligand 5
468	207238_s_at	PTPRC	protein tyrosine phosphatase, receptor type, c
469	34210_at	CD52	cd52 antigen (campath-1 antigen)
470	213737_x_at	GOLGA8C	golgi autoantigen, golgin subfamily a, 8c
471	214084_x_at	NA	na
472	227897_at	RAP2B	rap2b, member of ras oncogene family
473	228152_s_at	FLJ31033	hypothetical protein flj31033
474	232617_at	CTSS	cathepsin s
475	AFFX-r2-Ec-bioC-3_at	ECS0855	putative enzyme bioc
476	204834_at	FGL2	fibrinogen-like 2
477	213048_s_at	SET	set translocation (myeloid leukemia-associated)
478	210168_at	C6	complement component 6
479	217564_s_at	CPS1	carbamoyl-phosphate synthetase 1, mitochondrial
480	200887_s_at	STAT1	signal transducer and activator of transcription 1, 91kda
481	36711_at	MAFF	v-maf musculoaponeurotic fibrosarcoma oncogene homolog f (avian)
482	1555756_a_at	CLEC7A	c-type lectin domain family 7, member a
483	37512_at	HSD17B6	hydroxysteroid (17-beta) dehydrogenase 6
484	241914_s_at	ACSM2B	hypothetical protein loc123876
485	203828_s_at	IL32	interleukin 32
486	243509_at	BTG1	b-cell translocation gene 1, anti-proliferative
487	211922_s_at	CAT	catalase
488	205695_at	SDS	serine dehydratase
489	202951_at	STK38	serine/threonine kinase 38
490	214428_x_at	C4B	complement component 4a (rodgers blood group)
491	229723_at	TAGAP	t-cell activation gtpase activating protein
492	1552701_a_at	COP1	caspase-1 dominant-negative inhibitor pseudo-ice
493	209937_at	TM4SF4	transmembrane 4 l six family member 4
494	216187_x_at	KLC1	kinesin 2
495	201694_s_at	EGR1	early growth response 1
496	228155_at	C10ORF58	chromosome 10 open reading frame 58
497	225176_at	NA	na
498	211644_x_at	IGKC	immunoglobulin kappa constant
499	220626_at	SERPINA10	serpin peptidase inhibitor, clade a (alpha-1 antiproteinase, antitrypsin), member 10
500	212671_s_at	HLA-DQA2	major histocompatibility complex, class ii, dq alpha 1
501	1552703_s_at	CASP1	caspase 1, apoptosis-related cysteine peptidase (interleukin 1, beta, convertase)
502	200648_s_at	GLUL	glutamate-ammonia ligase (glutamine synthetase)
503	208918_s_at	NADK	nad kinase
504	200634_at	PFN1	profilin 1
505	215076_s_at	COL3A1	collagen, type iii, alpha 1 (ehlers-danlos syndrome type iv, autosomal dominant)
506	201044_x_at	DUSP1	dual specificity phosphatase 1
507	203416_at	CD53	cd53 antigen
508	235964_x_at	C20ORF118	chromosome 20 open reading frame 118

509	201786_s_at	ADAR	adenosine deaminase, rna-specific
510	204614_at	SERPINB2	serpin peptidase inhibitor, clade b (ovalbumin), member 2
511	220951_s_at	A1CF	apobec-1 complementation factor
512	224918_x_at	MGST1	microsomal glutathione s-transferase 1
513	201348_at	GPX3	glutathione peroxidase 3 (plasma)

Table S4: UFF selected genes for the TCGA glioblastoma multiforme datasets (Agilent and Affymetrix platforms).

Top 100 refers to list of top 100 primary glioblastoma-associated genes,
expressed at higher levels compared with normal brain tissue (Tso et al, Mol Cancer Res 2006, 4:607)
Bolded genes appear in both platforms (Agilent and Affymetrix platforms).

Minimal rank	Gene	Appears in Agilent_1	Appears in Agilent_2	Appears in Affymetrix	Appears in top 100
1	CACNG5	+	-	-	-
1	**RPS4Y1**	+	+	+	-
1	**SEC61G**	+	+	+	+
2	ATXN10	+	-	-	-
2	HBB	-	-	+	-
2	**POSTN**	+	+	+	+
3	CDK4	-	-	+	-
3	RPS4Y2	+	+	-	-
3	TAF3	+	-	-	-
4	HOXA4	+	+	-	-
4	LTF	-	-	+	-
4	UBTF	+	-	-	-
5	CD93	+	-	-	-
5	HBA2	-	-	+	-
5	LUZP2	+	+	-	-
6	BAG3	+	-	-	-
6	CHI3L1	-	-	+	+
7	C6orf159	+	-	-	-
7	CDKN2A	-	+	-	-
7	**ECOP**	-	+	+	-
8	CRYAB	-	-	+	-
8	EIF1AY	+	+	-	-
8	GGA3	+	-	-	-
9	C7orf55	+	-	-	-
9	**TMSL8**	+	+	+	+
10	HOMER3	+	-	-	-
10	SAA1	+	+	-	-
10	**SERPINA3**	-	+	+	+
11	APOD	-	-	+	-
11	KIAA1576	+	+	-	-
11	PAPSS2	+	-	-	-
12	**COL1A2**	-	+	+	+
12	CYP26B1	+	+	-	-
12	SYT13	+	-	-	-
13	HOPX	-	-	+	-

13	**NPTX2**	+	+	+	-
13	VAMP3	+	-	-	-
14	DACT2	+	+	-	-
14	LGALS4	+	-	-	-
14	**TIMP1**	-	+	+	+
15	C3orf41	-	+	-	-
15	CST3	-	-	+	-
15	PPIG	+	-	-	-
16	DAGLB	+	-	-	-
16	EGFR	+	+	-	+
16	PLP1	-	-	+	-
17	BBS4	+	-	-	-
17	CHCHD2	-	-	+	-
17	**VSNL1**	-	+	+	-
18	KRT16	+	-	-	-
18	PTGDS	-	-	+	-
18	RELN	-	+	-	-
19	IL8	+	+	-	+
19	NUP107	-	-	+	-
19	OTX1	+	-	-	-
20	**LGALS3**	-	+	+	-
20	ZNF358	+	+	-	-
21	C3	-	-	+	+
21	PCDHB5	-	+	-	-
21	RCSD1	+	-	-	-
22	C20orf30	+	+	-	-
22	COL3A1	-	-	+	+
22	MGAT4A	+	-	-	-
23	CDH19	-	+	-	-
23	**TGFBI**	-	+	+	+
24	EYA4	-	+	-	-
24	LOC541472	+	-	-	-
24	PMP2	-	-	+	-
25	**AGT**	+	-	+	-
25	LY6H	+	-	-	-
25	NPAT	+	+	-	-
26	CSF3	-	+	-	-
26	hCG_16001	+	-	-	-
27	CCT2	-	-	+	-
27	HOXA9	+	+	-	-
27	**ISG15**	+	-	+	-
28	FLJ39660	+	-	-	-
28	ODZ2	+	+	-	-
28	**SRPX**	-	+	+	+
29	ADAD1	+	-	-	-
29	**GPNMB**	+	+	+	+
29	MOXD1	-	+	-	-
30	SLN	-	-	+	-
30	TMEM177	+	-	-	-
30	ZNF404	+	+	-	-
31	FUT9	+	-	-	-
31	MGP	-	-	+	+

31	SEMA3E	-	+	-	-
32	EHBP1	+	-	-	-
32	FA2H	+	+	-	-
33	AQP1	-	-	+	-
33	NDN	-	+	-	-
33	PRPF40A	+	-	-	-
34	ANXA2	-	-	+	+
34	GAS2	-	+	-	-
34	RPSAP15	+	-	-	-
35	FABP7	-	-	+	+
35	HPR	-	+	-	-
35	UBE2O	+	-	-	-
36	**GJA1**	+	-	+	-
36	PAAF1	+	-	-	-
36	PRDM13	-	+	-	-
37	COL6A3	-	-	+	-
37	GSDMDC1	+	-	-	-
37	RARRES2	+	+	-	-
38	CHN1	-	-	+	-
38	COL1A1	-	+	-	-
38	NRGN	+	-	-	-
39	**NNMT**	-	+	+	-
39	SMCR5	+	-	-	-
39	SP8	-	+	-	-
40	MEOX2	-	+	-	-
40	**S100A10**	-	+	+	-
40	SLC22A18AS	+	-	-	-
41	CXorf38	-	+	-	-
41	FASTKD2	+	-	-	-
41	**MRPS17**	-	+	+	-
42	AKT1	-	-	+	-
42	C7orf41	+	-	-	-
42	NEFL	+	+	-	-
43	MLLT11	-	-	+	-
43	TSPAN15	+	-	-	-
44	**FABP5**	+	+	+	-
44	HS3ST3A1	-	+	-	-
44	SGK2	+	-	-	-
45	BARHL1	+	-	-	-
45	RPS3A	-	+	-	-
46	EPN3	+	-	-	-
46	**LANCL2**	+	+	+	-
46	TMEM125	-	+	-	-
47	ERCC1	+	-	-	-
47	**GPX3**	-	+	+	-
47	PITX2	+	+	-	-
48	CPXM2	-	+	-	-
48	**HLA-DRA**	+	-	+	+
48	STRA6	+	-	-	-
49	FLJ25770	+	-	-	-
49	GJB2	-	+	-	-
49	MT1X	-	-	+	-

50	COL9A3	-	+	-	-
50	PRR3	+	-	-	-
50	S100B	-	-	+	-
51	**FCGBP**	-	+	+	-
51	HSPB3	-	+	-	-
51	RDH5	+	-	-	-
52	KCNS1	+	-	-	-
52	TF	-	-	+	-
52	TPO	+	+	-	-
53	COMMD9	+	-	-	-
53	CXCL14	-	-	+	-
53	ZMAT4	-	+	-	-
54	NPTXR	+	-	-	-
54	**PBEF1**	-	+	+	+
55	CAMP	-	+	-	-
55	LPL	-	-	+	-
55	NR2F6	+	-	-	-
56	MAL2	+	-	-	-
56	SUSD5	+	+	-	-
57	BTF3	+	+	-	-
57	MYL1	+	-	-	-
57	NMB	-	-	+	+
58	CDH18	+	+	-	-
58	**GBAS**	-	+	+	-
59	APOC1	-	-	+	+
59	KCNK1	-	+	-	-
59	MPHOSPH8	+	-	-	-
60	HTATSF1	+	-	-	-
60	**MEST**	-	+	+	-
60	PDLIM4	-	+	-	-
61	CHD3	+	-	-	-
61	FOXC1	-	+	-	-
61	SNAP25	-	-	+	-
62	B3GNT5	+	-	-	-
62	DNMBP	-	+	-	-
62	UCHL1	-	-	+	-
63	HTRA1	-	-	+	-
63	**PFN2**	+	+	+	-
63	STX4	+	-	-	-
64	ANGPTL4	+	+	-	-
64	C6orf54	-	+	-	-
64	RTN1	-	-	+	-
65	C1orf61	-	-	+	-
65	EPB42	+	+	-	-
65	KIAA1199	+	+	-	-
66	ABI3BP	-	+	-	-
66	CAMK2N1	-	-	+	-
66	DDAH1	+	-	-	-
67	DMRT2	-	+	-	-
67	GPR82	+	-	-	-
67	VSIG4	-	-	+	-
68	ARMC3	+	+	-	-

68	BEX1	-	-	+	-
68	STT3A	+	-	-	-
69	A2M	-	-	+	-
69	GABRB1	-	+	-	-
69	ISX	+	-	-	-
70	BASP1	-	-	+	-
70	PDGFRA	-	+	-	-
70	TARBP1	+	-	-	-
71	HOXB6	-	+	-	-
71	HSPB1	-	-	+	-
71	USP36	+	-	-	-
72	C6orf114	+	-	-	-
72	CHRNA9	+	+	-	-
72	**SLC1A3**	-	+	+	-
73	CD74	-	-	+	-
73	PTX3	-	+	-	-
73	TBX1	+	-	-	-
74	CCL2	-	-	+	-
74	CCL28	+	-	-	-
74	RAD51C	-	+	-	-
75	C16orf3	+	-	-	-
75	G0S2	-	+	-	-
75	IGFBP3	-	-	+	+
76	CCDC140	-	+	-	-
76	CLIP2	+	-	-	-
76	ODC1	-	-	+	-
77	HLA-DPA1	-	-	+	-
77	INOC1	+	-	-	-
77	KLRC2	+	+	-	-
78	CKB	-	-	+	-
78	LOC389118	+	-	-	-
78	SMOC1	-	+	-	-
79	IFI6	-	-	+	-
79	ZBTB33	-	+	-	-
80	CYP1B1	-	+	-	-
80	LOC554235	+	-	-	-
81	**CSRP2**	+	-	+	+
81	JAKMIP1	-	+	-	-
81	PLAC1	+	-	-	-
82	CTHRC1	-	+	-	-
82	**PLA2G2A**	-	+	+	-
82	TMEM83	+	-	-	-
83	CST8	+	-	-	-
83	SLC10A4	-	+	-	-
83	TSPAN31	-	-	+	-
84	ABCC3	-	+	-	-
84	NES	-	-	+	+
84	PEX12	+	-	-	-
85	NKIRAS1	-	+	-	-
85	**SCG5**	+	-	+	-
86	FLJ38973	+	-	-	-
86	NGEF	-	+	-	-

86	STMN2	-	-	+	-
87	ACE2	+	-	-	-
87	C1QB	-	-	+	+
87	FGF13	-	+	-	-
88	ART3	-	+	-	-
88	**HIG2**	-	+	+	-
88	RAC3	+	-	-	-
89	CHI3L2	-	-	+	-
89	SLC14A1	-	+	-	-
89	ZC3H6	+	-	-	-
90	C19orf53	+	-	-	-
90	CD163	-	-	+	+
91	PCSK1	-	+	-	-
91	PRTN3	+	-	-	-
91	TIMP4	-	-	+	-
92	FLJ37543	+	-	-	-
92	TMEM158	-	-	+	-
93	FBLN5	-	+	-	-
93	P2RXL1	+	-	-	-
93	SCRG1	-	-	+	-
94	EFEMP1	-	-	+	+
94	SLIT2	-	+	-	-
95	HOXC9	+	+	-	-
95	IMPA2	+	-	-	-
95	**TNC**	-	+	+	+
96	NUP133	-	+	-	-
96	RAP1B	+	-	-	-
96	SCG2	-	-	+	-
97	**CCT6A**	-	+	+	-
97	CXorf57	-	+	-	-
97	IFNA21	+	-	-	-
98	MYLIP	+	-	-	-
98	NEFM	+	+	-	-
98	STXBP1	-	-	+	-
99	EXOSC8	-	+	-	-
99	HLA-DRB1	-	-	+	+
99	VSTM1	+	-	-	-
100	**ALDOC**	+	-	+	-
100	C1GALT1	+	-	-	-
100	PROM1	-	+	-	-
101	**HSPA1A**	-	+	+	-
101	PNOC	-	+	-	-
101	U2AF2	+	-	-	-
102	FAM92A3	+	-	-	-
102	LEMD1	-	+	-	-
103	ATP1B1	-	-	+	-
103	CAV2	+	+	-	-
103	SNX10	-	+	-	-
104	ADM	+	+	-	-
104	FXYD6	-	-	+	-
104	ITGAV	+	-	-	-
105	GPRC6A	+	-	-	-

105	LOX	-	+	-	-
105	MARS	-	-	+	-
106	DCTN2	-	-	+	-
106	GABRA2	-	+	-	-
106	TGFBRAP1	+	+	-	-
107	ATP1A2	-	-	+	-
107	PNLIPRP2	+	-	-	-
107	SOCS2	-	+	-	-
108	OCIAD2	+	+	-	-
108	RGS2	-	-	+	-
109	C19orf48	+	-	-	-
109	FREM1	-	+	-	-
109	SIVA1	-	-	+	-
110	DAPL1	+	+	-	-
110	KLK6	+	+	-	-
110	PON2	-	-	+	-
111	ATP6V1C2	-	+	-	-
111	GDA	+	-	-	-
111	SH3GLB1	-	-	+	-
112	HOXB3	-	+	-	-
112	SERPINE2	-	-	+	-
112	TRIM2	+	-	-	-
113	C20orf54	+	-	-	-
113	CHIC2	-	-	+	-
113	GSTT1	-	+	-	-
114	ACTA2	-	-	+	-
114	SFTPA1	+	-	-	-
114	WDR5	-	+	-	-
115	CAV1	-	-	+	+
115	IGFBP2	-	+	-	+
116	KLHL9	-	+	-	-
116	SEPP1	-	-	+	-
116	TTC18	+	-	-	-
117	ALOX5AP	-	-	+	-
117	LARP5	-	+	-	-
118	FAM107A	-	-	+	-
118	LRRC17	-	+	-	-
118	TUBGCP2	+	-	-	-
119	C13orf15	-	-	+	-
119	KIAA0513	+	-	-	-
119	SVIP	-	+	-	-
120	ANXA1	-	-	+	+
120	CAMK4	+	-	-	-
120	CCL7	-	+	-	-
121	HOXD10	-	+	-	-
121	IFI27	-	-	+	-
121	TNS1	+	-	-	-
122	HLA-B	-	-	+	-
122	OGN	-	+	-	-
122	POL3S	+	-	-	-
123	PFAS	+	-	-	-
123	PTN	-	-	+	-

124	PDPN	-	-	+	+
125	FTH1	-	-	+	-
125	NPTX1	+	+	-	-
126	ARSJ	-	+	-	-
126	DPEP2	+	-	-	-
126	**SLC35E3**	-	+	+	-
127	OS9	-	-	+	-
127	RBM4	+	-	-	+
127	SPON2	+	+	-	-
128	COL23A1	-	+	-	-
128	DCTN1	+	-	-	-
128	DKK1	-	-	+	-
129	CALB1	-	+	-	-
129	LYPLA1	+	-	-	-
129	NPC2	-	-	+	-
130	KIT	-	+	-	-
130	KLF7	+	-	-	-
130	**SRGN**	+	-	+	-
131	ALG14	+	-	-	-
131	**F13A1**	+	-	+	-
131	PCP4	-	+	-	-
132	HEPACAM	+	-	-	-
132	IRS2	-	-	+	-
132	WNT16	-	+	-	-
133	CHST8	-	+	-	-
133	S100A6	-	-	+	-
133	ZNF509	+	-	-	-
134	**CCK**	+	+	+	-
134	OLIG1	+	-	-	-
134	PCOLCE2	+	+	-	-
135	BTNL3	-	+	-	-
135	CD14	-	-	+	+
135	PPM1H	+	-	-	-
136	CENPO	-	+	-	-
136	**RP11-35N6.1**	-	+	+	-
137	MMP3	-	-	+	-
137	SH3GLB2	+	-	-	-
137	ZNF229	+	+	-	-
138	BHMT2	+	+	-	-
138	LGALS1	-	-	+	-
138	RASEF	-	+	-	-
139	GALNT13	-	+	-	-
139	**S100A13**	-	+	+	-
139	SETD5	+	-	-	-
140	DISC1	+	-	-	-
140	IGF2	-	-	+	-
140	RASL11B	-	+	-	-
141	D4S234E	-	+	-	-
141	IL20	+	-	-	-
142	C10orf81	-	+	-	-
142	DPM2	+	-	-	-
143	MXRA5	-	+	-	-

143	TBL1Y	+	-	-	-
144	CA14	-	+	-	-
144	QTRT1	+	-	-	-
145	FGF9	-	+	-	-
145	RGS7	+	-	-	-
146	BIRC4	+	-	-	-
147	EDG2	-	+	-	-
147	KRT75	+	-	-	-
148	KIAA0907	+	-	-	-
149	INSM1	+	-	-	-
149	SAA2	-	+	-	-
150	BCAT1	+	-	-	-
150	ST6GALNAC5	-	+	-	-
151	ASPM	+	-	-	-
151	RGS4	-	+	-	-
152	ALDH1A1	-	+	-	-
152	ZFP42	+	-	-	-
153	NIP30	+	-	-	-
154	FLRT3	-	+	-	-
154	PRKDC	+	-	-	-
155	CNTNAP1	-	+	-	-
155	RASD1	+	-	-	-
156	HOXB2	-	+	-	-
156	OR2L13	+	-	-	-
157	CP	-	+	-	-
158	BTNL2	+	+	-	-
159	STOM	+	-	-	-
159	TRPM8	-	+	-	-
160	CDC14B	+	-	-	-
160	MMP13	-	+	-	-
161	OR6W1P	+	-	-	-
161	ZNRD1	-	+	-	-
162	BDNF	+	+	-	-
163	C8G	+	-	-	-
163	ZNF642	-	+	-	-
164	CNN1	-	+	-	-
165	ADCY6	+	-	-	-
165	VWC2	+	+	-	-
166	OCC-1	+	-	-	-
166	UBD	-	+	-	-
167	OR2J3	+	-	-	-
167	THAP2	-	+	-	-
168	DOK6	-	+	-	-
169	CHRNB2	+	-	-	-
169	ZBTB3	+	+	-	-
170	FRMD5	-	+	-	-
170	ORMDL2	+	-	-	-
171	C4orf31	-	+	-	-
171	TPPP3	+	-	-	-
172	TMLHE	+	-	-	-
173	IL13RA2	-	+	-	-
174	OR51S1	+	-	-	-

174	SIM2	-	+	-	-
175	DPP4	-	+	-	-
175	NXPH3	+	-	-	-
176	LOC51252	+	-	-	-
176	PRAF2	-	+	-	-
177	C10orf116	-	+	-	-
177	TSFM	+	+	-	-
178	TSPYL5	+	+	-	-
178	WFDC2	+	-	-	-
179	CRLF1	+	-	-	-
179	HIST1H4C	-	+	-	-
180	KIAA1217	+	-	-	-
180	PLCXD3	-	+	-	-
181	SLITRK5	-	+	-	-
181	TGDS	+	-	-	-
182	GHITM	+	-	-	-
182	PTGIS	-	+	-	-
183	PACRG	+	-	-	-
183	WDR69	-	+	-	-
184	HOXD8	-	+	-	-
184	SUV39H2	+	-	-	-
185	ARHGDIA	-	+	-	-
185	C8orf37	+	-	-	-
186	ESX1	+	-	-	-
186	PCDHB17	-	+	-	-
187	SCAMP3	+	-	-	-
187	SCUBE2	-	+	-	-
188	FCGR3A	-	+	-	-
188	PHOX2A	+	-	-	-
189	CDK5RAP1	+	-	-	-
189	NEFH	-	+	-	-
190	C9orf125	-	+	-	-
190	TMEFF2	+	+	-	-
191	FAM137A	+	-	-	-
191	HOTAIR	-	+	-	-
192	KIAA1377	-	+	-	-
192	PODN	+	-	-	-
193	QARS	+	-	-	-
193	ZDHHC11	-	+	-	-
194	AP4B1	+	-	-	-
194	KCNJ3	-	+	-	-
195	DLX5	-	+	-	-
195	RYR3	+	-	-	-
196	KIAA1305	+	-	-	-
197	SPATA9	-	+	-	-
198	FAM77C	-	+	-	-
198	SLC5A9	+	-	-	-
199	KIAA1826	+	-	-	-
199	NPNT	-	+	-	-
200	OGDHL	-	+	-	-
200	OPRM1	+	-	-	-
201	ALPK2	-	+	-	-

201	TRIM21	+	-	-	-
202	EYA1	-	+	-	-
202	PDGFD	+	-	-	-
203	C10orf88	+	-	-	-
203	MMP7	+	+	-	-
204	METTL8	+	-	-	-
204	SLC9A9	-	+	-	-
205	CD200	+	-	-	-
205	TMEM132B	-	+	-	-
206	C11orf70	-	+	-	-
206	SFRP2	+	-	-	-
207	SERPINI1	-	+	-	-
207	TUFM	+	-	-	-
208	FAP	+	+	-	-
208	TFCP2L1	-	+	-	-
209	DLK1	+	-	-	-
209	LHX4	+	+	-	-
210	GDF10	-	+	-	-
211	LOC152485	-	+	-	-
211	USPL1	+	-	-	-
212	KIAA0746	-	+	-	-
212	NDUFA5	+	-	-	-
213	DNAJC7	+	-	-	-
213	PSPH	+	+	-	-
214	ZNF214	+	-	-	-
215	C3orf14	-	+	-	-
215	PCSK6	+	-	-	-
216	CCDC55	+	-	-	-
217	RGS20	-	+	-	-
218	COX7A1	+	-	-	-
218	ERP27	-	+	-	-
219	ELL3	+	-	-	-
219	TTC13	-	+	-	-
220	DACH2	-	+	-	-
220	TAC1	+	+	-	-
221	VCPIP1	-	+	-	-
222	RIF1	+	-	-	-
222	TMEM130	-	+	-	-
223	CDH13	-	+	-	-
223	KCNG1	+	-	-	-
224	FAM133A	-	+	-	-
225	MGC16291	-	+	-	-
225	VAT1	+	-	-	-
226	IDS	+	-	-	-
226	RBM11	-	+	-	-
227	CPM	-	+	-	-
228	CA12	-	+	-	-
228	FLJ36208	+	-	-	-
229	PI16	+	+	-	-
230	CLDN10	-	+	-	-
230	SIRPB1	+	-	-	-
231	ZFAND2B	+	-	-	-

232	DKFZP586P0123	+	-	-	-
232	SKIV2L	-	+	-	-
233	JARID1D	-	+	-	-
233	TRIM17	+	-	-	-
234	COLEC12	+	+	-	-
234	LRIG3	-	+	-	-
235	LRRC8D	+	-	-	-
236	FAM139A	+	-	-	-
236	PKD2	-	+	-	-
237	GSPT2	+	-	-	-
237	HOXA7	-	+	-	-
238	ARID4A	+	-	-	-
238	ZNF659	+	+	-	-
239	IL1B	-	+	-	-
239	SYT1	+	+	-	-
240	RPRML	+	-	-	-
240	XRCC1	-	+	-	-
241	PENK	-	+	-	-
241	TUBA3D	+	-	-	-
242	HS3ST5	-	+	-	-
242	NLRC5	+	-	-	-
243	C20orf144	+	-	-	-
243	CYTL1	-	+	-	-
244	CD52	-	+	-	-
244	ISL2	+	-	-	-
245	MGAT2	+	-	-	-
245	PKIB	-	+	-	-
246	FBXO2	-	+	-	-
246	TUBAL3	+	-	-	-
247	CELSR2	-	+	-	-
247	RPL5	+	-	-	-
248	HMOX1	-	+	-	+
248	MAL	+	-	-	-
249	DNASE1	+	-	-	-
250	CUL3	-	+	-	-
250	EAF1	+	-	-	-
251	ABCC1	+	-	-	-
251	CBLN2	-	+	-	-
252	CRHR2	+	-	-	-
253	OSBPL5	+	-	-	-
253	PCDHGB6	-	+	-	-
254	PAX3	-	+	-	-
254	RPL10L	+	-	-	-
255	ACSBG1	+	-	-	-
255	SHOX2	-	+	-	-
256	ODF3L1	+	-	-	-
257	ITGBL1	-	+	-	-
258	POLR2H	-	+	-	-
258	ZNF576	+	-	-	-
259	KIAA0226	-	+	-	-
260	EBF3	-	+	-	-
260	HBE1	+	-	-	-

261	CSTB	+	-	-	-
261	SPOCK1	-	+	-	-
262	ETS2	+	-	-	-
262	RGS22	-	+	-	-
263	CLEC2B	-	+	-	-
263	SMPDL3B	+	-	-	-
264	CAB39L	+	-	-	-
264	CCL13	-	+	-	-
265	DHRS9	-	+	-	-
265	EED	+	-	-	-
266	EGR3	-	+	-	-
266	MS4A2	+	-	-	-
267	RPL39L	-	+	-	-
267	TMCO2	+	-	-	-
268	KLRC3	-	+	-	-
269	IQCA	-	+	-	-
269	SPC25	+	-	-	-
270	ARMC4	+	+	-	-
270	TRH	-	+	-	-
271	CNDP1	+	-	-	-
271	CXCL3	-	+	-	-
272	CCNA1	-	+	-	-
272	COL11A1	+	-	-	-
273	CDH10	+	-	-	-
273	KHDRBS2	+	+	-	-
274	NPR3	-	+	-	-
275	CTNNBIP1	-	+	-	-
275	RPL34	+	-	-	-
276	ACTR6	+	-	-	-
276	H1F0	-	+	-	-
277	CBX2	+	-	-	-
277	IL7	-	+	-	-
278	CREG2	-	+	-	-
278	FAM40A	+	-	-	-
279	NCAM2	-	+	-	-
279	RPE65	+	-	-	-
280	HOXA10	-	+	-	-
280	PCSK9	+	-	-	-
281	C9orf47	-	+	-	-
282	AMZ1	-	+	-	-
282	SYT4	+	-	-	-
283	HIST1H4L	+	+	-	-
283	RPL26	+	-	-	-
284	AURKB	+	+	-	-
284	CFD	-	+	-	-
285	C5orf25	-	+	-	-
285	CYLC1	+	-	-	-
286	C6	+	-	-	-
286	UNC5D	-	+	-	-
287	DCN	-	+	-	-
287	SPTBN1	+	-	-	-
288	TGFA	-	+	-	-

289	FAM5C	+	-	-	-
289	FGFBP2	+	+	-	-
290	NAP1L2	-	+	-	-
290	RCHY1	+	-	-	-
291	OVOS2	-	+	-	-
291	S100G	+	-	-	-
292	ARMC9	+	-	-	-
292	CFI	-	+	-	-
293	BIRC3	-	+	-	-
293	ZNF429	+	-	-	-
294	DGKD	+	-	-	-
294	FAM81B	-	+	-	-
295	CCDC11	+	-	-	-
295	MGST1	-	+	-	-
296	RANBP9	+	-	-	-
296	SDPR	-	+	-	-
297	BMP4	+	-	-	-
297	LCE1B	-	+	-	-
298	HAMP	-	+	-	-
298	RNF26	+	-	-	-
299	SERPINF2	-	+	-	-
299	TREM1	+	-	-	-
300	PVRL3	-	+	-	-
300	ZNF195	+	-	-	-
301	LELP1	+	-	-	-
301	LOC221091	-	+	-	-
302	LEFTY2	-	+	-	-
302	PYGM	+	-	-	-
303	CYB5R2	-	+	-	-
303	MZF1	+	-	-	-
304	TUSC1	-	+	-	-
305	MMP9	-	+	-	+
305	THSD3	+	-	-	-
306	MGC39715	-	+	-	-
306	WDR85	+	-	-	-
307	TIMP2	-	+	-	-
308	MSTN	-	+	-	-
309	CXCL1	-	+	-	-
309	KIAA0258	+	-	-	-
310	FBN1	+	-	-	-
310	USP9Y	-	+	-	-
311	LIMS2	+	-	-	-
311	RGS17	-	+	-	-
312	CNTNAP3	+	+	-	-
312	SULF2	-	+	-	-
313	GAN	-	+	-	-
314	C7orf28B	+	-	-	-
314	LY75	-	+	-	-
315	PPP1R14C	-	+	-	-
315	TMED6	+	-	-	-
316	CXorf1	+	-	-	-
316	GLI1	-	+	-	-

317	AGER	+	-	-	-
317	COL13A1	-	+	-	-
318	LTK	+	-	-	-
318	SOX4	-	+	-	+
319	OR51M1	+	-	-	-
320	H2AFX	+	-	-	-
321	SGCE	-	+	-	-
322	DCLRE1B	+	-	-	-
322	RNF165	-	+	-	-
323	BXDC5	+	-	-	-
324	WFDC10B	+	-	-	-
325	INA	-	+	-	-
325	RPL3L	+	-	-	-
326	CD48	-	+	-	-
327	NPY	-	+	-	-
328	MYST4	-	+	-	-
328	NXT2	+	-	-	-
329	CLK2	+	-	-	-
329	FAM26F	-	+	-	-
330	NPPC	-	+	-	-
330	OAZ2	+	-	-	-
331	TCIRG1	+	-	-	-
332	C8orf46	+	-	-	-
332	PACSIN1	-	+	-	-
333	CPN2	+	-	-	-
334	DMRT3	-	+	-	-
334	NLGN4X	+	-	-	-
335	ARL6IP6	+	-	-	-
335	SH3GL3	-	+	-	-
336	NUMA1	+	-	-	-
336	ROPN1L	-	+	-	-
337	C7orf16	-	+	-	-
337	VSTM2A	+	+	-	-
338	LCAT	+	-	-	-
338	S100A3	-	+	-	-
339	HISPPD2A	+	-	-	-
339	RNF182	-	+	-	-
340	CUTL2	-	+	-	-
340	LINGO2	+	+	-	-
341	LGR6	-	+	-	-
341	WHSC1L1	+	-	-	-
342	RSRC1	-	+	-	-
342	VMAC	+	-	-	-
343	ANGPTL7	-	+	-	-
344	GABRB3	-	+	-	-
344	SPIB	+	-	-	-
345	AGPAT5	+	-	-	-
345	PI3	-	+	-	-
346	PLCH2	+	-	-	-
347	C11orf60	-	+	-	-
347	UNQ473	+	-	-	-
348	C16orf45	+	-	-	-

348	HAS3	-	+	-	-
349	EN2	+	-	-	-
350	FBXO32	+	-	-	-
350	TTC12	-	+	-	-
351	CDKN2B	-	+	-	-
351	COL15A1	+	-	-	-
352	DKK4	+	-	-	-
352	RHOB	-	+	-	-
353	NOV	-	+	-	-
353	NR2C2	+	-	-	-
354	ABCA5	-	+	-	-
355	P11	+	-	-	-
355	SOD3	-	+	-	-
356	POPDC3	-	+	-	-
357	CLUL1	-	+	-	-
357	LRRC37B	+	-	-	-
358	SAPS1	-	+	-	-
358	TRIP4	+	-	-	-
359	SLFN13	-	+	-	-
359	SPACA3	+	-	-	-
360	AIM1L	+	-	-	-
360	FAM64A	-	+	-	-
361	BRS3	+	-	-	-
362	MDM2	-	+	-	-
362	MYO1D	+	-	-	-
363	BLVRA	+	-	-	-
363	CNOT8	-	+	-	-
364	NR1I3	+	-	-	-
364	SCAND1	-	+	-	-
365	EPB41L3	-	+	-	-
365	FPR1	+	-	-	-
366	C19orf39	+	-	-	-
366	MFAP4	-	+	-	-
367	AIM2	-	+	-	-
367	SURF4	+	-	-	-
368	G30	-	+	-	-
368	SGMS1	+	-	-	-
369	DEFA3	-	+	-	-
369	GOT1	+	-	-	-
370	PMCH	-	+	-	-
370	REEP5	+	-	-	-
371	FBXO25	-	+	-	-
371	RNF181	+	-	-	-
372	DSN1	+	-	-	-
372	RUNDC1	-	+	-	-
373	GAS1	-	+	-	-
374	HYMAI	-	+	-	-
374	MCL1	+	-	-	-
375	FLJ22662	-	+	-	-
375	KLK5	+	-	-	-
377	BIRC7	+	-	-	-
378	BANP	+	-	-	-

378	SOSTDC1	-	+	-	-
379	CX3CR1	-	+	-	-
380	DSCAM	-	+	-	-
380	MGC21881	+	-	-	-
381	RBM17	-	+	-	-
382	SLC7A11	+	-	-	-
382	SNAI1	-	+	-	-
383	DYDC2	-	+	-	-
383	H2AFY2	+	-	-	-
384	CLIC6	-	+	-	-
384	TMEM25	+	-	-	-
385	FOXD3	+	-	-	-
385	PCDHA3	-	+	-	-
386	PLAU	-	+	-	-
386	ZNF530	+	-	-	-
387	C5orf39	-	+	-	-
387	PROS1	+	+	-	+
388	CKAP4	+	-	-	-
389	FBXO7	+	-	-	-
389	FOXN4	-	+	-	-
390	CDK8	-	+	-	-
391	CA8	-	+	-	-
392	INTS7	+	-	-	-
392	RND3	-	+	-	-
393	C8orf22	-	+	-	-
394	CSDE1	+	-	-	-
394	CXCL12	-	+	-	-
395	C17orf53	-	+	-	-
395	TH	+	-	-	-
396	DHRS7B	+	-	-	-
396	UTY	-	+	-	-
397	NDUFB10	-	+	-	-
398	CBLN4	-	+	-	-
398	IKIP	+	-	-	-
399	BCAS3	+	-	-	-
399	GSC	-	+	-	-
400	CPNE8	-	+	-	-
400	TAF5	+	-	-	-
401	C16orf35	+	-	-	-
402	CNDP2	+	-	-	-
402	HLA-DRB6	-	+	-	+
403	FLJ40292	+	-	-	-
403	STAC	-	+	-	-
404	BTC	-	+	-	-
404	KCTD21	+	-	-	-
405	GNG11	-	+	-	-
405	OR10T2	+	-	-	-
406	CPNE4	+	+	-	-
406	FAM131A	-	+	-	-
407	FLRT2	-	+	-	-
408	C10orf63	+	-	-	-
408	CHRNA3	-	+	-	-

409	DDX56	-	+	-	-
409	LHFPL3	+	-	-	-
410	GRN	+	-	-	-
410	MEX3A	-	+	-	-
411	SNTG2	-	+	-	-
412	CTSH	-	+	-	-
412	TPTE2	+	-	-	-
413	COLEC11	+	+	-	-
413	PEG3	-	+	-	-
414	RICH2	-	+	-	-
414	TNFRSF12A	+	-	-	-
415	NAV3	-	+	-	-
415	PTCD2	+	-	-	-
416	ACP5	-	+	-	-
416	PXDNL	+	-	-	-
417	BTN3A2	+	-	-	-
417	PDGFRL	-	+	-	-
418	LGALS7	-	+	-	-
419	COL24A1	-	+	-	-
419	FLJ40869	+	-	-	-
420	LRRC50	-	+	-	-
420	PAGE2B	+	-	-	-
421	BAAT	-	+	-	-
421	TUB	+	-	-	-
422	ABBA-1	-	+	-	-
422	PRKCB1	+	+	-	-
423	AQP7	-	+	-	-
423	PDE8B	+	-	-	-
424	MGAT4C	-	+	-	-
424	SVIL	+	-	-	-
425	KISS1	+	-	-	-
425	PTPRR	-	+	-	-
427	FGFR3	-	+	-	-
427	LTA4H	+	-	-	-
428	NCOA6	+	-	-	-
428	RPRM	-	+	-	-
429	HFM1	-	+	-	-
430	CMTM6	+	-	-	-
430	MEIS3	-	+	-	-
431	CEMP1	+	-	-	-
431	MEG3	-	+	-	-
432	IFITM5	+	-	-	-
432	REPS2	-	+	-	-
433	ARRDC3	+	-	-	-
433	RAB3C	-	+	-	-
434	KIAA1279	+	-	-	-
434	STAM	-	+	-	-
435	RBP1	+	-	-	-
435	TSHR	-	+	-	-
436	ABHD6	+	-	-	-
436	HADH	-	+	-	-
437	ANK1	+	-	-	-

437	CXCL13	-	+	-	-
438	C15orf39	+	-	-	-
438	RPS3	-	+	-	-
439	DAZL	-	+	-	-
440	CDH1	+	-	-	-
440	FKBP5	-	+	-	-
441	NTN4	+	-	-	-
441	SMPX	-	+	-	-
442	ABLIM1	+	-	-	-
442	PRB1	-	+	-	-
443	PRODH2	+	-	-	-
443	TMEM154	-	+	-	-
444	CRHBP	-	+	-	-
444	PRELID1	+	-	-	-
445	C20orf42	-	+	-	-
445	DUS2L	+	-	-	-
446	SLC9A10	-	+	-	-
447	HOXA3	-	+	-	-
447	PRKCDBP	+	-	-	-
448	GPR22	-	+	-	-
448	RNF213	+	-	-	-
449	C6orf25	+	-	-	-
449	LOC441054	-	+	-	-
450	OSR1	-	+	-	-
450	PLAC2	+	-	-	-
451	EVI2A	-	+	-	-
451	GNAL	+	-	-	-
452	C18orf26	+	-	-	-
452	ELOVL2	-	+	-	-
453	IFIT2	+	-	-	-
453	MGC4294	-	+	-	-
454	CFB	-	+	-	-
454	CNTNAP5	+	-	-	-
455	STT3B	+	-	-	-
455	ZNF451	-	+	-	-
456	CCDC142	-	+	-	-
456	FLJ16369	+	-	-	-
457	ASPN	+	-	-	-
457	LOC554174	-	+	-	-
458	CFH	-	+	-	-
458	ZNF44	+	-	-	-
459	KIF13B	+	-	-	-
459	LOC136288	-	+	-	-
460	CLEC4M	+	-	-	-
460	CPNE5	-	+	-	-
461	EXOSC3	+	-	-	-
461	TMEM16D	-	+	-	-
462	EDNRB	+	-	-	-
463	IL20RA	-	+	-	-
463	UBE2V2	+	-	-	-
464	LRRN3	-	+	-	-
464	VHLL	+	-	-	-

465	ABCC2	+	-	-	-
465	RSPO2	-	+	-	-
466	FABP6	-	+	-	-
466	KIAA0701	+	-	-	-
467	ARHGAP6	-	+	-	-
467	MAGEA9	+	-	-	-
468	C3orf10	-	+	-	-
468	CLMN	+	-	-	-
469	C8orf13	-	+	-	-
469	CD79A	+	-	-	-
470	EMP2	-	+	-	-
470	HSPE1	+	-	-	-
471	DLX6	-	+	-	-
471	LYPLA2	+	-	-	-
472	FCGR2B	+	-	-	-
472	LOC441046	-	+	-	-
473	KIAA1598	-	+	-	-
473	P2RY6	+	-	-	-
474	KCND2	+	-	-	-
474	LMO1	-	+	-	-
475	CTRL	-	+	-	-
475	DIABLO	+	-	-	-
476	hCG_1990170	-	+	-	-
476	MAML1	+	+	-	-
477	C1QL1	-	+	-	-
477	PYROXD1	+	+	-	-
478	DBX2	+	-	-	-
478	PPP1R16A	-	+	-	-
479	RNASET2	+	-	-	-
479	ZNF799	-	+	-	-
480	RFPL2	-	+	-	-
480	SRPX2	+	-	-	-
481	C9orf58	+	-	-	-
481	CSPG4	-	+	-	-
482	OSBPL10	+	-	-	-
482	TNFRSF25	-	+	-	-
483	CYB5R4	+	-	-	-
483	HOXC8	-	+	-	-
484	C10orf140	-	+	-	-
485	CLDN23	-	+	-	-
485	HTR3D	+	-	-	-
486	HCN4	+	-	-	-
486	LRFN5	-	+	-	-
488	FAM130A2	-	+	-	-
488	TNIP2	+	-	-	-
489	ITPKB	+	-	-	-
489	KIAA2022	-	+	-	-
490	MSH6	+	-	-	-
490	PDK4	-	+	-	-
491	C10orf35	-	+	-	-
492	ARPC1B	+	-	-	-
492	ENPP2	-	+	-	-

493	B4GALT6	-	+	-	-
493	FLJ45557	+	-	-	-
494	LAMP3	-	+	-	-
495	HUWE1	+	-	-	-
495	NBLA00301	-	+	-	-
496	NSUN7	-	+	-	-
496	STARD8	+	-	-	-
497	MCF2	-	+	-	-
497	NPR2	+	-	-	-
498	ZDHHC23	-	+	-	-
499	ASAH1	+	-	-	-
499	SLFN11	-	+	-	-
500	CCDC105	+	-	-	-
500	CPAMD8	-	+	-	-
501	ANLN	+	-	-	-
501	HIST1H2BJ	-	+	-	-
502	LRAP	-	+	-	-
502	STXBP5L	+	+	-	-
503	CEP68	+	-	-	-
503	FLJ21963	-	+	-	-
504	C20orf175	+	-	-	-
504	MYBPH	-	+	-	-
505	LYVE1	+	-	-	-
505	RET	-	+	-	-
506	ACN9	-	+	-	-
506	C1orf80	+	-	-	-
507	C22orf24	-	+	-	-
507	WDR62	+	-	-	-
508	NLGN2	-	+	-	-
508	SIDT1	+	-	-	-
509	CCBL1	+	-	-	-
509	PCDHB6	-	+	-	-
510	AGTR1	+	-	-	-
510	COQ7	-	+	-	-
511	KLHDC1	+	-	-	-
511	TTC29	-	+	-	-
512	NMU	+	-	-	-
512	RAMP3	-	+	-	-
513	C8orf53	+	-	-	-
513	EID3	-	+	-	-
514	HILS1	-	+	-	-
515	CA3	+	-	-	+
515	CCDC122	-	+	-	-
516	TRHDE	-	+	-	-
516	VCL	+	-	-	-
517	CSTA	-	+	-	-
517	PHOX2B	+	-	-	-
518	CCND2	-	+	-	-
518	FGFBP3	+	-	-	-
519	C1orf91	+	-	-	-
519	ZNF233	-	+	-	-
520	ADAMTS5	-	+	-	-

520	KIAA0157	+	-	-	-
521	C19orf7	+	-	-	-
521	LOC644186	-	+	-	-
522	AKAP6	+	-	-	-
522	F12	-	+	-	-
523	UBE3B	-	+	-	-
524	IL1R2	-	+	-	-
524	PPYR1	+	-	-	-
525	BEST2	+	-	-	-
525	DSPP	-	+	-	-
526	LMNB1	-	+	-	-
527	AKR1B10	+	-	-	-
527	RORB	-	+	-	-
528	ITIH2	+	-	-	-
528	MARVELD3	-	+	-	-
529	AGR2	-	+	-	-
530	SLC39A3	+	-	-	-
530	SPRY4	-	+	-	-
531	NRBP2	-	+	-	-
531	SMARCD2	+	-	-	-
532	CCDC136	-	+	-	-
532	KLHL6	+	-	-	-
533	CLDN2	+	-	-	-
533	ZNF701	-	+	-	-
534	C19orf47	+	-	-	-
534	DPEP1	-	+	-	-
535	BMP7	-	+	-	-
535	UXS1	+	-	-	-
536	KLHL29	-	+	-	-
536	TMEM166	+	-	-	-
537	BSN	+	-	-	-
537	MATN4	-	+	-	-
538	ADMR	+	-	-	-
538	LRRC16	-	+	-	-
539	EPHA5	-	+	-	-
540	AKAP12	-	+	-	-
541	C6orf142	+	-	-	-
541	CYP2D6	-	+	-	-
542	KERA	+	-	-	-
543	OASL	-	+	-	-
543	ZSCAN18	+	-	-	-
544	PTGS1	-	+	-	-
544	SRI	+	-	-	-
545	C21orf7	-	+	-	-
545	DHX16	+	-	-	-
546	RNGTT	-	+	-	-
546	SCN4A	+	-	-	-
547	FAM62B	-	+	-	-
547	ZNF767	+	-	-	-
548	NPY2R	-	+	-	-
549	LIMS3	-	+	-	-
549	TMC5	+	-	-	-

550	ZNF311	+	-	-	-
551	KLK15	+	-	-	-
552	DOCK7	+	-	-	-
552	HOXA2	-	+	-	-
553	MAPT	+	-	-	-
553	TPM2	-	+	-	-
554	DNM3	+	-	-	-
554	PRICKLE1	-	+	-	-
555	NOS2A	-	+	-	-
555	RASL12	+	-	-	-
556	PERQ1	-	+	-	-
556	SLC46A3	+	-	-	-
557	C20orf196	+	-	-	-
557	CRYBA2	-	+	-	-
558	CHRNA10	-	+	-	-
558	CTSK	+	+	-	-
559	RPL21	+	-	-	-
560	PFKL	+	-	-	-
561	CXCL6	-	+	-	-
561	TIP39	+	-	-	-
562	F2RL3	-	+	-	-
562	PNMA5	+	-	-	-
563	FZD3	+	-	-	-
563	UGT8	-	+	-	-
565	ALK	-	+	-	-
566	USP43	-	+	-	-
567	TSPAN2	-	+	-	-
568	EPHX2	-	+	-	-
569	PCDHGB7	-	+	-	-
570	LECT1	-	+	-	-
571	DSCR10	-	+	-	-
572	FCRLA	-	+	-	-
573	LNX1	-	+	-	-
574	HSPB7	-	+	-	-
575	C1orf85	-	+	-	-
576	FOXF1	-	+	-	-
577	HOXD11	-	+	-	-
578	KCTD14	-	+	-	-
579	NOX4	-	+	-	-
580	C6orf117	-	+	-	-
581	HSD17B6	-	+	-	-
582	RARRES1	-	+	-	-
583	PNPLA4	-	+	-	-
584	TMEM45A	-	+	-	-
585	PPP1R1A	-	+	-	-
586	AGBL2	-	+	-	-
587	TSPYL4	-	+	-	-
588	ATRNL1	-	+	-	-
589	SLC35F3	-	+	-	-
590	TMEM98	-	+	-	-
592	CMTM8	-	+	-	-
594	GAD1	-	+	-	-

595	MEIS2	-	+	-	-
596	LRAT	-	+	-	-
597	CASKIN1	-	+	-	-
599	FBXL2	-	+	-	-
600	SERPINE1	-	+	-	-
601	MAP3K8	-	+	-	-
602	CCDC19	-	+	-	-
603	MEX3B	-	+	-	-
604	BAIAP3	-	+	-	-
605	JPH3	-	+	-	-
606	ARP11	-	+	-	-
608	ASPHD1	-	+	-	-
610	BCL2A1	-	+	-	-
611	PCK1	-	+	-	-
612	ARPP-21	-	+	-	-
613	OPLAH	-	+	-	-
614	LYSMD3	-	+	-	-
615	HHAT	-	+	-	-
617	HIST1H4J	-	+	-	-
618	BEGAIN	-	+	-	-
619	PALM2-AKAP2	-	+	-	-
620	RICS	-	+	-	-
621	RBP7	-	+	-	-
622	OAS1	-	+	-	-
623	CCDC141	-	+	-	-
624	FAM90A1	-	+	-	-
625	C12orf63	-	+	-	-
626	FAM129A	-	+	-	-
627	CARTPT	-	+	-	-
628	SCGN	-	+	-	-
629	EFCAB1	-	+	-	-
630	TNMD	-	+	-	-
631	IFI16	-	+	-	-
632	PHC3	-	+	-	-
633	ZNF287	-	+	-	-
634	C22orf32	-	+	-	-
635	UBE2A	-	+	-	-
636	C10orf10	-	+	-	-
637	PLS3	-	+	-	-
638	TLN2	-	+	-	-
639	ZNF28	-	+	-	-
640	USP51	-	+	-	-
641	MICAL1	-	+	-	-
642	FAM89A	-	+	-	-
643	LIPG	-	+	-	-
644	ACTG2	-	+	-	-
645	CHRM2	-	+	-	-
646	HS6ST3	-	+	-	-
647	SKAP2	-	+	-	-
648	DNAJA4	-	+	-	-
650	MYCBPAP	-	+	-	-
651	SLC5A11	-	+	-	-

652	F2R	-	+	-	+
653	HIST1H4D	-	+	-	-
654	CRYBA1	-	+	-	-
655	NEUROD1	-	+	-	-
656	OPRK1	-	+	-	-
657	PIP5K1B	-	+	-	-
658	FAS	-	+	-	-
659	ACMSD	-	+	-	-
660	FOS	-	+	-	-
661	IRF4	-	+	-	-
662	MAPRE1	-	+	-	-
664	GLT8D4	-	+	-	-
665	FGF1	-	+	-	-
666	FLJ46266	-	+	-	-
667	BST2	-	+	-	-
668	UTRN	-	+	-	-
669	AMICA1	-	+	-	-
670	KIAA1913	-	+	-	-
671	TMEM74	-	+	-	-
672	BBOX1	-	+	-	-
673	KIAA1257	-	+	-	-
674	FAM96B	-	+	-	-
676	TXLNB	-	+	-	-
677	DDIT3	-	+	-	-
678	CDH4	-	+	-	-
679	TRRAP	-	+	-	-
680	CD2	-	+	-	-
681	GAL3ST1	-	+	-	-
682	STEAP1	-	+	-	-
683	RLBP1	-	+	-	-
684	MTBP	-	+	-	-
685	CXCR7	-	+	-	-
686	CAPZA2	-	+	-	-
687	MGRN1	-	+	-	-
688	POLR2E	-	+	-	-
690	CAPS	-	+	-	-
691	COL4A4	-	+	-	-
692	CACNA2D3	-	+	-	-
693	LONRF3	-	+	-	-
694	POPDC2	-	+	-	-
695	MDFI	-	+	-	-
696	PCDHGA8	-	+	-	-
697	LOC440248	-	+	-	-
698	NDRG1	-	+	-	-
699	MREG	-	+	-	-
700	TM4SF20	-	+	-	-
701	GALNAC4S-6ST	-	+	-	-
703	CYR61	-	+	-	-
704	TUBB2C	-	+	-	-
705	BCL3	-	+	-	-
706	NPPB	-	+	-	-
707	PLK2	-	+	-	-

708	CYP27B1	-	+	-	-
710	C15orf5	-	+	-	-
711	SERPINF1	-	+	-	-
712	CRYGD	-	+	-	-
713	CNTNAP2	-	+	-	-
714	OR52N1	-	+	-	-
715	RNF150	-	+	-	-
716	SSTR1	-	+	-	-
717	ENTHD1	-	+	-	-
718	LDHC	-	+	-	-
719	GYG2	-	+	-	-
720	CPVL	-	+	-	-
721	CCDC102A	-	+	-	-
722	FLJ32549	-	+	-	-
723	IL7R	-	+	-	-
724	C11orf24	-	+	-	-
725	ARHGAP22	-	+	-	-
726	C7orf51	-	+	-	-
727	EWSR1	-	+	-	-
728	FAM19A3	-	+	-	-
729	ACOX2	-	+	-	-
731	TK1	-	+	-	-

Table S5: UFF selected genes for the TCGA ovarian serous cystadenocarcinoma datasets (Agilent and Affymetrix platforms).

Minimal rank	Gene	Appears in Agilent	Appears in Affymetrix
1	IGF2	+	+
1	ZNF483	+	-
2	HOXA4	+	+
2	SST	-	+
3	POSTN	+	+
3	RP13-36C9.6	+	-
4	DEFB1	-	+
4	HPR	+	-
5	HLA-DQA1	-	+
5	LMO3	+	+
6	COL11A1	-	+
6	PAGE2	+	-
7	AGR3	+	-
7	ZIC1	+	+
8	HOXA9	+	+
8	PCP4	+	+
9	OVGP1	-	+
9	PON3	+	+
10	CXCL1	+	+
10	DPEP3	-	+
11	C6orf54	+	-
11	NLRP2	-	+
12	C3orf41	+	-
12	TMEM100	+	+

13	ATP1A2	+	-
13	IGKC	-	+
14	C10orf10	+	-
14	PROM1	+	+
15	AGR2	-	+
15	IL13RA2	+	+
16	CRABP1	+	+
16	MAGEA9	+	+
17	MAL	+	+
17	SERPINA5	+	+
18	CDKN2A	+	+
18	MMP7	-	+
19	DLK1	-	+
19	PAGE4	+	-
20	KRT23	+	+
20	SCGB1D2	-	+
21	CDH18	+	+
21	CRISP3	-	+
22	PCK1	-	+
22	POPDC2	+	-
23	KLK5	-	+
24	BEX1	-	+
24	EYA4	+	-
25	SHANK1	+	-
25	TSPAN1	-	+
26	SOSTDC1	+	+
26	TSPAN8	+	+
27	PAGE2B	+	-
27	VTCN1	-	+
28	DACH2	+	-
28	HBB	-	+
29	SERPINA3	-	+
29	UBD	+	+
30	FABP4	-	+
31	CLDN10	+	+
31	PAGE5	+	-
32	C5orf23	+	+
32	CYP4B1	-	+
33	CLGN	+	+
33	HMGA2	-	+
34	RPL36A	+	-
35	LDLRAD1	+	-
35	PTGDS	-	+
36	MSX1	-	+
36	TRHDE	+	+
37	ST6GALNAC5	-	+
38	NTS	-	+
40	RAB25	+	+
40	UGT2B7	+	-
41	HP	-	+
41	NPTX2	+	+
42	PNOC	-	+

42	ZDHHC11	+	+
43	LTF	-	+
43	NOX4	+	+
44	CA8	+	+
44	RARRES1	-	+
45	DCDC2	+	-
45	GPR64	-	+
46	CXCL14	+	+
46	FABP6	+	+
47	ATP6V1E1	+	-
47	CXCL10	-	+
48	APOA1	-	+
49	PTH2R	-	+
50	FUT6	+	-
50	S100A2	-	+
51	OGN	+	+
51	OXTR	-	+
52	EXPH5	+	-
52	HBA2	-	+
53	IGKV1-5	-	+
53	MGC10701	+	-
54	GNGT1	+	-
54	SCGB2A1	-	+
55	C10orf116	-	+
55	TTC13	+	-
56	FAM133A	+	-
56	SCG5	+	+
57	CCNA1	-	+
57	TDRD9	+	-
58	PDCL2	+	-
58	TKTL1	-	+
59	PRSS21	-	+
59	ZBTB33	+	-
60	PTN	-	+
60	ZFP42	+	-
61	ARMC4	+	-
61	ZBED2	-	+
62	CTTNBP2	+	-
62	PI3	-	+
63	MMP1	-	+
63	SLITRK5	+	+
64	C14orf105	+	-
64	CHL1	-	+
65	KHDRBS2	+	-
66	CTNNBIP1	+	-
66	HOXA5	-	+
67	CFTR	+	-
67	TUBB2B	-	+
68	CXCL2	-	+
68	TIMM8A	+	-
69	CXCL9	-	+
69	NUP133	+	-

70	MMP10	+	+
70	NNMT	+	+
71	HSPA1A	+	+
71	VAV3	+	+
72	SLITRK6	+	-
72	TMSL8	-	+
73	LCN2	-	+
73	LHX4	+	-
74	BCAT1	+	+
74	FREM1	+	-
75	MT1G	+	+
75	SFRP2	+	-
76	HPGD	-	+
76	PROK2	+	-
77	CLDN16	-	+
77	NTF5	+	-
78	SEMA3E	+	-
78	UCHL1	+	+
79	CPM	+	-
79	LAMA3	-	+
80	ABP1	-	+
81	LUM	-	+
81	RNF182	+	-
82	MT1H	+	-
82	TFPI2	-	+
83	GLDC	-	+
83	PCK2	+	-
84	THBS2	-	+
84	WDR72	+	-
85	TIMP2	+	-
85	TSPAN7	-	+
86	BNC1	+	+
87	CYP1B1	+	+
88	PDZK1IP1	-	+
88	SLC27A6	+	+
89	BHMT2	+	-
89	SERPINE2	-	+
90	EPB42	+	-
90	TDO2	-	+
91	CXorf38	+	-
91	HOXB6	-	+
92	BBOX1	-	+
92	DYNLRB2	+	-
93	KLK11	+	+
93	MUC16	-	+
94	AKR1C3	-	+
94	OLFM4	+	+
95	ACTG2	-	+
96	ANPEP	-	+
96	DSC3	+	-
97	MLC1	+	-
98	TTC29	+	-

99	DACT2	+	-
99	S100A9	-	+
100	AFAP1L2	+	-
100	S100A8	-	+
101	COL5A2	-	+
101	SPP1	+	-
102	COL9A1	+	+
102	KRT14	-	+
103	CCL20	-	+
103	SNIP1	+	-
104	ASPN	-	+
104	KCNMB4	+	+
105	EPYC	-	+
105	INDO	+	+
106	NDN	+	+
106	STXBP6	-	+
107	FGF9	+	+
107	PTX3	-	+
108	FCGBP	-	+
109	COL13A1	+	-
109	MAGEA11	-	+
110	LYZ	-	+
110	SOX11	+	+
111	GALNT14	+	+
111	ID1	-	+
112	ADRA2C	+	-
112	FBN2	-	+
113	IGF2BP3	-	+
113	NDP	+	+
114	CNN1	+	+
114	KRT6A	-	+
115	KLK6	-	+
115	PDE1A	+	+
116	EGR3	+	+
116	LRRC15	-	+
117	CSF3	+	-
117	UPK1B	-	+
118	FGFR1	+	-
118	HTR3A	-	+
119	GLDN	+	-
119	MFAP5	-	+
120	HNRPH2	+	-
120	NEFH	+	+
121	KRT12	+	-
121	TNNT1	-	+
122	GPC5	+	+
123	DMBT1	-	+
123	FLRT3	+	+
124	CILP	-	+
124	HBD	+	-
125	EDN3	+	-
125	PRAME	-	+

126	CTCFL	+	-
127	GSTA2	+	-
127	IGLV3-25	-	+
128	FZD10	-	+
128	MYBPHL	+	-
129	ABI3BP	+	-
129	DNAJC15	+	+
130	GSTT1	+	+
130	LEFTY1	+	+
131	G30	+	-
132	COL10A1	-	+
132	IL7	+	-
133	C10orf140	+	-
133	HOXD1	-	+
134	GDF10	+	-
134	REG1A	-	+
135	FRZB	-	+
135	ZNF750	+	+
136	FAP	+	+
136	TAGLN	-	+
137	CLCA1	+	+
137	GATA6	-	+
138	FAM26F	+	-
138	SULF1	-	+
139	GPX3	-	+
140	ESR1	+	-
140	PAEP	+	+
141	IFIT1	-	+
141	MEIS2	+	+
142	GLULD1	+	-
142	TFF3	+	+
143	GPRC5A	-	+
143	TMEM98	+	-
144	CAMP	+	-
144	HLA-DQB1	-	+
145	CP	+	+
146	C18orf26	+	-
146	C7	-	+
147	MAPK8IP2	+	-
147	VCAM1	-	+
148	ATP6V1B1	-	+
148	MMP12	+	+
149	CRYAB	-	+
149	PCDHB5	+	-
150	P2RX2	+	-
151	COLEC11	-	+
151	CTHRC1	+	-
152	HAPLN1	+	-
152	INHBB	-	+
153	IGFL2	+	-
154	C8orf4	+	+
154	NRF1	+	-

155	AMIGO2	-	+
155	KCNK1	+	+
156	THSD4	-	+
157	C15orf44	+	-
157	EYA2	-	+
158	HNRPUL1	+	-
159	HSPB3	+	-
159	KIF1A	-	+
160	CAMK2N1	-	+
160	HAS3	+	-
161	MFAP2	-	+
162	IFI44L	-	+
162	WNT16	+	-
163	KLK7	-	+
163	RUNX3	+	+
164	ALK	+	-
166	LRRC17	-	+
166	NAT8L	+	-
167	ITIH5	-	+
167	MBTD1	+	-
168	FOXN4	+	-
168	HIST1H1C	+	+
169	PRB1	+	-
169	SCGB1D1	+	+
170	BASP1	-	+
170	ZNF300	+	-
171	PTTG3	+	-
171	VCAN	-	+
172	CXCL11	-	+
172	CXXC4	+	-
173	CHRM5	+	-
173	MMP9	-	+
174	ATAD4	+	+
174	GOLSYN	-	+
175	TNC	-	+
176	MT1E	-	+
177	CRYGC	+	-
177	CTSK	-	+
178	PTGIS	+	-
179	C1orf168	+	-
179	SFRP1	-	+
180	GALP	+	-
180	LAMP3	-	+
181	ADAMDEC1	-	+
181	FLG	+	+
182	LRAP	-	+
183	COMP	-	+
183	GPR158	+	-
184	PLCXD3	+	-
184	SFRP4	-	+
185	EGR1	+	+
185	NELL2	-	+

186	FLJ22655	+	-
187	CXCR7	+	+
187	IGSF3	+	-
188	SERPINF1	-	+
188	TAC1	+	-
189	KCNJ16	-	+
189	MOGAT2	+	-
190	COL5A1	-	+
190	CXCL12	+	+
191	CDH16	+	-
191	FMO2	-	+
192	DPP4	+	-
192	TCEAL2	-	+
193	C8orf48	+	-
193	FOS	+	+
194	ATF3	-	+
194	TMEM130	+	-
195	FAM107A	-	+
196	KIAA1505	+	-
197	NPY	-	+
197	PCDHB3	+	-
198	S100A14	+	+
199	FOLR1	-	+
199	SULT1C2	+	+
200	NMU	+	+
200	S100A7	-	+
201	CRTAC1	-	+
201	MUCL1	+	-
202	HLA-DMA	-	+
202	SRPR	+	-
203	C12orf63	+	-
203	HCP5	-	+
204	TNNC1	-	+
204	UGT2B11	+	-
205	COL24A1	+	-
205	TPPP3	-	+
206	SPRY2	-	+
206	TSPAN19	+	-
207	TMEM16A	-	+
208	BTNL2	+	-
208	DCN	-	+
209	GCNT3	+	+
210	EMX2	-	+
210	MGC32805	+	-
211	NRBP2	+	-
211	RTP4	-	+
212	BTF3	+	-
212	SERPINA1	-	+
213	COX7B2	+	-
213	PLA2G4A	-	+
214	ALOX5AP	-	+
214	UQCRH	+	-

215	FOSB	-	+
215	TBX2	+	-
216	MAGEC2	+	+
217	DACH1	-	+
217	RASEF	+	-
218	EFEMP1	-	+
218	ZADH2	+	-
219	UPK3B	-	+
220	MEOX1	-	+
220	MT1B	+	-
221	KAL1	-	+
221	SELV	+	-
222	C1QB	-	+
222	VNN1	+	+
223	CFD	+	+
223	CRIP1	-	+
224	APOBEC3B	-	+
224	STEAP1	+	+
225	CNTNAP1	+	-
225	MGP	-	+
226	EGFL6	-	+
226	hCG_25371	+	-
227	ALDH1A1	+	+
228	D4S234E	-	+
228	LGI1	+	-
229	MLPH	-	+
230	BIRC3	+	+
230	GSTA1	-	+
231	PCDHB10	+	-
231	TMPRSS4	-	+
232	C1orf106	+	-
232	SERPINB2	-	+
233	KRT5	-	+
234	SCEL	-	+
234	VWC2	+	-
235	C3	-	+
235	EDIL3	+	-
236	ESD	+	-
236	RSAD2	-	+
237	GPC4	+	+
237	ZFPM2	-	+
238	CARTPT	+	-
238	TMEM158	-	+
239	CSTA	+	+
239	FGF13	+	+
240	DCD	+	-
240	TMEM176B	-	+
241	SYT4	+	-
242	NTF3	+	+
242	ST6GALNAC2	+	+
243	DPYD	-	+
243	SLC26A9	+	-

244	ANKRD39	+	-
244	CDH11	-	+
245	CCND1	-	+
245	TCN1	+	-
246	FAM129A	-	+
246	TNFSF12	+	-
247	HYMAI	+	-
247	RARRES3	-	+
248	CHI3L1	-	+
248	RERG	+	-
249	AHNAK2	-	+
249	ZNF556	+	-
250	MAGEA4	-	+
250	OIT3	+	-
251	ALS2CR11	+	-
251	SLC44A4	-	+
252	BMP7	+	-
252	TNFRSF11B	-	+
253	MS4A4A	-	+
253	PIP5K1B	+	-
254	HOPX	-	+
254	RGS9BP	+	-
255	LPHN2	-	+
255	PRSS35	+	-
256	MAOB	-	+
256	TKTL2	+	-
257	ACY1	+	-
257	IFI44	-	+
258	RYR3	+	-
259	TMEM47	-	+
260	HOXB7	-	+
260	SH3RF1	+	-
261	SCRG1	+	-
261	TMOD1	-	+
262	LOC441054	+	-
263	IGF1	-	+
263	PCDHB17	+	-
264	OAS1	-	+
264	TPO	+	-
265	BCAS1	+	-
265	TWIST1	-	+
266	C9orf135	+	-
267	CD52	+	-
268	CFH	-	+
268	MEX3A	+	-
269	CX3CR1	-	+
269	WDR66	+	-
270	ADM	-	+
270	HS3ST3A1	+	+
271	SRPX	-	+
272	OSR2	+	+
272	TNFSF10	-	+

273	KRT17	-	+
273	LGR5	+	+
274	DYNLRB1	+	-
274	NLGN4X	+	+
275	SLC13A2	+	-
275	TTYH1	-	+
276	S100A4	+	+
276	UGT2B17	-	+
277	LPL	+	+
277	NID2	-	+
278	FLJ44379	+	-
278	PHACTR1	-	+
279	MX1	-	+
279	RLN2	+	-
280	KIAA0226	+	-
280	RGS2	+	+
281	FBXO2	+	+
282	HLA-DRA	-	+
282	TIMP4	+	-
283	GRIA2	-	+
283	LINGO2	+	-
284	A2M	+	-
284	CD200	-	+
285	EAF1	+	-
285	SPON1	-	+
286	LCE1B	+	-
287	CA12	-	+
287	HELLS	+	-
288	C4orf31	+	+
288	PLAC8	-	+
289	FGF1	+	-
289	GSTM3	-	+
290	DHRS9	+	-
290	PLAGL1	-	+
291	FAM55D	+	-
291	TSPYL5	-	+
292	ENTPD7	+	-
293	ANKS6	+	-
293	GPR37	-	+
294	ATP10B	-	+
294	GSTA3	+	-
295	IHH	+	-
296	CHGA	-	+
297	CD163	-	+
297	HRH1	+	+
298	CCL28	+	-
298	GUCY1A3	-	+
299	IGFBP2	+	+
299	TSPAN12	-	+
300	AKAP12	-	+
300	C22orf24	+	-
301	NDUFB10	+	-

301	PLAT	-	+
302	BST2	+	+
302	C17orf67	+	-
303	CTGF	-	+
303	FGF12	+	-
304	IQCG	-	+
304	TSN	+	-
305	CADPS	+	-
305	GABRP	-	+
307	COL9A2	-	+
307	SLC23A1	+	-
308	MXRA5	-	+
308	PRTFDC1	+	-
309	CLIC3	-	+
309	NR2F1	+	-
310	C6orf124	+	-
310	VSIG4	-	+
311	CD14	-	+
311	ZNF771	+	-
312	EMP2	+	-
313	DPPA2	+	-
313	NME5	-	+
314	C1QA	-	+
314	MEGF11	+	-
315	NXF3	+	+
315	ODZ4	-	+
316	TFAP2C	+	-
316	ZNF423	-	+
317	CASC1	-	+
317	KLRG2	+	-
318	HOXC6	-	+
318	NPY2R	+	-
319	MT1JP	+	-
320	ITM2C	-	+
320	SDPR	+	-
321	PTPRR	+	-
322	C18orf45	+	-
322	NT5E	-	+
323	HIST1H3H	-	+
323	PCBD2	+	-
324	PTPN13	-	+
325	CEL	-	+
325	GALNAC4S-6ST	+	+
326	HERC5	-	+
326	PENK	+	+
327	hCG_1990170	+	-
327	IFI6	-	+
328	DEFA5	-	+
328	VWCE	+	-
329	OSTbeta	+	-
329	WISP3	-	+
330	CEACAM6	-	+

330	MFAP4	+	+
331	C20orf39	-	+
331	SLCO2A1	+	-
332	NEBL	+	-
333	C17orf81	-	+
333	FBXL16	+	-
334	CDH17	+	+
334	RGS1	-	+
335	GREM1	-	+
336	FAM26A	+	-
336	PSMB9	-	+
337	LOC644186	+	-
337	PIPOX	-	+
338	GPR137C	+	-
338	QPCT	-	+
339	DLEU7	+	-
339	KIAA1199	-	+
340	JAM2	+	+
341	CA2	-	+
341	RP13-102H20.1	+	-
342	DEFB103A	+	-
343	CRISPLD2	-	+
344	ZNF224	+	-
345	IL1B	+	+
345	PLAU	-	+
346	PPAPDC1A	+	-
346	PVRL3	-	+
347	MEOX2	+	-
347	S100P	-	+
348	GRHPR	+	-
349	C6	-	+
349	KLHL29	+	-
350	ARSJ	+	-
350	ISG15	-	+
351	CCL2	-	+
352	BAAT	+	-
352	MALL	-	+
353	LMO1	+	-
353	VGLL1	-	+
354	CDH12	+	+
355	FCGR2B	-	+
355	ZDHHC15	+	-
356	LRP4	-	+
356	ZDBF2	+	-
357	GABRE	-	+
357	RAB39B	+	-
358	FAT	-	+
358	SLC22A16	+	-
359	NGEF	+	-
359	SNAI2	-	+
360	COL9A3	-	+
360	SH2D2A	+	-

361	GUCY1B3	-	+
362	BAMBI	-	+
362	SLC35F3	+	-
363	PKIB	+	-
363	TOX3	-	+
364	GPC3	-	+
364	MGC45438	+	-
365	NTN4	+	-
365	PTPLA	-	+
366	FOXJ1	-	+
366	HS3ST5	+	-
367	AEBP1	-	+
367	MEST	+	-
368	COL3A1	-	+
368	UST	+	-
369	CCK	+	-
370	ID4	-	+
371	CALB1	-	+
371	CNTN4	+	-
372	CYBRD1	-	+
372	LRG1	+	-
373	THY1	-	+
373	UNQ338	+	-
374	CARD6	+	-
374	TMEM45A	-	+
375	HLA-DPB1	-	+
375	TGFB2	+	-
376	NRN1	-	+
376	RASD1	+	-
377	CEACAM5	-	+
377	PLCE1	+	-
378	COLEC12	-	+
378	TUSC3	+	+
379	C1orf129	+	-
380	KCNB1	+	-
380	MMP2	-	+
381	CHODL	-	+
381	MAOA	+	+
382	DAZL	+	-
382	LOC57228	-	+
383	CACNG4	+	-
383	IGHM	-	+
384	IFI27	-	+
384	LONRF2	+	-
385	CDH3	+	+
385	FMO1	-	+
386	CHST6	+	-
386	SERPINI2	-	+
387	ANXA10	+	-
388	TLE2	+	+
389	NBEA	+	+
389	PEG10	-	+

390	RNF212	+	-
390	SLC34A2	-	+
391	FXYD1	-	+
391	THAP2	+	-
392	C9orf39	+	-
392	SFN	-	+
393	HEPH	-	+
394	CLIC5	-	+
394	RAMP1	+	-
395	CCND2	-	+
395	FXYD3	+	+
396	MGLL	-	+
396	ROPN1L	+	-
397	FZD2	-	+
397	ZNF462	+	-
398	C20orf103	-	+
398	HOXA3	+	-
399	C19orf21	+	-
399	HLA-DPA1	-	+
400	GJA1	+	+
400	SULT1E1	+	-
401	C20orf30	+	-
401	DSC2	-	+
402	C14orf132	-	+
402	C6orf173	+	-
403	ADH1B	-	+
403	G0S2	+	-
404	PPP1R16A	+	-
405	AIM1	-	+
405	PTPRT	+	-
406	FAM134B	+	-
407	LY75	-	+
407	SCGB2A2	+	+
408	KIAA1324	+	-
408	TPBG	-	+
409	CDKN2C	-	+
409	PFN2	+	-
410	PAX6	-	+
410	TMEPAI	+	-
411	NUPR1	-	+
411	PLAC9	+	-
412	HIST1H2AE	-	+
412	TGFA	+	-
413	SCGB1A1	-	+
413	UNQ9368	+	-
414	LRFN5	+	-
414	PRSS1	-	+
415	LHX1	-	+
415	MMP13	+	+
416	CACNB4	+	-
416	FLJ23049	-	+
417	GPNMB	-	+

417	PPP1R14C	+	-
418	CALML5	-	+
418	DSCR10	+	-
419	HFM1	+	-
420	DOK5	-	+
420	KCNAB2	+	-
422	ATHL1	+	+
422	WNT5A	-	+
423	CH25H	+	-
423	MYH11	-	+
424	PID1	+	-
424	REG1B	-	+
425	PLA2G2A	-	+
425	WBP2NL	+	-
426	KCTD14	-	+
426	LAMB3	+	+
427	ANGPTL7	+	-
427	ANXA13	-	+
428	ADAMTS1	+	+
429	ADAMTS5	-	+
429	IL7R	+	-
430	CDH1	+	-
430	HIST1H4H	-	+
431	DEPDC6	-	+
431	RGS22	+	-
432	ALDH1A2	-	+
432	CCDC144B	+	-
433	MX2	-	+
434	IL32	-	+
435	POU2F3	-	+
436	ASRGL1	-	+
437	EHF	-	+
438	SPINK1	-	+
439	S100A1	-	+
442	C1orf38	-	+
443	CTSL2	-	+
444	KCNJ2	-	+
445	AREG	-	+
446	IFIT3	-	+
447	THSD7A	-	+
448	RAB38	-	+
449	CDH6	-	+
450	CLDN1	-	+
452	ACPP	-	+
453	PALMD	-	+
454	IGFBP6	-	+
455	ALDH1A3	-	+
456	PCOLCE	-	+
457	COCH	-	+
458	HEY2	-	+
459	GZMB	-	+
460	IGF2BP2	-	+

461	PDGFRL	-	+
462	KHDRBS3	-	+
463	CKB	-	+
464	RELN	-	+
465	TMPRSS3	-	+
466	PCDH17	-	+
468	ABCA8	-	+
469	COL6A3	-	+
470	MAP1B	-	+
471	IL8	-	+
474	GGH	-	+
476	BIK	-	+
477	BCHE	-	+
478	AKR1B10	-	+
479	GEM	-	+
480	PIR	-	+
481	CCNE1	-	+
483	GSPT2	-	+
484	PPAP2C	-	+
485	C3orf14	-	+
486	ACSL5	-	+
487	DDX58	-	+
488	TUBB2A	-	+
489	GOLGA8A	-	+
490	TYROBP	-	+
491	RARRES2	-	+
492	ENPP2	-	+
493	OBP2A	-	+
494	CST6	-	+
495	CHST2	-	+
496	LAMB1	-	+
497	NUAK1	-	+
498	PELI2	-	+
499	IRX5	-	+
500	ENC1	-	+
501	IL11RA	-	+
502	BAG2	-	+
503	CYR61	-	+
504	MSLN	-	+
505	TCF7L1	-	+
506	HERC6	-	+
508	LRRC32	-	+
509	KIF5C	-	+
510	CPVL	-	+
511	OAS3	-	+
512	SDC2	-	+
513	DPYSL3	-	+
514	ZBTB16	-	+
515	KLK8	-	+
516	SOBP	-	+
517	HSPA12A	-	+
518	CDH2	-	+

519	MATN2	-	+
520	HIST1H2BC	-	+
521	LRRC23	-	+
522	DNALI1	-	+
523	CCL5	-	+
524	PRELP	-	+
525	STC1	-	+
527	SMARCD3	-	+
528	GMPR	-	+
529	F13A1	-	+
530	MPPED2	-	+
531	CBS	-	+
532	CLU	-	+
533	LGALS4	-	+
534	CSRP2	-	+
535	ALCAM	-	+
536	CD53	-	+
537	CA9	-	+
538	PROS1	-	+
539	FCER1G	-	+
540	KRT20	-	+
541	LTB	-	+
543	TYRP1	-	+
544	FLJ22662	-	+
545	ACTA2	-	+
548	HIST2H2BE	-	+
549	HOXB2	-	+
550	FBLN5	-	+
551	PSAT1	-	+
552	EFNB2	-	+
553	HIST1H2BG	-	+
554	C13orf15	-	+
555	EFHD1	-	+
556	MUC1	-	+
557	PLAC1	-	+
558	HEY1	-	+
559	COBL	-	+
561	TMEM176A	-	+
562	MAGEC1	-	+
563	SUSD4	-	+
564	PBK	-	+
565	ADIPOQ	-	+
567	NR4A2	-	+
568	AQP9	-	+
569	SLC5A1	-	+
570	LRP2	-	+
571	TIMP3	-	+
572	TPK1	-	+
573	HSPA2	-	+
574	IGLV2-14	-	+
575	TMC5	-	+
576	SATB1	-	+

578	C2	-	+
579	IGFBP4	-	+
580	ROBO1	-	+
581	PSPH	-	+
582	MS4A6A	-	+
583	AOC3	-	+
584	MLLT11	-	+
585	FMOD	-	+
586	CXCL13	-	+
588	IER3	-	+
590	DDIT4	-	+
591	EDG7	-	+
592	EFHC1	-	+
593	MXRA8	-	+
594	PXDN	-	+
595	SQRDL	-	+
596	CAV1	-	+
597	PLS3	-	+
598	FILIP1L	-	+
600	HLA-DRB1	-	+
601	PLK2	-	+
602	PRKAR2B	-	+
603	PRUNE2	-	+
604	RASGRP1	-	+
605	FOLR3	-	+
606	GAL	-	+
607	WIF1	-	+
608	BLNK	-	+
609	ISG20	-	+
610	PKIA	-	+
611	REG3A	-	+
612	SPOCK1	-	+
613	ENO2	-	+
614	ERBB4	-	+
615	HLA-DMB	-	+
616	ITGB2	-	+
617	SPAG4	-	+
618	ALPL	-	+
619	STON1	-	+
622	TMEFF1	-	+
623	COL1A1	-	+
624	ACSM3	-	+
625	APOL1	-	+
626	IL1R2	-	+
627	CECR1	-	+
628	ISLR	-	+
629	IFIH1	-	+
630	VLDLR	-	+
631	CD69	-	+
632	SEMA3A	-	+
633	MYB	-	+
634	SOD2	-	+

635	CHST1	-	+
636	CCL4	-	+
637	SPA17	-	+
638	GPM6B	-	+
639	NETO2	-	+
640	FXYD6	-	+
642	CADPS2	-	+
643	SERPINE1	-	+
644	FLJ20035	-	+
645	XK	-	+
646	SOX9	-	+
647	EDNRB	-	+
648	HOXD3	-	+
649	CBR3	-	+
650	FABP1	-	+
652	RAP1GAP	-	+
653	XAF1	-	+
654	TEKT2	-	+
655	NQO1	-	+
656	RNASE1	-	+
657	PITPNC1	-	+
658	CST1	-	+
659	HPSE	-	+
660	FST	-	+
661	VEGFC	-	+
662	PART1	-	+
663	SLC26A2	-	+
664	PLEKHB1	-	+
665	LGR4	-	+
666	SIX3	-	+
667	FOXC1	-	+
669	NOV	-	+
670	AADAC	-	+
671	MYLK	-	+
672	DOCK4	-	+
673	PDGFD	-	+
674	LRRC6	-	+
675	THBS4	-	+
676	TGM1	-	+
677	EGLN3	-	+
678	PDZRN3	-	+
679	BANK1	-	+
680	POPDC3	-	+
681	CTSS	-	+
682	TRPS1	-	+
685	EBI2	-	+
687	SLC22A4	-	+
688	SDCCAG8	-	+
689	SDC1	-	+
690	COL15A1	-	+
691	EDN1	-	+
692	RNASE4	-	+

693	TRIM22	-	+
694	COL4A5	-	+
695	DEFA6	-	+
696	APOC1	-	+
697	OLFML3	-	+
698	ADFP	-	+
699	PPP1R1A	-	+
700	FHL1	-	+
701	EVI2B	-	+
702	OXCT1	-	+
703	GULP1	-	+
704	SPINK4	-	+
705	SERPINB5	-	+
706	REEP1	-	+
707	SLIT2	-	+
708	CD48	-	+
709	RPGR	-	+
710	F2R	-	+
711	RASIP1	-	+
712	ST3GAL1	-	+
713	DLG7	-	+
714	CLEC2B	-	+
715	FAM70A	-	+
717	SNCAIP	-	+
718	RAI2	-	+
719	PTP4A3	-	+
720	IL6	-	+
721	ABCC4	-	+
722	APOD	-	+
723	INA	-	+
724	CYB5R2	-	+
725	ECM2	-	+
726	EVI2A	-	+
727	DUSP6	-	+
728	SLC1A3	-	+
729	TNFAIP6	-	+
730	EFCAB1	-	+
731	ARNTL2	-	+
732	ZFP36	-	+
733	GPR177	-	+
734	SMPX	-	+
735	NCALD	-	+
736	PAX2	-	+
737	FZD3	-	+
738	FYN	-	+
739	IL15	-	+
741	CSAD	-	+
742	SYNGR3	-	+
743	PDYN	-	+
744	ST3GAL6	-	+
745	AIM2	-	+
746	HOXC4	-	+

747	RNASE6	-	+
748	APLP1	-	+
749	HDAC9	-	+
750	CTA-246H3.1	-	+
752	SERPINB1	-	+
753	SGCG	-	+
754	LAPTM5	-	+
755	CCL18	-	+
756	CCL11	-	+
757	HCLS1	-	+
758	SPRY4	-	+
759	FBXO17	-	+
760	PTBP2	-	+
761	PCSK1N	-	+
762	LRIG1	-	+
763	DIRAS2	-	+
764	GLT8D2	-	+
765	KCNMB2	-	+
766	GRAMD3	-	+
767	MOCOS	-	+
768	HOXB5	-	+
769	MFHAS1	-	+
770	ANXA1	-	+
771	CRABP2	-	+
772	TRIB2	-	+
773	FBN1	-	+
774	C1orf34	-	+
775	IGFBP3	-	+
777	PDE9A	-	+
778	DHRS3	-	+
779	AR	-	+
780	MMD	-	+
781	ALPPL2	-	+
782	CACNA2D3	-	+
783	TAP1	-	+
784	ADRA2A	-	+
785	PDZD2	-	+
786	MFGE8	-	+
787	LY6E	-	+
788	REC8	-	+
789	SCUBE2	-	+
790	PRKX	-	+
791	IFI35	-	+
792	PPAP2B	-	+
793	PLS1	-	+
794	TRIB3	-	+
795	POU2AF1	-	+
796	OLFML2A	-	+
797	PTGS1	-	+
798	IFIT2	-	+
799	WNT11	-	+
800	OASL	-	+

801	RP6-213H19.1	-	+
802	DNAJC12	-	+
804	ESRRG	-	+
805	IRF7	-	+
806	SRGN	-	+
807	DENND1A	-	+
808	DKFZP586H2123	-	+
809	WASF3	-	+
810	IGF1R	-	+
811	MT1X	-	+
812	CLUL1	-	+
813	CD36	-	+
814	LRRC48	-	+
815	GPM6A	-	+
816	NRTN	-	+
817	EPS8	-	+
818	C1orf114	-	+
819	GPR126	-	+
820	CHN1	-	+
821	CDR2L	-	+
822	NINJ2	-	+
823	CDKN3	-	+
824	ZNF652	-	+
825	EPHA4	-	+
826	DUSP2	-	+
827	PYCARD	-	+
828	CYTL1	-	+
829	HPN	-	+
830	NEDD9	-	+
831	PTPRM	-	+
833	APOBEC3G	-	+
834	WIT1	-	+
835	HTR2B	-	+
836	RHOBTB3	-	+
837	LTBP1	-	+
838	GNLY	-	+
839	CDC7	-	+
840	HIST3H2A	-	+
841	SV2C	-	+
842	SERPINI1	-	+
843	C1QTNF3	-	+
844	GYPC	-	+
845	MAFF	-	+
846	DEPDC1	-	+
847	PACRG	-	+
848	ADAMTS3	-	+
850	CORO1A	-	+
851	TUBA4A	-	+
852	CXCR4	-	+
853	TNFAIP2	-	+
854	HMGCS2	-	+
855	CDO1	-	+

856	DUSP4	-	+
857	SPAG6	-	+
858	PAMCI	-	+
859	TTK	-	+
860	TGFBI	-	+
861	ID3	-	+
863	SLC24A3	-	+
864	WNT7A	-	+
865	HRASLS3	-	+
866	CENTD1	-	+
867	PRSS16	-	+
869	EMP1	-	+
870	DSE	-	+
872	ITGB8	-	+
873	ZMYND10	-	+
874	STAP2	-	+
875	SLC46A3	-	+
876	GRAMD1C	-	+
877	RIBC2	-	+
878	EGR2	-	+
879	SNX7	-	+
880	FXYD5	-	+
881	HMMR	-	+
882	FCGR2A	-	+
883	CACNA2D2	-	+
885	ERBB3	-	+
887	CHST4	-	+
888	KIF20A	-	+
889	NMNAT2	-	+
890	ABHD3	-	+
891	CCNA2	-	+
892	PTPRC	-	+
893	GHR	-	+
894	BCL11A	-	+
895	C20orf42	-	+
896	FADS1	-	+
897	TP53	-	+
898	ABHD9	-	+
899	GALNT12	-	+
900	P2RY5	-	+
901	NPDC1	-	+
902	HSPB8	-	+
903	PYY	-	+
904	RBP1	-	+
905	CYP2J2	-	+
906	NP	-	+
907	MYC	-	+
908	CXCL6	-	+
909	RAB11FIP1	-	+
910	AMT	-	+
911	ATP7B	-	+
912	SPAG16	-	+

913	GPR143	-	+
914	ECM1	-	+
916	PLAG1	-	+
918	ACP5	-	+
919	TOP2A	-	+
920	SLC7A5	-	+
921	RTN1	-	+
922	DAB2	-	+
923	IRS1	-	+
924	KIF23	-	+
925	RRAD	-	+
926	FZD7	-	+
927	GPRC5C	-	+
928	PIP	-	+
929	EPHB3	-	+
930	NBL1	-	+
931	TNFAIP8	-	+
932	OAS2	-	+
933	PMAIP1	-	+
934	ALPP	-	+
935	PMP22	-	+
936	SPRY1	-	+
937	C11orf75	-	+
938	SLC39A4	-	+
939	AQP3	-	+
940	ITM2A	-	+
941	STK39	-	+
942	LHFP	-	+
943	SLC2A10	-	+
944	TNNI3	-	+
946	DUSP5	-	+
947	NR3C2	-	+
948	IMPA2	-	+
949	TMEM35	-	+
950	RAD51AP1	-	+
951	FGL2	-	+
952	MDK	-	+
953	C4orf19	-	+
954	SPC25	-	+
955	SLIT3	-	+
956	MNDA	-	+
957	HOMER2	-	+
958	CPE	-	+
959	C5orf13	-	+
960	PRKCQ	-	+
962	LY96	-	+
963	OPLAH	-	+
964	TGFB1I1	-	+
965	NDC80	-	+
966	PLCB4	-	+
967	CDCP1	-	+
968	SPOCK2	-	+

969	CASP1	-	+
970	CLDN6	-	+
971	CUGBP2	-	+
972	DEFB4	-	+
973	NEU1	-	+
974	NDUFA4L2	-	+
975	GIMAP4	-	+
976	CLEC5A	-	+
977	LOXL1	-	+
978	COX7A1	-	+
979	LRRC50	-	+
980	GBP2	-	+
981	PSCDBP	-	+
984	CHPT1	-	+
985	LPPR4	-	+
986	GALNT6	-	+
988	DUSP1	-	+
989	SCG2	-	+
990	TPX2	-	+
991	COL16A1	-	+
992	MAGEA12	-	+
993	PTPRG	-	+
994	SLC26A3	-	+
995	CMAS	-	+
996	RRM2	-	+
997	KCNN4	-	+
998	USP18	-	+
