# Supplementary material for: UFFizi: a generic platform for ranking informative features
Source: BMC Bioinformatics. 2010 Jun 3;11:300. doi: 10.1186/1471-2105-11-300 (PMC2893168; doi:10.1186/1471-2105-11-300)
Supplement: Additional file 2 — Enrichment of UFF selected genes for various datasets. Enrichment_tables.pdf: Enrichment of UFF selected genes for the viral infection disease and cancer datasets (using DAVID and ToppGene tools). ToppGene results that appear in DAVID tool were removed. All ToppGene enrichments have Bonferroni < 0.05. [file 1471-2105-11-300-S2.PDF]

Table S1: Enrichment of UFF selected genes for the Melanoma dataset (DAVID and toppgene tools).

Toppgene results that appear in DAVID tool were removed. All Toppgene enrichments have Bonferroni<0.05.

Category (DAVID tool)	Term	Count	% of selected	PValue	Bonferroni	Benjamini
BIOCARTA	h_mhcPathway:Antigen Processing and Presentation	5	2.31%	9.07E-05	2.905E-02	0.029049463
GOTERM_BP_ALL	GO:0007398~ectoderm development	24	11.11%	1.38E-17	7.052E-14	7.05E-14
GOTERM_BP_ALL	GO:0008544~epidermis development	21	9.72%	4.78E-15	2.436E-11	1.22E-11
GOTERM_BP_ALL	GO:0007156~homophilic cell adhesion	21	9.72%	6.74E-15	3.456E-11	1.15E-11
GOTERM_BP_ALL	GO:0009888~tissue development	28	12.96%	3.06E-13	1.560E-09	3.90E-10
GOTERM_BP_ALL	GO:0019882~antigen processing and presentation	14	6.48%	1.03E-11	5.245E-08	1.05E-08
GOTERM_BP_ALL	GO:0030216~keratinocyte differentiation	11	5.09%	6.11E-11	3.118E-07	5.20E-08
GOTERM_BP_ALL	GO:0016337~cell-cell adhesion	23	10.65%	9.58E-11	4.891E-07	6.99E-08
GOTERM_BP_ALL	GO:0006955~immune response	34	15.74%	4.65E-10	2.374E-06	2.97E-07
GOTERM_BP_ALL	GO:0048002~antigen processing and presentation of peptide antigen	10	4.63%	1.20E-09	6.118E-06	6.80E-07
GOTERM_BP_ALL	GO:0002474~antigen processing and presentation of peptide antigen via MHC class I	9	4.17%	5.82E-09	2.969E-05	2.97E-06
GOTERM_BP_ALL	GO:0002376~immune system process	37	17.13%	2.20E-08	1.121E-04	1.02E-05
GOTERM_BP_ALL	GO:0022610~biological adhesion	32	14.81%	1.41E-07	7.188E-04	5.99E-05
GOTERM_BP_ALL	GO:0007155~cell adhesion	32	14.81%	1.41E-07	7.188E-04	5.99E-05
GOTERM_BP_ALL	GO:0018149~peptide cross-linking	7	3.24%	1.72E-07	8.771E-04	6.27E-05
GOTERM_BP_ALL	GO:0048513~organ development	44	20.37%	3.79E-07	1.930E-03	1.29E-04
GOTERM_BP_ALL	GO:0050896~response to stimulus	63	29.17%	1.01E-06	5.154E-03	3.23E-04
GOTERM_BP_ALL	GO:0048731~system development	52	24.07%	1.54E-06	7.845E-03	4.63E-04
GOTERM_CC_ALL	GO:0005882~intermediate filament	19	8.80%	4.03E-16	3.713E-13	1.86E-13
GOTERM_CC_ALL	GO:0045111~intermediate filament cytoskeleton	19	8.80%	4.03E-16	3.713E-13	1.86E-13
GOTERM_CC_ALL	GO:0005576~extracellular region	54	25.00%	1.88E-14	1.578E-11	5.26E-12
GOTERM_CC_ALL	GO:0042611~MHC protein complex	12	5.56%	3.60E-12	3.011E-09	7.53E-10

GOTERM_CC_ALL	GO:0001533~cornified envelope	9	4.17%	1.29E-10	1.080E-07	2.16E-08
GOTERM_CC_ALL	GO:0042612~MHC class I protein complex	9	4.17%	2.39E-09	1.996E-06	3.33E-07
GOTERM_CC_ALL	GO:0044421~extracellular region part	36	16.67%	4.15E-09	3.471E-06	4.96E-07
GOTERM_CC_ALL	GO:0005615~extracellular space	26	12.04%	3.20E-07	2.677E-04	3.35E-05
GOTERM_CC_ALL	GO:0005856~cytoskeleton	35	16.20%	1.70E-06	1.419E-03	1.58E-04
GOTERM_CC_ALL	GO:0044430~cytoskeletal part	24	11.11%	1.53E-05	1.269E-02	0.001275842
GOTERM_CC_ALL	GO:0043228~non-membrane-bound organelle	46	21.30%	2.12E-05	1.755E-02	0.001608265
GOTERM_CC_ALL	GO:0043232~intracellular non-membrane-bound organelle	46	21.30%	2.12E-05	1.755E-02	0.001608265
GOTERM_CC_ALL	GO:0005830~cytosolic ribosome (sensu Eukaryota)	9	4.17%	4.68E-05	3.835E-02	0.003003611
GOTERM_MF_ALL	GO:0005198~structural molecule activity	45	20.83%	3.16E-17	8.804E-14	8.80E-14
GOTERM_MF_ALL	GO:0005509~calcium ion binding	39	18.06%	3.61E-10	1.003E-06	5.02E-07
GOTERM_MF_ALL	GO:0005200~structural constituent of cytoskeleton	14	6.48%	1.12E-09	3.112E-06	1.04E-06
GOTERM_MF_ALL	GO:0003823~antigen binding	11	5.09%	7.48E-09	2.080E-05	5.20E-06
GOTERM_MF_ALL	GO:0005515~protein binding	131	60.65%	5.36E-07	1.491E-03	2.98E-04
INTERPRO	IPR015492:Protocadherin gamma	21	9.72%	2.63E-35	1.464E-31	1.46E-31
INTERPRO	IPR013164:Cadherin, N-terminal	21	9.72%	9.83E-23	5.471E-19	2.74E-19
INTERPRO	IPR003597:Immunoglobulin C1-set	19	8.80%	3.03E-19	1.687E-15	5.62E-16
INTERPRO	IPR003006:Immunoglobulin/major histocompatibility complex motif	19	8.80%	1.87E-18	1.043E-14	2.61E-15
INTERPRO	IPR002126:Cadherin	21	9.72%	5.54E-17	3.082E-13	6.16E-14
INTERPRO	IPR001664:Intermediate filament protein	15	6.94%	2.93E-13	1.633E-09	2.72E-10
INTERPRO	IPR003054:Type II keratin	9	4.17%	2.15E-10	1.195E-06	1.71E-07
INTERPRO	IPR003596:Immunoglobulin V-set, subgroup	11	5.09%	1.75E-09	9.720E-06	1.22E-06
INTERPRO	IPR007110:Immunoglobulin-like	23	10.65%	1.98E-08	1.103E-04	1.23E-05
INTERPRO	IPR013787:S100/CaBP-9k-type, calcium binding, subdomain	8	3.70%	3.32E-08	1.847E-04	1.85E-05
INTERPRO	IPR001751:S100/CaBP-9k-type, calcium binding	8	3.70%	4.80E-08	2.670E-04	2.43E-05
INTERPRO	IPR013783:Immunoglo	22	10.19%	6.49E-07	3.606E-	3.01E-04

	bulin-like fold				03	
KEGG_PATHWAY	hsa01430:Cell Communication	24	11.11%	3.97E-16	1.004E-13	1.00E-13
KEGG_PATHWAY	hsa04612:Antigen processing and presentation	14	6.48%	2.46E-08	5.564E-06	2.78E-06
KEGG_PATHWAY	hsa03010:Ribosome	10	4.63%	8.76E-05	1.960E-02	0.006577284
PIR_SUPERFAMILY	PIRSF002282:cytoskeletal keratin	13	6.02%	4.67E-10	1.351E-06	1.35E-06
PIR_SUPERFAMILY	PIRSF002353:S-100 protein	7	3.24%	1.02E-06	2.949E-03	0.001475668
SMART	SM00407:IGc1	19	8.80%	4.72E-19	2.817E-16	2.82E-16
SMART	SM00112:CA	21	9.72%	8.51E-17	6.628E-14	3.31E-14
SMART	SM00406:IGv	11	5.09%	4.03E-07	2.404E-04	8.01E-05
SP_PIR_KEYWORDS	Direct protein sequencing	110	50.93%	4.66E-34	4.942E-31	4.94E-31
SP_PIR_KEYWORDS	signal	83	38.43%	1.13E-14	1.200E-11	6.00E-12
SP_PIR_KEYWORDS	calcium	39	18.06%	2.13E-13	2.256E-10	7.52E-11
SP_PIR_KEYWORDS	Keratin	14	6.48%	2.39E-13	2.536E-10	6.34E-11
SP_PIR_KEYWORDS	intermediate filament	15	6.94%	2.56E-13	2.718E-10	5.44E-11
SP_PIR_KEYWORDS	heterotetramer	14	6.48%	4.48E-13	4.747E-10	7.91E-11
SP_PIR_KEYWORDS	immunoglobulin	10	4.63%	3.58E-12	3.792E-09	5.42E-10
SP_PIR_KEYWORDS	Immunoglobulin C region	8	3.70%	3.74E-11	3.960E-08	4.95E-09
SP_PIR_KEYWORDS	cell adhesion	27	12.50%	3.99E-11	4.227E-08	4.70E-09
SP_PIR_KEYWORDS	glycoprotein	82	37.96%	7.39E-09	7.829E-06	7.83E-07
SP_PIR_KEYWORDS	Secreted	45	20.83%	9.05E-09	9.593E-06	8.72E-07
SP_PIR_KEYWORDS	pyroglutamic acid	10	4.63%	5.41E-08	5.738E-05	4.78E-06
SP_PIR_KEYWORDS	structural protein	13	6.02%	1.51E-07	1.599E-04	1.23E-05
SP_PIR_KEYWORDS	EF hand	10	4.63%	2.74E-07	2.909E-04	2.08E-05
SP_PIR_KEYWORDS	acetylated amino end	11	5.09%	1.57E-06	1.665E-03	1.11E-04
SP_PIR_KEYWORDS	heterodimer	11	5.09%	3.24E-06	3.431E-03	2.15E-04
SP_PIR_KEYWORDS	duplication	16	7.41%	4.31E-06	4.563E-03	2.69E-04
SP_PIR_KEYWORDS	mhc i	5	2.31%	8.89E-06	9.384E-03	5.24E-04
SP_PIR_KEYWORDS	skin	4	1.85%	1.81E-05	1.902E-02	0.0010101
SP_PIR_KEYWORDS	glycation	5	2.31%	2.27E-05	2.377E-02	0.00120195
SP_PIR_KEYWO	inflammation	6	2.78%	2.58E-05	2.694E-	0.0012997

RDS					02	4
SP_PIR_KEYWORDS	calcium binding	10	4.63%	3.11E-05	3.239E-02	0.001495314
UP_SEQ_FEATURE	domain:Cadherin 6	21	9.72%	1.75E-20	2.780E-16	2.78E-16
UP_SEQ_FEATURE	domain:Cadherin 5	21	9.72%	1.55E-17	2.456E-13	1.23E-13
UP_SEQ_FEATURE	domain:Cadherin 4	21	9.72%	4.39E-17	6.964E-13	1.74E-13
UP_SEQ_FEATURE	domain:Cadherin 3	21	9.72%	4.39E-17	6.964E-13	1.74E-13
UP_SEQ_FEATURE	domain:Cadherin 2	21	9.72%	9.23E-17	1.761E-12	2.94E-13
UP_SEQ_FEATURE	domain:Cadherin 1	21	9.72%	9.23E-17	1.761E-12	2.94E-13
UP_SEQ_FEATURE	region of interest:Linker 12	12	5.56%	7.65E-12	1.213E-07	1.73E-08
UP_SEQ_FEATURE	signal peptide	77	35.65%	8.21E-12	1.302E-07	1.63E-08
UP_SEQ_FEATURE	region of interest:Coil 1B	12	5.56%	1.64E-11	2.603E-07	2.89E-08
UP_SEQ_FEATURE	region of interest:Coil 1A	12	5.56%	1.64E-11	2.603E-07	2.89E-08
UP_SEQ_FEATURE	region of interest:Linker 1	12	5.56%	1.64E-11	2.603E-07	2.89E-08
UP_SEQ_FEATURE	region of interest:Rod	12	5.56%	2.35E-11	3.734E-07	2.67E-08
UP_SEQ_FEATURE	region of interest:Head	12	5.56%	2.35E-11	3.734E-07	2.67E-08
UP_SEQ_FEATURE	region of interest:Tail	12	5.56%	2.35E-11	3.734E-07	2.67E-08
UP_SEQ_FEATURE	region of interest:Coil 2	11	5.09%	4.56E-11	7.241E-07	4.83E-08
UP_SEQ_FEATURE	site:Stutter	7	3.24%	6.57E-08	1.042E-03	6.52E-05
UP_SEQ_FEATURE	domain:Ig-like C1-type	8	3.70%	1.49E-07	2.357E-03	1.39E-04
UP_SEQ_FEATURE	calcium-binding region:2; high affinity	7	3.24%	6.57E-07	1.037E-02	5.79E-04
UP_SEQ_FEATURE	glycosylation site:N-linked (GlcNAc...)	72	33.33%	1.89E-06	2.948E-02	0.001573861

**Category (toppgene tool)**	**Term**	**Hit Count in Query List**	**P-value**	**Source**
GO: Molecular Function	GO:0019838~growth factor binding	9	0.000147	
GO: Molecular Function	GO:0030674~protein binding, bridging	8	0.00099	
GO: Molecular Function	GO:0048407~platelet-derived growth factor binding	4	0.000997	
GO: Molecular Function	GO:0005507~copper ion binding	7	0.001098	
GO: Molecular Function	GO:0032393~MHC class I receptor activity	4	0.003557	
GO: Molecular Function	GO:0004857~enzyme inhibitor activity	11	0.015026	

GO: Molecular Function	GO:0030280~structural constituent of epidermis	3	0.018446	
GO: Molecular Function	GO:0030246~carbohydrate binding	12	0.023065	
GO: Molecular Function	GO:0046870~cadmium ion binding	3	0.035729	
GO: Biological Process	GO:0048730~epidermis morphogenesis	11	0	
GO: Biological Process	GO:0009913~epidermal cell differentiation	9	0.000006	
GO: Biological Process	GO:0031424~keratinization	8	0.000022	
GO: Biological Process	GO:0042438~melanin biosynthetic process	5	0.000031	
GO: Biological Process	GO:0006582~melanin metabolic process	5	0.000057	
GO: Biological Process	GO:0006583~melanin biosynthetic process from tyrosine	4	0.000195	
GO: Biological Process	GO:0008283~cell proliferation	30	0.000631	
GO: Biological Process	GO:0009611~response to wounding	20	0.00066	
GO: Biological Process	GO:0050793~regulation of developmental process	34	0.000859	
GO: Biological Process	GO:0022603~regulation of anatomical structure morphogenesis	17	0.001629	
GO: Biological Process	GO:0048729~tissue morphogenesis	14	0.001635	
GO: Biological Process	GO:0042127~regulation of cell proliferation	23	0.001918	
GO: Biological Process	GO:0032963~collagen metabolic process	6	0.001918	
GO: Biological Process	GO:0002504~antigen processing and presentation of peptide or polysaccharide antigen via MHC class II	5	0.002974	
GO: Biological Process	GO:0044259~multicellular organismal macromolecule metabolic process	6	0.003134	
GO: Biological Process	GO:0051128~regulation of cellular component organization	21	0.005465	
GO: Biological Process	GO:0045104~intermediate filament cytoskeleton organization	5	0.007191	
GO: Biological Process	GO:0030162~regulation of proteolysis	6	0.007443	
GO: Biological Process	GO:0009605~response to external stimulus	24	0.011534	
GO: Biological Process	GO:0006570~tyrosine metabolic process	4	0.012234	

GO: Biological Process	GO:0045109~intermediate filament organization	4	0.012234	
GO: Biological Process	GO:0044236~multicellular organismal metabolic process	6	0.012362	
GO: Biological Process	GO:0045103~intermediate filament-based process	5	0.01267	
GO: Biological Process	GO:0048583~regulation of response to stimulus	15	0.013681	
GO: Biological Process	GO:0042221~response to chemical stimulus	26	0.018245	
GO: Biological Process	GO:0009887~organ morphogenesis	24	0.020054	
GO: Biological Process	GO:0030198~extracellular matrix organization	8	0.021626	
GO: Biological Process	GO:0002478~antigen processing and presentation of exogenous peptide antigen	4	0.036241	
GO: Biological Process	GO:0022604~regulation of cell morphogenesis	13	0.041981	
GO: Biological Process	GO:0006979~response to oxidative stress	8	0.042968	
GO: Biological Process	GO:0002682~regulation of immune system process	14	0.044078	
GO: Biological Process	GO:0045861~negative regulation of proteolysis	4	0.045546	
GO: Biological Process	GO:0032269~negative regulation of cellular protein metabolic process	9	0.048816	
GO: Cellular Component	GO:0045095~keratin filament	9	0.000025	
GO: Cellular Component	GO:0000323~lytic vacuole	12	0.000032	
GO: Cellular Component	GO:0005764~lysosome	12	0.000032	
GO: Cellular Component	GO:0005578~proteinaceous extracellular matrix	15	0.000087	
GO: Cellular Component	GO:0031012~extracellular matrix	15	0.000196	
GO: Cellular Component	GO:0005773~vacuole	12	0.000264	
GO: Cellular Component	GO:0048770~pigment granule	8	0.000419	
GO: Cellular Component	GO:0042470~melanosome	8	0.000419	
GO: Cellular Component	GO:0031988~membrane-bounded vesicle	17	0.001604	
GO: Cellular Component	GO:0022626~cytosolic ribosome	7	0.001651	
GO: Cellular Component	GO:0031982~vesicle	18	0.00202	

GO: Cellular Component	GO:0016023~cytoplasmic membrane-bounded vesicle	16	0.005151	
GO: Cellular Component	GO:0033162~melanosome membrane	3	0.008632	
GO: Cellular Component	GO:0045009~chitosome	3	0.008632	
GO: Cellular Component	GO:0044445~cytosolic part	8	0.015886	
GO: Cellular Component	GO:0005584~collagen type I	2	0.017674	
GO: Cellular Component	GO:0044420~extracellular matrix part	7	0.018971	
GO: Cellular Component	GO:0031410~cytoplasmic vesicle	16	0.019134	
GO: Cellular Component	GO:0005583~fibrillar collagen	3	0.03301	
GO: Cellular Component	GO:0033279~ribosomal subunit	7	0.038915	
GO: Cellular Component	GO:0031093~platelet alpha granule lumen	4	0.047546	
Human Phenotype	HP:0000972~Palmoplantar hyperkeratosis	9	0	
Human Phenotype	HP:0001035~Abnormality of keratinization	13	0.000001	
Human Phenotype	HP:0000962~Hyperkeratosis	11	0.000003	
Human Phenotype	HP:0000951~Skin abnormality	33	0.000006	
Human Phenotype	HP:0001574~Integument abnormality	33	0.000085	
Human Phenotype	HP:0001597~Abnormality of the nails	14	0.000158	
Human Phenotype	HP:0001794~Dystrophic nails	7	0.001272	
Human Phenotype	HP:0001595~Hair abnormality	17	0.001939	
Human Phenotype	HP:0007475~Epidermolytic hyperkeratosis	3	0.007596	
Human Phenotype	HP:0006292~Abnormalities of dental eruption	7	0.020841	
Domain	PF00038~Filament	10	0	Pfam
Domain	PS00226~IF	10	0	PROSITE
Domain	PS00303~S100_CABP	8	0	PROSITE
Domain	PF07654~C1-set	9	0	Pfam
Domain	SM00407~IGc1	9	0	SMART
Domain	PS00290~IG_MHC	9	0.000001	PROSITE
Domain	PD003407~CaBP_S100	7	0.000001	ProDom
Domain	IPR011000~ApoLp_III_like	7	0.000007	InterPro
Domain	PS00018~EF_HAND_1	11	0.000436	PROSITE
Domain	IPR002048~EF_hand_Ca_bd	11	0.001051	InterPro

Domain	PS50222~EF_HAND_2	11	0.00124	PROSITE
Domain	IPR002957~Keratin_I	5	0.0021	InterPro
Domain	IPR000006~Metallthion_1	4	0.003855	InterPro
Domain	PF00131~Metallothio	4	0.003855	Pfam
Domain	IPR003019~Metallthion_	4	0.003855	InterPro
Domain	PS00203~METALLOTHIONEIN_VRT	4	0.003855	PROSITE
Domain	G3DSA:1.10.238.10~EF-Hand_type	11	0.006017	Gene3D
Domain	IPR011992~EF-Hand_type	11	0.006292	InterPro
Domain	PD000050~MHC_I	3	0.011263	ProDom
Domain	PS51162~THYROGLOBULIN_1_2	4	0.012428	PROSITE
Domain	IPR000716~Thyroglobulin_1	4	0.012428	InterPro
Domain	PS00484~THYROGLOBULIN_1_1	4	0.012428	PROSITE
Domain	SM00211~TY	4	0.012428	SMART
Domain	PF00086~Thyroglobulin_1	4	0.012428	Pfam
Pathway	hsa05330~Allograft rejection	7	0.000301	KEGG pathway
Pathway	BREAST_CANCER_ESTROGEN_SIGNALING~BREAST_CANCER_ESTROGEN_SIGNALING	10	0.000501	MSigDB
Pathway	hsa05332~Graft-versus-host disease	7	0.000614	KEGG pathway
Pathway	hsa04940~Type I diabetes mellitus	7	0.000853	KEGG pathway
Pathway	hsa05320~Autoimmune thyroid disease	7	0.003091	KEGG pathway
Pubmed	1286667~Microsequences of 145 proteins recorded in the two-dimensional gel protein database of normal human epidermal keratinocytes.	13	0	
Pubmed	8999895~Direct evidence that involucrin is a major early isopeptide cross-linked component of the keratinocyte cornified cell envelope.	8	0	
Pubmed	16831889~New consensus nomenclature for mammalian keratins.	12	0	
Pubmed	10908733~Solution structure of DFF40 and DFF45 N-terminal domain complex and	6	0	

	mutual chaperone activity of DFF40 and DFF45.			
Pubmed	7759097~Isolation of a YAC clone covering a cluster of nine S100 genes on human chromosome 1q21: rationale for a new nomenclature of the S100 calcium-binding protein family.	7	0	
Pubmed	12427289~HIV-1 gp120 modulates the immunological function and expression of accessory and co-stimulatory molecules of monocyte-derived dendritic cells.	7	0.000001	
Pubmed	8084338~HIV-1 gp41 binding proteins and antibodies to gp41 could inhibit enhancement of human Raji cell MHC class I and II expression by gp41.	7	0.000001	
Pubmed	16130169~Proteomics of human umbilical vein endothelial cells applied to etoposide-induced apoptosis.	10	0.000002	
Pubmed	16091223~HIV accessory proteins and surviving the host cell.	6	0.000005	
Pubmed	17373842~HDM2-binding partners: interaction with translation elongation factor EF1alpha.	6	0.000009	
Pubmed	9751712~HIV-1 tat binds TAFII250 and represses TAFII250-dependent transcription of major histocompatibility class I genes.	5	0.000015	
Pubmed	1409674~Activation of expression of genes coding for extracellular matrix proteins in Tat-producing glioblastoma cells.	4	0.000037	
Pubmed	1468447~Interactions between thrombospondin and the small proteoglycan decorin: interference	4	0.000037	

	with cell attachment.			
Pubmed	12704215~S100 protein subcellular localization during epidermal differentiation and psoriasis.	4	0.000037	
Pubmed	9263011~gp 120s derived from four syncytium-inducing HIV-1 strains induce different patterns of CD4 association with lymphocyte surface molecules.	6	0.000067	
Pubmed	11751963~Native HIV-1 Tat protein targets monocyte-derived dendritic cells and enhances their maturation, function, and antigen-specific T cell responses.	6	0.000094	
Pubmed	8701470~The S100 family of EF-hand calcium-binding proteins: functions and pathology.	5	0.000118	
Pubmed	8341667~Six S100 genes are clustered on human chromosome 1q21: identification of two genes coding for the two previously unreported calcium-binding proteins S100D and S100E.	5	0.000118	
Pubmed	18025061~Matrix metalloproteinase expression in the ascending aorta and aortic valve.	4	0.000182	
Pubmed	12651627~Expression and regulation of tissue inhibitor of metalloproteinase-1 and matrix metalloproteinases by intestinal myofibroblasts in inflammatory bowel disease.	4	0.000182	
Pubmed	18181974~Multimarker real-time reverse transcription-PCR for quantitative detection of melanoma-associated	4	0.000182	

	antigens: a novel possible staging method.			
Pubmed	1713141~Three epidermal and one simple epithelial type II keratin genes map to human chromosome 12.	4	0.000182	
Pubmed	15944607~Genetic analysis of polymorphisms in biologically relevant candidate genes in patients with abdominal aortic aneurysms.	5	0.000324	
Pubmed	8877415~Three regions of HIV-1 gp160 contain clusters of immunodominant CTL epitopes.	4	0.000541	
Pubmed	14595379~The cationic region from HIV tat enhances the cell-surface expression of epitope/MHC class I complexes.	4	0.000541	
Pubmed	18563784~SLC45A2: a novel malignant melanoma-associated gene.	4	0.000541	
Pubmed	10366557~The dileucine-based sorting motif in HIV-1 Nef is not required for down-regulation of class I MHC.	4	0.000541	
Pubmed	10799863~Sequential cleavage by metallopeptidases and proteasomes is involved in processing HIV-1 ENV epitope for endogenous MHC class I antigen presentation.	4	0.000541	
Pubmed	12034345~Expression of matrix metalloproteinases in ovarian endometriomas: immunohistochemical study and enzyme immunoassay.	4	0.000541	
Pubmed	9582271~The SH3 domain-binding surface and an acidic motif in HIV-1 Nef regulate trafficking of class I MHC complexes.	4	0.000541	

Pubmed	8493575~Repression of MHC class I gene promoter activity by two-exon Tat of HIV.	4	0.000541	
Pubmed	10982373~Distinct trafficking pathways mediate Nef-induced and clathrin-dependent major histocompatibility complex class I down-regulation.	4	0.000541	
Pubmed	9120272~Degenerate MHC restriction reveals the contribution of class I MHC molecules in determining the fine specificity of CTL recognition of an immunodominant determinant of HIV-1 gp160 V3 loop.	4	0.000541	
Pubmed	10546855~Enhanced HIV infectivity and changes in GP120 conformation associated with viral incorporation of human leucocyte antigen class I molecules.	4	0.000541	
Pubmed	8612235~Endocytosis of major histocompatibility complex class I molecules is induced by the HIV-1 Nef protein.	4	0.000541	
Pubmed	9373217~The immunosuppressive peptide of HIV-1 gp41 like human type I interferons up-regulates MHC class I expression on H9 and U937 cells.	4	0.000541	
Pubmed	2789433~A single amino acid interchange yields reciprocal CTL specificities for HIV-1 gp160.	4	0.000541	
Pubmed	18588753~Epithelial genes in chronic rhinosinusitis with and without nasal polyps.	4	0.000541	
Pubmed	9840288~On the role of the second coding exon of the HIV-1 Tat protein in virus replication and MHC class I downregulation.	4	0.000541	

Pubmed	8671651~Molecular analysis of presentation by HLA-A2.1 of a promiscuously binding V3 loop peptide from the HIV-envelope protein to human cytotoxic T lymphocytes.	4	0.000541	
Pubmed	11932387~Effect of the V3 loop deletion of envelope glycoprotein on cellular responses and protection against challenge with recombinant vaccinia virus expressing gp160 of primary human immunodeficiency virus type 1 isolates.	4	0.000541	
Pubmed	1602151~Treatment of Haemophilus aphrophilus endocarditis with ciprofloxacin.	9	0.000788	
Pubmed	12665801~Exploring proteomes and analyzing protein processing by mass spectrometric identification of sorted N-terminal peptides.	13	0.000994	
Pubmed	11578695~Deletion of N-terminal myristoylation site of HIV Nef abrogates both MHC-1 and CD4 down-regulation.	4	0.001256	
Pubmed	17564313~Expression of matrix metalloproiteinases (MMPs) and tissue inhibitors of metalloproteinases (TIMPs) in hepatocellular carcinoma tissue, compared with the surrounding non-tumor tissue.	4	0.001256	
Pubmed	9586638~Nef interacts with the mu subunit of clathrin adaptor complexes and reveals a cryptic sorting signal in MHC I molecules.	4	0.001256	
Pubmed	11289809~HIV-1 Nef blocks transport of MHC class I molecules to the cell surface via a	4	0.001256	

	PI 3-kinase-dependent pathway.			
Pubmed	12414957~Direct binding of human immunodeficiency virus type 1 Nef to the major histocompatibility complex class I (MHC-I) cytoplasmic tail disrupts MHC-I trafficking.	4	0.001256	
Pubmed	10684310~Nef-induced major histocompatibility complex class I down-regulation is functionally dissociated from its virion incorporation, enhancement of viral infectivity, and CD4 down-regulation.	4	0.001256	
Pubmed	17589947~Role of matrix metalloproteinase, tissue inhibitor of metalloproteinase and tumor necrosis factor-alpha single nucleotide gene polymorphisms in inflammatory bowel disease.	4	0.001256	
Pubmed	10707087~HIV-1 Nef protein binds to the cellular protein PACS-1 to downregulate class I major histocompatibility complexes.	4	0.001256	
Pubmed	1316930~Serum angiotensin-1 converting enzyme activity processes a human immunodeficiency virus 1 gp160 peptide for presentation by major histocompatibility complex class I molecules.	4	0.001256	
Pubmed	17893005~Multiple-polymorphism associations of 7 matrix metalloproteinase and tissue inhibitor metalloproteinase genes with myocardial infarction and angiographic coronary artery disease.	4	0.001256	

Pubmed	12836198~[The HIV nef and the Kaposi-sarcoma-associated virus K3/K5 proteins: \parasites\of the endocytosis pathway]	5	0.001519	
Pubmed	17081065~Proteomic and bioinformatic characterization of the biogenesis and function of melanosomes.	8	0.001767	
Pubmed	7621073~HIV Tat represses transcription through Sp1-like elements in the basal promoter.	4	0.002498	
Pubmed	12879219~Gene polymorphisms, bone mineral density and bone mineral content in young children: the Iowa Bone Development Study.	4	0.002498	
Pubmed	9052838~The HIV-1 Nef protein acts as a connector with sorting pathways in the Golgi and at the plasma membrane.	4	0.002498	
Pubmed	15078178~HIV/SIV escape from immune surveillance: focus on Nef.	5	0.00282	
Pubmed	12526811~HIV-1 Nef downregulates MHC-I by a PACS-1- and PI3K-regulated ARF6 endocytic pathway.	4	0.00447	
Pubmed	10852826~Interaction of plakophilins with desmoplakin and intermediate filament proteins: an in vitro analysis.	4	0.00447	
Pubmed	7539755~Modulation of CD4 lateral interaction with lymphocyte surface molecules induced by HIV-1 gp120.	5	0.004877	
Pubmed	18468407~Berberine induced down-regulation of matrix metalloproteinase-1, -2 and -9 in human gastric cancer cells (SNU-5) in vitro.	3	0.005025	

Pubmed	17852800~Temporal changes of the plasma levels of cystatin C, beta-trace protein, beta2-microglobulin, urate and creatinine during pregnancy indicate continuous alterations in the renal filtration process.	3	0.005025	
Pubmed	17660250~Constraining specificity in the N-domain of tissue inhibitor of metalloproteinases-1; gelatinase-selective inhibitors.	3	0.005025	
Pubmed	16213873~Difference in expression of matrix metalloproteinase-2 and matrix metalloproteinase-9 in patients with persistent ovarian cysts.	3	0.005025	
Pubmed	7034958~Osteonectin, a bone-specific protein linking mineral to collagen.	3	0.005025	
Pubmed	17157219~HLA-G, -E and -F: allelism, function and evolution.	3	0.005025	
Pubmed	18005116~Keratin and filaggrin expression in keratoacanthoma.	3	0.005025	
Pubmed	17146610~Expression of decorin and collagens I and III in different layers of human skin in vivo: a laser capture microdissection study.	3	0.005025	
Pubmed	18488027~ASIP and TYR pigmentation variants associate with cutaneous melanoma and basal cell carcinoma.	3	0.005025	
Pubmed	18194849~Infrared plus visible light and heat from natural sunlight participate in the expression of MMPs and type I procollagen as well as infiltration of inflammatory cell in human skin in vivo.	3	0.005025	
Pubmed	17200659~Tyrosinase gene family and Vogt-Koyanagi-Harada disease in Japanese	3	0.005025	

	patients.			
Pubmed	12925214~Cytotoxic T lymphocyte reactivity to gp100, MelanA/MART-1, and tyrosinase, in HLA-A2-positive vitiligo patients.	3	0.005025	
Pubmed	16410344~Proteasome blockade exerts an antifibrotic activity by coordinately down-regulating type I collagen and tissue inhibitor of metalloproteinase-1 and up-regulating metalloproteinase-1 production in human dermal fibroblasts.	3	0.005025	
Pubmed	12376362~Gelatinase activities in the airways of premature infants and development of bronchopulmonary dysplasia.	3	0.005025	
Pubmed	8468356~Inhibition of fibronectin binding and fibronectin-mediated cell adhesion to collagen by a peptide from the second type I repeat of thrombospondin.	3	0.005025	
Pubmed	16570139~HLA-E, HLA-F, and HLA-G polymorphism: genomic sequence defines haplotype structure and variation spanning the nonclassical class I genes.	3	0.005025	
Pubmed	17766092~Anemonin is a natural bioactive compound that can regulate tyrosinase-related proteins and mRNA in human melanocytes.	3	0.005025	
Pubmed	12874228~Protein expression and peptide binding suggest unique and interacting functional roles for HLA-E, F, and G in maternal-placental immune recognition.	3	0.005025	

Pubmed	8168508~Recombinant expression and structural and binding properties of alpha 1(VI) and alpha 2(VI) chains of human collagen type VI.	3	0.005025	
Pubmed	12589965~Cathepsin B and H activities and cystatin C concentrations in cerebrospinal fluid from patients with leptomeningeal metastasis.	3	0.005025	
Pubmed	16840178~Matrix metalloproteinase-2, -9, and tissue inhibitor of metalloproteinase-1 in patients with hypertension.	3	0.005025	
Pubmed	7525601~Making a connection: direct binding between keratin intermediate filaments and desmosomal proteins.	3	0.005025	
Pubmed	16280123~Elevated serum levels of proinflammatory cytokines and biomarkers of matrix remodeling in never-treated patients with familial hypercholesterolemia.	3	0.005025	
Pubmed	8636216~Onset of re-epithelialization after skin injury correlates with a reorganization of keratin filaments in wound edge keratinocytes: defining a potential role for keratin 16.	3	0.005025	
Pubmed	8040609~High-molecular-weight forms of tyrosinase and the tyrosinase-related proteins: evidence for a melanogenic complex.	3	0.005025	
Pubmed	11762702~Effects of 17beta-estradiol on the expression of matrix metalloproteinase-1, -2 and tissue inhibitor of metalloproteinase-1 in human osteoblast-like cell cultures.	3	0.005025	

Pubmed	18802702~Association of (-1,607) 1G/2G polymorphism of matrix metalloproteinase-1 gene with knee osteoarthritis in the Turkish population (knee osteoarthritis and MMPs gene polymorphisms).	3	0.005025	
Pubmed	10811646~Nuclear import of insulin-like growth factor-binding protein-3 and -5 is mediated by the importin beta subunit.	3	0.005025	
Pubmed	11886499~Novel and recurrent mutations in the genes encoding keratins K6a, K16 and K17 in 13 cases of pachyonychia congenita.	3	0.005025	
Pubmed	17719747~A spectrum of mutations in keratins K6a, K16 and K17 causing pachyonychia congenita.	3	0.005025	
Pubmed	12527107~Galectin 1 inhibits incorporation of vitronectin and chondroitin sulfate B into the extracellular matrix of human vascular smooth muscle cells.	3	0.005025	
Pubmed	17109104~Matrix metalloproteinases in rectal mucosa, tumour and plasma: response after preoperative irradiation.	3	0.005025	
Pubmed	16359987~Plasma matrix metalloproteinase activity during the menstrual cycle of healthy ovulatory patients who underwent tubal ligation.	3	0.005025	
Pubmed	7543104~Cloning and characterization of multiple human genes and cDNAs encoding highly related type II keratin 6 isoforms.	3	0.005025	
Pubmed	14718574~The human plasma proteome: a nonredundant list developed by	10	0.005834	

	combination of four separate sources.			
Pubmed	18818748~Preterm birth in Caucasians is associated with coagulation and inflammation pathway gene variants.	9	0.006067	
Pubmed	9545232~ER-60, a chaperone with thiol-dependent reductase activity involved in MHC class I assembly.	4	0.007407	
Pubmed	16272310~Functional analysis of HIV type 1 Nef reveals a role for PAK2 as a regulator of cell phenotype and function in the murine dendritic cell line, DC2.4.	4	0.007407	
Pubmed	11707406~Proteomic and functional evidence for a P2X7 receptor signalling complex.	4	0.011572	
Pubmed	2745554~SPARC, a secreted protein associated with cellular proliferation, inhibits cell spreading in vitro and exhibits Ca+2-dependent binding to the extracellular matrix.	4	0.011572	
Pubmed	12539042~HIV-1 Tat reprograms immature dendritic cells to express chemoattractants for activated T cells and macrophages.	6	0.012146	
Pubmed	14760718~Screening for N-glycosylated proteins by liquid chromatography mass spectrometry.	6	0.015817	
Pubmed	14965316~Interactions of HIV-1 proteins gp120 and Nef with cellular partners define a novel allosteric paradigm.	4	0.017258	
Pubmed	15609325~Identification and validation of novel ERBB2 (HER2, NEU) targets including genes involved in angiogenesis.	4	0.017258	
Pubmed	18337837~Lack of functionally active Melan-A(26-35)-specific	3	0.01999	

	T cells in the blood of HLA-A2+ vitiligo patients.			
Pubmed	14983226~Plasma MMP-2 and MMP-9 and their inhibitors TIMP-1 and TIMP-2 during human orthotopic liver transplantation. The effect of aprotinin and the relation to ischemia/reperfusion injury.	3	0.01999	
Pubmed	12880418~Skin expression of metalloproteinases and tissue inhibitor of metalloproteinases in sibling patients with recessive dystrophic epidermolysis and intrafamilial phenotypic variation.	3	0.01999	
Pubmed	17008230~Matrix-metalloproteinases and their inhibitors are elevated in severe sepsis: prognostic value of TIMP-1 in severe sepsis.	3	0.01999	
Pubmed	12479097~[Expression of matrix metalloproteinase-2, 9 and their inhibitor-TIMP 1,2 in human squamous cell carcinoma of uterine cervix]	3	0.01999	
Pubmed	17493602~Relationship between matrix metalloproteinases/tissue inhibitors of matrix metalloproteinases systems and autoantibody patterns in systemic sclerosis.	3	0.01999	
Pubmed	16042227~Serum MMP-9/TIMP-1 and MMP-2/TIMP-2 ratios in multiple sclerosis: relationships with different magnetic resonance imaging measures of disease activity during IFN-beta-1a treatment.	3	0.01999	
Pubmed	16005367~Alterations in peripheral blood levels of TIMP-1, MMP-2, and MMP-9 in	3	0.01999	

	patients with type-2 diabetes.			
Pubmed	1730778~Lymphocyte CD44 binds the COOH-terminal heparin-binding domain of fibronectin.	3	0.01999	
Pubmed	14973177~MMP-2 and MMP-9 and their tissue inhibitors in the plasma of preterm and term neonates.	3	0.01999	
Pubmed	11297531~A role for Hsc70 in regulating nucleocytoplasmic transport of a temperature-sensitive p53 (p53Val-135).	3	0.01999	
Pubmed	18042068~Differential expression of matrix metalloproteinase (MMP)-2, MMP-9 and tissue inhibitor of metalloproteinase (TIMP)-1 and TIMP-2 in non-melanoma skin cancer: implications for tumour progression.	3	0.01999	
Pubmed	15754326~Tissue inhibitor of metalloproteinase 1 is an independent predictor of prognosis in patients with nonsmall cell lung carcinoma who undergo resection with curative intent.	3	0.01999	
Pubmed	12970724~Expression of matrix metalloproteinases (MMP-2 and -9) and their inhibitors (TIMP-1 and -2) in prostate cancer tissue.	3	0.01999	
Pubmed	1544908~Binding of the proteoglycan decorin to collagen type VI.	3	0.01999	
Pubmed	14661256~Matrix metalloproteinases MMP-2, -9 and tissue inhibitors TIMP-1, -2 expression and secretion by primary human osteoblast cells in response to titanium, zirconia, and alumina ceramics.	3	0.01999	

Pubmed	18292822~TGF superfamily and MMP2, MMP9, TIMP1 genes expression in the endometrium of women with impaired reproduction.	3	0.01999	
Pubmed	17205957~Specific changes in plasma concentrations of matrix metalloproteinase-2 and -9, TIMP-1 and TGF-beta1 in patients with distinct types of primary glomerulonephritis.	3	0.01999	
Pubmed	9344465~Interaction of matrix metalloproteinases-2 and -9 with pregnancy zone protein and alpha2-macroglobulin.	3	0.01999	
Pubmed	17786346~Expression of metalloproteinases and their tissue inhibitors in squamous cell laryngeal carcinoma.	3	0.01999	
Pubmed	18323784~Akt blockade downregulates collagen and upregulates MMP1 in human dermal fibroblasts.	3	0.01999	
Pubmed	15955221~Association of functional gene polymorphisms of matrix metalloproteinase (MMP)-1, MMP-3 and MMP-9 with the progression of chronic liver disease.	3	0.01999	
Pubmed	18217401~Matrix metalloproteinases and their tissue inhibitors in children with chronic hepatitis B treated with lamivudine.	3	0.01999	
Pubmed	6203523~The place of human gamma-trace (cystatin C) amongst the cysteine proteinase inhibitors.	3	0.01999	
Pubmed	8119276~Induction by zinc of specific metallothionein isoforms in human monocytes.	3	0.01999	

Pubmed	15248826~Intrafollicular expression of matrix metalloproteinases and their inhibitors in normally ovulating women compared with patients undergoing in vitro fertilization treatment.	3	0.01999	
Pubmed	18477480~Comparative assessment of matrix metalloproteinase (MMP)-2 and MMP-9, and their inhibitors, tissue inhibitors of metalloproteinase (TIMP)-1 and TIMP-2 in preeclampsia and gestational hypertension.	3	0.01999	
Pubmed	10837826~S100 proteins in Corpora amylacea from normal human brain.	3	0.01999	
Pubmed	16739355~Matrix metalloproteinase (MMP) -1, -2, -3 and -9 promoter polymorphisms in colorectal cancer.	3	0.01999	
Pubmed	12034705~The transforming growth factor-beta-inducible matrix protein (beta)ig-h3 interacts with fibronectin.	3	0.01999	
Pubmed	15557756~Matrix metalloproteinases 2 and 9 and their tissue inhibitors in low malignant potential ovarian tumors.	3	0.01999	
Pubmed	7523391~Matrix metalloproteinases degrade insulin-like growth factor-binding protein-3 in dermal fibroblast cultures.	3	0.01999	
Pubmed	16772717~Serum levels of matrix metalloproteinases-2 and -9 and their tissue inhibitors in inflammatory neuromuscular disorders.	3	0.01999	

Pubmed	14503883~Grafting of features of cystatins C or B into the N-terminal region or second binding loop of cystatin A (stefin A) substantially enhances inhibition of cysteine proteinases.	3	0.01999	
Pubmed	17350093~Expression of matrix metalloproteinase (MMP) and tissue inhibitor of MMP (TIMP) genes in blasts of infant acute lymphoblastic leukemia with organ involvement.	3	0.01999	
Pubmed	17071711~Gelatinases and their tissue inhibitors during human ovulation: increased expression of tissue inhibitor of matrix metalloproteinase-1.	3	0.01999	
Pubmed	17226791~Regulation of matrix metalloproteinases and tissue inhibitors of matrix metalloproteinases by Porphyromonas gingivalis in an engineered human oral mucosa model.	3	0.01999	
Pubmed	7913356~HIV-1 gp41 enhances major histocompatibility complex class I and ICAM-1 expression on H9 and U937 cells.	4	0.024785	
Pubmed	15607035~Systematic identification of hepatocellular proteins interacting with NS5A of the hepatitis C virus.	4	0.024785	
Pubmed	12643545~Proteomic analysis of early melanosomes: identification of novel melanosomal proteins.	5	0.027129	
Pubmed	16289162~The identification of myocilin-associated proteins in the human trabecular meshwork.	5	0.032361	
Pubmed	15189156~The molecular mechanics of eukaryotic translation.	7	0.048033	

Pubmed	9675033~Decorin core protein fragment Leu155-Val260 interacts with TGF-beta but does not compete for decorin binding to type I collagen.	3	0.049702	
Pubmed	16650578~Alpha2-macroglobulin, lipoprotein receptor-related protein and lipoprotein receptor-associated protein and the genetic risk for developing Alzheimer's disease.	3	0.049702	
Pubmed	11598182~Activation of matrix-metalloproteinase-2 and membrane-type-1-matrix-metalloproteinase in endothelial cells and induction of vascular permeability in vivo by human immunodeficiency virus-1 Tat protein and basic fibroblast growth factor.	3	0.049702	
Pubmed	15365990~Stability related bias in residues replacing glycines within the collagen triple helix (Gly-Xaa-Yaa) in inherited connective tissue disorders.	3	0.049702	
Pubmed	17554369~TNF-alpha suppresses alpha-smooth muscle actin expression in human dermal fibroblasts: an implication for abnormal wound healing.	3	0.049702	
Pubmed	11973338~The epithelial mitogen keratinocyte growth factor binds to collagens via the consensus sequence glycine-proline-hydroxyproline.	3	0.049702	
Pubmed	18364388~A novel translation re-initiation mechanism for the p63 gene revealed by amino-terminal truncating mutations in Rapp-Hodgkin/Hay-	3	0.049702	

	Wells-like syndromes.			
Pubmed	18035073~Matrix metalloproteinase circulating levels, genetic polymorphisms, and susceptibility to acute myocardial infarction among patients with coronary artery disease.	3	0.049702	
Pubmed	11359786~Pro-collagenase-1 (matrix metalloproteinase-1) binds the alpha(2)beta(1) integrin upon release from keratinocytes migrating on type I collagen.	3	0.049702	
Pubmed	18676742~Gene expression profiling of papillary thyroid carcinoma identifies transcripts correlated with BRAF mutational status and lymph node metastasis.	3	0.049702	
Disease	D009914~Oral Submucous Fibrosis	7	0.000004	CTD
Disease	D053549~Pachyonychia Congenita	4	0.00007	CTD
Disease	D002294~Carcinoma, Squamous Cell	7	0.000099	CTD
Disease	D012878~Skin Neoplasms	6	0.000747	CTD

Table S2: Enrichment of UFF selected genes for the HIV dataset (DAVID and toppgene tools).

Category (DAVID tool)	Term	Count	%	P-Value	Bonferroni	Benjamini
BIOCARTA	h_nkcellsPathway:Ras-Independent pathway in NK cell-mediated cytotoxicity	6	3.53%	1.24E-04	3.92E-02	3.92E-02
GOTERM_BP_ALL	GO:0002376~immune system process	49	28.82%	2.20E-21	1.16E-17	1.16E-17
GOTERM_BP_ALL	GO:0006955~immune response	42	24.71%	5.28E-19	2.77E-15	1.39E-15
GOTERM_BP_ALL	GO:0050896~response to stimulus	59	34.71%	2.12E-10	1.11E-06	3.71E-07
GOTERM_BP_ALL	GO:0019882~antigen processing and presentation	16	9.41%	8.52E-09	4.47E-05	1.12E-05
GOTERM_BP_ALL	GO:0002474~antigen processing and presentation of peptide antigen via MHC class I	9	5.29%	1.06E-08	5.55E-05	1.11E-05

GOTERM_BP_ALL	GO:0048002~antigen processing and presentation of peptide antigen	9	5.29%	2.27E-08	1.19E-04	1.99E-05
GOTERM_BP_ALL	GO:0006968~cellular defense response	9	5.29%	2.36E-07	1.24E-03	1.77E-04
GOTERM_BP_ALL	GO:0009607~response to biotic stimulus	15	8.82%	4.58E-07	2.40E-03	3.00E-04
GOTERM_BP_ALL	GO:0006952~defense response	19	11.18%	1.94E-06	1.02E-02	1.13E-03
GOTERM_BP_ALL	GO:0006915~apoptosis	22	12.94%	2.87E-06	1.50E-02	1.51E-03
GOTERM_BP_ALL	GO:0012501~programmed cell death	22	12.94%	3.30E-06	1.72E-02	1.57E-03
GOTERM_BP_ALL	GO:0051704~multi-organism process	14	8.24%	3.50E-06	1.82E-02	1.53E-03
GOTERM_BP_ALL	GO:0016265~death	22	12.94%	7.48E-06	3.86E-02	3.02E-03
GOTERM_BP_ALL	GO:0008219~cell death	22	12.94%	7.48E-06	3.86E-02	3.02E-03
GOTERM_BP_ALL	GO:0051707~response to other organism	11	6.47%	8.60E-06	4.42E-02	3.01E-03
GOTERM_CC_ALL	GO:0043234~protein complex	45	26.47%	1.68E-09	1.45E-06	1.45E-06
GOTERM_CC_ALL	GO:0032991~macromolecular complex	50	29.41%	4.21E-09	3.66E-06	1.83E-06
GOTERM_CC_ALL	GO:0042612~MHC class I protein complex	9	5.29%	7.14E-08	6.20E-05	2.07E-05
GOTERM_CC_ALL	GO:0044445~cytosolic part	11	6.47%	3.13E-07	2.72E-04	6.79E-05
GOTERM_CC_ALL	GO:0042611~MHC protein complex	13	7.65%	7.15E-07	6.20E-04	1.24E-04
GOTERM_CC_ALL	GO:0044459~plasma membrane part	36	21.18%	1.21E-05	1.05E-02	1.75E-03
GOTERM_CC_ALL	GO:0005829~cytosol	16	9.41%	2.57E-05	2.20E-02	3.18E-03
GOTERM_MF_ALL	GO:0005515~protein binding	85	50.00%	1.03E-07	2.95E-04	2.95E-04
GOTERM_MF_ALL	GO:0032393~MHC class I receptor activity	6	3.53%	4.17E-07	1.20E-03	6.00E-04
GOTERM_MF_ALL	GO:0032395~MHC class II receptor activity	5	2.94%	6.26E-07	1.80E-03	6.01E-04
INTERPRO	IPR003597:Immunoglobulin C1-set	18	10.59%	1.37E-14	8.08E-11	8.08E-11
INTERPRO	IPR003006:Immunoglobulin/major histocompatibility complex motif	17	10.00%	6.27E-13	3.71E-09	1.85E-09
INTERPRO	IPR007110:Immunoglobulin-like	20	11.76%	1.30E-06	7.68E-03	2.57E-03
INTERPRO	IPR013783:Immunoglobulin-like fold	21	12.35%	1.98E-06	1.16E-02	2.92E-03
KEGG_PATHWAY	hsa04612:Antigen processing and presentation	15	8.82%	1.65E-12	3.31E-10	3.31E-10
KEGG_PATHWAY	hsa04940:Type I diabetes mellitus	10	5.88%	1.68E-09	3.38E-07	1.69E-07
KEGG_PATHWAY	hsa04514:Cell adhesion molecules (CAMs)	14	8.24%	1.26E-08	2.52E-06	8.41E-07
KEGG_PATHWAY	hsa04650:Natural killer cell mediated cytotoxicity	10	5.88%	6.14E-05	1.23E-02	3.08E-03
SMART	SM00407:IGc1	18	10.59%	4.00E-14	2.43E-11	2.43E-11
SP_PIR_KEYWORDS	Direct protein sequencing	64	37.65%	9.47E-20	1.01E-16	1.01E-16

SP_PIR_KEYWORDS	heterodimer	16	9.41%	1.73E-15	1.89E-12	9.47E-13
SP_PIR_KEYWORDS	acetylation	23	13.53%	1.01E-09	1.08E-06	3.59E-07
SP_PIR_KEYWORDS	immune response	14	8.24%	1.20E-09	1.28E-06	3.19E-07
SP_PIR_KEYWORDS	transmembrane protein	21	12.35%	8.95E-08	9.54E-05	1.91E-05
SP_PIR_KEYWORDS	T-cell	7	4.12%	4.83E-07	5.15E-04	8.58E-05
SP_PIR_KEYWORDS	homotetramer	6	3.53%	1.16E-06	1.23E-03	1.76E-04
SP_PIR_KEYWORDS	mhc i	6	3.53%	4.92E-06	5.24E-03	6.56E-04
SP_PIR_KEYWORDS	heterotetramer	9	5.29%	1.06E-05	1.13E-02	1.26E-03
SP_PIR_KEYWORDS	immunoglobulin	7	4.12%	1.24E-05	1.31E-02	1.32E-03
UP_SEQ_FEATURE	domain:Ig-like C1-type	10	5.88%	1.41E-09	2.66E-05	2.66E-05
UP_SEQ_FEATURE	region of interest:Connecting peptide	9	5.29%	7.85E-08	1.48E-03	7.40E-04
UP_SEQ_FEATURE	region of interest:Alpha-2	7	4.12%	2.04E-06	3.77E-02	1.27E-02
UP_SEQ_FEATURE	region of interest:Alpha-1	7	4.12%	2.04E-06	3.77E-02	1.27E-02

**Category (toppgene tool)**	**Term**	**Hit Count in Query List**	**P-value**	**Source**
GO: Molecular Function	GO:0005344~oxygen transporter activity	3	0.03905	
GO: Biological Process	GO:0050793~regulation of developmental process	30	0.000581	
GO: Biological Process	GO:0002504~antigen processing and presentation of peptide or polysaccharide antigen via MHC class II	5	0.001136	
GO: Biological Process	GO:0042221~response to chemical stimulus	24	0.004002	
GO: Biological Process	GO:0010941~regulation of cell death	20	0.00524	
GO: Biological Process	GO:0051094~positive regulation of developmental process	17	0.009763	
GO: Biological Process	GO:0030097~hemopoiesis	13	0.011153	
GO: Biological Process	GO:0002682~regulation of immune system process	13	0.020972	
GO: Biological Process	GO:0001775~cell activation	13	0.034007	
GO: Biological Process	GO:0019884~antigen processing and presentation of exogenous antigen	4	0.048097	
GO: Cellular Component	GO:0042613~MHC class II protein complex	4	0.000381	

GO: Cellular Component	GO:0009897~external side of plasma membrane	9	0.000403	
GO: Cellular Component	GO:0009986~cell surface	11	0.004902	
GO: Cellular Component	GO:0022626~cytosolic ribosome	5	0.041538	
Mouse Phenotype	MP:0008081~abnormal single-positive T cell number	14	0.001619	
Mouse Phenotype	MP:0008079~decreased CD8-positive T cell number	10	0.002543	
Mouse Phenotype	MP:0002435~abnormal effector T cell morphology	15	0.003525	
Mouse Phenotype	MP:0005010~abnormal CD8-positive T cell morphology	12	0.003627	
Mouse Phenotype	MP:0008083~decreased single-positive T cell number	12	0.003627	
Mouse Phenotype	MP:0008075~decreased CD4-positive T cell number	10	0.004381	
Mouse Phenotype	MP:0005069~abnormal NK cell physiology	8	0.004876	
Mouse Phenotype	MP:0008077~abnormal CD8-positive T cell number	11	0.006638	
Mouse Phenotype	MP:0008073~abnormal CD4-positive T cell number	11	0.008471	
Mouse Phenotype	MP:0005015~increased T cell number	13	0.010511	
Mouse Phenotype	MP:0003945~abnormal lymphocyte physiology	22	0.018543	
Mouse Phenotype	MP:0005078~abnormal cytotoxic T cell physiology	7	0.023661	
Mouse Phenotype	MP:0005013~increased lymphocyte cell number	15	0.033895	
Mouse Phenotype	MP:0002429~abnormal blood cell morphology/development	32	0.047975	
Mouse Phenotype	MP:0008344~abnormal mature gamma-delta T cell morphology	4	0.05	
Pathway	hsa05332~Graft-versus-host disease	11	0	KEGG pathway
Pathway	hsa04612~Antigen processing and presentation	14	0	KEGG pathway
Pathway	hsa05330~Allograft rejection	10	0	KEGG pathway
Pathway	hsa04940~Type I diabetes mellitus	10	0	KEGG pathway
Pathway	hsa05320~Autoimmune thyroid disease	10	0	KEGG pathway
Pathway	hsa04514~Cell adhesion molecules (CAMs)	14	0	KEGG pathway
Pathway	P00053~T cell activation	9	0.000204	PantherDB
Pathway	CTLPATHWAY~CTLPATHWAY	5	0.00034	MSigDB

Pathway	NKCELLSPATHWAY~NKCELLSPATHWAY	5	0.001672	MSigDB
Pathway	SETPATHWAY~SETPATHWAY	4	0.003226	MSigDB
Pathway	hsa04650~Natural killer cell mediated cytotoxicity	10	0.003412	KEGG pathway
Pathway	P00031~Inflammation mediated by chemokine and cytokine signaling	11	0.010449	PantherDB
Pathway	MTA3PATHWAY~MTA3PATHWAY	4	0.016961	MSigDB
Pathway	hsa04660~T cell receptor signaling pathway	8	0.027019	KEGG pathway
Pubmed	12427289~HIV-1 gp120 modulates the immunological function and expression of accessory and co-stimulatory molecules of monocyte-derived dendritic cells.	8	0	
Pubmed	8084338~HIV-1 gp41 binding proteins and antibodies to gp41 could inhibit enhancement of human Raji cell MHC class I and II expression by gp41.	8	0	
Pubmed	10809759~Classical and nonclassical class I major histocompatibility complex molecules exhibit subtle conformational differences that affect binding to CD8alphaalpha.	6	0	
Pubmed	11751963~Native HIV-1 Tat protein targets monocyte-derived dendritic cells and enhances their maturation, function, and antigen-specific T cell responses.	7	0	
Pubmed	9751712~HIV-1 tat binds TAFII250 and represses TAFII250-dependent transcription of major histocompatibility class I genes.	5	0.000003	
Pubmed	17703412~Genetic susceptibility to respiratory syncytial virus bronchiolitis is predominantly associated with innate immune genes.	12	0.000043	
Pubmed	14965316~Interactions of HIV-1 proteins gp120 and Nef with cellular partners define a novel allosteric paradigm.	5	0.000048	
Pubmed	16091223~HIV accessory proteins and surviving the host cell.	5	0.000119	

Pubmed	9582271~The SH3 domain-binding surface and an acidic motif in HIV-1 Nef regulate trafficking of class I MHC complexes.	4	0.0001 57	
Pubmed	8877415~Three regions of HIV-1 gp160 contain clusters of immunodominant CTL epitopes.	4	0.0001 57	
Pubmed	8493575~Repression of MHC class I gene promoter activity by two-exon Tat of HIV.	4	0.0001 57	
Pubmed	10982373~Distinct trafficking pathways mediate Nef-induced and clathrin-dependent major histocompatibility complex class I down-regulation.	4	0.0001 57	
Pubmed	9120272~Degenerate MHC restriction reveals the contribution of class I MHC molecules in determining the fine specificity of CTL recognition of an immunodominant determinant of HIV-1 gp160 V3 loop.	4	0.0001 57	
Pubmed	14595379~The cationic region from HIV tat enhances the cell-surface expression of epitope/MHC class I complexes.	4	0.0001 57	
Pubmed	10546855~Enhanced HIV infectivity and changes in GP120 conformation associated with viral incorporation of human leucocyte antigen class I molecules.	4	0.0001 57	
Pubmed	8612235~Endocytosis of major histocompatibility complex class I molecules is induced by the HIV-1 Nef protein.	4	0.0001 57	
Pubmed	9373217~The immunosuppressive peptide of HIV-1 gp41 like human type I interferons up-regulates MHC class I expression on H9 and U937 cells.	4	0.0001 57	
Pubmed	2789433~A single amino acid interchange yields reciprocal CTL specificities for HIV-1 gp160.	4	0.0001 57	
Pubmed	9840288~On the role of the second coding exon of the HIV-1 Tat protein in virus replication and MHC class I	4	0.0001 57	

	downregulation.			
Pubmed	10366557~The dileucine-based sorting motif in HIV-1 Nef is not required for down-regulation of class I MHC.	4	0.0001 57	
Pubmed	10799863~Sequential cleavage by metallopeptidases and proteasomes is involved in processing HIV-1 ENV epitope for endogenous MHC class I antigen presentation.	4	0.0001 57	
Pubmed	8671651~Molecular analysis of presentation by HLA-A2.1 of a promiscuously binding V3 loop peptide from the HIV-envelope protein to human cytotoxic T lymphocytes.	4	0.0001 57	
Pubmed	11932387~Effect of the V3 loop deletion of envelope glycoprotein on cellular responses and protection against challenge with recombinant vaccinia virus expressing gp160 of primary human immunodeficiency virus type 1 isolates.	4	0.0001 57	
Pubmed	15638726~Nef: "necessary and enforcing factor" in HIV infection.	6	0.0002 38	
Pubmed	12836198~[The HIV nef and the Kaposi-sarcoma-associated virus K3/K5 proteins: \parasites\of the endocytosis pathway]	5	0.0003 64	
Pubmed	11289809~HIV-1 Nef blocks transport of MHC class I molecules to the cell surface via a PI 3-kinase-dependent pathway.	4	0.0003 64	
Pubmed	12414957~Direct binding of human immunodeficiency virus type 1 Nef to the major histocompatibility complex class I (MHC-I) cytoplasmic tail disrupts MHC-I trafficking.	4	0.0003 64	
Pubmed	12445315~The association of specific HLA class I and II alleles with type 1 diabetes among Filipinos.	4	0.0003 64	

Pubmed	11578695~Deletion of N-terminal myristoylation site of HIV Nef abrogates both MHC-1 and CD4 down-regulation.	4	0.000364	
Pubmed	10684310~Nef-induced major histocompatibility complex class I down-regulation is functionally dissociated from its virion incorporation, enhancement of viral infectivity, and CD4 down-regulation.	4	0.000364	
Pubmed	10707087~HIV-1 Nef protein binds to the cellular protein PACS-1 to downregulate class I major histocompatibility complexes.	4	0.000364	
Pubmed	9586638~Nef interacts with the mu subunit of clathrin adaptor complexes and reveals a cryptic sorting signal in MHC I molecules.	4	0.000364	
Pubmed	1316930~Serum angiotensin-1 converting enzyme activity processes a human immunodeficiency virus 1 gp160 peptide for presentation by major histocompatibility complex class I molecules.	4	0.000364	
Pubmed	15078178~HIV/SIV escape from immune surveillance: focus on Nef.	5	0.000677	
Pubmed	9052838~The HIV-1 Nef protein acts as a connector with sorting pathways in the Golgi and at the plasma membrane.	4	0.000724	
Pubmed	17785583~High-resolution donor-recipient HLA matching contributes to the success of unrelated donor marrow transplantation.	4	0.000724	
Pubmed	11181188~[Two loci HLA haplotypes in celiac children. Linkage imbalance and haplotype frequencies. Comparative study with a control population]	4	0.000724	
Pubmed	7621073~HIV Tat represses transcription through Sp1-like elements in the basal promoter.	4	0.000724	
Pubmed	12734410~HIV-1 Nef control of cell signalling molecules: multiple strategies to promote virus replication.	5	0.000898	

Pubmed	7539755~Modulation of CD4 lateral interaction with lymphocyte surface molecules induced by HIV-1 gp120.	5	0.0011 73	
Pubmed	12526811~HIV-1 Nef downregulates MHC-I by a PACS-1- and PI3K-regulated ARF6 endocytic pathway.	4	0.0012 97	
Pubmed	3262682~Characterization of three serine esterases isolated from human IL-2 activated killer cells.	3	0.0017 7	
Pubmed	11397794~Inhibition of AP-1 by the glucocorticoid-inducible protein GILZ.	3	0.0017 7	
Pubmed	15998831~Quiescent and activated mouse granulocytes do not express granzyme A and B or perforin: similarities or differences with human polymorphonuclear leukocytes?	3	0.0017 7	
Pubmed	8649448~Selective association of a 22-38 kDa glycoprotein with MHC class II DP antigen on activated human lymphocytes at the plasma membrane.	3	0.0017 7	
Pubmed	16354571~Roles of HIV-1 auxiliary proteins in viral pathogenesis and host-pathogen interactions.	5	0.0019 21	
Pubmed	9545232~ER-60, a chaperone with thiol-dependent reductase activity involved in MHC class I assembly.	4	0.0021 52	
Pubmed	12956878~Roles of HLA-B, HLA-C and HLA-DPA1 incompatibilities in the outcome of unrelated stem-cell transplantation.	4	0.0021 52	
Pubmed	16272310~Functional analysis of HIV type 1 Nef reveals a role for PAK2 as a regulator of cell phenotype and function in the murine dendritic cell line, DC2.4.	4	0.0021 52	
Pubmed	11827988~Adapters in lymphocyte signaling.	5	0.0024 15	
Pubmed	16702430~Rapid evolution of major histocompatibility complex class I genes in primates generates new disease alleles in humans via hitchhiking diversity.	5	0.0065 84	

Pubmed	17317585~Effects of HLA allele and killer immunoglobulin-like receptor ligand matching on clinical outcome in leukemia patients undergoing transplantation with T-cell-replete marrow from an unrelated donor.	3	0.0070 46	
Pubmed	11861287~Soluble HLA class I molecules induce natural killer cell apoptosis through the engagement of CD8: evidence for a negative regulation exerted by members of the inhibitory receptor superfamily.	3	0.0070 46	
Pubmed	14623968~Translationally controlled tumor protein acts as a guanine nucleotide dissociation inhibitor on the translation elongation factor eEF1A.	3	0.0070 46	
Pubmed	12594841~Soluble HLA-A,-B,-C and -G molecules induce apoptosis in T and NK CD8+ cells and inhibit cytotoxic T cell activity through CD8 ligation.	3	0.0070 46	
Pubmed	16459714~Intrathecal synthesis of soluble HLA-G and HLA-I molecules are reciprocally associated to clinical and MRI activity in patients with multiple sclerosis.	3	0.0070 46	
Pubmed	11513152~Structural basis of MHC class I recognition by natural killer cell receptors.	3	0.0070 46	
Pubmed	12419805~Exogenous Nef protein activates NF-kappa B, AP-1, and c-Jun N-terminal kinase and stimulates HIV transcription in promonocytic cells. Role in AIDS pathogenesis.	3	0.0070 46	
Pubmed	11053448~Identification of SWI.SNF complex subunit BAF60a as a determinant of the transactivation potential of Fos/Jun dimers.	3	0.0070 46	
Pubmed	16228294~The integrin alpha6beta1 modulation of PI3K and Cdc42 activities induces dynamic filopodium formation in human platelets.	3	0.0070 46	

Pubmed	17681951~Heterodimerization with Jun family members regulates c-Fos nucleocytoplasmic traffic.	3	0.007046	
Pubmed	7913356~HIV-1 gp41 enhances major histocompatibility complex class I and ICAM-1 expression on H9 and U937 cells.	4	0.007224	
Pubmed	7512597~HLA class II antigens and the HIV envelope glycoprotein gp120 bind to the same face of CD4.	4	0.010066	
Pubmed	1869305~Interaction of CD4 with HLA class II antigens and HIV gp120.	4	0.010066	
Pubmed	8168144~Envelope glycoproteins of HIV-1 interfere with T-cell-dependent B cell differentiation: role of CD4-MHC class II interaction in the effector phase of T cell help.	4	0.013661	
Pubmed	3489470~AIDS and related syndromes as a viral-induced autoimmune disease of the immune system: an anti-MHC II disorder. Therapeutic implications.	4	0.013661	
Pubmed	10729169~Efficient incorporation of HLA class II onto human immunodeficiency virus type 1 requires envelope glycoprotein packaging.	4	0.013661	
Pubmed	1967269~HIV-gp120 can block CD4-class II MHC-mediated adhesion.	4	0.013661	
Pubmed	7602119~Endocytosis of endogenously synthesized HIV-1 envelope protein. Mechanism and role in processing for association with class II MHC.	4	0.013661	
Pubmed	9079699~The enhanced immune response to the HIV gp160/LAMP chimeric gene product targeted to the lysosome membrane protein trafficking pathway.	4	0.013661	
Pubmed	8376762~HIV-1 envelope protein is expressed on the surface of infected cells before its processing and presentation to class II-restricted T lymphocytes.	4	0.013661	

Pubmed	11909973~HMG2 interacts with the nucleosome assembly protein SET and is a target of the cytotoxic T-lymphocyte protease granzyme A.	3	0.0175 37	
Pubmed	15507668~Constitutive expression of the AP-1 transcription factors c-jun, junD, junB, and c-fos and the marginal zone B-cell transcription factor Notch2 in splenic marginal zone lymphoma.	3	0.0175 37	
Pubmed	17495958~Biphasic regulation of AP-1 subunits during human epidermal wound healing.	3	0.0175 37	
Pubmed	11983170~Visualization of interactions among bZIP and Rel family proteins in living cells using bimolecular fluorescence complementation.	3	0.0175 37	
Pubmed	12899833~Sequence variability analysis of human class I and class II MHC molecules: functional and structural correlates of amino acid polymorphisms.	3	0.0175 37	
Pubmed	18486765~Type 1 diabetes risk for human leukocyte antigen (HLA)-DR3 haplotypes depends on genotypic context: association of DPB1 and HLA class I loci among DR3- and DR4-matched Italian patients and controls.	3	0.0175 37	
Pubmed	16001969~Human immunodeficiency virus type 1 envelope glycoprotein 120 induces cyclooxygenase-2 expression in neuroblastoma cells through a nuclear factor-kappaB and activating protein-1 mediated mechanism.	3	0.0175 37	
Pubmed	8769474~Roles for calreticulin and a novel glycoprotein, tapasin, in the interaction of MHC class I molecules with TAP.	3	0.0175 37	
Pubmed	2543930~Identification of human CD4 residues affecting class II MHC versus HIV-1 gp120 binding.	4	0.0181 29	

Pubmed	1978941~Identification and structural analysis of residues in the V1 region of CD4 involved in interaction with human immunodeficiency virus envelope glycoprotein gp120 and class II major histocompatibility complex molecules.	4	0.0181 29	
Pubmed	2846691~Inhibition of CD4+ T cell function by the HIV envelope protein, gp120.	4	0.0181 29	
Pubmed	10661406~Tat competes with CIITA for the binding to P-TEFb and blocks the expression of MHC class II genes in HIV infection.	4	0.0301 94	
Pubmed	11118314~Reciprocal modulation of transcriptional activities between HIV-1 Tat and MHC class II transactivator CIITA.	4	0.0301 94	
Pubmed	16019679~Analysis of HLA class I and class II gene polymorphisms in Japanese patients with human T-cell lymphotropic virus type 1-associated uveitis.	3	0.0349 18	
Pubmed	15191952~Impact of HLA class I and class II high-resolution matching on outcomes of unrelated donor bone marrow transplantation: HLA-C mismatching is associated with a strong adverse effect on transplantation outcome.	3	0.0349 18	
Pubmed	12853576~Human inhibitory receptors Ig-like transcript 2 (ILT2) and ILT4 compete with CD8 for MHC class I binding and bind preferentially to HLA-G.	3	0.0349 18	
Pubmed	18976432~HLA-A, B, Cw, DRB1, DQB1 and DPB1 alleles and haplotypes in the genetically homogenous Irish population.	3	0.0349 18	
Pubmed	18976440~The clinical implications of HLA mismatches in unrelated donor haematopoietic cell transplantation.	3	0.0349 18	
Pubmed	18958335~Inhibitor-Immunology-Study. Different HLA-types seem to be involved in the inhibitor development in haemophilia A.	3	0.0349 18	

Pubmed	16327313~Inhibitory effect of HIV-1 Tat protein on the sodium-D-glucose symporter of human intestinal epithelial cells.	3	0.0349 18	
Pubmed	9872330~Nrf2 and Nrf1 in association with Jun proteins regulate antioxidant response element-mediated expression and coordinated induction of genes encoding detoxifying enzymes.	3	0.0349 18	
Pubmed	17554059~High-risk HLA allele mismatch combinations responsible for severe acute graft-versus-host disease and implication for its molecular mechanism.	3	0.0349 18	
Pubmed	11984513~HLA DRB10405-DQB10401 haplotype is associated with autoimmune pancreatitis in the Japanese population.	3	0.0349 18	
Pubmed	16866883~High-resolution molecular characterization of the HLA class I and class II in the Tarahumara Amerindian population.	3	0.0349 18	
Pubmed	18544086~Impact of HLA-DPB1 allelic and single amino acid mismatches on HSCT.	3	0.0349 18	
Pubmed	8570175~B-ATF: a novel human bZIP protein that associates with members of the AP-1 transcription factor family.	3	0.0349 18	
Pubmed	12774051~The degree of matching at HLA-DPB1 predicts for acute graft-versus-host disease and disease relapse following haematopoietic stem cell transplantation.	3	0.0349 18	
Pubmed	17726164~The importance of HLA-DPB1 in unrelated donor hematopoietic cell transplantation.	3	0.0349 18	
Pubmed	11169242~Allele frequencies and haplotypic associations defined by allelic DNA typing at HLA class I and class II loci in the Japanese population.	3	0.0349 18	
Pubmed	16454711~Modelling thymic HIV-1 Nef effects.	4	0.0380 65	

Pubmed	9263011~gp 120s derived from four syncytium-inducing HIV-1 strains induce different patterns of CD4 association with lymphocyte surface molecules.	4	0.047356	
Pubmed	7541827~HIV gp120 inhibits T cell activation by interfering with expression of costimulatory molecules CD40 ligand and CD80 (B71).	4	0.047356	

Table S3: Enrichment of UFF selected genes for the Hepatitis-C dataset (DAVID and toppgene tools).

Category	Term	Count	%	PValue	Bonferroni	Benjamini
BIOCARTA	h_arenrf2Pathway:Oxidative Stress Induced Gene Expression Via Nrf2	11	2.64%	3.26E-06	1.06E-03	1.06E-03
BIOCARTA	h_classicPathway:Classical Complement Pathway	7	1.68%	2.51E-05	8.12E-03	4.07E-03
BIOCARTA	h_compPathway:Complement Pathway	8	1.92%	3.08E-05	9.98E-03	3.34E-03
GOTERM_BP_ALL	GO:0050896~response to stimulus	190	45.56%	2.47E-55	1.30E-51	1.30E-51
GOTERM_BP_ALL	GO:0006955~immune response	89	21.34%	2.39E-40	1.25E-36	6.27E-37
GOTERM_BP_ALL	GO:0002376~immune system process	99	23.74%	3.96E-38	2.08E-34	6.93E-35
GOTERM_BP_ALL	GO:0009605~response to external stimulus	81	19.42%	6.19E-33	3.25E-29	8.12E-30
GOTERM_BP_ALL	GO:0006952~defense response	75	17.99%	6.12E-32	3.21E-28	6.43E-29
GOTERM_BP_ALL	GO:0009611~response to wounding	66	15.83%	1.44E-31	7.55E-28	1.26E-28
GOTERM_BP_ALL	GO:0006954~inflammatory response	52	12.47%	5.84E-27	3.06E-23	4.38E-24
GOTERM_BP_ALL	GO:0002526~acute inflammatory response	28	6.71%	1.98E-24	1.04E-20	1.30E-21
GOTERM_BP_ALL	GO:0042221~response to chemical stimulus	65	15.59%	2.89E-23	1.52E-19	1.68E-20
GOTERM_BP_ALL	GO:0006950~response to stress	87	20.86%	2.57E-22	1.35E-18	1.35E-19
GOTERM_BP_ALL	GO:0051707~response to other organism	30	7.19%	9.94E-15	5.25E-11	4.77E-12
GOTERM_BP_ALL	GO:0051704~multi-organism process	37	8.87%	2.22E-14	1.17E-10	9.72E-12
GOTERM_BP_ALL	GO:0009607~response to biotic stimulus	35	8.39%	4.37E-14	2.30E-10	1.77E-11
GOTERM_BP_ALL	GO:0006805~xenobiotic metabolic process	15	3.60%	6.47E-14	3.40E-10	2.43E-11
GOTERM_BP_ALL	GO:0009410~response to xenobiotic stimulus	15	3.60%	4.08E-13	2.14E-09	1.43E-10
GOTERM_BP_ALL	GO:0002541~activation of plasma proteins during acute inflammatory response	14	3.36%	2.54E-12	1.33E-08	8.33E-10

GOTERM_BP_ALL	GO:0006956~complement activation	14	3.36%	2.54E-12	1.33E-08	8.33E-10
GOTERM_BP_ALL	GO:0006953~acute-phase response	13	3.12%	3.89E-12	2.05E-08	1.14E-09
GOTERM_BP_ALL	GO:0009615~response to virus	20	4.80%	5.85E-12	3.07E-08	1.62E-09
GOTERM_BP_ALL	GO:0006959~humoral immune response	18	4.32%	7.30E-12	3.83E-08	1.92E-09
GOTERM_BP_ALL	GO:0002455~humoral immune response mediated by circulating immunoglobulin	12	2.88%	9.78E-11	5.14E-07	2.45E-08
GOTERM_BP_ALL	GO:0002253~activation of immune response	16	3.84%	1.90E-10	9.97E-07	4.53E-08
GOTERM_BP_ALL	GO:0050778~positive regulation of immune response	17	4.08%	5.05E-10	2.65E-06	1.15E-07
GOTERM_BP_ALL	GO:0002684~positive regulation of immune system process	17	4.08%	6.09E-10	3.20E-06	1.33E-07
GOTERM_BP_ALL	GO:0050776~regulation of immune response	18	4.32%	8.11E-10	4.26E-06	1.70E-07
GOTERM_BP_ALL	GO:0006958~complement activation, classical pathway	11	2.64%	9.41E-10	4.94E-06	1.90E-07
GOTERM_BP_ALL	GO:0002682~regulation of immune system process	18	4.32%	1.12E-09	5.90E-06	2.18E-07
GOTERM_BP_ALL	GO:0002252~immune effector process	19	4.56%	1.26E-09	6.59E-06	2.36E-07
GOTERM_BP_ALL	GO:0042330~taxis	21	5.04%	1.55E-09	8.12E-06	2.71E-07
GOTERM_BP_ALL	GO:0006935~chemotaxis	21	5.04%	1.55E-09	8.12E-06	2.71E-07
GOTERM_BP_ALL	GO:0051240~positive regulation of multicellular organismal process	17	4.08%	2.06E-08	1.08E-04	3.48E-06
GOTERM_BP_ALL	GO:0007596~blood coagulation	16	3.84%	2.65E-08	1.39E-04	4.35E-06
GOTERM_BP_ALL	GO:0042060~wound healing	18	4.32%	2.87E-08	1.51E-04	4.57E-06
GOTERM_BP_ALL	GO:0050817~coagulation	16	3.84%	3.55E-08	1.86E-04	5.48E-06
GOTERM_BP_ALL	GO:0007626~locomotory behavior	22	5.28%	4.19E-08	2.20E-04	6.29E-06
GOTERM_BP_ALL	GO:0007599~hemostasis	16	3.84%	5.43E-08	2.85E-04	7.93E-06
GOTERM_BP_ALL	GO:0045087~innate immune response	16	3.84%	8.19E-08	4.30E-04	1.16E-05
GOTERM_BP_ALL	GO:0016064~immunoglobulin mediated immune response	12	2.88%	1.49E-07	7.80E-04	2.05E-05
GOTERM_BP_ALL	GO:0002460~adaptive immune response based on somatic recombination of immune receptors built from immunoglobulin superfamily domains	14	3.36%	1.82E-07	9.57E-04	2.46E-05
GOTERM_BP_ALL	GO:0002250~adaptive immune response	14	3.36%	1.82E-07	9.57E-04	2.46E-05
GOTERM_BP_ALL	GO:0065008~regulation of biological quality	50	11.99%	2.04E-07	1.07E-03	2.61E-05
GOTERM_B	GO:0019724~B cell mediated	12	2.88%	2.20E-07	1.16E-03	2.75E-05

P_ALL	immunity					
GOTERM_BP_ALL	GO:0007610~behavior	27	6.47%	5.20E-07	2.73E-03	6.35E-05
GOTERM_BP_ALL	GO:0030005~cellular di-, tri-valent inorganic cation homeostasis	18	4.32%	5.40E-07	2.83E-03	6.44E-05
GOTERM_BP_ALL	GO:0002449~lymphocyte mediated immunity	13	3.12%	5.57E-07	2.92E-03	6.50E-05
GOTERM_BP_ALL	GO:0050878~regulation of body fluid levels	16	3.84%	5.76E-07	3.02E-03	6.57E-05
GOTERM_BP_ALL	GO:0055066~di-, tri-valent inorganic cation homeostasis	18	4.32%	5.94E-07	3.11E-03	6.64E-05
GOTERM_BP_ALL	GO:0002443~leukocyte mediated immunity	13	3.12%	1.32E-06	6.92E-03	1.45E-04
GOTERM_BP_ALL	GO:0030003~cellular cation homeostasis	18	4.32%	3.39E-06	1.76E-02	3.63E-04
GOTERM_BP_ALL	GO:0055080~cation homeostasis	18	4.32%	3.67E-06	1.91E-02	3.85E-04
GOTERM_BP_ALL	GO:0006629~lipid metabolic process	41	9.83%	6.16E-06	3.18E-02	6.34E-04
GOTERM_BP_ALL	GO:0055082~cellular chemical homeostasis	19	4.56%	9.46E-06	4.84E-02	9.55E-04
GOTERM_BP_ALL	GO:0006873~cellular ion homeostasis	19	4.56%	9.46E-06	4.84E-02	9.55E-04
GOTERM_CC_ALL	GO:0005576~extracellular region	113	27.10%	1.20E-37	1.04E-34	1.04E-34
GOTERM_CC_ALL	GO:0005615~extracellular space	65	15.59%	2.29E-27	1.99E-24	9.97E-25
GOTERM_CC_ALL	GO:0044421~extracellular region part	69	16.55%	8.18E-20	7.11E-17	2.37E-17
GOTERM_CC_ALL	GO:0005792~microsome	32	7.67%	2.07E-18	1.80E-15	4.50E-16
GOTERM_CC_ALL	GO:0042598~vesicular fraction	32	7.67%	3.53E-18	3.07E-15	6.14E-16
GOTERM_CC_ALL	GO:0000267~cell fraction	57	13.67%	2.79E-09	2.43E-06	4.05E-07
GOTERM_CC_ALL	GO:0005624~membrane fraction	44	10.55%	2.19E-07	1.90E-04	2.71E-05
GOTERM_CC_ALL	GO:0005783~endoplasmic reticulum	45	10.79%	8.63E-07	7.50E-04	9.38E-05
GOTERM_CC_ALL	GO:0005577~fibrinogen complex	5	1.20%	1.98E-06	1.72E-03	1.92E-04
GOTERM_CC_ALL	GO:0042611~MHC protein complex	9	2.16%	2.03E-06	1.76E-03	1.76E-04
GOTERM_MF_ALL	GO:0004866~endopeptidase inhibitor activity	23	5.52%	3.28E-12	9.42E-09	9.42E-09
GOTERM_MF_ALL	GO:0030414~protease inhibitor activity	23	5.52%	4.40E-12	1.27E-08	6.33E-09
GOTERM_MF_ALL	GO:0015020~glucuronosyltransferase activity	12	2.88%	1.57E-11	4.53E-08	1.51E-08
GOTERM_MF_ALL	GO:0003823~antigen binding	15	3.60%	2.33E-11	6.70E-08	1.68E-08
GOTERM_MF_ALL	GO:0004857~enzyme inhibitor activity	27	6.47%	8.65E-10	2.49E-06	4.98E-07
GOTERM_MF_ALL	GO:0005319~lipid transporter activity	15	3.60%	6.76E-09	1.94E-05	3.24E-06
GOTERM_MF_ALL	GO:0005506~iron ion binding	27	6.47%	7.37E-09	2.12E-05	3.03E-06

GOTERM_MF_ALL	GO:0004867~serine-type endopeptidase inhibitor activity	15	3.60%	3.85E-08	1.11E-04	1.38E-05
GOTERM_MF_ALL	GO:0016712~oxidoreductase activity, acting on paired donors, with incorporation or reduction of molecular oxygen, reduced flavin or flavoprotein as one donor, and incorporation of one atom of oxygen	9	2.16%	4.53E-07	1.30E-03	1.45E-04
GOTERM_MF_ALL	GO:0046870~cadmium ion binding	5	1.20%	1.77E-06	5.08E-03	5.09E-04
GOTERM_MF_ALL	GO:0001664~G-protein-coupled receptor binding	12	2.88%	1.79E-06	5.14E-03	4.69E-04
GOTERM_MF_ALL	GO:0008009~chemokine activity	10	2.40%	2.58E-06	7.39E-03	6.18E-04
GOTERM_MF_ALL	GO:0008194~UDP-glycosyltransferase activity	14	3.36%	2.71E-06	7.75E-03	5.99E-04
GOTERM_MF_ALL	GO:0016491~oxidoreductase activity	42	10.07%	2.91E-06	8.34E-03	5.98E-04
GOTERM_MF_ALL	GO:0042379~chemokine receptor binding	10	2.40%	3.08E-06	8.81E-03	5.90E-04
GOTERM_MF_ALL	GO:0019825~oxygen binding	9	2.16%	3.19E-06	9.13E-03	5.73E-04
GOTERM_MF_ALL	GO:0008201~heparin binding	12	2.88%	3.47E-06	9.94E-03	5.87E-04
GOTERM_MF_ALL	GO:0046906~tetrapyrrole binding	15	3.60%	3.49E-06	9.99E-03	5.57E-04
GOTERM_MF_ALL	GO:0020037~heme binding	15	3.60%	3.49E-06	9.99E-03	5.57E-04
GOTERM_MF_ALL	GO:0001871~pattern binding	14	3.36%	5.46E-06	1.56E-02	7.84E-04
GOTERM_MF_ALL	GO:0005539~glycosaminoglycan binding	13	3.12%	6.83E-06	1.94E-02	9.35E-04
GOTERM_MF_ALL	GO:0016705~oxidoreductase activity, acting on paired donors, with incorporation or reduction of molecular oxygen	14	3.36%	7.25E-06	2.06E-02	9.47E-04
GOTERM_MF_ALL	GO:0030247~polysaccharide binding	13	3.12%	1.02E-05	2.90E-02	1.28E-03
GOTERM_MF_ALL	GO:0005102~receptor binding	39	9.35%	1.14E-05	3.22E-02	1.36E-03
INTERPRO	IPR003597:Immunoglobulin C1-set	21	5.04%	2.09E-18	1.23E-14	1.23E-14
INTERPRO	IPR002213:UDP-glucuronosyl/UDP-glucosyltransferase	12	2.88%	7.68E-14	4.51E-10	2.25E-10
INTERPRO	IPR003596:Immunoglobulin V-set, subgroup	14	3.36%	1.51E-10	8.86E-07	2.95E-07
INTERPRO	IPR003006:Immunoglobulin/major histocompatibility complex motif	16	3.84%	2.17E-10	1.27E-06	3.18E-07
INTERPRO	IPR000436:Sushi/SCR/CCP	13	3.12%	9.17E-09	5.38E-05	1.08E-05
INTERPRO	IPR013106:Immunoglobulin V-set	21	5.04%	3.31E-08	1.94E-04	3.24E-05
INTERPRO	IPR007110:Immunoglobulin-like	32	7.67%	1.14E-07	6.71E-04	9.58E-05
INTERPRO	IPR000006:Metallothionein,	6	1.44%	5.78E-07	3.38E-03	3.77E-04

	vertebrate					
INTERPRO	IPR003019:Metallothionein superfamily	6	1.44%	5.78E-07	3.38E-03	3.77E-04
INTERPRO	IPR013783:Immunoglobulin-like fold	32	7.67%	9.42E-07	5.51E-03	5.53E-04
KEGG_PATHWAY	hsa00980:Metabolism of xenobiotics by cytochrome P450	28	6.71%	4.79E-21	1.11E-18	1.11E-18
KEGG_PATHWAY	hsa04610:Complement and coagulation cascades	24	5.76%	1.16E-15	2.56E-13	1.28E-13
KEGG_PATHWAY	hsa00040:Pentose and glucuronate interconversions	13	3.12%	2.25E-11	5.20E-09	1.73E-09
KEGG_PATHWAY	hsa00860:Porphyrin and chlorophyll metabolism	14	3.36%	2.12E-09	4.91E-07	1.23E-07
KEGG_PATHWAY	hsa00150:Androgen and estrogen metabolism	15	3.60%	2.21E-08	5.11E-06	1.02E-06
PIR_SUPERFAMILY	PIRSF005678:glucuronosyltransferase	12	2.88%	1.32E-11	4.08E-08	4.08E-08
PIR_SUPERFAMILY	PIRSF002367:apolipoprotein A-I	6	1.44%	7.03E-07	2.17E-03	1.09E-03
PIR_SUPERFAMILY	PIRSF002564:metallothionein	6	1.44%	6.13E-06	1.87E-02	6.29E-03
PIR_SUPERFAMILY	PIRSF002488:apolipoprotein H	5	1.20%	1.43E-05	4.32E-02	1.10E-02
SMART	SM00407:IGc1	21	5.04%	6.14E-19	3.72E-16	3.72E-16
SMART	SM00032:CCP	13	3.12%	5.00E-09	3.03E-06	1.51E-06
SMART	SM00406:IGv	15	3.60%	1.15E-08	6.97E-06	2.32E-06
SMART	SM00199:SCY	9	2.16%	6.77E-06	4.10E-03	1.03E-03
SMART	SM00093:SERPIN	8	1.92%	2.72E-05	1.64E-02	3.29E-03
SMART	SM00186:FBG	7	1.68%	3.98E-05	2.38E-02	4.01E-03
SP_PIR_KEYWORDS	Direct protein sequencing	191	45.80%	2.49E-61	2.65E-58	2.65E-58
SP_PIR_KEYWORDS	plasma	46	11.03%	2.60E-48	2.77E-45	1.38E-45
SP_PIR_KEYWORDS	Secreted	112	26.86%	8.15E-31	8.68E-28	2.89E-28
SP_PIR_KEYWORDS	acute phase	20	4.80%	3.99E-24	4.25E-21	1.06E-21
SP_PIR_KEYWORDS	signal	148	35.49%	1.57E-22	1.67E-19	3.34E-20
SP_PIR_KEYWORDS	liver	22	5.28%	1.31E-20	1.40E-17	2.33E-18
SP_PIR_KEYWORDS	microsome	26	6.24%	6.53E-18	6.95E-15	9.93E-16
SP_PIR_KEYWORDS	inflammation	12	2.88%	1.20E-12	1.27E-09	1.59E-10
SP_PIR_KEYWORDS	pyrrolidone carboxylic acid	17	4.08%	1.32E-12	1.41E-09	1.57E-10
SP_PIR_KEYWORDS	innate immunity	15	3.60%	5.04E-12	5.37E-09	5.37E-10
SP_PIR_KEYWORDS	duplication	27	6.47%	7.23E-11	7.70E-08	7.00E-09
SP_PIR_KEYWORDS	immune response	22	5.28%	1.00E-10	1.07E-07	8.88E-09
SP_PIR_KEYWORDS	heterotetramer	14	3.36%	1.12E-10	1.20E-07	9.20E-09
SP_PIR_KE	glycoprotein	139	33.33%	1.20E-10	1.28E-07	9.12E-09

YWORDS						
SP_PIR_KEYWORDS	sushi	14	3.36%	1.97E-10	2.10E-07	1.40E-08
SP_PIR_KEYWORDS	immunoglobulin	10	2.40%	2.68E-10	2.85E-07	1.78E-08
SP_PIR_KEYWORDS	metalloprotein	20	4.80%	3.01E-10	3.20E-07	1.88E-08
SP_PIR_KEYWORDS	chromoprotein	15	3.60%	3.05E-10	3.25E-07	1.80E-08
SP_PIR_KEYWORDS	complement pathway	11	2.64%	5.12E-10	5.45E-07	2.87E-08
SP_PIR_KEYWORDS	pyroglutamic acid	13	3.12%	9.55E-10	1.02E-06	5.08E-08
SP_PIR_KEYWORDS	Immunoglobulin C region	8	1.92%	1.81E-09	1.92E-06	9.16E-08
SP_PIR_KEYWORDS	iron	25	6.00%	4.52E-09	4.82E-06	2.19E-07
SP_PIR_KEYWORDS	oxidoreductase	37	8.87%	1.22E-08	1.30E-05	5.67E-07
SP_PIR_KEYWORDS	heme	17	4.08%	1.30E-08	1.38E-05	5.77E-07
SP_PIR_KEYWORDS	hdl	8	1.92%	3.23E-08	3.44E-05	1.38E-06
SP_PIR_KEYWORDS	transmembrane protein	41	9.83%	4.77E-08	5.08E-05	1.95E-06
SP_PIR_KEYWORDS	Serine protease inhibitor	13	3.12%	5.36E-08	5.71E-05	2.11E-06
SP_PIR_KEYWORDS	chelation	6	1.44%	5.72E-08	6.10E-05	2.18E-06
SP_PIR_KEYWORDS	inflammatory response	13	3.12%	6.43E-08	6.85E-05	2.36E-06
SP_PIR_KEYWORDS	blood coagulation	11	2.64%	8.13E-08	8.65E-05	2.88E-06
SP_PIR_KEYWORDS	lipid binding	8	1.92%	1.50E-07	1.59E-04	5.14E-06
SP_PIR_KEYWORDS	endoplasmic reticulum	37	8.87%	1.82E-07	1.94E-04	6.05E-06
SP_PIR_KEYWORDS	protease inhibitor	14	3.36%	2.46E-07	2.62E-04	7.93E-06
SP_PIR_KEYWORDS	serine proteinase	11	2.64%	3.05E-07	3.24E-04	9.54E-06
SP_PIR_KEYWORDS	acetylation	38	9.11%	3.06E-07	3.26E-04	9.31E-06
SP_PIR_KEYWORDS	metal-thiolate cluster	6	1.44%	5.12E-07	5.46E-04	1.52E-05
SP_PIR_KEYWORDS	hexosyltransferase	9	2.16%	5.31E-07	5.65E-04	1.53E-05
SP_PIR_KEYWORDS	metal binding	7	1.68%	1.56E-06	1.66E-03	4.38E-05
SP_PIR_KEYWORDS	acetylated amino end	13	3.12%	1.72E-06	1.83E-03	4.70E-05
SP_PIR_KEYWORDS	cadmium	5	1.20%	1.92E-06	2.04E-03	5.11E-05
SP_PIR_KEYWORDS	homodimer	13	3.12%	1.95E-06	2.07E-03	5.06E-05
SP_PIR_KEYWORDS	chylomicron	6	1.44%	2.21E-06	2.35E-03	5.61E-05
SP_PIR_KEYWORDS	glycation	6	1.44%	3.97E-06	4.22E-03	9.84E-05

SP_PIR_KEYWORDS	vldl	6	1.44%	3.97E-06	4.22E-03	9.84E-05
SP_PIR_KEYWORDS	immunoglobulin domain	28	6.71%	4.74E-06	5.03E-03	1.12E-04
SP_PIR_KEYWORDS	amyloid	7	1.68%	4.96E-06	5.27E-03	1.15E-04
SP_PIR_KEYWORDS	complement alternate pathway	6	1.44%	6.67E-06	7.08E-03	1.51E-04
SP_PIR_KEYWORDS	antiviral defense	9	2.16%	6.80E-06	7.22E-03	1.51E-04
SP_PIR_KEYWORDS	chemotaxis	11	2.64%	7.05E-06	7.48E-03	1.53E-04
SP_PIR_KEYWORDS	extracellular protein	8	1.92%	9.32E-06	9.88E-03	1.99E-04
SP_PIR_KEYWORDS	heparin-binding	10	2.40%	1.30E-05	1.37E-02	2.71E-04
SP_PIR_KEYWORDS	disease mutation	61	14.63%	1.51E-05	1.60E-02	3.10E-04
SP_PIR_KEYWORDS	electron transfer	9	2.16%	2.70E-05	2.83E-02	5.41E-04
SP_PIR_KEYWORDS	heparin binding	7	1.68%	2.85E-05	2.99E-02	5.62E-04
SP_PIR_KEYWORDS	antimicrobial	9	2.16%	3.14E-05	3.29E-02	6.08E-04
UP_SEQ_FEATURE	signal peptide	142	34.05%	1.06E-20	1.90E-16	1.90E-16
UP_SEQ_FEATURE	disulfide bond	122	29.26%	2.65E-18	4.78E-14	2.39E-14
UP_SEQ_FEATURE	sequence variant	217	52.04%	1.70E-14	3.06E-10	1.02E-10
UP_SEQ_FEATURE	glycosylation site:O-linked (GalNAc...)	16	3.84%	5.79E-10	1.04E-05	2.61E-06
UP_SEQ_FEATURE	domain:Sushi 2	12	2.88%	9.50E-10	1.71E-05	2.85E-06
UP_SEQ_FEATURE	domain:Sushi 1	12	2.88%	9.50E-10	1.71E-05	2.85E-06
UP_SEQ_FEATURE	glycosylation site:N-linked (GlcNAc...)	121	29.02%	3.30E-07	5.93E-03	8.50E-04
UP_SEQ_FEATURE	region of interest:Beta	6	1.44%	4.39E-07	7.88E-03	9.88E-04
UP_SEQ_FEATURE	region of interest:Alpha	6	1.44%	4.39E-07	7.88E-03	9.88E-04
UP_SEQ_FEATURE	metal ion-binding site:Cluster B	6	1.44%	1.14E-06	2.04E-02	1.71E-03
UP_SEQ_FEATURE	metal ion-binding site:Cluster A	6	1.44%	1.14E-06	2.04E-02	1.71E-03
UP_SEQ_FEATURE	glycosylation site:N-linked (Glc) (glycation)	6	1.44%	1.14E-06	2.04E-02	1.71E-03

**Category**	**Term**	**Hit Count in Query List**	**P-value**	**Source**
GO: Molecular Function	GO:0030246~carbohydrate binding	24	0.000104	
GO: Molecular Function	GO:0070325~lipoprotein receptor binding	6	0.000342	

GO: Molecular Function	GO:0030234~enzyme regulator activity	41	0.000448	
GO: Molecular Function	GO:0043498~cell surface binding	7	0.001103	
GO: Molecular Function	GO:0055102~lipase inhibitor activity	5	0.00623	
GO: Molecular Function	GO:0005496~steroid binding	12	0.006689	
GO: Molecular Function	GO:0016616~oxidoreductase activity, acting on the CH-OH group of donors, NAD or NADP as acceptor	11	0.007217	
GO: Molecular Function	GO:0043499~eukaryotic cell surface binding	5	0.016875	
GO: Molecular Function	GO:0016614~oxidoreductase activity, acting on CH-OH group of donors	11	0.01876	
GO: Molecular Function	GO:0008289~lipid binding	25	0.019078	
GO: Molecular Function	GO:0030492~hemoglobin binding	3	0.022517	
GO: Molecular Function	GO:0060228~phosphatidylcholine-sterol O-acyltransferase activator activity	3	0.022517	
GO: Biological Process	GO:0048583~regulation of response to stimulus	41	0	
GO: Biological Process	GO:0019752~carboxylic acid metabolic process	41	0	
GO: Biological Process	GO:0006082~organic acid metabolic process	41	0	
GO: Biological Process	GO:0008202~steroid metabolic process	24	0	
GO: Biological Process	GO:0034369~plasma lipoprotein particle remodeling	9	0	
GO: Biological Process	GO:0034368~protein-lipid complex remodeling	9	0	
GO: Biological Process	GO:0034367~macromolecular complex remodeling	9	0	
GO: Biological Process	GO:0048584~positive regulation of response to stimulus	24	0.000001	
GO: Biological Process	GO:0044255~cellular lipid metabolic process	44	0.000003	
GO:	GO:0006641~triglyceride	11	0.0000	

Biological Process	metabolic process		06	
GO: Biological Process	GO:0032101~regulation of response to external stimulus	18	0.0000 07	
GO: Biological Process	GO:0032787~monocarboxylic acid metabolic process	26	0.0000 17	
GO: Biological Process	GO:0019439~aromatic compound catabolic process	8	0.0000 23	
GO: Biological Process	GO:0032368~regulation of lipid transport	8	0.0000 36	
GO: Biological Process	GO:0032374~regulation of cholesterol transport	7	0.0000 37	
GO: Biological Process	GO:0032371~regulation of sterol transport	7	0.0000 37	
GO: Biological Process	GO:0006639~acylglycerol metabolic process	11	0.0000 42	
GO: Biological Process	GO:0040011~locomotion	36	0.0000 44	
GO: Biological Process	GO:0033344~cholesterol efflux	8	0.0000 54	
GO: Biological Process	GO:0033700~phospholipid efflux	6	0.0000 59	
GO: Biological Process	GO:0006638~neutral lipid metabolic process	11	0.0000 64	
GO: Biological Process	GO:0046486~glycerolipid metabolic process	11	0.0000 64	
GO: Biological Process	GO:0015918~sterol transport	10	0.0000 71	
GO: Biological Process	GO:0030301~cholesterol transport	10	0.0000 71	
GO: Biological Process	GO:0006662~glycerol ether metabolic process	11	0.0000 78	
GO: Biological Process	GO:0006066~cellular alcohol metabolic process	28	0.0000 79	
GO: Biological Process	GO:0019216~regulation of lipid metabolic process	13	0.0000 83	
GO: Biological Process	GO:0034381~lipoprotein particle clearance	7	0.0001 07	
GO: Biological Process	GO:0030168~platelet activation	9	0.0001 38	

GO: Biological Process	GO:0019835~cytolysis	8	0.0001 62	
GO: Biological Process	GO:0044242~cellular lipid catabolic process	14	0.0001 63	
GO: Biological Process	GO:0034384~high-density lipoprotein particle clearance	5	0.0002 73	
GO: Biological Process	GO:0065005~protein-lipid complex assembly	6	0.0004 52	
GO: Biological Process	GO:0034377~plasma lipoprotein particle assembly	6	0.0004 52	
GO: Biological Process	GO:0055114~oxidation reduction	36	0.0004 6	
GO: Biological Process	GO:0009617~response to bacterium	17	0.0007 38	
GO: Biological Process	GO:0045834~positive regulation of lipid metabolic process	8	0.0009 7	
GO: Biological Process	GO:0050727~regulation of inflammatory response	11	0.0010 41	
GO: Biological Process	GO:0031099~regeneration	8	0.0012 56	
GO: Biological Process	GO:0010033~response to organic substance	15	0.0013 83	
GO: Biological Process	GO:0034372~very-low-density lipoprotein particle remodeling	5	0.0015 8	
GO: Biological Process	GO:0034370~triglyceride-rich lipoprotein particle remodeling	5	0.0015 8	
GO: Biological Process	GO:0055072~iron ion homeostasis	8	0.0016 1	
GO: Biological Process	GO:0030195~negative regulation of blood coagulation	7	0.0016 62	
GO: Biological Process	GO:0045833~negative regulation of lipid metabolic process	7	0.0016 62	
GO: Biological Process	GO:0031347~regulation of defense response	14	0.0017 19	
GO: Biological Process	GO:0016125~sterol metabolic process	13	0.0018 15	
GO: Biological Process	GO:0043691~reverse cholesterol transport	6	0.0019 91	
GO: Biological	GO:0048878~chemical homeostasis	30	0.0020 71	

Process				
GO: Biological Process	GO:0034382~chylomicron remnant clearance	4	0.002969	
GO: Biological Process	GO:0032372~negative regulation of sterol transport	4	0.002969	
GO: Biological Process	GO:0032375~negative regulation of cholesterol transport	4	0.002969	
GO: Biological Process	GO:0032879~regulation of localization	31	0.003132	
GO: Biological Process	GO:0008203~cholesterol metabolic process	12	0.004409	
GO: Biological Process	GO:0050994~regulation of lipid catabolic process	6	0.004442	
GO: Biological Process	GO:0050819~negative regulation of coagulation	7	0.005261	
GO: Biological Process	GO:0046503~glycerolipid catabolic process	5	0.005581	
GO: Biological Process	GO:0046464~acylglycerol catabolic process	5	0.005581	
GO: Biological Process	GO:0044269~glycerol ether catabolic process	5	0.005581	
GO: Biological Process	GO:0046461~neutral lipid catabolic process	5	0.005581	
GO: Biological Process	GO:0008219~cell death	50	0.006304	
GO: Biological Process	GO:0006879~cellular iron ion homeostasis	7	0.006801	
GO: Biological Process	GO:0016265~death	50	0.007334	
GO: Biological Process	GO:0042592~homeostatic process	40	0.007804	
GO: Biological Process	GO:0010872~regulation of cholesterol esterification	4	0.008748	
GO: Biological Process	GO:0010873~positive regulation of cholesterol esterification	4	0.008748	
GO: Biological Process	GO:0034447~very-low-density lipoprotein particle clearance	4	0.008748	
GO: Biological Process	GO:0006979~response to oxidative stress	13	0.009051	
GO:	GO:0016042~lipid catabolic	16	0.0097	

Biological Process	process		59	
GO: Biological Process	GO:0030193~regulation of blood coagulation	7	0.0110 2	
GO: Biological Process	GO:0006631~fatty acid metabolic process	18	0.0111 89	
GO: Biological Process	GO:0050793~regulation of developmental process	57	0.0115 3	
GO: Biological Process	GO:0034641~cellular nitrogen compound metabolic process	26	0.0122 55	
GO: Biological Process	GO:0006915~apoptosis	46	0.0127 72	
GO: Biological Process	GO:0051239~regulation of multicellular organismal process	41	0.0148 92	
GO: Biological Process	GO:0002920~regulation of humoral immune response	5	0.0149 76	
GO: Biological Process	GO:0032369~negative regulation of lipid transport	5	0.0149 76	
GO: Biological Process	GO:0051093~negative regulation of developmental process	31	0.0155 79	
GO: Biological Process	GO:0010743~regulation of foam cell differentiation	6	0.0165 37	
GO: Biological Process	GO:0012501~programmed cell death	46	0.0177 8	
GO: Biological Process	GO:0060192~negative regulation of lipase activity	4	0.0200 48	
GO: Biological Process	GO:0010903~negative regulation of very-low-density lipoprotein particle remodeling	3	0.0269 69	
GO: Biological Process	GO:0006807~nitrogen compound metabolic process	26	0.0275 16	
GO: Biological Process	GO:0051004~regulation of lipoprotein lipase activity	5	0.0336 64	
GO: Biological Process	GO:0006957~complement activation, alternative pathway	5	0.0336 64	
GO: Biological Process	GO:0055088~lipid homeostasis	8	0.0347 47	
GO: Biological Process	GO:0001775~cell activation	24	0.0350 16	
GO: Biological Process	GO:0048585~negative regulation of response to stimulus	10	0.0363 84	

GO: Biological Process	GO:0030212~hyaluronan metabolic process	4	0.0393 8	
GO: Biological Process	GO:0034374~low-density lipoprotein particle remodeling	4	0.0393 8	
GO: Biological Process	GO:0055092~sterol homeostasis	7	0.0461 63	
GO: Biological Process	GO:0042632~cholesterol homeostasis	7	0.0461 63	
GO: Biological Process	GO:0050818~regulation of coagulation	7	0.0461 63	
GO: Cellular Component	GO:0034364~high-density lipoprotein particle	12	0	
GO: Cellular Component	GO:0034358~plasma lipoprotein particle	13	0	
GO: Cellular Component	GO:0032994~protein-lipid complex	13	0	
GO: Cellular Component	GO:0034366~spherical high-density lipoprotein particle	7	0	
GO: Cellular Component	GO:0031093~platelet alpha granule lumen	10	0	
GO: Cellular Component	GO:0042627~chylomicron	7	0	
GO: Cellular Component	GO:0031091~platelet alpha granule	11	0	
GO: Cellular Component	GO:0060205~cytoplasmic membrane-bounded vesicle lumen	10	0	
GO: Cellular Component	GO:0031983~vesicle lumen	10	0	
GO: Cellular Component	GO:0034385~triglyceride-rich lipoprotein particle	7	0.0000 02	
GO: Cellular Component	GO:0034361~very-low-density lipoprotein particle	7	0.0000 02	
GO: Cellular Component	GO:0034363~intermediate-density lipoprotein particle	4	0.0000 45	
GO: Cellular Component	GO:0005626~insoluble fraction	35	0.0085 9	
GO: Cellular Component	GO:0044432~endoplasmic reticulum part	29	0.0127 18	
GO: Cellular Component	GO:0042613~MHC class II protein complex	4	0.0383 7	
Human Phenotype	HP:0001939~Metabolism abnormality	78	0	
Human Phenotype	HP:0003216~Generalized amyloid deposition	4	0.0231 67	
Human Phenotype	HP:0002621~Atherosclerosis	8	0.0455 74	
Mouse Phenotype	MP:0001793~altered susceptibility to infection	43	0	
Mouse Phenotype	MP:0005025~abnormal response to infection	43	0	
Mouse Phenotype	MP:0002419~abnormal innate immunity	41	0	

Mouse Phenotype	MP:0002406~increased susceptibility to infection	34	0	
Mouse Phenotype	MP:0001790~abnormal immune system physiology	88	0	
Mouse Phenotype	MP:0002723~abnormal immune serum protein physiology	52	0.0000 01	
Mouse Phenotype	MP:0002421~abnormal cell-mediated immunity	64	0.0000 01	
Mouse Phenotype	MP:0001819~abnormal immune cell physiology	64	0.0000 01	
Mouse Phenotype	MP:0002420~abnormal adaptive immunity	64	0.0000 01	
Mouse Phenotype	MP:0002442~abnormal leukocyte physiology	63	0.0000 02	
Mouse Phenotype	MP:0002488~abnormal phagocyte physiology	33	0.0000 03	
Mouse Phenotype	MP:0002452~abnormal antigen presenting cell physiology	47	0.0000 12	
Mouse Phenotype	MP:0002139~abnormal liver/biliary system physiology	29	0.0000 13	
Mouse Phenotype	MP:0005428~abnormal clinical chemistry	76	0.0000 13	
Mouse Phenotype	MP:0002412~increased susceptibility to bacterial infection	23	0.0000 13	
Mouse Phenotype	MP:0000179~abnormal blood chemistry	72	0.0000 29	
Mouse Phenotype	MP:0000609~abnormal liver physiology	26	0.0001 13	
Mouse Phenotype	MP:0008469~abnormal protein level	32	0.0001 25	
Mouse Phenotype	MP:0005370~liver/biliary system phenotype	49	0.0001 67	
Mouse Phenotype	MP:0001800~abnormal humoral immune response	32	0.0002 35	
Mouse Phenotype	MP:0002501~abnormal inflammatory mediator physiology	38	0.0002 43	
Mouse Phenotype	MP:0002451~abnormal macrophage physiology	26	0.0002 49	
Mouse Phenotype	MP:0005164~abnormal response to injury	25	0.0005 4	
Mouse Phenotype	MP:0001657~abnormal induced morbidity/mortality	26	0.0009 14	
Mouse Phenotype	MP:0005397~hematopoietic system phenotype	82	0.0009 58	
Mouse Phenotype	MP:0001845~abnormal inflammatory response	56	0.0014 3	
Mouse Phenotype	MP:0005502~abnormal urinary system physiology	33	0.0018 88	
Mouse Phenotype	MP:0005416~abnormal circulating protein level	26	0.0025 94	
Mouse Phenotype	MP:0005338~atherosclerotic lesions	11	0.0027 94	

Mouse Phenotype	MP:0000685~abnormal immune system morphology	72	0.0029 49	
Mouse Phenotype	MP:0000249~abnormal blood vessel physiology	26	0.0039 58	
Mouse Phenotype	MP:0003991~arteriosclerosis	11	0.0041 3	
Mouse Phenotype	MP:0002463~abnormal neutrophil physiology	15	0.0044 52	
Mouse Phenotype	MP:0002396~abnormal hematopoietic system morphology/development	78	0.0061 36	
Mouse Phenotype	MP:0005000~abnormal immune tolerance	23	0.0068 83	
Mouse Phenotype	MP:0002644~decreased circulating triglyceride level	12	0.0122 63	
Mouse Phenotype	MP:0005318~decreased triglyceride level	14	0.0125 85	
Mouse Phenotype	MP:0000716~abnormal immune system cell morphology	60	0.0129 65	
Mouse Phenotype	MP:0005005~abnormal self tolerance	22	0.0154 81	
Mouse Phenotype	MP:0001844~autoimmune response	22	0.0154 81	
Mouse Phenotype	MP:0008246~abnormal leukocyte morphology	59	0.0178 22	
Mouse Phenotype	MP:0003009~abnormal cytokine secretion	28	0.0219 15	
Mouse Phenotype	MP:0008250~abnormal myeloid leukocyte morphology	32	0.0232 67	
Mouse Phenotype	MP:0003945~abnormal lymphocyte physiology	43	0.0301 88	
Mouse Phenotype	MP:0008251~abnormal phagocyte morphology	28	0.0305 6	
Mouse Phenotype	MP:0001846~increased inflammatory response	38	0.0341 46	
Mouse Phenotype	MP:0008195~abnormal antigen presenting cell morphology	30	0.0418 27	
Mouse Phenotype	MP:0002411~decreased susceptibility to bacterial infection	10	0.0421 87	
Mouse Phenotype	MP:0002468~abnormal complement physiology	6	0.0445 75	
Mouse Phenotype	MP:0005146~decreased circulating VLDL cholesterol level	6	0.0445 75	
Mouse Phenotype	MP:0002551~abnormal blood coagulation	19	0.0462 04	
Mouse Phenotype	MP:0002462~abnormal granulocyte physiology	17	0.0491 03	
Mouse Phenotype	MP:0005144~abnormal circulating VLDL cholesterol level	8	0.0491 77	
Pathway	hsa04610~Complement and coagulation cascades	24	0	KEGG pathway

Pathway	hsa00980~Metabolism of xenobiotics by cytochrome P450	23	0	KEGG pathway
Pathway	140192~Xenobiotic metabolism	22	0	Reactome
Pathway	hsa00982~Drug metabolism - cytochrome P450	20	0	KEGG pathway
Pathway	hsa00830~Retinol metabolism	18	0	KEGG pathway
Pathway	MAP00071_Fatty_acid_metabolism~MAP00071 Fatty acid metabolism	14	0	GenMAPP
Pathway	109582~Hemostasis	18	0.000001	Reactome
Pathway	MAP00380_Tryptophan_metabolism~MAP00380 Tryptophan metabolism	13	0.000001	GenMAPP
Pathway	TRYPTOPHAN_METABOLISM~TRYPTOPHAN_METABOLISM	14	0.000002	MSigDB
Pathway	PW:0000484~altered lipoprotein metabolic	6	0.000007	Pathway Ontology
Pathway	ANDROGEN_AND_ESTROGEN_METABOLISM~ANDROGEN_AND_ESTROGEN_METABOLISM	10	0.000019	MSigDB
Pathway	FIBRINOLYSISPATHWAY~FIBRINOLYSISPATHWAY	7	0.000023	MSigDB
Pathway	PENTOSE_AND_GLUCURONATE_INTERCONVERSIONS~PENTOSE_AND_GLUCURONATE_INTERCONVERSIONS	8	0.000036	MSigDB
Pathway	hsa00040~Pentose and glucuronate interconversions	9	0.000045	KEGG pathway
Pathway	PW:0000482~lipoprotein metabolic	7	0.000049	Pathway Ontology
Pathway	hsa00983~Drug metabolism - other enzymes	12	0.000059	KEGG pathway
Pathway	COMPPATHWAY~COMPPATHWAY	8	0.00006	MSigDB
Pathway	hsa00053~Ascorbate and aldarate metabolism	9	0.000067	KEGG pathway
Pathway	CLASSICPATHWAY~CLASSICPATHWAY	7	0.000094	MSigDB
Pathway	P00011~Blood coagulation	10	0.00018	PantherDB
Pathway	INTRINSICPATHWAY~INTRINSICPATHWAY	8	0.000347	MSigDB
Pathway	hsa00860~Porphyrin and chlorophyll metabolism	10	0.000515	KEGG pathway
Pathway	IRINOTECAN_PATHWAY_PHARMGKB~IRINOTECAN_PATHWAY_PHARMGKB	7	0.00078	MSigDB
Pathway	PORPHYRIN_AND_CHLOROPHYLL_METABOLISM~PORPHYRIN_AND_CHLOROPHYLL_METABOLISM	8	0.001014	MSigDB
Pathway	P00050~Plasminogen	6	0.0014	PantherD

	activating cascade		46	B
Pathway	hsa00150~Androgen and estrogen metabolism	10	0.0019 93	KEGG pathway
Pathway	MAP00361_gamma_Hexachlo rocyclohexane_degradation~M AP00361 gamma Hexachlorocyclohexane degradation	8	0.0025 52	GenMAP P
Pathway	BLOOD_CLOTTING_CASCA DE~BLOOD_CLOTTING_CAS CADE	7	0.0026 14	MSigDB
Pathway	ALTERNATIVEPATHWAY~AL TERNATIVEPATHWAY	5	0.0035 26	MSigDB
Pathway	GAMMA_HEXACHLOROCYC LOHEXANE_DEGRADATION ~GAMMA_HEXACHLOROCY CLOHEXANE_DEGRADATIO N	8	0.0057 31	MSigDB
Pathway	h_intrinsicPathway~Intrinsic Prothrombin Activation	6	0.0061 99	CGAP BioCarta
Pathway	h_alternativePathway~Alternat ive Complement	5	0.0068 58	CGAP BioCarta
Pathway	LAIRPATHWAY~LAIRPATHW AY	6	0.0093 12	MSigDB
Pathway	BILE_ACID_BIOSYNTHESIS~ BILE_ACID_BIOSYNTHESIS	7	0.0167 96	MSigDB
Pathway	h_lectinPathway~Lectin Induced Complement	5	0.0203 94	CGAP BioCarta
Pathway	EXTRINSICPATHWAY~EXTR INSICPATHWAY	5	0.0322 36	MSigDB
Pathway	71291~Metabolism of nitrogenous molecules	10	0.0353 22	Reactom e
Pathway	hsa00591~Linoleic acid metabolism	7	0.0353 26	KEGG pathway
Pubmed	14718574~The human plasma proteome: a nonredundant list developed by combination of four separate sources.	52	0	
Pubmed	14760718~Screening for N-glycosylated proteins by liquid chromatography mass spectrometry.	32	0	
Pubmed	16335952~Human plasma N-glycoproteome analysis by immunoaffinity subtraction, hydrazide chemistry, and mass spectrometry.	55	0	
Pubmed	12539042~HIV-1 Tat reprograms immature dendritic cells to express chemoattractants for activated T cells and macrophages.	22	0	
Pubmed	17703412~Genetic susceptibility to respiratory syncytial virus bronchiolitis is predominantly associated with innate immune genes.	27	0	

Pubmed	18842294~Association between the SERPING1 gene and age-related macular degeneration: a two-stage case-control study.	12	0	
Pubmed	15084671~A proteomic analysis of human bile.	10	0	
Pubmed	12754519~Identification and quantification of N-linked glycoproteins using hydrazide chemistry, stable isotope labeling and mass spectrometry.	14	0	
Pubmed	11752456~Insight into hepatocellular carcinogenesis at transcriptome level by comparing gene expression profiles of hepatocellular carcinoma with those of corresponding noncancerous liver.	13	0.000001	
Pubmed	18818748~Preterm birth in Caucasians is associated with coagulation and inflammation pathway gene variants.	17	0.000001	
Pubmed	16740002~Identification of N-linked glycoproteins in human saliva by glycoprotein capture and mass spectrometry.	11	0.000001	
Pubmed	4345202~Interchange of apolipoproteins between chylomicrons and high density lipoproteins during alimentary lipemia in man.	6	0.000003	
Pubmed	18936436~Prevalence in the United States of Selected Candidate Gene Variants: Third National Health and Nutrition Examination Survey, 1991-1994.	11	0.000008	
Pubmed	11162594~Apolipoprotein specificity for lipid efflux by the human ABCAI transporter.	6	0.000012	
Pubmed	16682745~Isolation and characterization of human apolipoprotein M-containing lipoproteins.	6	0.000012	
Pubmed	1602151~Treatment of Haemophilus aphrophilus endocarditis with ciprofloxacin.	14	0.000028	
Pubmed	18660489~Multiple genetic variants along candidate pathways influence plasma high-density lipoprotein cholesterol concentrations.	21	0.000029	
Pubmed	18636124~Polymorphisms in the estrogen receptor 1 and vitamin C and matrix metalloproteinase gene	16	0.000042	

	families are associated with susceptibility to lymphoma.			
Pubmed	14746139~Molecular variation at functional genes and the history of human populations--data on candidate genes for cardiovascular risk in the Mediterranean.	7	0.0000 43	
Pubmed	17048007~Association of warfarin dose with genes involved in its action and metabolism.	7	0.0000 75	
Pubmed	18974842~Gender differences in genetic risk profiles for cardiovascular disease.	10	0.0000 75	
Pubmed	12082592~Multi-locus interactions predict risk for post-PTCA restenosis: an approach to the genetic analysis of common complex disease.	11	0.0000 83	
Pubmed	16006997~A comprehensive analysis of phase I and phase II metabolism gene polymorphisms and risk of colorectal cancer.	8	0.0001 02	
Pubmed	10774930~Metallothionein isoform gene expression in zinc-treated human peripheral blood lymphocytes.	5	0.0001 67	
Pubmed	15648851~Phenotype and allele frequencies of some serum protein polymorphisms in populations of the Balkans.	5	0.0001 67	
Pubmed	15469410~Genetic variation in eleven phase I drug metabolism genes in an ethnically diverse population.	6	0.0001 97	
Pubmed	12624729~Identification of a novel human angiopoietin-like gene expressed mainly in heart.	6	0.0001 97	
Pubmed	16199891~Comparative proteomic analysis of intra- and interindividual variation in human cerebrospinal fluid.	7	0.0002 02	
Pubmed	12732844~A study to survey susceptible genetic factors responsible for troglitazone-associated hepatotoxicity in Japanese patients with type 2 diabetes mellitus.	9	0.0003 37	
Pubmed	11714857~Genetic influences on lipid metabolism trait variability within the Stanislas Cohort.	6	0.0003 89	

Pubmed	11083865~Versican interacts with chemokines and modulates cellular responses.	6	0.000389	
Pubmed	15774926~Association study of detoxification genes in age related macular degeneration.	6	0.000389	
Pubmed	18161889~Detoxification enzyme polymorphisms are not involved in duodenal adenomatosis in familial adenomatous polyposis.	6	0.000389	
Pubmed	15076187~Genetic influences on blood pressure within the Stanislas Cohort.	6	0.000713	
Pubmed	9890157~Molecular genetics of the human cytochrome P450 monooxygenase superfamily.	7	0.001024	
Pubmed	9295054~The UDP glycosyltransferase gene superfamily: recommended nomenclature update based on evolutionary divergence.	6	0.001231	
Pubmed	18193044~Six new loci associated with blood low-density lipoprotein cholesterol, high-density lipoprotein cholesterol or triglycerides in humans.	8	0.001499	
Pubmed	15319294~Genetic polymorphisms in UDP-glucuronosyltransferases and glutathione S-transferases and colorectal cancer risk.	5	0.001516	
Pubmed	2465147~Human plasma inter-alpha-trypsin inhibitor is encoded by four genes on three chromosomes.	4	0.001731	
Pubmed	18512730~Translationally regulated C/EBP beta isoform expression upregulates metastatic genes in hormone-independent prostate cancer cells.	4	0.001731	
Pubmed	8245722~Identification of disulfide-linked apolipoprotein species in human lipoproteins.	4	0.001731	
Pubmed	18257091~ProApolipoprotein A1: a serum marker of brain metastases in lung cancer patients.	4	0.001731	
Pubmed	2742826~High-resolution NMR studies of fibrinogen-like peptides in solution: interaction of thrombin with residues 1-23 of the A alpha chain of human fibrinogen.	4	0.001731	

Pubmed	6328445~Human apolipoproteins AI, AII, CII and CIII. cDNA sequences and mRNA abundance.	4	0.0017 31	
Pubmed	16136540~Apolipoproteins (apoproteins) and LPL variation in Mennonite populations of Kansas and Nebraska.	4	0.0017 31	
Pubmed	11367533~Complement C3b interactions studied with surface plasmon resonance technique.	4	0.0017 31	
Pubmed	1385302~Homologous chromosomal locations of the four genes for inter-alpha-inhibitor and pre-alpha-inhibitor family in human and mouse: assignment of the ancestral gene for the lipocalin superfamily.	4	0.0017 31	
Pubmed	17482181~Levels of complement components iC3b, C3c, C4, and SC5b-9 in peritoneal fluid and serum of infertile women with endometriosis.	4	0.0017 31	
Pubmed	11341749~Candidate gene polymorphisms in cardiovascular disease: a comparative study of frequencies between a French and an Italian population.	6	0.0020 23	
Pubmed	18990750~Red meat intake, doneness, polymorphisms in genes that encode carcinogen-metabolizing enzymes, and colorectal cancer risk.	6	0.0020 23	
Pubmed	18927546~Lp(a) and Risk of Recurrent Cardiac Events in Obese Postinfarction Patients.	6	0.0048 65	
Pubmed	11465080~UDP-glucuronosyltransferases.	5	0.0066 4	
Pubmed	18193043~Newly identified loci that influence lipid concentrations and risk of coronary artery disease.	7	0.0084 82	
Pubmed	8630395~Identification of complement activation sites in human immunodeficiency virus type-1 glycoprotein gp120.	4	0.0085 41	
Pubmed	12782148~Effects of plasma apolipoproteins on lipoprotein lipase-mediated lipolysis of small and large lipid emulsions.	4	0.0085 41	

Pubmed	11434514~Thirteen UDPglucuronosyltransferase genes are encoded at the human UGT1 gene complex locus.	5	0.012008	
Pubmed	16538176~Genetic susceptibility to carbamazepine-induced cutaneous adverse drug reactions.	5	0.012008	
Pubmed	12447867~Genetic polymorphisms in interferon pathway and response to interferon treatment in hepatitis B patients: A pilot study.	5	0.012008	
Pubmed	16985026~Metabolic gene variants and risk of non-Hodgkin's lymphoma.	5	0.012008	
Pubmed	14672974~Multiple variable first exons: a mechanism for cell- and tissue-specific gene regulation.	5	0.020307	
Pubmed	2286373~Human metallothionein genes: structure of the functional locus at 16q13.	5	0.020307	
Pubmed	9398194~Kinetics of ferric cytochrome P450 reduction by NADPH-cytochrome P450 reductase: rapid reduction in the absence of substrate and variations among cytochrome P450 systems.	4	0.025289	
Pubmed	11171287~Common genetic variants that relate to disorders of lipid transport in Spanish subjects with premature coronary artery disease.	4	0.025289	
Pubmed	12964943~Genetic study of common variants at the Apo E, Apo AI, Apo CIII, Apo B, lipoprotein lipase (LPL) and hepatic lipase (LIPC) genes and coronary artery disease (CAD): variation in LIPC gene associates with clinical outcomes in patients with established CAD.	4	0.025289	
Pubmed	3175763~Cloning and chromosomal location of human genes inducible by type I interferon.	4	0.025289	
Pubmed	18231117~Pharmacokinetics/genotype associations for major cytochrome P450 enzymes in native and first- and third-generation Japanese populations: comparison with Korean, Chinese, and	4	0.025289	

	Caucasian populations.			
Pubmed	11991719~Cloning and characterization of a novel apolipoprotein A-I binding protein, AI-BP, secreted by cells of the kidney proximal tubules in response to HDL or ApoA-I.	4	0.0252 89	
Pubmed	12871600~Genetic risk factors for cerebrovascular disease in children with sickle cell disease: design of a case-control association study and genomewide screen.	6	0.0273 55	
Pubmed	19015224~Polymorphisms in C2, CFB and C3 are associated with progression to Advanced Age-Related Macular Degeneration associated with visual loss.	5	0.0325 53	
Pubmed	18513389~New application of intelligent agents in sporadic amyotrophic lateral sclerosis identifies unexpected specific genetic background.	7	0.0335 63	
Pubmed	18258609~A comprehensive analysis of phase I and phase II metabolism gene polymorphisms and risk of non-small cell lung cancer in smokers.	5	0.0499 54	
Pubmed	10836148~Human UDP-glucuronosyltransferases: metabolism, expression, and disease.	5	0.0499 54	
Disease	D006523~Hepatitis, Toxic	11	0	CTD
Disease	D007105~Immune Complex Diseases	4	0.0258 91	CTD
Disease	105200~AMYLOIDOSIS, FAMILIAL VISCERAL	3	0.0389 81	OMIM
Disease	D016871~Pasteurellaceae Infections	3	0.0389 81	CTD

Table S4: Enrichment of UFF selected genes for the TCGA glioblastome multiforme datasets (DAVID and toppgene tools).

Platform abbr: Agil=Agilent, Affy=Affymetrix

Category	Term	Count	PValue	Bonferroni	Benjamini	Platform
GOTERM_BP_ALL	GO:0007275~multicellular organismal development	170	4.55E-23	2.39E-19	2.39E-19	Agil+Affy

GOTERM_BP_ALL	GO:0032502~developmental process	198	2.68E-18	1.41E-14	7.03E-15	Agil+Affy
GOTERM_BP_ALL	GO:0032501~multicellular organismal process	218	3.62E-18	1.90E-14	6.33E-15	Agil+Affy
GOTERM_BP_ALL	GO:0048731~system development	126	5.70E-16	2.92E-12	7.29E-13	Agil+Affy
GOTERM_BP_ALL	GO:0048856~anatomical structure development	144	7.34E-16	4.08E-12	8.17E-13	Agil+Affy
GOTERM_BP_ALL	GO:0048513~organ development	88	4.18E-10	2.20E-06	3.66E-07	Agil+Affy
GOTERM_BP_ALL	GO:0022610~biological adhesion	62	9.75E-10	5.12E-06	7.32E-07	Agil
GOTERM_BP_ALL	GO:0007155~cell adhesion	62	9.75E-10	5.12E-06	7.32E-07	Agil
GOTERM_BP_ALL	GO:0008283~cell proliferation	24	3.79E-08	1.99E-04	3.32E-05	Affy
GOTERM_BP_ALL	GO:0009605~response to external stimulus	50	1.45E-07	7.62E-04	8.47E-05	Agil
GOTERM_BP_ALL	GO:0007399~nervous system development	56	2.96E-07	0.001554386	1.56E-04	Agil
GOTERM_BP_ALL	GO:0007267~cell-cell signaling	50	3.18E-07	0.001669944	1.52E-04	Agil
GOTERM_BP_ALL	GO:0007626~locomotory behavior	24	3.26E-07	0.001709565	1.43E-04	Agil
GOTERM_BP_ALL	GO:0007610~behavior	32	3.46E-07	0.00181747	1.40E-04	Agil
GOTERM_BP_ALL	GO:0042127~regulation of cell proliferation	17	1.49E-06	0.007808287	9.79E-04	Affy
GOTERM_BP_ALL	GO:0042330~taxis	19	3.54E-06	0.018431252	0.0013279	Agil
GOTERM_BP_ALL	GO:0006935~chemotaxis	19	3.54E-06	0.018431252	0.0013279	Agil
GOTERM_BP_ALL	GO:0006954~inflammatory response	13	3.77E-06	0.019609965	0.0021981	Affy
GOTERM_BP_ALL	GO:0009653~anatomical structure morphogenesis	69	3.97E-06	0.020639256	0.0013026	Agil
GOTERM_BP_ALL	GO:0051179~localization	47	4.42E-06	0.022977252	0.0023218	Affy
GOTERM_BP_ALL	GO:0051234~establishment of localization	43	6.12E-06	0.031638499	0.0029184	Affy
GOTERM_BP_ALL	GO:0048468~cell development	26	8.07E-06	0.041504794	0.0035263	Affy
GOTERM_CC_ALL	GO:0005576~extracellular region	48	8.78E-20	7.62E-17	7.62E-17	Agil+Affy
GOTERM_CC_ALL	GO:0044421~extracellular region part	83	7.06E-18	6.13E-15	6.13E-15	Agil+Affy
GOTERM_CC_ALL	GO:0005615~extracellular space	24	1.12E-11	9.69E-09	3.23E-09	Agil+Affy
GOTERM_CC_ALL	GO:0005578~proteinaceous extracellular matrix	41	4.31E-11	3.74E-08	1.25E-08	Agil
GOTERM_CC_ALL	GO:0031012~extracellular matrix	41	7.33E-11	6.37E-08	1.59E-08	Agil+Affy
GOTERM_CC_ALL	GO:0044420~extracellular matrix part	16	1.26E-05	0.010907118	0.0018262	Agil
GOTERM_CC_ALL	GO:0005578~proteinaceous extracellular matrix	13	1.49E-05	0.012864514	0.0032318	Affy
GOTERM_CC_ALL	GO:0005883~neurofilament	5	5.12E-05	0.043496834	0.0063329	Agil
GOTERM_CC_ALL	GO:0060053~neurofilament	5	5.12E-05	0.0434968	0.006332	Agil

	cytoskeleton			34	9	
GOTERM_MF_ALL	GO:0005515~protein binding	313	5.31E-09	1.53E-05	1.53E-05	Agil+Affy
GOTERM_MF_ALL	GO:0004857~enzyme inhibitor activity	15	1.26E-08	3.63E-05	3.63E-05	Affy
GOTERM_MF_ALL	GO:0005102~receptor binding	60	2.51E-08	7.22E-05	3.61E-05	Agil+Affy
GOTERM_MF_ALL	GO:0005488~binding	495	2.98E-08	8.58E-05	2.86E-05	Agil
GOTERM_MF_ALL	GO:0030234~enzyme regulator activity	20	6.16E-06	0.017581109	0.0044246	Affy
INTERPRO	IPR001827:Homeobox protein, antennapedia type	9	6.85E-07	0.004048925	0.0040489	Agil
INTERPRO	IPR001356:Homeobox	23	6.17E-06	0.035883842	0.0181058	Agil
SMART	SM00389:HOX	23	6.22E-05	0.037046171	0.0370462	Agil
SP_PIR_KEYWORDS	signal	207	2.00E-25	2.13E-22	2.13E-22	Agil+Affy
SP_PIR_KEYWORDS	Secreted	124	4.48E-21	4.77E-18	2.39E-18	Agil+Affy
SP_PIR_KEYWORDS	Direct protein sequencing	63	6.05E-21	6.45E-18	6.45E-18	Affy
SP_PIR_KEYWORDS	glycoprotein	217	8.07E-15	8.64E-12	2.88E-12	Agil
SP_PIR_KEYWORDS	Developmental protein	57	1.07E-10	1.14E-07	2.85E-08	Agil
SP_PIR_KEYWORDS	plasma	11	1.21E-09	1.29E-06	3.21E-07	Affy
SP_PIR_KEYWORDS	extracellular matrix	26	2.54E-07	2.71E-04	5.42E-05	Agil
SP_PIR_KEYWORDS	cell adhesion	36	3.18E-07	3.39E-04	5.64E-05	Agil
SP_PIR_KEYWORDS	glycoprotein	52	1.30E-06	0.001385179	2.77E-04	Affy
SP_PIR_KEYWORDS	pyroglutamic acid	7	1.47E-06	0.001562749	2.61E-04	Affy
SP_PIR_KEYWORDS	acetylation	17	5.91E-06	0.006278322	8.99E-04	Affy
SP_PIR_KEYWORDS	calcium	51	6.04E-06	0.006416667	9.19E-04	Agil
SP_PIR_KEYWORDS	Homeobox	23	2.50E-05	0.026247351	0.0033192	Agil
SP_PIR_KEYWORDS	Cleavage on pair of basic residues	25	3.13E-05	0.032860285	0.0037056	Agil
SP_PIR_KEYWORDS	heparin-binding	11	4.15E-05	0.043311563	0.004418	Agil
SP_PIR_KEYWORDS	disease mutation	25	4.67E-05	0.048560317	0.0062031	Affy
UP_SEQ_FEATURE	signal peptide	182	4.89E-17	9.23E-13	9.23E-13	Agil+Affy
UP_SEQ_FEATURE	disulfide bond	138	1.45E-06	0.027045799	0.0136156	Agil
UP_SEQ_FEATURE	glycosylation site:N-linked (GlcNAc...)	176	2.34E-06	0.043139842	0.0145918	Agil

**Category(Toppgene tool)**	**ID**	**Hit Count in Query**	**P-value**	**Source**	**Platform**

		List			
GO: Biological Process	GO:0009611~response to wounding	23	0.00E+00		Affy
GO: Biological Process	GO:0001501~skeletal system development	4.50E+01	0.00E+00		Agil
GO: Biological Process	GO:0007389~pattern specification process	3.60E+01	0.00E+00		Agil
GO: Biological Process	GO:0048705~skeletal system morphogenesis	20	2.60E-05		Agil
GO: Biological Process	GO:0009887~organ morphogenesis	69	5.30E-05		Agil
GO: Biological Process	GO:0048706~embryonic skeletal system development	16	7.40E-05		Agil
GO: Biological Process	GO:0048598~embryonic morphogenesis	33	4.51E-04		Agil
GO: Biological Process	GO:0048704~embryonic skeletal system morphogenesis	13	5.17E-04		Agil
GO: Biological Process	GO:0046394~carboxylic acid biosynthetic process	9	6.80E-04		Affy
GO: Biological Process	GO:0016053~organic acid biosynthetic process	9	6.80E-04		Affy
GO: Biological Process	GO:0006631~fatty acid metabolic process	12	1.02E-03		Affy
GO: Biological Process	GO:0003002~regionalizatio n	25	1.42E-03		Agil
GO: Biological Process	GO:0030198~extracellular matrix organization	17	1.93E-03		Agil+Affy
GO: Biological Process	GO:0006952~defense response	20	1.98E-03		Affy
GO: Biological Process	GO:0048583~regulation of response to stimulus	15	2.23E-03		Affy
GO: Biological Process	GO:0009790~embryonic development	49	2.53E-03		Agil
GO: Biological Process	GO:0006633~fatty acid biosynthetic process	8	2.65E-03		Affy
GO: Biological Process	GO:0043062~extracellular structure organization	22	3.54E-03		Agil+Affy
GO: Biological Process	GO:0032101~regulation of response to external stimulus	9	5.48E-03		Affy
GO: Biological Process	GO:0050793~regulation of developmental process	29	6.77E-03		Affy
GO: Biological Process	GO:0019216~regulation of lipid metabolic process	7	1.03E-02		Affy
GO: Biological Process	GO:0019217~regulation of fatty acid metabolic process	5	1.55E-02		Affy
GO: Biological Process	GO:0032787~monocarboxy lic acid metabolic process	12	2.04E-02		Affy
GO: Biological Process	GO:0010810~regulation of cell-substrate adhesion	10	2.25E-02		Agil
GO: Biological Process	GO:0051239~regulation of multicellular organismal	21	2.46E-02		Affy

	process				
GO: Biological Process	GO:0034369~plasma lipoprotein particle remodeling	4	3.55E-02		Affy
GO: Biological Process	GO:0034368~protein-lipid complex remodeling	4	3.55E-02		Affy
GO: Biological Process	GO:0034367~macromolecular complex remodeling	4	3.55E-02		Affy
GO: Biological Process	GO:0002526~acute inflammatory response	7	3.57E-02		Affy
GO: Biological Process	GO:0019752~carboxylic acid metabolic process	16	4.05E-02		Affy
GO: Biological Process	GO:0006082~organic acid metabolic process	16	4.41E-02		Affy
GO: Biological Process	GO:0009954~proximal/distal pattern formation	7.00E+00	4.87E-02		Agil
GO: Cellular Component	GO:0005625~soluble fraction	22	2.72E-02		Agil
GO: Cellular Component	GO:0042613~MHC class II protein complex	3	3.47E-02		Affy
GO: Cellular Component	GO:0042611~MHC protein complex	4	4.78E-02		Affy
GO: Molecular Function	GO:0030247~polysaccharide binding	1.90E+01	6.50E-04		Agil
GO: Molecular Function	GO:0008201~heparin binding	1.60E+01	7.05E-04		Agil
GO: Molecular Function	GO:0030246~carbohydrate binding	3.20E+01	7.91E-04		Agil
GO: Molecular Function	GO:0004866~endopeptidase inhibitor activity	9	1.06E-03		Affy
GO: Molecular Function	GO:0030414~peptidase inhibitor activity	9	1.32E-03		Affy
GO: Molecular Function	GO:0005539~glycosaminoglycan binding	1.80E+01	1.61E-03		Agil
GO: Molecular Function	GO:0008289~lipid binding	15	2.96E-03		Affy
GO: Molecular Function	GO:0001871~pattern binding	1.90E+01	3.79E-03		Agil
GO: Molecular Function	GO:0008199~ferric iron binding	3	1.38E-02		Affy
GO: Molecular Function	GO:0032395~MHC class II receptor activity	3	1.96E-02		Affy
GO: Molecular Function	GO:0005184~neuropeptide hormone activity	7.00E+00	3.19E-02		Agil
GO: Molecular Function	GO:0004859~phospholipase inhibitor activity	3	3.55E-02		Affy
Pathway	h_mhcPathway~Antigen Processing and Presentation	3	4.07E-02	CGAP BioCarta	Affy
Pubmed	16335952~Human plasma N-glycoproteome analysis by immunoaffinity subtraction, hydrazide chemistry, and mass spectrometry.	17	0.00E+00		Affy
Pubmed	11857506~Complete mutation analysis panel of	1.40E+01	0.00E+00		Agil

	the 39 human HOX genes.				
Pubmed	1358459~Vertebrate homeobox gene nomenclature.	1.30E+01	1.00E-06		Agil
Pubmed	1973146~Nomenclature for human homeobox genes.	13	4.00E-06		Agil
Pubmed	8646877~Fine mapping of human HOX gene clusters.	10	3.20E-05		Agil
Pubmed	14718574~The human plasma proteome: a nonredundant list developed by combination of four separate sources.	11	7.30E-05		Affy
Pubmed	2574852~The human HOX gene family.	10	1.12E-04		Agil
Pubmed	1602151~Treatment of Haemophilus aphrophilus endocarditis with ciprofloxacin.	9	1.38E-04		Affy
Pubmed	16199891~Comparative proteomic analysis of intra- and interindividual variation in human cerebrospinal fluid.	5	7.33E-04		Affy
Pubmed	18818748~Preterm birth in Caucasians is associated with coagulation and inflammation pathway gene variants.	9	1.10E-03		Affy
Pubmed	2576652~Organization of human class I homeobox genes.	8	2.47E-03		Agil
Pubmed	6955026~Membrane insertion and oligomeric assembly of HLA-DR histocompatibility antigens.	3	2.55E-03		Affy
Pubmed	7519244~Mapping functional regions in the lumenal domain of the class II-associated invariant chain.	3	2.55E-03		Affy
Pubmed	16530434~Interaction of human neutrophils with endothelial cells regulates the expression of endogenous proteins annexin 1, galectin-1 and galectin-3.	3	2.55E-03		Affy
Pubmed	8316295~Three-dimensional structure of the human class II histocompatibility antigen HLA-DR1.	3	2.55E-03		Affy
Pubmed	14760718~Screening for N-glycosylated proteins by liquid chromatography mass spectrometry.	6	4.71E-03		Affy

Pubmed	15121303~Associations between human leukocyte antigen (HLA) alleles and very high levels of measles antibody following vaccination.	3	1.02E-02		Affy
Pubmed	11749962~Transferrin binds insulin-like growth factors and affects binding properties of insulin-like growth factor binding protein-3.	3	1.02E-02		Affy
Pubmed	18939942~Association of Smoking Behavior with an Odorant Receptor Allele Telomeric to the Human Major Histocompatibility Complex.	3	1.02E-02		Affy
Pubmed	9192850~Transcript mapping in a 46-kb sequenced region at the core of 12q13.3 amplification in human cancers.	3	1.02E-02		Affy
Pubmed	11927549~Regulation of intracellular trafficking of human CD1d by association with MHC class II molecules.	3	1.02E-02		Affy
Pubmed	17662350~Cerebral cavernomas and human leukocyte antigens: preliminary clinical results.	3	1.02E-02		Affy
Pubmed	12373032~Evidence for human leukocyte antigen-related susceptibility in idiopathic childhood ischemic stroke.	3	1.02E-02		Affy
Pubmed	15688398~Tumor HLA-DR expression linked to early intrahepatic recurrence of hepatocellular carcinoma.	3	1.02E-02		Affy
Pubmed	16289162~The identification of myocilin-associated proteins in the human trabecular meshwork.	5	1.15E-02		Affy
Pubmed	12975309~The secreted protein discovery initiative (SPDI), a large-scale effort to identify novel human secreted and transmembrane proteins: a bioinformatics assessment.	58	1.29E-02		Agil
Pubmed	7759097~Isolation of a YAC clone covering a cluster of nine S100 genes on human chromosome 1q21: rationale for a new nomenclature of the S100	4	1.98E-02		Affy

	calcium-binding protein family.				
Pubmed	12082592~Multi-locus interactions predict risk for post-PTCA restenosis: an approach to the genetic analysis of common complex disease.	6	2.06E-02		Affy
Pubmed	18434090~Caveolin-1 increases basal and TGF-beta1-induced expression of type I procollagen through PI-3 kinase/Akt/mTOR pathway in human dermal fibroblasts.	3	2.53E-02		Affy
Pubmed	18636124~Polymorphisms in the estrogen receptor 1 and vitamin C and matrix metalloproteinase gene families are associated with susceptibility to lymphoma.	8	3.11E-02		Affy
Pubmed	18987644~Genetic variants of the HLA-A, HLA-B and AIF1 loci show independent associations with type 1 diabetes in Norwegian families.	4	4.38E-02		Affy

Table S5: Enrichment of UFF selected genes for the TCGA ovarian serous cystadenocarcinoma datasets (DAVID and toppgene tools).

Platform abbr: Agil=Agilent, Affy=Affymetrix

Category (DAVID tool)	Term	Count	PValue	Bonferroni	Benjamini	Platform
GOTERM_BP_ALL	GO:0007275~multicellular organismal development	97	1.15E-12	6.06E-09	6.06E-09	Agil
GOTERM_BP_ALL	GO:0007267~cell-cell signaling	43	3.09E-11	1.63E-07	8.13E-08	Agil
GOTERM_BP_ALL	GO:0032502~developmental process	116	5.02E-11	2.64E-07	8.79E-08	Agil
GOTERM_BP_ALL	GO:0032501~multicellular organismal process	157	2.06E-10	1.04E-06	1.04E-06	Affy
GOTERM_BP_ALL	GO:0048856~anatomical structure development	86	2.35E-10	1.24E-06	3.09E-07	Agil
GOTERM_BP_ALL	GO:0032501~multicellular organismal process	125	4.87E-10	2.56E-06	5.12E-07	Agil
GOTERM_BP_ALL	GO:0048731~system development	74	7.89E-10	4.15E-06	6.91E-07	Agil
GOTERM_BP_ALL	GO:0006952~defense response	46	2.27E-09	1.15E-05	5.77E-06	Affy
GOTERM_BP_ALL	GO:0032502~developmental process	145	3.00E-08	1.52E-04	5.08E-05	Affy
GOTERM_BP_ALL	GO:0007399~nervous	41	4.48E-08	2.36E-04	3.36E-05	Agil

	system development					
GOTERM_BP_ALL	GO:0007275~multicellular organismal development	112	7.42E-08	3.77E-04	9.42E-05	Affy
GOTERM_BP_ALL	GO:0008283~cell proliferation	40	2.20E-07	0.0011575 42	1.45E-04	Agil
GOTERM_BP_ALL	GO:0009605~response to external stimulus	46	2.37E-07	0.0012021 04	2.41E-04	Affy
GOTERM_BP_ALL	GO:0009611~response to wounding	36	2.49E-07	0.0012618 98	2.10E-04	Affy
GOTERM_BP_ALL	GO:0006954~inflammatory response	28	1.10E-06	0.0055913 52	8.01E-04	Affy
GOTERM_BP_ALL	GO:0006955~immune response	42	6.30E-06	0.0315127 47	0.003994 5	Affy
GOTERM_BP_ALL	GO:0048856~anatomical structure development	100	6.63E-06	0.0331102 82	0.003734 2	Affy
GOTERM_BP_ALL	GO:0019226~transmission of nerve impulse	22	7.58E-06	0.0390304 1	0.004413 8	Agil
GOTERM_CC_ALL	GO:0005576~extracellular region	122	4.32E-37	3.61E-34	3.61E-34	Affy
GOTERM_CC_ALL	GO:0044421~extracellular region part	92	5.60E-30	4.68E-27	2.34E-27	Affy
GOTERM_CC_ALL	GO:0005615~extracellular space	59	9.39E-18	7.85E-15	2.62E-15	Affy
GOTERM_CC_ALL	GO:0031012~extracellular matrix	43	2.48E-16	1.86E-13	4.64E-14	Affy
GOTERM_CC_ALL	GO:0005578~proteinaceou s extracellular matrix	42	8.13E-16	6.50E-13	1.30E-13	Affy
GOTERM_CC_ALL	GO:0005576~extracellular region	74	1.22E-14	1.06E-11	1.06E-11	Agil
GOTERM_CC_ALL	GO:0044421~extracellular region part	54	1.02E-13	8.89E-11	4.45E-11	Agil
GOTERM_CC_ALL	GO:0005615~extracellular space	33	4.03E-08	3.50E-05	1.17E-05	Agil
GOTERM_CC_ALL	GO:0005578~proteinaceou s extracellular matrix	25	2.18E-07	1.90E-04	4.74E-05	Agil
GOTERM_CC_ALL	GO:0031012~extracellular matrix	25	3.00E-07	2.60E-04	5.21E-05	Agil
GOTERM_MF_ALL	GO:0008083~growth factor activity	19	8.01E-08	2.31E-04	2.31E-04	Agil
GOTERM_MF_ALL	GO:0005102~receptor binding	39	6.14E-07	0.0017641 32	8.82E-04	Agil
GOTERM_MF_ALL	GO:0001871~pattern binding	15	7.82E-06	0.0213706 12	0.021370 6	Affy
GOTERM_MF_ALL	GO:0005201~extracellular matrix structural constituent	13	1.47E-05	0.0399046 08	0.020155 4	Affy
GOTERM_MF_ALL	GO:0030247~polysacchari de binding	14	1.55E-05	0.0418090 22	0.014135 2	Affy
SMART	SM00041:CT	7	1.74E-05	0.0103080 07	0.010308	Affy
SP_PIR_KEYWOR DS	signal	175	2.37E-33	2.49E-30	2.49E-30	Agil+Affy
SP_PIR_KEYWOR DS	Secreted	118	5.40E-33	5.68E-30	2.84E-30	Agil+Affy
SP_PIR_KEYWOR DS	glycoprotein	171	1.93E-19	2.02E-16	6.75E-17	Agil+Affy
SP_PIR_KEYWOR DS	Direct protein sequencing	128	8.31E-13	8.73E-10	2.18E-10	Affy
SP_PIR_KEYWOR	extracellular matrix	26	1.04E-09	1.09E-06	2.19E-07	Affy

DS						
SP_PIR_KEYWORDS	growth factor	17	4.15E-08	4.42E-05	1.11E-05	Agil
SP_PIR_KEYWORDS	Cleavage on pair of basic residues	20	3.05E-06	0.003243731	6.50E-04	Agil
SP_PIR_KEYWORDS	triple helix	9	3.78E-06	0.003969574	6.63E-04	Affy
SP_PIR_KEYWORDS	hydroxylysine	9	5.01E-06	0.005250579	7.52E-04	Affy
SP_PIR_KEYWORDS	inflammation	8	5.42E-06	0.005683923	7.12E-04	Affy
SP_PIR_KEYWORDS	neuropeptide	8	5.70E-06	0.006056517	0.001012	Agil
SP_PIR_KEYWORDS	hydroxyproline	9	8.48E-06	0.008873406	9.90E-04	Affy
SP_PIR_KEYWORDS	inflammatory response	11	2.59E-05	0.026816353	0.0027146	Affy
SP_PIR_KEYWORDS	collagen	11	4.04E-05	0.04159311	0.0038546	Affy
SP_PIR_KEYWORDS	cell adhesion	22	4.75E-05	0.049386232	0.0072092	Agil
UP_SEQ_FEATURE	signal peptide	156	1.99E-25	3.13E-21	3.13E-21	Agil+Affy
UP_SEQ_FEATURE	disulfide bond	116	3.04E-13	4.79E-09	2.39E-09	Affy
UP_SEQ_FEATURE	glycosylation site:N-linked (GlcNAc...)	136	4.05E-10	6.37E-06	2.12E-06	Affy

**Category (Toppgene tool)**	**Term**	**Hit Count in Query List**	**P-value**	**Source**	**Platform**
Disease	D013274~Stomach Neoplasms	32	0.0000	CTD	Affy
Disease	603932~INTERVERTEBRAL DISC DISEASE; IDD	7	0.0001	OMIM	Affy
Disease	D001249~Asthma	13	0.0084	CTD	Affy
Disease	D001943~Breast Neoplasms	14	0.0371	CTD	Agil
GO: Biological Process	GO:0009887~organ morphogenesis	124	0.0000		Agil+Affy
GO: Biological Process	GO:0022610~biological adhesion	104	0.0000		Agil+Affy
GO: Biological Process	GO:0007155~cell adhesion	104	0.0000		Agil+Affy
GO: Biological Process	GO:0001501~skeletal system development	56	0.0000		Agil+Affy
GO: Biological Process	GO:0009888~tissue development	106	0.0000		Affy
GO: Biological Process	GO:0002376~immune system process	132	0.0000		Affy
GO: Biological Process	GO:0040011~locomotion	77	0.0000		Affy
GO: Biological Process	GO:0051674~localization of cell	80	0.0000		Affy
GO: Biological Process	GO:0006928~cell motion	80	0.0000		Affy
GO: Biological Process	GO:0006935~chemotaxis	37	0.0000		Affy

GO: Biological Process	GO:0042330~taxis	37	0.0000		Affy
GO: Biological Process	GO:0042221~response to chemical stimulus	114	0.0000		Affy
GO: Biological Process	GO:0042127~regulation of cell proliferation	88	0.0000		Affy
GO: Biological Process	GO:0051239~regulation of multicellular organismal process	98	0.0000		Affy
GO: Biological Process	GO:0007610~behavior	65	0.0000		Affy
GO: Biological Process	GO:0050793~regulation of developmental process	134	0.0000		Affy
GO: Biological Process	GO:0016477~cell migration	54	0.0000		Affy
GO: Biological Process	GO:0007626~locomotory behavior	46	0.0000		Affy
GO: Biological Process	GO:0008544~epidermis development	33	0.0000		Affy
GO: Biological Process	GO:0032989~cellular component morphogenesis	83	0.0000		Affy
GO: Biological Process	GO:0007398~ectoderm development	34	0.0000		Affy
GO: Biological Process	GO:0048870~cell motility	56	0.0000		Affy
GO: Biological Process	GO:0000902~cell morphogenesis	80	0.0001		Affy
GO: Biological Process	GO:0032963~collagen metabolic process	13	0.0002		Affy
GO: Biological Process	GO:0044236~multicellular organismal metabolic process	15	0.0002		Affy
GO: Biological Process	GO:0048729~tissue morphogenesis	41	0.0003		Affy
GO: Biological Process	GO:0051707~response to other organism	40	0.0003		Affy
GO: Biological Process	GO:0009790~embryonic development	70	0.0004		Affy
GO: Biological Process	GO:0006959~humoral immune response	20	0.0004		Affy
GO: Biological Process	GO:0002504~antigen processing and presentation of peptide or polysaccharide antigen via MHC class II	10	0.0005		Affy
GO: Biological Process	GO:0044259~multicellular organismal macromolecule metabolic process	13	0.0005		Affy
GO: Biological Process	GO:0019886~antigen processing and presentation of exogenous peptide antigen via MHC class II	8	0.0009		Affy
GO: Biological Process	GO:0002495~antigen processing and presentation of peptide antigen via MHC class II	8	0.0009		Affy
GO: Biological Process	GO:0048598~embryonic morphogenesis	43	0.0010		Affy

GO: Biological Process	GO:0030198~extracellular matrix organization	22	0.0011		Affy
GO: Biological Process	GO:0022603~regulation of anatomical structure morphogenesis	51	0.0017		Affy
GO: Biological Process	GO:0043062~extracellular structure organization	29	0.0025		Affy
GO: Biological Process	GO:0002009~morphogenesis of an epithelium	26	0.0027		Affy
GO: Biological Process	GO:0060429~epithelium development	26	0.0027		Affy
GO: Biological Process	GO:0048583~regulation of response to stimulus	48	0.0027		Affy
GO: Biological Process	GO:0002526~acute inflammatory response	20	0.0028		Affy
GO: Biological Process	GO:0051704~multi-organism process	67	0.0035		Affy
GO: Biological Process	GO:0008015~blood circulation	32	0.0065		Affy
GO: Biological Process	GO:0003013~circulatory system process	32	0.0065		Affy
GO: Biological Process	GO:0008285~negative regulation of cell proliferation	43	0.0082		Affy
GO: Biological Process	GO:0000904~cell morphogenesis involved in differentiation	36	0.0105		Affy
GO: Biological Process	GO:0002478~antigen processing and presentation of exogenous peptide antigen	8	0.0108		Affy
GO: Biological Process	GO:0009615~response to virus	21	0.0111		Affy
GO: Biological Process	GO:0001944~vasculature development	38	0.0118		Affy
GO: Biological Process	GO:0009607~response to biotic stimulus	45	0.0130		Affy
GO: Biological Process	GO:0042098~T cell proliferation	18	0.0157		Affy
GO: Biological Process	GO:0007586~digestion	20	0.0178		Affy
GO: Biological Process	GO:0001568~blood vessel development	37	0.0182		Affy
GO: Biological Process	GO:0031175~neuron projection development	35	0.0193		Affy
GO: Biological Process	GO:0032101~regulation of response to external stimulus	23	0.0209		Affy
GO: Biological Process	GO:0032990~cell part morphogenesis	46	0.0209		Affy
GO: Biological Process	GO:0030030~cell projection organization	46	0.0209		Affy
GO: Biological Process	GO:0048858~cell projection morphogenesis	46	0.0209		Affy
GO: Biological Process	GO:0046651~lymphocyte proliferation	21	0.0221		Affy
GO: Biological Process	GO:0048584~positive regulation of response to	31	0.0263		Affy

	stimulus				
GO: Biological Process	GO:0030155~regulation of cell adhesion	17	0.0265		Agil
GO: Biological Process	GO:0048646~anatomical structure formation involved in morphogenesis	28	0.0279		Agil
GO: Biological Process	GO:0032943~mononuclear cell proliferation	21	0.0288		Affy
GO: Biological Process	GO:0045595~regulation of cell differentiation	49	0.0294		Affy
GO: Biological Process	GO:0048667~cell morphogenesis involved in neuron differentiation	31	0.0345		Affy
GO: Biological Process	GO:0030595~leukocyte chemotaxis	12	0.0358		Affy
GO: Biological Process	GO:0048514~blood vessel morphogenesis	23	0.0408		Agil
GO: Biological Process	GO:0007507~heart development	31	0.0448		Affy
GO: Biological Process	GO:0060326~cell chemotaxis	12	0.0452		Affy
GO: Cellular Component	GO:0044420~extracellular matrix part	30	0.0000		Agil+Affy
GO: Cellular Component	GO:0005581~collagen	14	0.0000		Affy
GO: Cellular Component	GO:0042613~MHC class II protein complex	8	0.0001		Affy
GO: Cellular Component	GO:0005887~integral to plasma membrane	112	0.0001		Affy
GO: Cellular Component	GO:0031226~intrinsic to plasma membrane	113	0.0001		Affy
GO: Cellular Component	GO:0031225~anchored to membrane	24	0.0002		Affy
GO: Cellular Component	GO:0043205~fibril	6	0.0008		Affy
GO: Cellular Component	GO:0001527~microfibril	5	0.0012		Affy
GO: Cellular Component	GO:0005583~fibrillar collagen	6	0.0077		Affy
GO: Cellular Component	GO:0030934~anchoring collagen	5	0.0211		Affy
GO: Molecular Function	GO:0005539~glycosamino glycan binding	31	0.0000		Agil+Affy
GO: Molecular Function	GO:0030246~carbohydrate binding	50	0.0000		Affy
GO: Molecular Function	GO:0008201~heparin binding	24	0.0000		Agil+Affy
GO: Molecular Function	GO:0008009~chemokine activity	16	0.0000		Affy
GO: Molecular Function	GO:0042379~chemokine receptor binding	16	0.0000		Affy
GO: Molecular Function	GO:0004857~enzyme inhibitor activity	41	0.0000		Affy
GO: Molecular Function	GO:0001664~G-protein-coupled receptor binding	21	0.0000		Agil+Affy
GO: Molecular Function	GO:0005509~calcium ion binding	93	0.0000		Affy

GO: Molecular Function	GO:0004866~endopeptidase inhibitor activity	26	0.0001		Affy
GO: Molecular Function	GO:0030414~peptidase inhibitor activity	26	0.0002		Affy
GO: Molecular Function	GO:0004867~serine-type endopeptidase inhibitor activity	19	0.0007		Affy
GO: Molecular Function	GO:0005125~cytokine activity	33	0.0028		Affy
GO: Molecular Function	GO:0005178~integrin binding	14	0.0030		Affy
GO: Molecular Function	GO:0032403~protein complex binding	23	0.0034		Affy
GO: Molecular Function	GO:0005520~insulin-like growth factor binding	9	0.0042		Affy
GO: Molecular Function	GO:0019838~growth factor binding	18	0.0113		Affy
GO: Molecular Function	GO:0008131~amine oxidase activity	4	0.0421		Affy
Human Phenotype	HP:0002758~Osteoarthritis	14	0.0000		Affy
Human Phenotype	HP:0001369~Arthritis	19	0.0068		Affy
Human Phenotype	HP:0002616~Aortic root dilatation	5	0.0253		Affy
Human Phenotype	HP:0001075~Atrophic scars	7	0.0268		Affy
Human Phenotype	HP:0000665~Lens dislocation	8	0.0373		Affy
Human Phenotype	HP:0001367~Abnormality of the joints	45	0.0490		Affy
Pathway	BREAST_CANCER_ESTROGEN_SIGNALING~BREAST_CANCER_ESTROGEN_SIGNALING	28	0.0000	MSigDB	Affy
Pathway	PW:0000238~insulin-like growth factor signaling	8	0.0002	Pathway Ontology	Affy
Pathway	hsa04512~ECM-receptor interaction	20	0.0008	KEGG pathway	Affy
Pathway	hsa04514~Cell adhesion molecules (CAMs)	26	0.0013	KEGG pathway	Affy
Pathway	hsa04610~Complement and coagulation cascades	16	0.0143	KEGG pathway	Affy
Pathway	h_fibrinolysisPathway~Fibrinolysis	6	0.0176	CGAP BioCarta	Affy
Pathway	140192~Xenobiotic metabolism	12	0.0182	Reactome	Agil
Pathway	hsa05310~Asthma	10	0.0201	KEGG pathway	Affy
Pathway	h_LairPathway~Cells and Molecules involved in local acute inflammatory response	7	0.0485	CGAP BioCarta	Affy
Pubmed	16335952~Human plasma N-glycoproteome analysis by immunoaffinity subtraction, hydrazide chemistry, and mass spectrometry.	49	0.0000		Affy

Pubmed	12539042~HIV-1 Tat reprograms immature dendritic cells to express chemoattractants for activated T cells and macrophages.	19	0.0000		Affy
Pubmed	18636124~Polymorphisms in the estrogen receptor 1 and vitamin C and matrix metalloproteinase gene families are associated with susceptibility to lymphoma.	28	0.0000		Affy
Pubmed	14718574~The human plasma proteome: a nonredundant list developed by combination of four separate sources.	30	0.0000		Affy
Pubmed	18818748~Preterm birth in Caucasians is associated with coagulation and inflammation pathway gene variants.	26	0.0000		Affy
Pubmed	16335952~Human plasma N-glycoproteome analysis by immunoaffinity subtraction, hydrazide chemistry, and mass spectrometry.	26	0.0001		Agil
Pubmed	16356191~Regulatory polymorphisms in extracellular matrix protease genes and susceptibility to rheumatoid arthritis: a case-control study.	7	0.0001		Affy
Pubmed	17703412~Genetic susceptibility to respiratory syncytial virus bronchiolitis is predominantly associated with innate immune genes.	30	0.0002		Affy
Pubmed	14760718~Screening for N-glycosylated proteins by liquid chromatography mass spectrometry.	14	0.0003		Affy
Pubmed	2576652~Organization of human class I homeobox genes.	10	0.0004		Affy
Pubmed	15944607~Genetic analysis of polymorphisms in biologically relevant candidate genes in patients with abdominal aortic aneurysms.	8	0.0005		Affy
Pubmed	17607721~Functional polymorphisms and haplotypes in the promoter of the MMP2 gene are associated with risk of nasopharyngeal carcinoma.	6	0.0018		Affy

Pubmed	15340161~Signal peptide prediction based on analysis of experimentally verified cleavage sites.	33	0.0018		Affy
Pubmed	7602119~Endocytosis of endogenously synthesized HIV-1 envelope protein. Mechanism and role in processing for association with class II MHC.	8	0.0023		Affy
Pubmed	8376762~HIV-1 envelope protein is expressed on the surface of infected cells before its processing and presentation to class II-restricted T lymphocytes.	8	0.0023		Affy
Pubmed	3489470~AIDS and related syndromes as a viral-induced autoimmune disease of the immune system: an anti-MHC II disorder. Therapeutic implications.	8	0.0023		Affy
Pubmed	10729169~Efficient incorporation of HLA class II onto human immunodeficiency virus type 1 requires envelope glycoprotein packaging.	8	0.0023		Affy
Pubmed	9079699~The enhanced immune response to the HIV gp160/LAMP chimeric gene product targeted to the lysosome membrane protein trafficking pathway.	8	0.0023		Affy
Pubmed	2543930~Identification of human CD4 residues affecting class II MHC versus HIV-1 gp120 binding.	8	0.0045		Affy
Pubmed	2846691~Inhibition of CD4+ T cell function by the HIV envelope protein, gp120.	8	0.0045		Affy
Pubmed	1978941~Identification and structural analysis of residues in the V1 region of CD4 involved in interaction with human immunodeficiency virus envelope glycoprotein gp120 and class II major histocompatibility complex molecules.	8	0.0045		Affy
Pubmed	18549475~Genome wide SNP comparative analysis between EGFR and KRAS mutated NSCLC and characterization of two models of oncogenic	5	0.0061		Affy

	cooperation in non-small cell lung carcinoma.				
Pubmed	10407151~Biosensor measurement of the interaction kinetics between insulin-like growth factors and their binding proteins.	6	0.0068		Affy
Pubmed	1973146~Nomenclature for human homeobox genes.	12	0.0070		Affy
Pubmed	12975309~The secreted protein discovery initiative (SPDI), a large-scale effort to identify novel human secreted and transmembrane proteins: a bioinformatics assessment.	53	0.0100		Agil
Pubmed	10661406~Tat competes with CIITA for the binding to P-TEFb and blocks the expression of MHC class II genes in HIV infection.	8	0.0142		Affy
Pubmed	11118314~Reciprocal modulation of transcriptional activities between HIV-1 Tat and MHC class II transactivator CIITA.	8	0.0142		Affy
Pubmed	11827988~Adapters in lymphocyte signaling.	9	0.0148		Affy
Pubmed	17388661~Exquisite sensitivity of TP53 mutant and basal breast cancers to a dose-dense epirubicin-cyclophosphamide regimen.	9	0.0148		Affy
Pubmed	11857506~Complete mutation analysis panel of the 39 human HOX genes.	11	0.0152		Affy
Pubmed	11390394~Kinetic investigation of chemokine truncation by CD26/dipeptidyl peptidase IV reveals a striking selectivity within the chemokine family.	6	0.0197		Affy
Pubmed	1358459~Vertebrate homeobox gene nomenclature.	11	0.0271		Affy
Pubmed	1869305~Interaction of CD4 with HLA class II antigens and HIV gp120.	7	0.0295		Affy
Pubmed	7512597~HLA class II antigens and the HIV envelope glycoprotein gp120 bind to the same face of CD4.	7	0.0295		Affy

Pubmed	18006768~Matrix metalloproteinase single-nucleotide polymorphisms and haplotypes predict breast cancer progression.	5	0.0355		Affy
Pubmed	17934860~Mcp-1, eNOS, tPA and PAI-1 gene polymorphism and correlation of genotypes and phenotypes in hepatopulmonary syndrome.	5	0.0355		Affy
Pubmed	12006706~Insulin-like growth factors and insulin-like growth factor binding proteins in adult patients with severe liver disease before and after orthotopic liver transplantation.	5	0.0355		Affy
Pubmed	15661050~Altered messenger RNA and protein expressions for insulin-like growth factor family members in clear cell and papillary renal cell carcinomas.	5	0.0355		Affy
Pubmed	15523498~Mutations in the known genes are not the major cause of MED; distinctive phenotypic entities among patients with no identified mutations.	5	0.0355		Affy
Pubmed	7541827~HIV gp120 inhibits T cell activation by interfering with expression of costimulatory molecules CD40 ligand and CD80 (B71).	8	0.0379		Affy
Pubmed	2446864~Characterization of binding properties of the myelin-associated glycoprotein to extracellular matrix constituents.	6	0.0474		Affy
