# Supplementary material for: UFFizi: a generic platform for ranking informative features
Source: BMC Bioinformatics. 2010 Jun 3;11:300. doi: 10.1186/1471-2105-11-300 (PMC2893168; doi:10.1186/1471-2105-11-300)
Supplement: Additional file 3 — Additional figures. Supp_figures.pdf: Comparison of UFF with other selection methods in terms of clustering results on the Melanoma, HIV and Hepatitis-C datasets. [file 1471-2105-11-300-S3.PDF]

Supplementary figures

UFFizi: A Generic Platform for Ranking Informative Features

Assaf Gottlieb[1], Roy Varshavsky[2], Michal Linial[3], David Horn[1]

[1] School of Physics and Astronomy, Tel Aviv University

[2] Israel Innovation Labs, Microsoft Israel R&D Center

[3] Department of Biological Chemistry, Institute of Life Sciences, The Hebrew University of Jerusalem

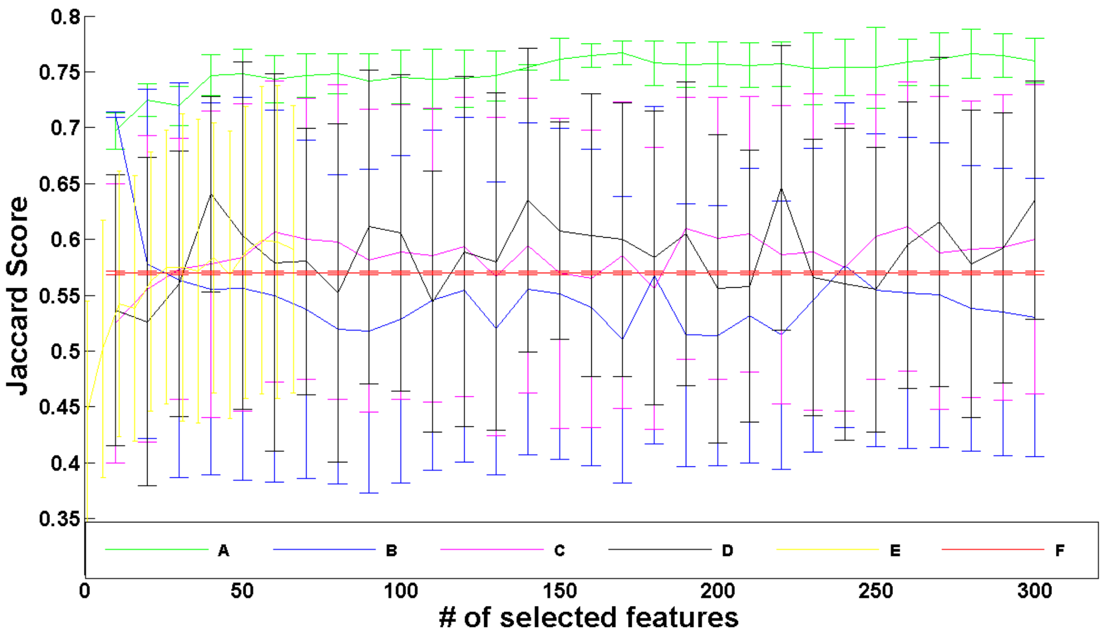

Figure S1 – Comparison of UFF with other selection methods on the Melanoma dataset.

Jaccard scores of clustering results for different selection methods on the melanoma dataset. Tested methods include (A) UFF, (B) Variance, (C) Feature entropy, (D) Random selection, (E) PCA feature extraction and (F) All features. Error bars denote standard deviation across different k-means runs.

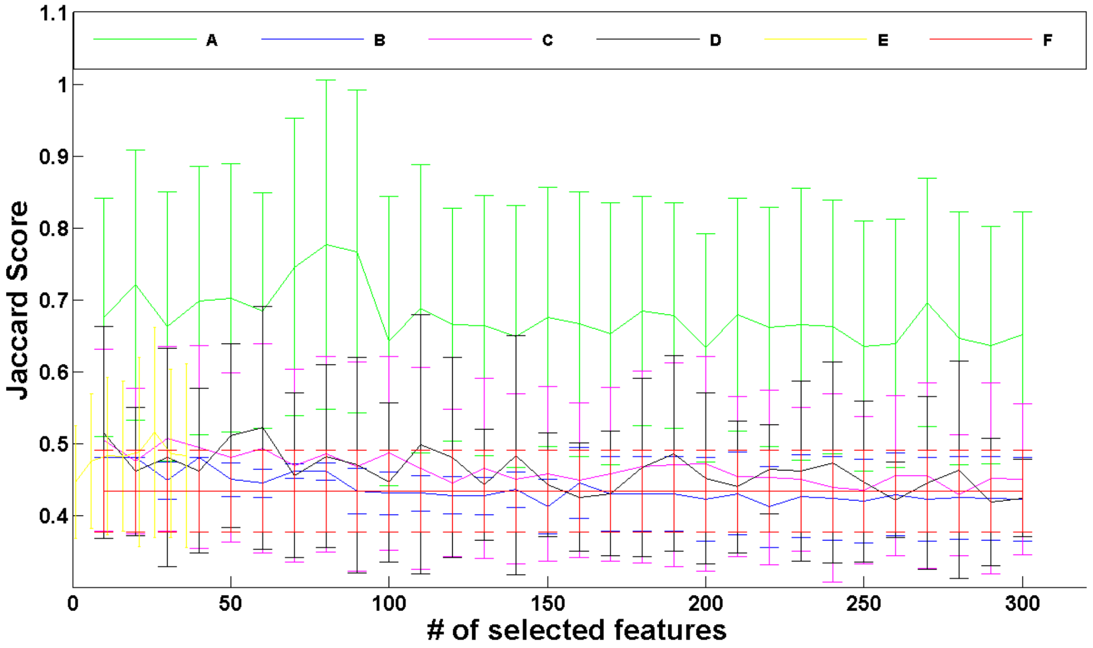

Figure S2 – Comparison of UFF with other selection methods on the HIV dataset. Jaccard scores of clustering results for different selection methods on the melanoma dataset. Tested methods include (A) UFF, (B) Variance, (C) Feature entropy, (D) Random selection, (E) PCA feature extraction and (F) All features. Error bars denote standard deviation across different *k*-means runs.

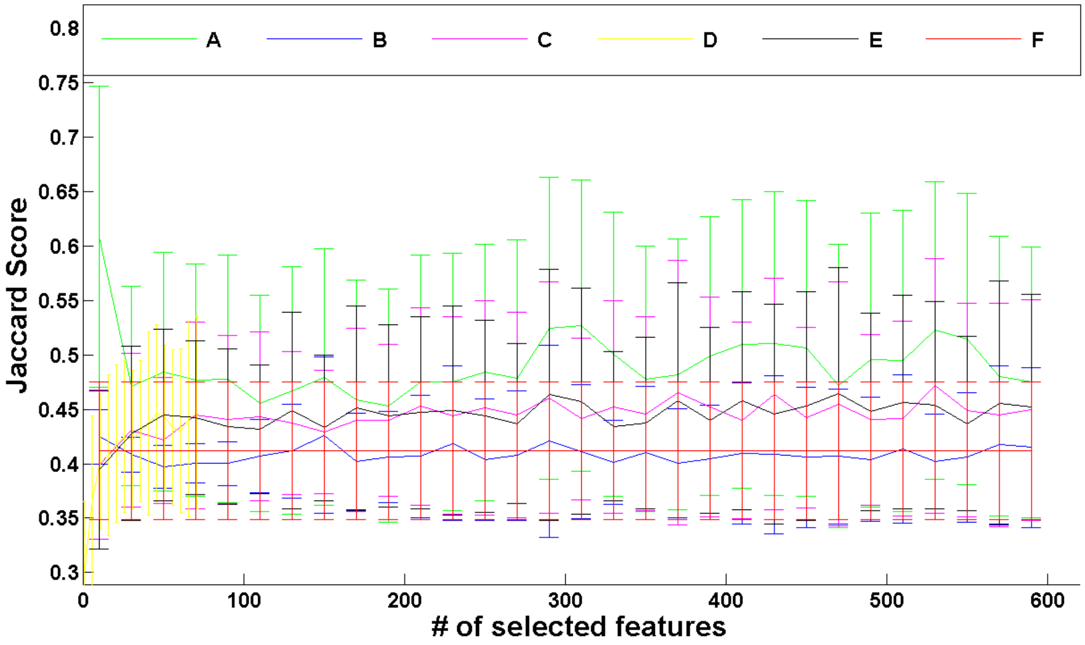

Figure S3 – Comparison of UFF with other selection methods on the Hepatitis-C dataset.

Jaccard scores of clustering results for different selection methods on the melanoma dataset. Tested methods include (A) UFF, (B) Variance, (C) Feature entropy, (D) Random selection, (E) PCA feature extraction and (F) All features. Error bars denote standard deviation across different *k*-means runs.
