# Supplementary material for: UFFizi: a generic platform for ranking informative features
Source: BMC Bioinformatics. 2010 Jun 3;11:300. doi: 10.1186/1471-2105-11-300 (PMC2893168; doi:10.1186/1471-2105-11-300)
Supplement: Additional file 4 — Clustering of various dataset instances using UFF selected genes. Clustering_results_tables.pdf: Clustering of the melanoma, Hepatitis-C, GBM and OV dataset instances using UFF selected genes. [file 1471-2105-11-300-S4.PDF]

Table S1: Clustering of the Melanoma dataset instances using UFF selected genes.
QC=Quantum Clustering algorithm.

Name	GEO Name	Type	cluster number (QC)	cluster number (*k*-means, *k*=3)
430MM	GSM71671	Normal skin	4	2
435MM	GSM71672	Normal skin	4	2
437MM	GSM71673	Normal skin	4	2
431MM	GSM71674	Normal skin	4	2
432MM	GSM71675	Normal skin	4	2
433MM	GSM71676	Normal skin	2	2
485MM	GSM71677	Normal skin	4	2
503MM	GSM71678	Benign skin nevi	2	2
504MM	GSM71679	Benign skin nevi	2	2
507MM	GSM71680	Benign skin nevi	2	1
508MM	GSM71681	Benign skin nevi	2	2
509MM	GSM71682	Benign skin nevi	2	2
495MM	GSM71683	Benign skin nevi	2	2
497MM	GSM71684	Benign skin nevi	2	1
500MM	GSM71685	Benign skin nevi	2	1
501MM	GSM71686	Benign skin nevi	2	2
502MM	GSM71687	Benign skin nevi	2	2
487MM	GSM71688	Benign skin nevi	2	2
489MM	GSM71689	Benign skin nevi	4	2
490MM	GSM71690	Benign skin nevi	2	2
491MM	GSM71691	Benign skin nevi	2	2
493MM	GSM71692	Benign skin nevi	4	2
496MM	GSM71693	Benign skin nevi	2	2
498MM	GSM71694	Benign skin nevi	4	2
499MM	GSM71695	Benign skin nevi	2	2
397MM	GSM71696	Primary malignant melanoma	1	3
440MM	GSM71697	Primary malignant melanoma	1	3
441MM	GSM71698	Primary malignant melanoma	1	3
459MM	GSM71699	Primary malignant melanoma	1	3
460MM	GSM71700	Primary malignant melanoma	1	3
464MM	GSM71701	Primary malignant melanoma	1	3
465MM	GSM71702	Primary malignant melanoma	1	3
466MM	GSM71703	Primary malignant melanoma	1	3
468MM	GSM71704	Primary malignant melanoma	1	3
469MM	GSM71705	Primary malignant melanoma	1	3
472MM	GSM71706	Primary malignant melanoma	1	3
475MM	GSM71707	Primary malignant melanoma	1	3
476MM	GSM71708	Primary malignant	1	3

		melanoma		
478MM	GSM71709	Primary malignant melanoma	1	1
480MM	GSM71710	Primary malignant melanoma	1	3
481MM	GSM71711	Primary malignant melanoma	1	3
511MM	GSM71712	Primary malignant melanoma	1	1
512MM	GSM71713	Primary malignant melanoma	1	3
392MM	GSM71714	Primary malignant melanoma	1	3
405MM	GSM71715	Primary malignant melanoma	1	3
407MM	GSM71716	Primary malignant melanoma	1	3
409MM	GSM71717	Primary malignant melanoma	1	3
442MM	GSM71718	Primary malignant melanoma	1	3
443MM	GSM71719	Primary malignant melanoma	1	3
444MM	GSM71720	Primary malignant melanoma	1	3
445MM	GSM71721	Primary malignant melanoma	1	3
446MM	GSM71722	Primary malignant melanoma	1	3
447MM	GSM71723	Primary malignant melanoma	1	3
448MM	GSM71724	Primary malignant melanoma	1	3
449MM	GSM71725	Primary malignant melanoma	1	1
450MM	GSM71726	Primary malignant melanoma	3	1
452MM	GSM71727	Primary malignant melanoma	3	1
453MM	GSM71728	Primary malignant melanoma	1	1
454MM	GSM71729	Primary malignant melanoma	3	1
455MM	GSM71730	Primary malignant melanoma	3	1
456MM	GSM71731	Primary malignant melanoma	1	3
457MM	GSM71732	Primary malignant melanoma	1	3
461MM	GSM71733	Primary malignant melanoma	1	3
463MM	GSM71734	Primary malignant melanoma	1	3
470MM	GSM71735	Primary malignant melanoma	1	3
473MM	GSM71736	Primary malignant melanoma	1	3
477MM	GSM71737	Primary malignant melanoma	1	3

482MM	GSM71738	Primary malignant melanoma	1	3
483MM	GSM71739	Primary malignant melanoma	1	2
484MM	GSM71740	Primary malignant melanoma	1	3

Table S2: Clustering of the Hepatitis-C dataset instances using UFF selected genes.

Name	GEO Accession	Type	cluster id QC using UFF selected genes	cluster id QC using all genes	cluster id *k*-means (*k*=4) using UFF selected genes	cluster id *k*-means (*k*=4) using all genes
LC2-BC14	GSM281890	Post-interferon blood sample	4	2	1	1
LC2-BJ23	GSM281914	Post-interferon blood sample	4	2	1	1
LC2-CE	GSM281856	Post-interferon blood sample	4	2	1	1
LC2-CH22	GSM281912	Post-interferon blood sample	4	2	1	1
LC2-EM	GSM281848	Post-interferon blood sample	4	5	1	2
LC2-GD	GSM281846	Post-interferon blood sample	4	5	1	2
LC2-GP	GSM281880	Post-interferon blood sample	4	2	1	1
LC2-HC	GSM281850	Post-interferon blood sample	4	2	1	1
LC2-HT20	GSM281908	Post-interferon blood sample	4	2	1	2
LC2-HW	GSM281886	Post-interferon blood sample	4	2	1	1
LC2-LP21	GSM281910	Post-interferon blood sample	4	2	1	2
LC2-PS	GSM281860	Post-interferon blood sample	4	2	1	2
LC2-RS	GSM281864	Post-interferon blood sample	4	2	1	1
LC2-SA	GSM281852	Post-interferon blood sample	4	2	1	1
LC2-SC	GSM281876	Post-interferon blood sample	4	2	1	2
LC2-SK15	GSM281894	Post-interferon blood sample	4	2	1	1
LC2-SZ13	GSM281906	Post-interferon blood sample	4	2	1	1
LC2-WE	GSM281868	Post-interferon blood sample	4	2	1	1
LC2-WV	GSM281872	Post-interferon blood sample	4	2	1	1
B2-BC14	GSM281888	Post-interferon liver biopsy	2	1	3	4
B2-BJ23	GSM281904	Post-interferon liver biopsy	2	1	3	4

B2-CE	GSM281854	Post-interferon liver biopsy	1	1	4	4
B2-CH22	GSM281902	Post-interferon liver biopsy	2	1	3	4
B2-EM	GSM281840	Post-interferon liver biopsy	1	1	4	4
B2-GD	GSM281838	Post-interferon liver biopsy	1	1	4	4
B2-GP	GSM281878	Post-interferon liver biopsy	2	1	3	4
B2-HC	GSM281842	Post-interferon liver biopsy	1	1	4	4
B2-HT20	GSM281898	Post-interferon liver biopsy	2	1	3	4
B2-HW	GSM281884	Post-interferon liver biopsy	2	1	3	4
B2-LP21	GSM281900	Post-interferon liver biopsy	2	1	3	4
B2-PS	GSM281858	Post-interferon liver biopsy	1	1	4	4
B2-RS	GSM281862	Post-interferon liver biopsy	1	1	4	4
B2-SA	GSM281844	Post-interferon liver biopsy	1	1	4	4
B2-SC	GSM281874	Post-interferon liver biopsy	1	1	4	4
B2-SK15	GSM281892	Post-interferon liver biopsy	2	1	3	4
B2-SZ13	GSM281896	Post-interferon liver biopsy	2	1	3	4
B2-WE	GSM281866	Post-interferon liver biopsy	1	1	4	4
B2-WV	GSM281870	Post-interferon liver biopsy	1	1	4	4
LC1-BC14	GSM281889	Pre-interferon blood sample	3	4	2	3
LC1-BJ23	GSM281913	Pre-interferon blood sample	3	4	2	3
LC1-CE	GSM281855	Pre-interferon blood sample	3	3	2	3
LC1-CH22	GSM281911	Pre-interferon blood sample	3	4	2	3
LC1-EM	GSM281847	Pre-interferon blood sample	3	3	2	3
LC1-GD	GSM281845	Pre-interferon blood sample	3	3	2	3
LC1-GP	GSM281879	Pre-interferon blood sample	3	4	2	3
LC1-HC	GSM281849	Pre-interferon blood sample	3	3	2	3
LC1-HT20	GSM281907	Pre-interferon blood sample	3	4	2	3
LC1-HW	GSM281885	Pre-interferon blood sample	3	4	2	3
LC1-LP21	GSM281909	Pre-interferon blood sample	3	4	2	3
LC1-PS	GSM281859	Pre-interferon blood sample	3	3	2	3
LC1-RS	GSM281863	Pre-interferon blood	3	3	2	3

		sample				
LC1-SA	GSM281851	Pre-interferon blood sample	3	3	2	3
LC1-SC	GSM281875	Pre-interferon blood sample	3	4	2	3
LC1-SK15	GSM281893	Pre-interferon blood sample	3	4	2	3
LC1-SZ13	GSM281905	Pre-interferon blood sample	3	4	2	3
LC1-WE	GSM281867	Pre-interferon blood sample	3	3	2	3
LC1-WV	GSM281871	Pre-interferon blood sample	3	3	2	3
B1-BC14	GSM281887	Pre-interferon liver biopsy	2	1	3	4
B1-BJ23	GSM281903	Pre-interferon liver biopsy	2	1	3	4
B1-CE	GSM281853	Pre-interferon liver biopsy	1	1	4	4
B1-CH22	GSM281901	Pre-interferon liver biopsy	2	1	3	4
B1-EM	GSM281839	Pre-interferon liver biopsy	1	1	4	4
B1-GD	GSM281837	Pre-interferon liver biopsy	1	1	4	4
B1-GP	GSM281877	Pre-interferon liver biopsy	2	1	3	4
B1-HC	GSM281841	Pre-interferon liver biopsy	1	1	4	4
B1-HT20	GSM281897	Pre-interferon liver biopsy	2	1	3	4
B1-HW	GSM281883	Pre-interferon liver biopsy	2	1	3	4
B1-LP21	GSM281899	Pre-interferon liver biopsy	2	1	3	4
B1-OR	GSM281882	Pre-interferon liver biopsy	1	1	4	4
B1-PS	GSM281857	Pre-interferon liver biopsy	1	1	4	4
B1-RS	GSM281861	Pre-interferon liver biopsy	1	1	4	4
B1-SA	GSM281843	Pre-interferon liver biopsy	1	1	4	4
B1-SC	GSM281873	Pre-interferon liver biopsy	2	1	3	4
B1-SK15	GSM281891	Pre-interferon liver biopsy	2	1	3	4
B1-SZ13	GSM281895	Pre-interferon liver biopsy	2	1	3	4
B1-TT	GSM281881	Pre-interferon liver biopsy	1	1	4	4
B1-WE	GSM281865	Pre-interferon liver biopsy	1	1	4	4
B1-WV	GSM281869	Pre-interferon liver biopsy	1	1	4	4

Table S3: Clustering of TCGA glioblastoma multiforme selected datasets (instances>30)

Platform	Agilent				Affymetrix					
**Dataset #**	**1.4.3**	**2.5.3**	**2.8.1**	**2.6.2**	**5.4**	**6.4**	**4.4**	**11.4**	**8.4**	**3.4**
**Instances #**	**54**	**64**	**39**	**35**	**64**	**46**	**39**	**39**	**38**	**34**
**clusters # (QC)**	**4**	**5**	**1**	**3**	**2**	**3**	**3**	**4**	**3**	**4**
**Instance ID**	**cluster**				**cluster**					
1	2	4	1	3	1	2	1	2	3	1
2	1	1	1	2	1	3	2	4	1	2
3	4	3	1	3	2	1	2	1	1	4
4	3	3	1	1	1	1	1	4	3	4
5	3	3	1	3	1	1	1	1	1	3
6	3	5	1	3	2	2	2	3	1	3
7	4	4	2	2	1	3	1	4	1	2
8	3	1	2	3	1	1	1	2	3	4
9	2	1	2	3	2	1	3	1	3	1
10	4	3	2	2	1	2	3	2	2	3
11	3	5	2	2	1	2	1	1	3	1
12	3	2	2	1	1	3	1	2	1	2
13	3	3	2	3	2	1	1	4	1	1
14	1	4	2	1	2	3	1	1	1	2
15	4	1	2	3	1	1	1	4	3	4
16	3	4	2	3	2	1	1	2	1	3
17	2	4	1	2	1	1	1	3	3	2
18	3	5	1	3	1	2	3	4	1	2
19	3	2	1	3	1	1	1	1	1	4
20	3	2	1	3	1	1	1	4	1	2
21	4	1	1	2	1	2	2	4	1	2
22	1	3	1	1	1	1	1	4	3	2
23	1	4	1	3	1	1	2	2	3	2
24	1	1	1	3	1	1	1	1	1	1
25	4	2	1	2	2	1	1	1	3	2
26	2	4	1	1	1	3	3	1	2	4
27	1	3	1	1	1	1	1	1	2	2
28	4	1	1	3	2	3	1	1	2	1
29	2	1	1	2	2	2	2	2	2	2
30	1	2	1	1	2	2	2	4	2	4
31	1	5	1	1	1	3	1	2	2	2
32	3	4	1	2	1	1	1	4	2	3
33	3	1	1	2	1	1	1	4	2	2
34	1	5	1	3	1	3	2	4	2	3
35	4	1	1	1	1	2	2	2	1	
36	3	2	1		1	2	1	2	1	
37	1	1	1		2	1	3	1	1	
38	3	3	1		1	1	3	2	1	
39	2	2			2	2	3	2		
40	1	2			1	2				
41	3	1			2	3				
42	1	2			1	1				
43	3	3			1	2				

44	3	1			1	2				
45	3	1			1	1				
46	1	1			1	2				
47	1	2			1					
48	2	3			1					
49	4	1			2					
50	2	3			1					
51	3	3			1					
52	1	3			1					
53	3	3			2					
54	1	2			1					
55		2			2					
56		2			1					
57		4			2					
58		1			1					
59		3			1					
60		3			2					
61		3			2					
62		3			1					
63		5			1					
64		2			1					

Table S4: Clustering of TCGA ovarian serous cystadenocarcinoma selected datasets (instances>30)

**Platform**	**Agilent**				**Affymetrix**				
**Dataset #**	**2.2**	**3.1**	**4.1**	**5.0**	**9.8**	**11.8**	**12.8**	**13.8**	**14.8**
**Instances #**	**38**	**48**	**48**	**48**	**48**	**37**	**47**	**48**	**48**
**clusters # (QC)**	**3**	**3**	**4**	**3**	**3**	**3**	**3**	**4**	**4**
**Instance ID**	**cluster**				**cluster**				
1	2	1	2	2	1	2	1	2	2
2	1	3	2	1	3	1	2	1	3
3	1	1	1	2	1	3	1	1	2
4	1	2	1	1	1	1	3	2	1
5	1	1	1	1	1	1	1	1	2
6	2	2	2	1	3	1	3	3	3
7	1	3	4	1	2	2	2	2	1
8	1	3	3	1	3	3	1	2	1
9	1	3	3	1	1	2	2	2	1
10	1	1	1	1	1	3	1	2	3
11	2	1	1	1	1	2	1	1	1
12	1	2	4	1	2	3	1	2	2
13	1	2	1	1	1	3	1	2	3
14	1	1	3	1	1	1	2	2	2
15	3	3	2	2	1	2	1	3	2
16	1	2	2	1	1	1	1	1	3
17	1	1	1	1	3	3	2	3	1
18	1	2	1	1	2	1	1	3	1
19	2	1	3	2	1	1	1	3	4
20	1	1	3	1	1	1	1	3	1

21	1	1	1	1	1	2	1	2	2
22	3	2	2	1	1	3	1	2	1
23	1	2	4	1	2	1	1	2	1
24	1	2	1	1	3	3	2	2	3
25	3	3	1	1	1	3	3	1	5
26	4	2	3	1	1	3	3	2	3
27	1	1	3	2	3	2	2	2	4
28	1	3	4	2	1	1	2	1	2
29	1	1	1	1	2	2	3	2	3
30	1	2	1	1	1	2	1	2	2
31	2	2	1	1	3	1	1	1	2
32	1	2	2	1	1	1	2	3	5
33	1	3	2	1	1	2	2	2	3
34	1	2	3	2	2	2	3	1	2
35	3	2	2	1	3	3	1	1	2
36	1	2	1	1	1	2	1	1	1
37	1	3	4	1	1	1	3	3	4
38	1	2	1	1	1		2	3	1
39		1	2	1	2		1	2	2
40		3	4	2	2		1	1	4
41		1	1	1	1		2	1	2
42		3	2	1	1		1	3	5
43		1	2	2	1		3	2	2
44		2	1	1	2		3	1	5
45		2	2	1	2		1	3	5
46		2	2	1	1		1	2	3
47		1	1	1	1		3	1	5
48		1	2	3	1			1	
