# Supplementary material for: UFFizi: a generic platform for ranking informative features
Source: BMC Bioinformatics. 2010 Jun 3;11:300. doi: 10.1186/1471-2105-11-300 (PMC2893168; doi:10.1186/1471-2105-11-300)
Supplement: Additional file 5 — Top ranked genes, selected on all platforms of TCGA datasets. TCGA_top_ranked_genes.pdf: Top ranked genes, selected on all platforms of TCGA datasets. [file 1471-2105-11-300-S5.PDF]

Table S1: Top 10 ranked glioblastoma multiforme genes, selected on all platforms of TCGA datasets.

Genes with asterisk appear on the list of [1].

Gene name	Minimal UFF rank across platforms	References
RPS4Y1	1	The only ribosomal protein with two non identical genes. Expression correlates with jumonji (Histone demethylase JARID1D) and other Chromatin modifier proteins
SEC61G	1	[2] finds SEC61G frequently amplified, although its role in cancer development is poorly understood.
POSTN (*)	2	SAGE analysis showed that periostin was overexpressed 10-fold in glioblastoma compared to normal brain tissue [3]
ECOP	7	Amplified and overexpressed in at least a third of glioblastomas [4]
TMSL8 (*)	9	High expression in thymus / spleen. Binds actin monomers and inhibits polymerization
SERPINA3 (*)	10	Regulating hematopoietic microenvironment and imflammatory cytokine response.
COL1A2 (*)	12	differentially expressed genes in gbm [5]
NPTX2	13	Related to glioblastoma [6]
TIMP1 (*)	14	Low expression of TIMP-1 in glioblastoma predicts longer patient survival [7]
VSNL1	17	Involvement of VSNL-1 in regulating proliferative and invasive properties of neuroblastoma [8]

Table S2: Top 15 ranked genes, selected on all platforms of ovarian serous cystadenocarcinoma

Gene name	Minimal UFF rank across platforms	References
IGF2	1	Elevated IGF2 expression is a frequent event in serous ovarian cancer [9]
HOXA4	2	Increased expression appears to constitute a tumor-suppressive, homeostatic response to aberrant cell behavior, [10]
POSTN	3	SAGE analysis showed that periostin was overexpressed 10-fold in glioblastoma compared to normal brain tissue [3]
LMO3	5	Acts as an oncogene in neuroblastoma [11]
ZIC1	7	Downregulated through promoter hypermethylation in gastric cancer [12]
HOXA9	8	Ectopic expression of Hoxa9 in tumorigenic mouse OSE cells gave rise to papillary tumors resembling serous EOCs [13]
PCP4	8	-
OVGP1	9	differentiation-based indicator present in early ovarian epithelial neoplasia [14] and associated with Endometrial Cancer [15]

PON3	9	-
CXCL1	10	Generally connected to melanoma [16], but may be used in early screening of ovarian cancer [17]
IL13RA2	15	a potential biomarker and molecular target for ovarian cancer therapy [18]
MAGEA9	16	Potential marker for OV [19]
SERPINA5	17	Downregulated in serous carcinomas in contrast to Ovarian serous borderline tumors [20]
CDKN2A	18	Tumor suppressor, involved in the tumorigenesis of ovarian cancer [21]
KRT23	20	Differentially expressed in OV [22]

Table S3: Common genes to glioblastoma multiforme and ovarian serous cystadenocarcinoma

Gene	References
POSTN	SAGE analysis showed that periostin was overexpressed 10-fold in glioblastoma compared to normal brain tissue [3]
NPTX2	Related to glioblastoma [6]
GJA1	Relation to prostate and breast cancer [23], [24]
NNMT	Genetic marker for glioblastoma. (patent #7115265), upregulated in GBMs [25]
CSRP2	Transcriptional inactivity of *CSRP2* in a variety of human cancer cells [26]
SCG5	SNPs near *SCG5* are strongly associated with increased colorectal cancer risk [27]
HSPA1A	highly expressed in cancers of various origins [28]

References

1. Tso C-L, Shintaku P, Chen J, Liu Q, Liu J, Chen Z, Yoshimoto K, Mischel PS, Cloughesy TF, Liau LM *et al*: **Primary Glioblastomas Express Mesenchymal Stem-Like Properties**. *Mol Cancer Res* 2006, **4**:607.
2. Sheu JJ-C, Hua C-H, Wan L, Lin Y-J, Lai M-T, Tseng H-C, Jinawath N, Tsai M-H, Chang N-W, Lin C-F *et al*: **Functional Genomic Analysis Identified Epidermal Growth Factor Receptor Activation as the Most Common Genetic Event in Oral Squamous Cell Carcinoma**. *Cancer Res* 2009, **69**:2568.
3. Sasaki H, Yu C-Y, MeiruDai, Tam C, Loda M, Auclair D, Chen LB, Elias A: **Elevated serum periostin levels in patients with bone metastases from breast but not lung cancer**. *Breast Cancer Res and Treatment* 2003, **77**:245–252.
4. Park S, James CD: **ECop (EGFR-Coamplified and overexpressed protein), a novel protein, regulates NF-κB transcriptional activity and associated apoptotic response in an IκBα-dependent manner**. *Oncogene* 2005, **24**:2495–2502.
5. Günther HS, Schmidt NO, Phillips HS, Kemming D, Kharbanda S, Soriano R, Modrusan Z, Meissner H, Westphal M, Lamszus K: **Glioblastoma-derived stem cell-enriched cultures form distinct subgroups according to molecular and phenotypic criteria**. *Oncogene* 2008(2897–2909).
6. Carlson MRJ, Pope WB, Horvath S, Braunstein JG, Nghiemphu P, Tso C-L, Mellinghoff I, Lai A, Liau LM, Mischel PS *et al*: **Relationship between Survival and Edema in Malignant Gliomas: Role of Vascular Endothelial Growth Factor and Neuronal Pentraxin 2**. *Clinical Cancer Research* 2007, **13**:2592.
7. Aaberg-Jessen C, Christensen K, Offenberg H, Bartels A, Dreehsen T, Hansen S, Schrøder HD, Brünner N, Kristensen BW: **Low expression of tissue inhibitor of metalloproteinases-1 (TIMP-1) in glioblastoma predicts longer patient survival**. *Journal of Neuro-Oncology* 2009.
8. Xie Y, Chan H, Fan J, Chen Y, Young J, Li W, Miao X, Yuan Z, Wang H, Tam PKH *et al*: **Involvement of visinin-like protein-1 (VSNL-1) in regulating proliferative and invasive properties of neuroblastoma**. *Carcinogenesis* 2007, **28**:2122-2130.
9. Murphy12 SK, Huang1 Z, Wen1 Y, Spillman1 MA, Whitaker1 RS, Simel1 LR, Nichols1 TD, Marks23 JR, Berchuck A: **Frequent IGF2/H19 Domain Epigenetic Alterations and Elevated IGF2 Expression in Epithelial Ovarian Cancer**. *Mol cancer Res* 2006, **4**:283.

10. Otaa T, Klausena C, Salamancaa MC, Wooa HL, Leunga PCK, Auersperg N: **Expression and function of HOXA genes in normal and neoplastic ovarian epithelial cells/**. *differentiation* 2009, **77**(2):162-171.

11. Aoyama M, Ozaki T, Inuzuka H, Tomotsune D, Hirato J, Okamoto Y, Tokita H, Ohira M, Nakagawara A: **LMO3 Interacts with Neuronal Transcription Factor, HEN2, and Acts as an Oncogene in Neuroblastoma**. *Cancer Res* 2005, **65**:4587-4597.

12. Wang LJ, Jin HC, Wang X, Lamb EKY, Zhang JB, Liu X, Chan FKL, Sid JM, Sung JJY: **ZIC1 is downregulated through promoter hypermethylation in gastric cancer**. *Biochemical and Biophysical Research Communications* 2009, **379**(4):959-963

13. Cheng W, Liu J, Yoshida H, Rosen D, Naora H: **Lineage infidelity of epithelial ovarian cancers is controlled by HOX genes that specify regional identity in the reproductive tract**. *Nature Medicine* 2005, **11**:531 - 537.

14. Woo MMM, Gilks CB, Verhage HG, Longacre TA, Leung PCK, Auersperg N: **Oviductal glycoprotein, a new differentiation-based indicator present in early ovarian epithelial neoplasia and cortical inclusion cysts**. *Gynecologic Oncology* 2004, **93**(2):315-319.

15. Woo MMM, Alkushi A, Verhage HG, Magliocco AM, Leung PCK, Gilks CB, Auersperg N: **Gain of OGP, an Estrogen-Regulated Oviduct-Specific Glycoprotein, Is Associated with the Development of Endometrial Hyperplasia and Endometrial Cancer**. *Clinical Cancer Research* 2004, **10**:7958–7964.

16. Dhawan P, Richmond A: **Role of CXCL1 in tumorigenesis of melanoma**. *Journal of Leukocyte Biology* 2002, **72**:9-18.

17. Wang Q, Zhang W, Li DR, Li L: **Identification of two potential serum biomarkers for ovarian cancer and clinical validation thereof**. *Zhonghua Yi Xue Za Zhi* 2008, **88**(15):1012-1016.

18. Kioi M, Kawakami M, Shimamura T, Husain SR, Puri RK: **Interleukin-13 receptor 2 chain, a potential biomarker and molecular target for ovarian cancer therapy.** *Cancer* 2006, **107**(6):1407-1418.

19. Ferguson DA, Muenster MR, Zang Q, Spencer JA, Schageman JJ, Lian Y, Garner HR, Gaynor RB, Huff JW, Pertsemlidis A *et al*: **Selective Identification of Secreted and Transmembrane Breast Cancer Markers using Escherichia coli Ampicillin Secretion Trap**. *Cancer Res* 2005, **65**(18).

20. Sieben NLG, Oosting J, Flanagan AM, Prat J, Roemen GMJM, Kolkman-Uljee SM, Eijk Rv, Cornelisse CJ, Fleuren GJ, Engeland Mv: **Differential Gene Expression in Ovarian Tumors Reveals Dusp 4 and Serpina 5 As Key Regulators for Benign Behavior of Serous Borderline Tumors** *Journal of Clinical Oncology* 2005, **23**(29):7257-7264.

21. Niederacher D, Yan H-Y, An H-X, Bender HG, Beckmann MW: **CDKN2A gene inactivation in epithelial sporadic ovarian cancer**. *British Journal of Cancer* 1999, **80**:1920-1926.

22. Wojnarowicz PM, Breznan A, Arcand SL, Filali-Mouhim A, Provencher DM, Mes-Masson A-M, Tonin PN: **Construction of a chromosome 17 transcriptome in serous ovarian cancer identifies differentially expressed genes**. *International Journal of Gynecological Cancer* 2007, **18**(5):963-975.

23. Wang M, Berthoud VM, Beyer EC: **Connexin43 increases the sensitivity of prostate cancer cells to TNF{alpha}-induced apoptosis**. *Journal of Cell Science* 2007, **120**:320-329.

24. Pollmann M-A, Shao Q, Sandig DWL: **Connexin 43 mediated gap junctional communication enhances breast tumor cell diapedesis in culture**. *Breast Cancer Res* 2005, **7**:R522-R534.

25. Markert JM, Fuller CM, Gillespie GY, Bubien JK, Mclean LA, Hong RL, Lee K, Gullans SR, Mapstone TB, Benos DJ: **Differential gene expression profiling in human brain tumors**. *Physiol Genomics* 2001, **5**:21-33.

26. Herrmann J, Borkham-Kamphorst E, Haas U, Leur EVd, Fraga MF, Esteller M, Gressner AM, Weiskirchen R: **The expression of CSRP2 encoding the LIM domain protein CRP2 is mediated by TGF-β in smooth muscle and hepatic stellate cellsstar, open**. *Biochemical and Biophysical Research Communications* 2006, **345**(4):1526-1535.

27. Jaeger E, Webb E, Howarth K, Carvajal-Carmona L, Rowan A, Broderick P, Walther A, Spain S, Pittman A, Kemp Z *et al*: **Common genetic variants at the CRAC1 (HMPS) locus on chromosome 15q13.3 influence colorectal cancer risk**. *Nature Genetics* 2008, **40**:26 - 28.

28. Rohde M, Daugaard M, Jensen MH, Helin K, Nylandsted J, Jäättelä M: **Members of the heat-shock protein 70 family promote cancer cell growth by distinct mechanisms**. *Genes & Dev* 2005, **19**:570-582.
