# Supplementary material for: UFFizi: a generic platform for ranking informative features
Source: BMC Bioinformatics. 2010 Jun 3;11:300. doi: 10.1186/1471-2105-11-300 (PMC2893168; doi:10.1186/1471-2105-11-300)
Supplement: Additional file 6 — microRNAs, selected by UFF on the TCGA datasets. miRNA_tables.pdf: lists of microRNAs, selected by UFF on the TCGA datasets. [file 1471-2105-11-300-S6.PDF]

Table S1: MicroRNAs common to GBM and OV, selected by UFF on the TCGA datasets

[1] up or down-regulated microRNAs relative to normal tissue according to [1]

[2] MicroRNAs that affect the properties of cancer cells according to [1]

[3] down-regulated in ovarian cancer [1]

[4] Differentially expressed miRNAs in ovarian cancer tissues and cell lines [2].

microRNA	Minimal UFF rank	References
hsa-mir-181a [1]	3	tumor suppressors in human glioma cells [3]
hsa-mir-363	4	-
hsa-mir-210 [2]	6	Prognostic Factor in Breast Cancer [4]
hsa-mir-451	7	Up-regulated in multidrug resistant (MDR) cancer cell lines [5]; Involvement in resistance of the MCF-7 breast cancer cells to chemotherapeutic drug doxorubicin [6]
hsa-mir-10a	7	Connection to chronic myeloid leukemia [7] and urothelial carcinoma [8]. Shown to affect cell growth (PMID: 19074828)
hsa-mir-31[1]	8	Inhibits breast cancer metastasis [9]
hsa-mir-196a [1]	8	Potential biomarker for breast cancer [10]. Might promote colorectal cancer [11]
hsa-mir-145* [2,3]	10	Tumor suppressor in lung cancer [12]
hsa-mir-135b [1]	11	Increasing expression is correlated with decreasing expression of adenomatous polyposis coli gene during colorectal tumorigenesis. [13]
hsa-mir-10b [1,2,4]	11	Upregulated in glioblastomas [14, 15], Tumour invasion and metastasis initiated in breast cancer [16]
hsa-mir-10b* [1,2,4]	11	Upregulated in glioblastomas [14, 15], Tumour invasion and metastasis initiated in breast cancer [16]
hsa-mir-31* [1]	12	Inhibits breast cancer metastasis [9]
hsa-mir-424 [4]	18	Downregulated in ovarian cancer tissues and cell lines [2]
hsa-mir-155 [1,4]	20	Biomarker of early pancreatic neoplasia [17]
hsa-mir-222 [1,2]	25	Differentially expressed in ovarian cancer tissues and cell lines [2]
hsa-mir-30a* [1,4]	26	Differentially expressed in lung cancer tissues [18]
hsa-mir-517*	31	-

Table S2: MicroRNAs selected by UFF on the TCGA glioblastoma multiforme datasets

UFF rank	miRNA
1	hsa-mir-204
2	hsa-mir-182
3	hsa-mir-26a
4	hsa-mir-335
5	hsa-mir-149
6	hsa-mir-210
7	hsa-mir-10a

8	hsa-mir-196a
9	hsa-mir-148a
10	hsa-mir-145
11	hsa-mir-10b
12	hsa-mir-31
13	hsa-mir-451
14	hsa-mir-768-3p
15	hsa-mir-34a
16	hsa-let-7i
17	hsa-let-7b
18	hsa-mir-424
19	hsa-mir-574
20	hsa-mir-155
21	hsa-mir-193b
22	hsa-mir-365
23	hsa-mir-579
24	hsa-mir-521
25	hsa-mir-222
26	hsa-mir-30a-5p
27	hsa-mir-649
28	hsa-mir-23b
29	hsa-mir-801
30	hsa-mir-363
31	hsa-mir-517b
32	hsa-mir-221
33	hsa-mir-633
34	hsa-mir-135b
35	hsa-mir-126
36	hsa-mir-197
37	hsa-mir-345
38	hsa-mir-130a
39	hsa-mir-181a
40	hsa-mir-185
41	hsa-mir-200c
42	hsa-mir-146a
43	hsa-mir-296

Table S2: MicroRNAs selected by UFF on the TCGA ovarian serous cystadenocarcinoma datasets

UFF rank	miRNA
1	hsa-mir-205
2	hsa-mir-449a
3	hsa-mir-181a
4	hsa-mir-363
5	hcmv-mir-ul22a
6	hsa-mir-151-3p
7	hsa-mir-451
8	hsa-mir-31

9	hsa-mir-338-3p
10	hsa-mir-224
11	hsa-mir-135b
12	hsa-mir-744
13	hsa-mir-10b
14	hsa-mir-10a
15	hsa-mir-223
16	hsa-mir-605
17	hsa-mir-96
18	hsa-mir-144
19	hsa-mir-203
20	hsa-mir-375
21	hsa-mir-497*
22	hsa-mir-517c
23	hsa-mir-183
24	hsa-mir-31*
25	hsa-mir-452
26	hsa-mir-7
27	hsa-mir-34b*
28	hsa-mir-196a
29	hsa-mir-449b
30	hsa-mir-218
31	hsa-mir-150
32	hsa-mir-582-5p
33	hcmv-mir-us25-1*
34	hsa-mir-630
35	hsa-mir-301a
36	hsa-mir-517*
37	hsa-mir-625
38	hsa-mir-210
39	hsa-mir-184
40	hsa-mir-551b
41	hsa-mir-30b*
42	hsa-mir-378
43	hsa-mir-155
44	hsa-mir-95
45	hsa-mir-769-5p
46	hsa-mir-483-3p
47	hsa-mir-222
48	hsa-mir-30a*
49	hsa-mir-409-5p
50	hsa-mir-145*
51	hsa-mir-30a
52	hsa-mir-34c-5p
53	hsa-mir-10b*
54	hsa-mir-503
55	hsa-mir-29a
56	hsa-mir-514
57	hsa-mir-768-5p
58	hsa-mir-29b
59	hsa-mir-130b

60	hsa-mir-424
61	hsa-mir-532-5p
62	hsa-mir-486-5p
63	hsa-mir-429

References

1. Lee YS, Dutta A: **MicroRNAs in cancer**. *Annual Review of Pathology: Mechanisms of Disease* 2008, **4**:199-227.
2. Dahiya N, Sherman-Baust CA, Wang TL, Davidson B, Shih Ie M, Zhang Y, Wood W, 3rd, Becker KG, Morin PJ: **MicroRNA expression and identification of putative miRNA targets in ovarian cancer**. *PLoS One* 2008, **3**(6):e2436.
3. Shi L, Cheng Z, Zhang J, Li R, Zhao P, Fu Z, You Y: **hsa-mir-181a and hsa-mir-181b function as tumor suppressors in human glioma cells**. *Brain Res* 2008, **1236**:185-193.
4. Camps C, Buffa FM, Colella S, Moore J, Sotiriou C, Sheldon H, Harris AL, Gleadle JM, Ragoussis J: **hsa-miR-210 Is Induced by Hypoxia and Is an Independent Prognostic Factor in Breast Cancer**. *Clinical Cancer Res* 2008, **14**:1340.
5. Zhu H, Wu H, Liu X, Evans BR, Medina DJ, Liu CG, Yang JM: **Role of MicroRNA miR-27a and miR-451 in the regulation of MDR1/P-glycoprotein expression in human cancer cells**. *Biochem Pharmacol* 2008, **76**(5):582-588.
6. Kovalchuk O, Filkowski J, Meservy J, Ilnytskyy Y, Tryndyak VP, Chekhun VF, Pogribny IP: **Involvement of microRNA-451 in resistance of the MCF-7 breast cancer cells to chemotherapeutic drug doxorubicin**. *Mol Cancer Ther* 2008, **7**(7):2152-2159.
7. Agirre X, Jiménez-Velasco A, José-Enériz ES, Garate L, Bandrés E, Cordeu L, Aparicio O, Saez B, Navarro G, Vilas-Zornoza A *et al*: **Down-Regulation of hsa-miR-10a in Chronic Myeloid Leukemia CD34+ Cells Increases USF2-Mediated Cell Growth**. *Mol Cancer Res* 2008, **6**:1830.
8. Veerla S, Lindgren D, Kvist A, Frigyesi A, Staaf J, Persson H, Liedberg F, Chebil G, Gudjonsson S, Borg Å *et al*: **MiRNA expression in urothelial carcinomas: Important roles of miR-10a, miR-222, miR-125b, miR-7 and miR-452 for tumor stage and metastasis, and frequent homozygous losses of miR-31**. *International Journal of Cancer* 2008, **124**(9):2236-2242.
9. Valastyan S, Reinhardt F, Benaich N, Calogrias D, Szasz AM, Wang ZC, Brock JE, Richardson AL, Weinberg RA: **A pleiotropically acting microRNA, miR-31, inhibits breast cancer metastasis**. *Cell* 2009, **137**(6):1032-1046.
10. Hoffman AE, Zheng T, Yi C, Leaderer D, Weidhaas J, Slack F, Zhang Y, Paranjape T, Zhu Y: **microRNA miR-196a-2 and Breast Cancer: A Genetic and Epigenetic Association Study and Functional Analysis**. *Cancer Res* 2009, **69**:5970.
11. Schimanski CC, Frerichs K, Rahman F, Berger M, Lang H, Galle PR, Moehler M, Gockel I: **High miR-196a levels promote the oncogenic phenotype of colorectal cancer cells**. *World J Gastroenterol* 2009, **15**(17):2089–2096.
12. Cho WCS, Chowa ASC, Au JSK: **Restoration of tumour suppressor hsa-miR-145 inhibits cancer cell growth in lung adenocarcinoma patients with epidermal growth factor receptor mutation**. *European Journal of Cancer* 2009, **In Press**.
13. Nagel R, le Sage C, Diosdado B, van der Waal M, Oude Vrielink JA, Bolijn A, Meijer GA, Agami R: **Regulation of the adenomatous polyposis coli gene by the miR-135 family in colorectal cancer**. *Cancer Res* 2008, **68**(14):5795-5802.
14. Ciafre SA, Galardi S, Mangiola A, Ferracin M, Liu CG, Sabatino G, Negrini M, Maira G, Croce CM, Farace MG: **Extensive modulation of a set of microRNAs in primary glioblastoma**. *Biochem Biophys Res Commun* 2005, **334**(4):1351-1358.
15. Silber J, Lim DA, Petritsch C, Persson AI, Maunakea AK, Yu M, Vandenberg SR, Ginzinger DG, James CD, Costello JF *et al*: **miR-124 and miR-137 inhibit proliferation of glioblastoma multiforme cells and induce differentiation of brain tumor stem cells**. *BMC Med* 2008, **6**:14.
16. Ma L, Teruya-Feldstein J, Weinberg RA: **Tumour invasion and metastasis initiated by microRNA-10b in breast cancer**. *Nature* 2007, **449**(7163):682-688.
17. Habbe N, Koorstra J-BM, Mendell JT, Offerhaus GJ, Ryu JK, Feldmann G, Mullendore ME, Goggins MG, Hong S-M, Maitra A: **MicroRNA miR-155 is a biomarker of early pancreatic neoplasia**. *Cancer Biology & Therapy* 2009, **8**(4):340 - 346.
18. Yanaihara N, Caplen N, Bowman E, Seike M, Kumamoto K, Yi M, Stephens RM, Okamoto A, Yokota J, Tanaka T *et al*: **Unique microRNA molecular profiles in lung cancer diagnosis and prognosis**. *Cancer Cell* 2006, **9**(3):189-198.
