# Supplementary material for: UFFizi: a generic platform for ranking informative features
Source: BMC Bioinformatics. 2010 Jun 3;11:300. doi: 10.1186/1471-2105-11-300 (PMC2893168; doi:10.1186/1471-2105-11-300)
Supplement: Additional file 7 — List of datasets used in this paper. Datasets.pdf: List of datasets used in this paper. GEO, Gene Expression Omnibus; TCGA, The Cancer Genome Atlas; GBM, glioblastoma multiforme; OV, ovarian serous cystadenocarcinoma. [file 1471-2105-11-300-S7.PDF]

Table S1: List of datasets used in this paper.

GEO= Gene Expression Omnibus, TCGA=The Cancer Genome Atlas, GBM=glioblastoma multiforme, OV= ovarian serous cystadenocarcinoma.

Dataset type	Dataset name	Platform	Origin
Melanoma	GSE3189	Affymetrix Human Genome U133A Array	GEO
HIV	GSE6740	Affymetrix Human Genome U133A Array	GEO
Hepatitis C	GSE11190	Affymetrix Human Genome U133A plus 2.0 Array	GEO
GBM	AgilentG4502A_07_1.1.3.0	Agilent 244K Custom Gene Expression G4502A-07-1	TCGA
GBM	AgilentG4502A_07_1.2.2.0	Agilent 244K Custom Gene Expression G4502A-07-1	TCGA
GBM	AgilentG4502A_07_1.3.3.0	Agilent 244K Custom Gene Expression G4502A-07-1	TCGA
GBM	AgilentG4502A_07_1.4.3.0	Agilent 244K Custom Gene Expression G4502A-07-1	TCGA
GBM	AgilentG4502A_07_2.5.3.0	Agilent 244K Custom Gene Expression G4502A-07-2	TCGA
GBM	AgilentG4502A_07_2.6.2.0	Agilent 244K Custom Gene Expression G4502A-07-2	TCGA
GBM	AgilentG4502A_07_2.7.2.0	Agilent 244K Custom Gene Expression G4502A-07-2	TCGA
GBM	AgilentG4502A_07_2.8.1.0	Agilent 244K Custom Gene Expression G4502A-07-2	TCGA
GBM	AgilentG4502A_07_2.10.0.0	Agilent 244K Custom Gene Expression G4502A-07-2	TCGA
GBM	HT_HG-U133A.1.4.0	Affymetrix HT Human Genome U133 Array Plate Set	TCGA
GBM	HT_HG-U133A.2.4.0	Affymetrix HT Human Genome U133 Array Plate Set	TCGA
GBM	HT_HG-U133A.3.4.0	Affymetrix HT Human Genome U133 Array Plate Set	TCGA
GBM	HT_HG-U133A.4.4.0	Affymetrix HT Human Genome U133 Array Plate Set	TCGA
GBM	HT_HG-U133A.5.4.0	Affymetrix HT Human Genome U133 Array Plate Set	TCGA
GBM	HT_HG-U133A.6.4.0	Affymetrix HT Human Genome U133 Array Plate Set	TCGA
GBM	HT_HG-U133A.7.4.0	Affymetrix HT Human Genome U133 Array Plate Set	TCGA
GBM	HT_HG-U133A.8.4.0	Affymetrix HT Human Genome U133 Array Plate Set	TCGA
GBM	HT_HG-U133A.11.4.0	Affymetrix HT Human Genome U133 Array Plate Set	TCGA
GBM	H-miRNA_8x15Kv2.1.0.0	Agilent Human miRNA Microarray Rel12.0	TCGA
GBM	H-miRNA_8x15K.1.2.0	Agilent 8 x 15K Human miRNA-specific microarray	TCGA

GBM	H-miRNA_8x15K.2.2.0	Agilent 8 x 15K Human miRNA-specific microarray	TCGA
GBM	H-miRNA_8x15K.3.2.0	Agilent 8 x 15K Human miRNA-specific microarray	TCGA
GBM	H-miRNA_8x15K.4.1.0	Agilent 8 x 15K Human miRNA-specific microarray	TCGA
GBM	H-miRNA_8x15K.5.1.0	Agilent 8 x 15K Human miRNA-specific microarray	TCGA
GBM	H-miRNA_8x15K.6.1.0	Agilent 8 x 15K Human miRNA-specific microarray	TCGA
GBM	H-miRNA_8x15K.7.1.0	Agilent 8 x 15K Human miRNA-specific microarray	TCGA
GBM	H-miRNA_8x15K.8.0.0	Agilent 8 x 15K Human miRNA-specific microarray	TCGA
GBM	H-miRNA_8x15K.10.1.0	Agilent 8 x 15K Human miRNA-specific microarray	TCGA
OV	AgilentG4502A_07_3.1.2.0	Agilent 244K Custom Gene Expression G4502A-07-3	TCGA
OV	AgilentG4502A_07_3.2.2.0	Agilent 244K Custom Gene Expression G4502A-07-3	TCGA
OV	AgilentG4502A_07_3.3.1.0	Agilent 244K Custom Gene Expression G4502A-07-3	TCGA
OV	AgilentG4502A_07_3.4.1.0	Agilent 244K Custom Gene Expression G4502A-07-3	TCGA
OV	AgilentG4502A_07_3.5.0.0	Agilent 244K Custom Gene Expression G4502A-07-3	TCGA
OV	AgilentG4502A_07_3.6.0.0	Agilent 244K Custom Gene Expression G4502A-07-3	TCGA
OV	H-miRNA_8x15Kv2.1.0.0	Agilent Human miRNA Microarray Rel12.0	TCGA
OV	H-miRNA_8x15Kv2.2.2.0	Agilent Human miRNA Microarray Rel12.0	TCGA
OV	H-miRNA_8x15Kv2.3.0.0	Agilent Human miRNA Microarray Rel12.0	TCGA
OV	H-miRNA_8x15Kv2.4.0.0	Agilent Human miRNA Microarray Rel12.0	TCGA
OV	H-miRNA_8x15Kv2.5.0.0	Agilent Human miRNA Microarray Rel12.0	TCGA
OV	H-miRNA_8x15Kv2.6.0.0	Agilent Human miRNA Microarray Rel12.0	TCGA
OV	H-miRNA_8x15Kv2.7.0.0	Agilent Human miRNA Microarray Rel12.0	TCGA
OV	HT_HG-U133A.9.8.0	Affymetrix HT Human Genome U133 Array Plate Set	TCGA
OV	HT_HG-U133A.11.8.0	Affymetrix HT Human Genome U133 Array Plate Set	TCGA
OV	HT_HG-U133A.12.8.0	Affymetrix HT Human Genome U133 Array Plate Set	TCGA
OV	HT_HG-U133A.13.8.0	Affymetrix HT Human Genome U133 Array Plate Set	TCGA
OV	HT_HG-U133A.14.8.0	Affymetrix HT Human Genome U133 Array Plate Set	TCGA

OV	HT_HG-U133A.15.8.0	Affymetrix HT Human Genome U133 Array Plate Set	TCGA
